# 3D Topography of the Young Adult Anal Sphincter Complex Reconstructed from Undeformed Serial Anatomical Sections

**DOI:** 10.1371/journal.pone.0132226

**Published:** 2015-08-25

**Authors:** Yi Wu, Noshir F. Dabhoiwala, Jaco Hagoort, Jin-Lu Shan, Li-Wen Tan, Bin-Ji Fang, Shao-Xiang Zhang, Wouter H. Lamers

**Affiliations:** 1 Tytgat Institute for Liver and Intestinal Research, Academic Medical Center, University of Amsterdam, Amsterdam, the Netherlands; 2 Department of Anatomy & Embryology, Academic Medical Center, University of Amsterdam, Amsterdam, the Netherlands; 3 Institute of Computing Medicine, Third Military Medical University, Chongqing, 400038, China; University General Hospital of Heraklion and Laboratory of Tumor Cell Biology, School of Medicine, University of Crete, GREECE

## Abstract

**Background:**

Pelvic-floor anatomy is usually studied by artifact-prone dissection or imaging, which requires prior anatomical knowledge. We used the serial-section approach to settle contentious issues and an interactive 3D-pdf to make the results widely accessible.

**Method:**

3D reconstructions of undeformed thin serial anatomical sections of 4 females and 2 males (21–35y) of the Chinese Visible Human database.

**Findings:**

Based on tendinous septa and muscle-fiber orientation as segmentation guides, the anal-sphincter complex (ASC) comprised the subcutaneous external anal sphincter (EAS) and the U-shaped puborectal muscle, a part of the levator ani muscle (LAM). The anococcygeal ligament fixed the EAS to the coccygeal bone. The puborectal-muscle loops, which define the levator hiatus, passed around the anorectal junction and inserted anteriorly on the perineal body and pubic bone. The LAM had a common anterior attachment to the pubic bone, but separated posteriorly into puborectal and “pubovisceral” muscles. This pubovisceral muscle was bilayered: its internal layer attached to the conjoint longitudinal muscle of the rectum and the rectococcygeal fascia, while its outer, patchy layer reinforced the inner layer. ASC contraction makes the ano-rectal bend more acute and lifts the pelvic floor. Extensions of the rectal longitudinal smooth muscle to the coccygeal bone (rectococcygeal muscle), perineal body (rectoperineal muscle), and endopelvic fascia (conjoint longitudinal and pubovisceral muscles) formed a “diaphragm” at the inferior boundary of the mesorectum that suspended the anorectal junction. Its contraction should straighten the anorectal bend.

**Conclusion:**

The serial-section approach settled contentious topographic issues of the pelvic floor. We propose that the ASC is involved in continence and the rectal diaphragm in defecation.

## Introduction

The anatomical body of knowledge is usually stated to have evolved completely. Nevertheless, considerable differences are noticed in modern anatomical atlases with respect to the architecture of the levator and sphincter ani complex, and the structures that affix the rectum to its surroundings. In fact, the structural features of the pelvic floor were bones of contention for well over a century and continue to attract attention [1–8]. Such disparate information, which negatively affects the correct interpretation of imaging data, functional tests, and surgical planning, can probably be accounted for by the deep position and, hence, limited accessibility of the pelvic floor, and the near total reliance on dissection. Although dissection is clinically highly relevant, if it were only as the tool of surgeons, this tool depends almost entirely on recognition at first sight and cannot be reversed. As a result, dissection is prone to generating artifacts. A different approach is, therefore, necessary to confirm or correct the anatomic descriptions of the anal sphincter complex.

The obvious alternative is a sectional approach. This approach does not suffer from limited accessibility and (stored) sections can be studied repeatedly, but “real” sectioning causes deformation and loss of alignment of the sections, and poses limitations on the size of the specimen. These drawbacks do not apply to a sectional approach that is based on serial “shaving” of thin layers from a frozen specimen and photographic registration of the “new” surface. This technique was pioneered by Ackerman *c*.*s*. [9] and implemented in our Chongqing laboratory [10,11]. With this approach, most structures can be unambiguously identified by their natural color and separated from adjacent structures by connective-tissue sheaths. Based on an in-depth study of 6 specimens, we revisited the contentious issues and generated a detailed interactive 3D topographic model of the pelvic floor.

## Material and Methods

### Specimens

Four female (CVH2, -4, -5 and CVO) and 2 male Chinese specimens (CVH1 and -3) were studied. All cadavers were enrolled in the body donation program of the Chinese Visible Human (CVH) project. The study was approved by the Ethics Committee of Third Military Medical University (Chongqing, China). Written informed consent was obtained from the donors or their family members. Although little is known about the medical history of the specimens, CT and MRI scans did not reveal pathology. Furthermore, no pathologically changed structures were observed in the sections of the pelvis. Some available biometric details are given in S1A Table. The preparation of the CVH images followed the protocols described earlier [12] and was described elsewhere [10,11]. We also studied both specimens of the Visible Human Project (VHP) [9], but these specimens differed substantially from the Chinese specimens, because the male individual had practiced body building, while the female was postmenopausal. We studied only the true pelvis, taking the pelvic bones as lateral boundaries, the perineum and anus as inferior boundary, and the peritoneum covering the pelvic organs as upper boundary. We use superior, inferior, anterior, and posterior for description of topographical relations.

### Segmentation

Pelvic structures with clear boundaries (bone, muscles, veins, hollow organs) were segmented semi-automatically with Amira software tools ((http://www.amiravis.com, version 5.3.3). Thereafter, structures with less obvious boundaries, such as the different parts of the levator ani and rectal diaphragm, were segmented manually. Criteria for segmentation were (thin) fibrous tissue fascia, and differences in tissue architecture and color. Furthermore, we always inspected the corresponding sections in all other CVH specimens before proceeding to segmentation. When manually segmenting, we always identified and delineated the easiest identifiable (parts of a) structure first and then proceeded to the less clear-cut portions.

### Three-dimensional reconstruction

Segmented structures were reconstructed using the 3D surface-rendering and-smoothing tools of Amira software. The data from CVH5 were exported to Adobe Acrobat 9 Pro Extended (http://www.adobe.com) to generate an interactive 3D-pdf file [13]. All 47 structures identified (S1C Table) are represented in this 3D reconstruction in an interactive 3D-pdf file format that is provided on line and that can be displayed on almost all PCs (S1–S3 Figs). This 3D pdf allows the reader to three-dimensionally visualize all structures separately or in self-chosen combinations to inspect the shape, relative size, and topographical relations of the respective structures. All 93 sections of specimen CVH5 that were used to build the reconstruction shown in S3 Fig are available as S4 Fig.

## Results

### Physical differences between specimens

Apart from their relations to the sex-specific organs, the pelvic floor and anorectum were remarkably similar in both sexes. The position of the uteri of CVH2 and CVH5 was anteverted, whereas those of CVH4 and CVO occupied the in Orientals common retroverted position. The biometric data of its position [14] indicate that the post-mortem levator hiatus was ~61 mm longer and ~23 mm narrower, and had descended 3–5 mm more (S1B Table) than measured in life Chinese females of the same age [15,16]. We ascribe these differences to the relaxation of muscles, including those of the pelvic floor, in cadavers.

### Levator ani muscles

**Fig 1:**
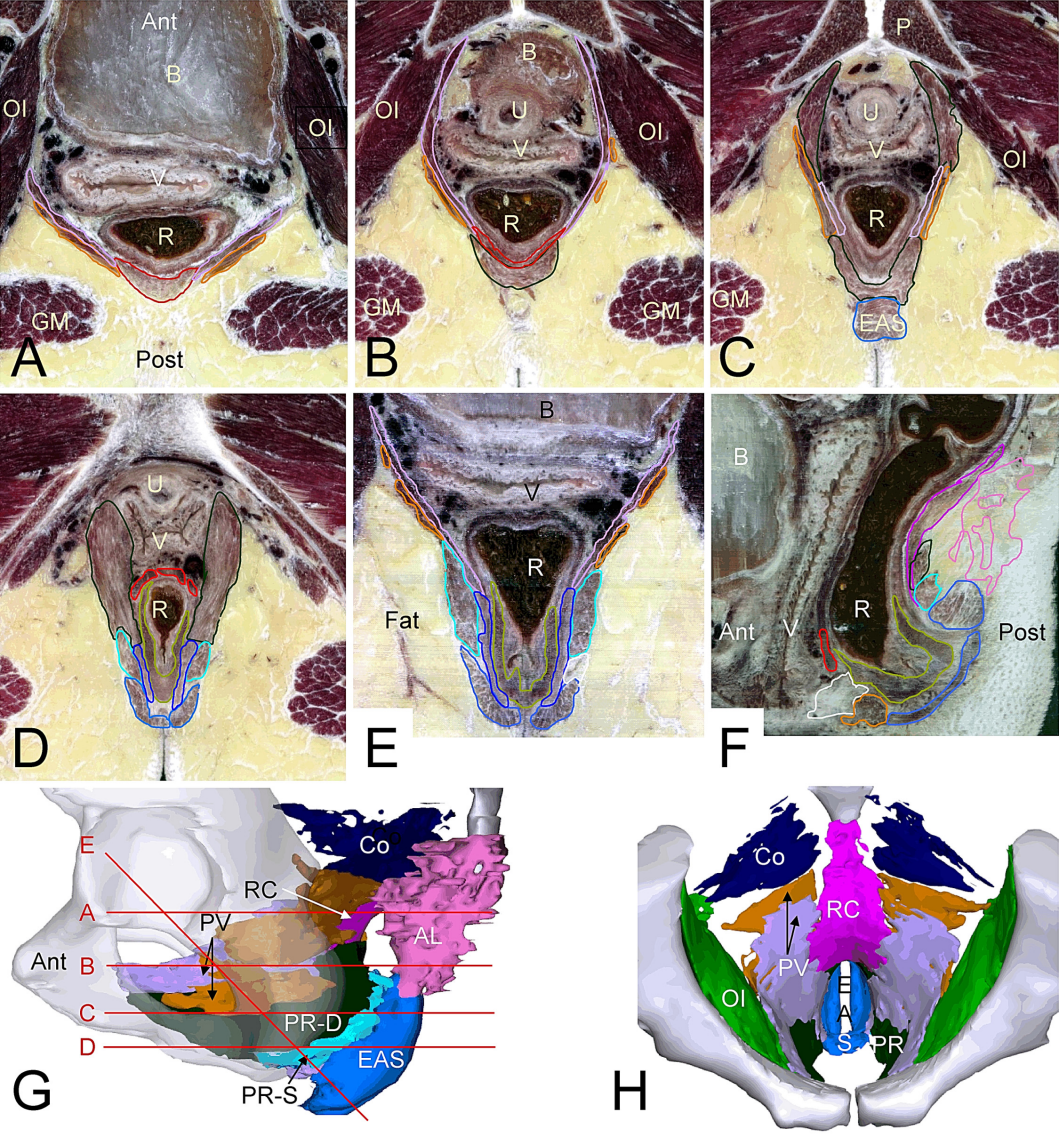
The architecture of the levator ani muscle. Panel G shows the positions of the transverse sections in panels A-D and that of obliquely sectioned panel E (calculated from reconstruction). The sections show the bilayered make-up of the pubovisceral muscle (A-C) and its attachment to the rectococcygeal muscle (A,B), the extension of the pubovisceral and deep layer of the puborectal muscles to the pubic bone (B,C), the external anal sphincter, the superficial layer of the puborectal muscle, and the conjoint longitudinal muscle of the rectum (C-E), and the rectoperineal muscle (D). Panel E visualizes the successive layers of the anal-sphincter complex, while panel F, a calculated midsagittal section of this specimen, shows the muscles surrounding the anal canal. Panels G and H show reconstructions of the levator and sphincter ani muscles as seen from the left (G) and anterosuperior (H). The color code of the (outlined) structures is shown in S2 Fig and sections without outlines S5 Fig. Abbreviations: AL, anococcygeal ligament; Ant, anterior; B, bladder; Co, coccygeal muscle; EAS, external anal sphincter; GM, gluteus maximus muscle; Post, posterior; PR-D, puborectal muscle, deep portion; PR-S, puborectal muscle, superficial portion; PV, pubovisceral muscle with deep (purple) and superficial (orange) layers; RC, rectococcygeal muscle; U, urethra; R, rectum; V, vagina.

In all specimens, the Levator ani muscle (LAM) was a funnel-shaped structure with its superior attachment on the pubic bone anteriorly, and the internal obturator muscle and the ischial spine laterally (Fig 1G and 1H). The fibrous attachment of the LAM to the internal obturator muscle (its “tendinous” arch) was remarkably inconspicuous. The volume of LAM was similar in females and males (S1B Table). Posterosuperiorly, the LAM bordered the coccygeal muscle. The posterosuperior portion of the LAM and the coccygeal muscle contained many fibrous parts. The anterior portion of the LAM was well developed, but we observed no perimysial septa between puborectal and pubococcygeal portions that would allow a separation as shown in virtually all textbooks. More posteriorly, however, fibrous septa and the orientation of muscle fibers allowed us to identify two main muscle groups, the puborectal muscle inferiorly and the “pubovisceral” muscle medially and superiorly (Fig 1C, 1D and 1H). The pubovisceral muscle comprised the pubo- and iliococcygeal muscles that are shown in most atlases (S1D Table), and was renamed pubovisceral muscle because of its role in the suspension of the distal gut (see next paragraph). The topography of the puborectal muscle did not differ from that shown in textbooks and coincided with the “deep portion” of the external anal sphincter (EAS), that is, both muscles proved to be one-and-the-same structure. Like the puborectal muscle, the “superficial portion” of the EAS was an anteriorly open sling that merged near its anterior attachment on the perineal body with the puborectal muscle. Our serial-sectioning approach, therefore, demonstrated that the deep and superficial portions of the EAS had a similar shape and differed only in that the superficial part was the more inferior one and did not reach as far anteriorly. Based on their similar shape and topography, they were renamed the “deep” and “superficial” parts of the puborectal portion of the LAM, respectively. Their topography is further detailed in the section on the EAS.

The pubovisceral muscle extended from the pubic bone, tendinous arch and the ischial spine backwards with medial and lateral layers (Fig 1A and 1B). The medial layer was continuous inferiorly with the perineal body and the conjoint longitudinal muscle of the rectum, and attached posteriorly to the fascia surrounding the rectococcygeal muscle (Fig 1H). The lateral layer consisted of discontinuous muscle sheets, which appeared to reinforce the medial layer locally and also attached to the rectococcygeal fascia (Fig 1A and 1E). The muscle fiber orientation of the anterior portion of the LAM inclined ~30° with respect to the horizontal plane and that of the puborectal and pubovisceral muscles in the narrow part of the funnel, laterally to the rectum, ~45°, while that of the superior portion of the pubovisceral muscle was predominantly oriented in the horizontal plane.

### Sphincter-ani muscle complex

**Fig 2:**
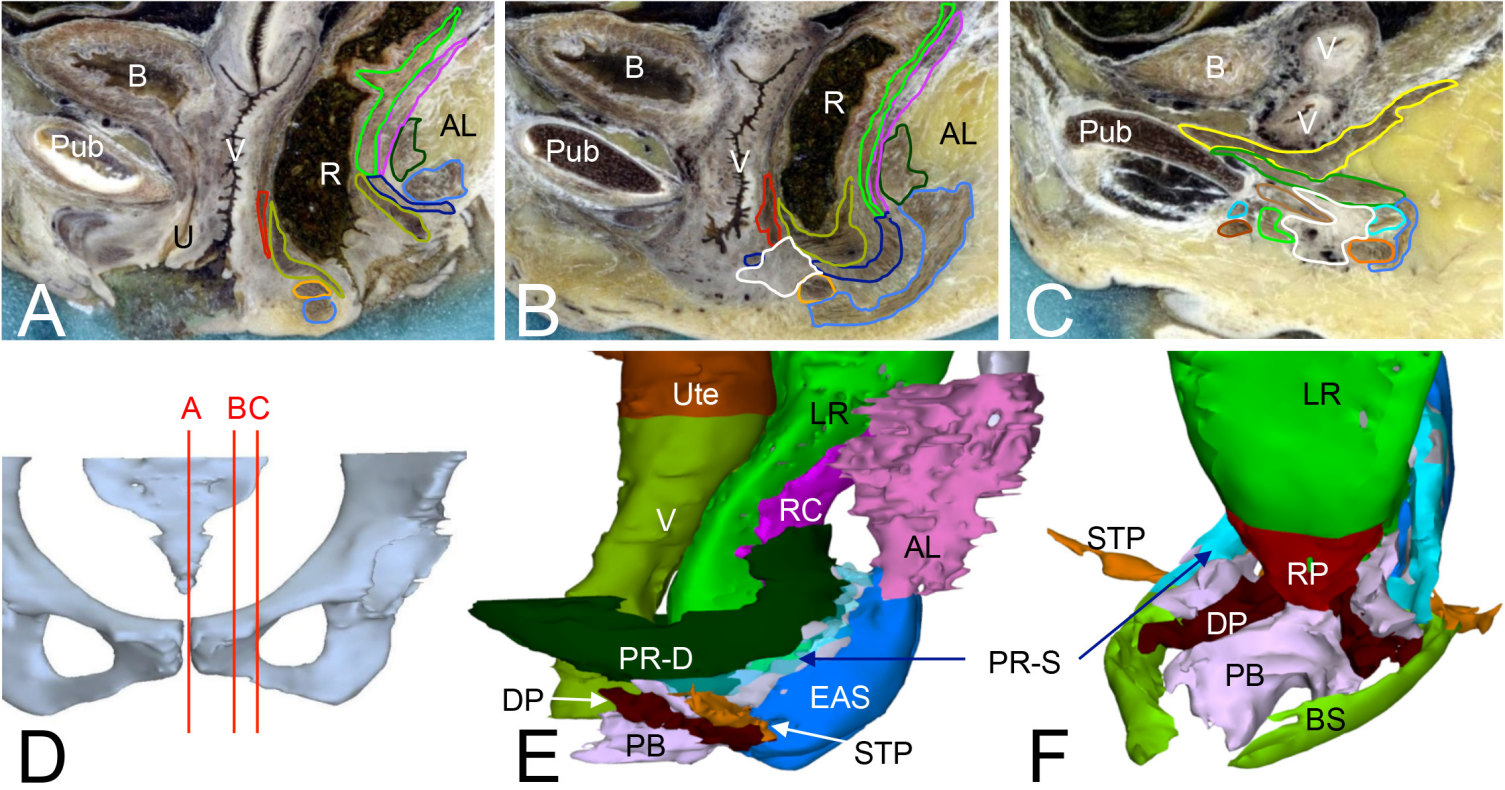
The architecture of the anal sphincter complex and perineal body. Panel D shows the positions of the sagittal sections of panels A-C. The sections show the position of muscles and the perineal body around the rectum (A: midsagittal; C: most lateral). Panels E and F show a left-lateral and left-anterior view of the muscles surrounding the rectum and vagina that have insertions on the perineal body. The rectococcygeal muscle and anococcygeal ligament are shown in panel E. The bulbospongious muscle is not shown in panel E so as not to hide the perineal body and deep perineal muscle. For the same reason, the vagina and uterus are not shown in panel F. The color code of the (outlined) structures is shown in S2 Fig and sections without outlines in S6 Fig. Abbreviations: AL, anococcygeal ligament; B, bladder; BS, bulbospongious muscle; DP, deep perineal muscle; EAS, external anal sphincter; LR, longitudinal smooth muscle of rectum; PB, perineal body; PR-D, puborectal muscle, deep portion; PR-S, puborectal muscle, superficial portion; Pub: pubic bone; RC, rectococcygeal muscle; RP, rectoperineal muscle; STP, superficial transverse perineal muscle; U, urethra; Ute, uterus; UVS, urethrovaginal sphincter; R, rectum; V, vagina.

Using muscle-fiber orientation and tendinous tissue between muscle layers as guides, we identified the standard three (subcutaneous, superficial and deep) portions of the EAS (Figs 1C–1E and 2A–2C). As described in the previous section, the deep portion of the EAS and the puborectal muscle were one-and-the-same structure and designated the “deep layer of the puborectal muscle” (S1D Table). This deep layer was continuous with its contralateral part behind the anorectal bend. Its most inferior portion also attached to the perineal body (Fig 2E). The superficial portion of the EAS formed, like its deep counterpart, an anteriorly open sling around the anorectal bend. Anteriorly, this superficial portion became more and more apposed to the deep portion of the puborectal muscle to insert on the perineal body, just posteriorly and inferiorly to the attachment of the deep portion (Fig 2E). Based on its topography, we, therefore, denote this structure as the superficial layer of the puborectal muscle rather than the EAS (S1D Table). The superficial and deep portions of the puborectal muscle were best discernible posteriorly, where the deep portion formed a ~16 mm wide sleeve on the posterior surface of the anorectal junction, whereas the superficial portion formed a more-or-less round muscle band (diameter ~10 mm) on the posterior and inferior surface of the deep portion (Fig 2A–2C).

Well-developed fibrous tissue continuous with the superficial perineal fascia (a.k.a. transverse fibrous septum of the ischioanal fossa) separated the subcutaneous portion of the EAS from the superficial portion of the puborectal muscle. The subcutaneous portion enveloped the rectum and internal anal sphincter completely (Fig 2A–2C), thus forming the only “real” sphincter. Hence, we denoted this portion the “EAS proper”. The sphincter was markedly oblong. On its anterior side, the EAS covered the crossing fibers of the superficial transverse perineal and bulbospongious muscles superficially, and, therefore, only indirectly contacted the perineal body (Fig 2A–2C). The EAS in males differed from that in females in the long subcutaneous spur that extended anteriorly towards the base of the scrotum. Posteriorly, the anococcygeal ligament, a mesh of well-developed ligamentous tissue surrounding small adipose-tissue compartments, connected the EAS to the coccygeal bone (Fig 2E).

The internal anal sphincter, which is the thick distal continuation of the circular smooth-muscle layer of the rectum, extended from the superior end of the anal columns and the anorectal junction superiorly to the intermuscular groove or “white line of Hilton” (Fig 2A–2C) inferiorly. This line is laterally continuous with separating the puborectal muscle from the EAS. It is of note that we did not observe an internal venous plexus at the anorectal junction in any of the specimens.

**Fig 3:**
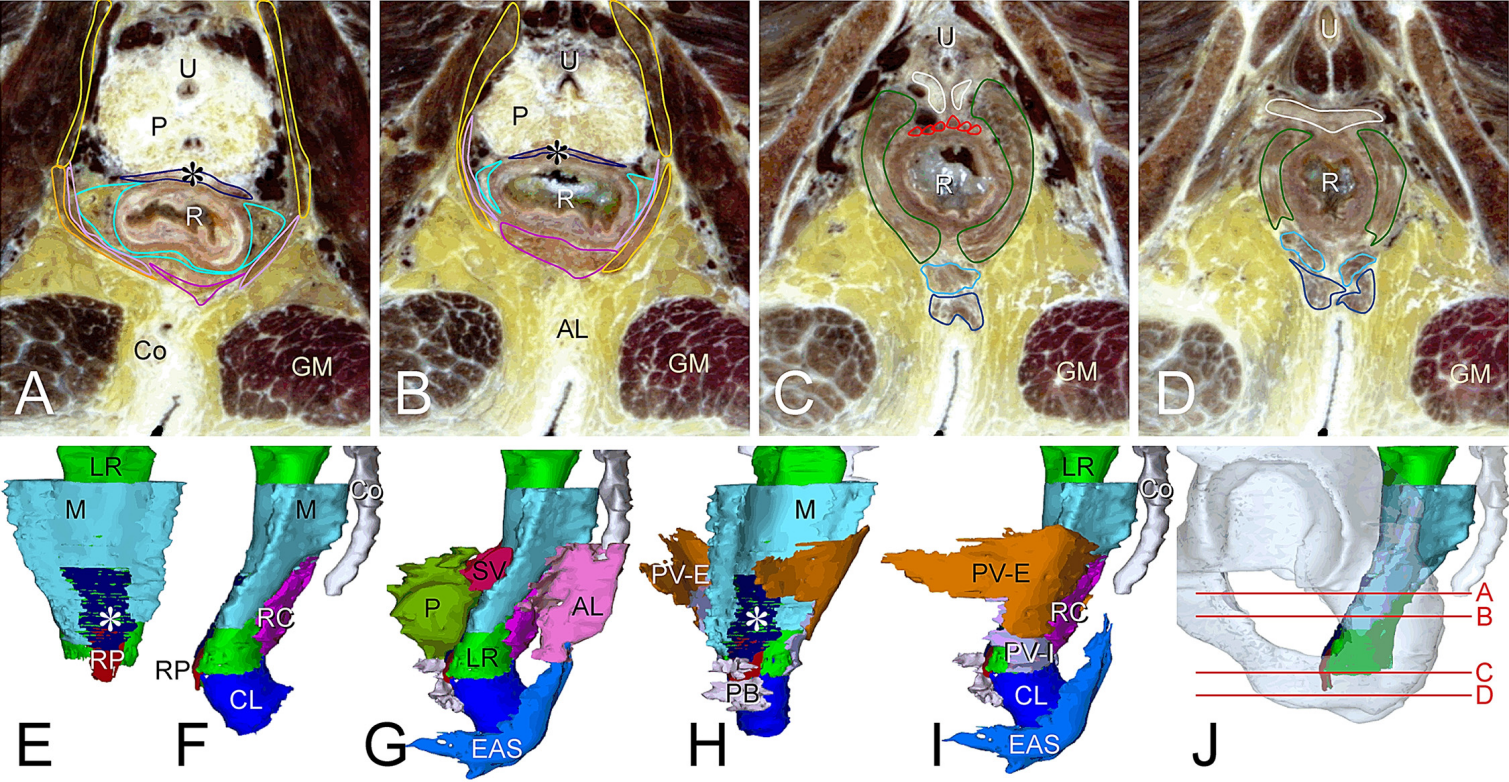
Shape and topographic relations of the mesorectum, rectal diaphragm, and perineal body in the male. Panel J shows the positions of the transverse sections in panels A-D. The sections show the position of the rectococcygeal (A,B) and rectoperineal (C) muscles and perineal body (C,D). Panels E-I show anterior (E), left-lateral (F,G,I), and anterolateral (H) views of the mesorectum (E-I) and rectal diaphragm (H,I), and perineal body (G-I). Note anterior absence of mesorectum at Denonvilliers’ fascia (E), the rectoperineal and rectococcygeal muscles forming the inferior boundary of the mesorectum (E,F) and the connection of the pubovisceral muscle with the conjoint longitudinal muscle of the rectum (H,I). The color code of the (outlined) structures is shown in S2 Fig and sections without outlines in S7 Fig. Abbreviations: AL, anococcygeal ligament; Co, coccygeal bone, CL, conjoint longitudinal muscle of rectum; EAS, external anal sphincter; GM, gluteus maximus muscle; LR, longitudinal smooth muscle of rectum; M, mesorectum; P, prostate; PB, perineal body; PV, pubovisceral muscle with deep (purple) and superficial (orange) layers; RC, rectococcygeal muscle; RP, rectoperineal muscle; SV, seminal vesicles, U, urethra; R, rectum; *, Denonvilliers’ fascia.

The conjoint longitudinal muscle, the distal continuation of the longitudinal smooth-muscle layer of the rectum and the medial layer of the pubovisceral muscle, extended between the internal and external anal sphincters. Distally, its fibers intermingled with the EAS (Fig 1D and 1E). Posteriorly and laterally, the conjoint longitudinal muscle reached up to the anorectal junction. Anteriorly, however, the conjoint longitudinal muscle could only be identified inferiorly, because more superiorly, thick fiber bundles of the longitudinal smooth muscle of the rectum did not follow the bend of the anorectal junction, but continued downward. Only a much thinner layer of the longitudinal smooth muscle followed the wall of the rectum through the bend in a posteroinferior direction. The thick anterior longitudinal muscle fibers are known as the “rectovaginal” or “rectourethral”, or independent of the sex, the “rectoperineal” muscle, and usually divided into a few strands (Figs 1D, 2A, 2B, 2F and 3C, 3E–3J). The rectoperineal muscle was widest superiorly and tapered off towards its insertion on the perineal body.

While the rectoperineal muscle was the anterior extension of the longitudinal smooth muscle from the anorectal junction to the perineal body, the flat and triangular rectococcygeal muscle was the posterior extension of the longitudinal smooth muscle from the anorectal junction to the presacral fascia in front of the coccygeal vertebrae (Figs 1A, 1B, 1G, 1H, 2A, 2B, 2E and 3A, 3B, 3F–3I). Its junction with the longitudinal smooth muscle layer was often marked by a thin tendon (Fig 2G). From here, the rectococcygeal muscle passed the puborectal muscles anteriorly and superiorly.

### Adnexa of rectum

The mesorectum—the perirectal space that is filled mostly with fat, lymph nodes, but contains remarkably few veins, and is surrounded by the rectal adventitia—was identifiable in all specimens (Fig 3A, 3B and 3E–3J). Three-dimensionally, the mesorectum was an anteriorly concave, inverted cone. Between the coccyx and the rectal adventitia, a presacral space surrounded by connective tissue and containing some fat tissue and vessels was present. Superiorly, the mesorectum surrounded the rectum on all sides. Inferior to the rectouterine or rectovesical recess, the rectum was a midline structure that was apposed to the vagina or prostate. Here, the (anterior) adventitia of the rectum and the posterior adventitia of the vagina or prostate together form “Denonvilliers’ fascia” (Fig 3A, 3B, 3E and 3H). This junction and the junction of the longitudinal smooth muscle of the rectum with the conjoint muscle laterally and the rectococcygeal muscle posteriorly, formed the lower boundary of the mesorectal space (Fig 3).

### Perineal body

**Fig 4:**
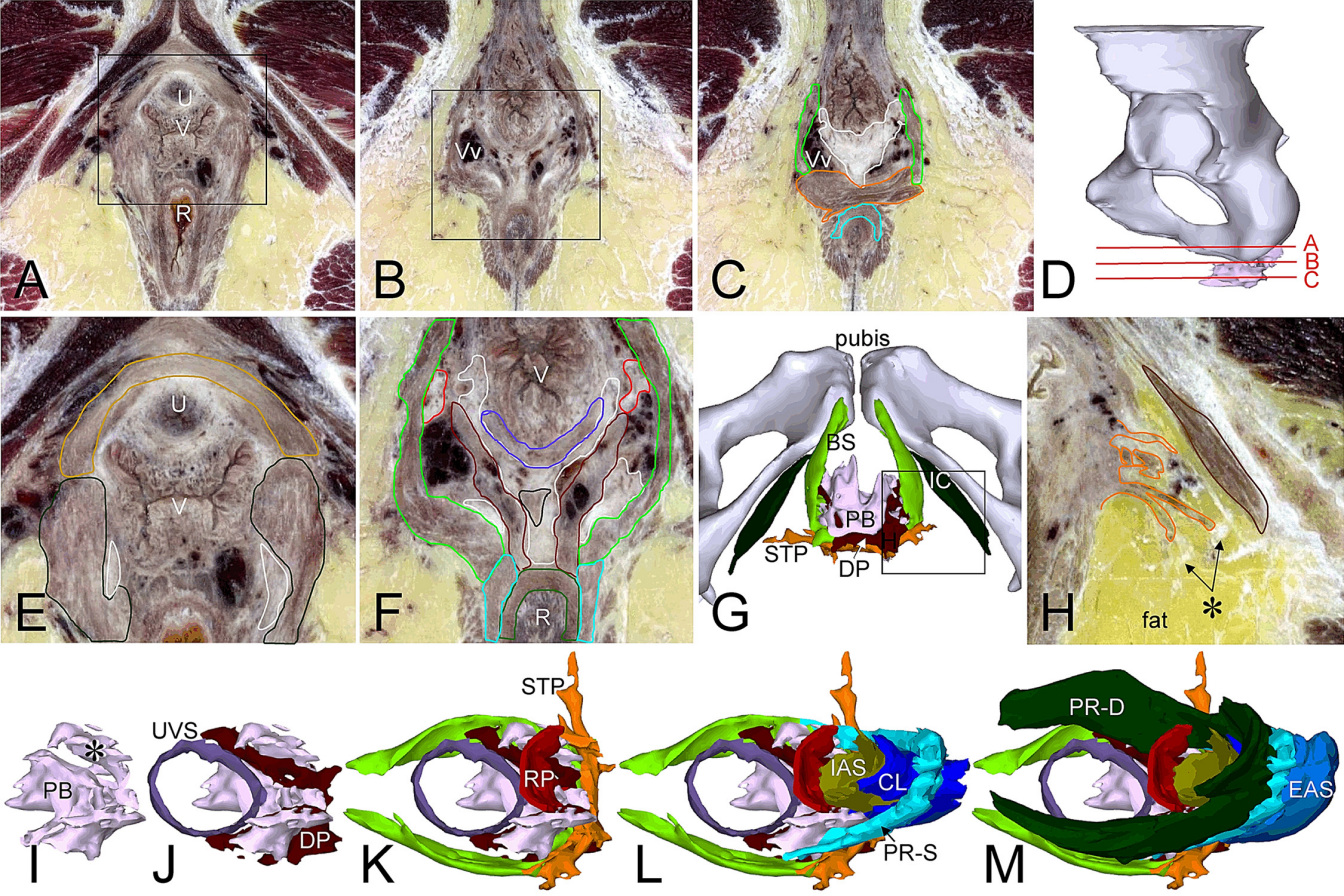
Shape and topographic relations of the perineal body and superficial perineal muscles. Panel D shows the positions of the transverse sections in panels A-C. Panels E, F and H show magnifications of the boxes in panels A and B. Panel G shows an inferior view of the superficial perineal area, while panel H shows a section of the square in panel G. The empty area between the bulbospongious and ischiocavernous muscles is mostly occupied by fat. The asterisk in panel H identifies attachments of the superficial transverse perineal muscle to connective tissue septa in the ischioanal fat. Panels I-M show slightly left oblique view of perineal body as seen from superior (I; note basal and wing (asterisk) portions), with attachments of urethrovaginal sphincter and deep perineal muscle (J), rectoperineal, superficial transverse perineal and bulbospongious muscles (K), superficial portion of puborectal muscle (L; the internal anal sphincter and conjoint longitudinal muscle of the rectum were added to visualize the anorectal bend), and deep portion of puborectal muscle (M). The color code of the (outlined) structures is shown in S2 Fig and sections without outlines in S8 Fig. Abbreviations: AL, anococcygeal ligament; BS, bulbospongious muscle; CL, conjoint longitudinal muscle of rectum; DP, deep perineal muscle; EAS, external anal sphincter; IAS, internal anal sphincter; IC, ischiocavernosus muscle; PB, perineal body; PR-D, puborectal muscle, deep portion; PR-S, puborectal muscle, superficial portion; RP, rectoperineal muscle; STP, superficial transverse perineal muscle; U, urethra; UVS, urethrovaginal sphincter; R, rectum; V, vagina; Vv, veins.

While passing the pelvic floor, the rectum made a ~45° posterior bend, the anorectal or perineal bend of the rectum. The resulting wedge-shaped space between the lower portions of the rectum and the vagina or urethra was largely occupied by the perineal body and the muscles that attach to it (Fig 2E and 2F). The perineal body was a fibrous structure with many antenna- or wing-like extensions, that served as tendinous attachments for the rectoperineal and deep perineal muscles posteriorly, the medial layer of the pubovisceral muscle and both portions of the puborectal muscle laterally, and the superficial transverse perineal and bulbospongious muscles anteriorly (Fig 4). Its volume was ~2-fold larger in CVH females than males (S1B Table). If well-developed, as in the CVH5 specimen, the perineal body had superficial and deep parts, while the deep part was less obvious if the perineal body was smaller. In females, the superficial (inferior) part was “V”-shaped, with wings extending anterolaterally along the vagina, whereas this part was a median structure in males. The deep (superior) part formed, if present, a tendinous plate on the inferior side of the puborectal muscle (Fig 2E). In both males and females, the superficial and deep, superior parts of the perineal body were incompletely separated by the smooth “deep perineal” muscle that extended from the posteroinferior side of the perineal body to the junction of the (compressor part of the) urethral sphincter with the puborectal muscle anterosuperiorly (Figs 2E, 2F and 4F, 4G, 4J–4L). The deep perineal muscle was, therefore, present as a “V”-shaped muscle on the posterolateral sides of the vagina, but, due to the absence of a vagina, as a median structure in males. Many large veins were present in and lateral to the perineal body.

## Discussion

We studied the normal anatomy of the pelvic floor and anorectum of the young adult specimens in the Chinese Visible Human dataset [10,11]. Precise knowledge of its uncompromised topography is important for the proper interpretation of physiologic measurements, the identification of changes in patients with sphincter dysfunction, and the modeling of surgical interventions and reconstructions. Pelvic-floor topography is usually studied by dissection, which is artifact-prone, or MRI, which requires prior anatomical knowledge. In contrast, the topography in specimens that were processed according to the Visible Human protocol [9] does not suffer from the artifacts of dissection, because this approach allows an iterative approach to carefully and properly delineate structures.

The guideline to identify separate structures in both dissection and the serial-section approaches is the presence of connective-tissue septa between anatomical structures. We were able to study 6 CVH bodies and compared them, where appropriate, to both VHP specimens [9]. Although this number appears small, processing a body this way is very labor-intensive. In agreement, only 7 adult female and 6 adult male “visible human” data sets exist as far as we are aware. Using this serial-section approach, we found that the levator and sphincter ani muscles have a different architecture than usually described, that the anal canal is suspended by a diaphragm, and that the perineal body can best be understood an insertional node for no fewer than 8 muscles. These findings were consistent in all specimens, although the development of connective tissue differed markedly between specimens.

### Architecture of pelvic floor

Most descriptions of the pelvic floor follow Thompson’s [17] division of the LAM into puborectal, pubococcygeal, and iliococcygeal parts and acknowledge that the levator muscle is thick anteriorly and thin, mostly aponeurotic posteriorly [18,19]. However, the question whether fibers of the pubococcygeal muscle insert into the lateral walls of the pelvic organs as pubovaginal, puboperineal, and puboanal muscles [2,20] has always been contentious. The dissectional approach usually produced a layered muscle architecture, with a deep layer that attached to the prostate/vagina and rectum, a middle layer that attached to more caudal (perineum and anus) and posterior (coccygeal bone) structures, and a superficial perineal layer that attached to the EAS [6,18,19,21]. However, Fritsch *c*.*s*. found no muscular connections between the LAM and the pelvic organs in 3–5 mm thick epoxy resin-embedded sections [22]. Our findings, which are based on 0.2–1.0 mm thick serial sections, confirmed that vagina and prostate were attached by a well-developed fibrous-tissue layer to the pelvic floor, but not with muscular connections. However, such an attachment did exist with the anorectum, because the medial layer of the pubovisceral muscle formed, together with the longitudinal smooth muscle of the rectum, the conjoint longitudinal muscle, which, in turn, inserted into the EAS proper [23].

The serial-section approach also proved informative for the architecture of the LAM itself (S1D Table). In its thick anterior portion, we, like others [19,21,22], could not separate the pubovisceral (pubococcygeal) from the puborectal portion of the LAM and concluded that they had a common insertion (“head”) on the pubic bone. Further posteriorly, we could not differentiate the pubo- and iliococcygeal muscles as separate, side-by-side entities. Instead, we observed a single, bilayered “pubovisceral” muscle, which attached to the lateral and posterior pelvic wall and the rectum. The tendinous arch, its lateral attachment to the internal obturator muscle, was, as noted earlier [19], not a well-developed structure. Posterosuperiorly, the pubovisceral muscle attached to the perimysium of the rectococcygeal muscle, which may account for its reported “staggered” insertion on the coccygeal bone [22]. Posteroinferiorly, its internal layer was continuous with the conjoint longitudinal muscle of the rectum and inferiorly, on the medial side of the puborectal muscle, with the upper border of the perineal body. The bilayered appearance of the pubovisceral muscle is reminiscent of the multilayered architecture of the LAM in some dissections [6,18,19]. A further difference between our sectional and dissectional studies is that the area, in which we and others [1] identified the smooth rectococcygeal muscle, is described as the midline raphe of the LAM [24] or the anterior layer of the anococcygeal ligament [25,26].

### Architecture of external anal sphincter

The serial-section approach was also instructive with respect to the question whether the EAS has to be divided into three parts as is commonly done [4,6,17,22,27,28], or in two parts as was more recently proposed [28,29], and whether the puborectal muscle is part of the levator or the sphincter ani muscle [6,22,27,28] (S1D Table). The circular “subcutaneous portion” of the anal sphincter (EAS “proper”) was separated by a well-developed fibrous septum from the deeper, so-called “superficial portion” of the sphincter. Furthermore and in agreement with earlier descriptions [6,22,27,28], the so-called “deep portion” of the EAS coincided with the puborectal muscle. These “superficial” and “deep” portions of the sphincter were not or incompletely separated by connective tissue anteriorly, but present as separate “slings” [4] behind the anorectal junction. Because they shared a U-shaped architecture and an inseparable anterior attachment, we renamed these muscles the superficial (anterior attachment to perineal body) and deep portions of the puborectal muscle (anterior attachment to the perineal body and pubic bone), respectively. In agreement with Fritsch *c*.*s*. [28], we therefore describe the anal sphincter complex as consisting of two parts: the U-shaped puborectal muscle and the EAS proper. The EAS proper was the only striated muscle that surrounded the anus completely. Anteriorly, the EAS covered the superficial transverse perineal muscle and was not directly attached to the perineal body as dissection had suggested [30]. Posteriorly, the anococcygeal ligament connected the EAS to the coccygeal bone. Some authors [25] recognized two layers in “their” anococcygeal ligament: a posterior layer that extended between the coccyx and the EAS proper (“our” anococcygeal ligament), and an anterior layer that extended from the presacral fascia to the conjoint longitudinal layer muscle (“our” rectococcygeal muscle). This muscle was ~2-fold thicker in females than in males.

### Rectal diaphragm

Our reconstructions showed that the anal canal was suspended at the anorectal junction by extensions of the longitudinal smooth muscle of the rectum to the coccygeal bone posterosuperiorly (rectococcygeal muscle) and to the perineal body anteroinferiorly (rectoperineal muscle). Furthermore, the anorectal junction was suspended laterally by the medial layer of the pubovisceral muscle via its continuity with the conjoint longitudinal muscle of the rectum. This rectal “diaphragm” had an inclination of ~135° with the transverse plane and was, as far as we know, not recognized thus far. It forms the inferior boundary of the mesorectum. The rectoperineal muscle was frequently described in males as the rectourethral muscle [31–33], but only twice in females [32,34]. Its volume was ~2-fold thicker in the CVH females than males, but very well developed in the VHP male [9], a body builder. Since the perineal body is the primary attachment of both rectovaginal and rectourethral muscles, the term “rectoperineal” is topographically and functionally more informative.

### Function of LAM and EAS

During “squeezing”, contraction of the puborectal muscles moves the anorectal bend anteriorly [35,36], while contraction of the EAS proper tilts, due to its attachment to the anococcygeal ligament, the anal canal superiorly [37]. A coordinated contraction of both muscles will, therefore, close the anorectum by making the anorectal bend more acute and increasing the wall pressure. The LAM, therefore, forms an integral part of the anal-sphincter complex. In contrast, defecation is preceded by a relaxation of the puborectal muscle and EAS [36], whereas changes in wall-pressure distribution [36] indicate that the rectal diaphragm contracts, which straightens the anorectal bend and, due to the anterolateral muscle-fiber orientation of the pubovisceral muscle, widens the lumen of the anal canal.

### Mesorectum and surrounding structures

The mesorectum is, as the fatty-tissue envelop of the rectum [38], a very important structure in oncological surgery of the rectum [39]. It is widest on the posterior and lateral sides of the rectum, where its adventitial cover merges with the presacral fascia and the vascular sheath of the iliac vessels [8]. Anteriorly, the rectal wall below the pelvic peritoneal reflection attaches directly to that covering the prostate or vagina (“Denonvilliers’ fascia”), but arguments whether there are one or two layers [32,34,40] remain unsettled. Inferiorly, the “rectal diaphragm” delimits the mesorectum at the level of the anorectal bend, that is, the rectococcygeal muscle posteriorly, the conjoint longitudinal muscle laterally and, if rectal adventitia and Denonvilliers’ fascia comprise two layers, the rectoperineal muscle anteriorly. We did not observe “lateral ligaments” of the rectum around a middle rectal artery [13].

### Perineal body

The perineal body is located in the space between the prostate or vagina, the anal canal and the perineal skin with, in males, the anterior extension of the EAS. It is usually described as a fibromuscular node with superficial, middle and deep layers, to which the subcutaneous EAS and the superficial and deep portions of the puborectal muscle, respectively, attach [41]. However, dissection of such a fibrous structure is difficult [24]. In sections, we found the perineal body to be a true central tendon of the perineum, unto which no fewer than 8 muscles inserted (rectoperineal muscle posterosuperiorly, urethral sphincter in males and urethrovaginal sphincter in females anterosuperiorly, deep and superficial portions of the puborectal muscles laterosuperiorly and-posteriorly, respectively; medial layer of the pubovisceral muscle mediosuperiorly, and superficial transverse perineal, bulbospongious, and deep perineal muscles posteriorly). The size and extension of the perineal body was much smaller in males than in females and varied in females with the degree of development of the connective tissue. If well developed, as in many females, its anterior extension is sometimes called the perineal membrane [24]. There is some controversy as to whether the bulbospongious and superficial transverse perineal muscles, and the EAS proper insert into the perineal body or pass the structure posteriorly [42,43]. Our observations showed that the fibres of the bulbospongious and superficial transverse perineal muscles were indistinguishable in the midline, where they formed a muscle bundle that lay deep to the EAS proper and attached to the posterior part of the perineal body.

In anatomical atlases, the superficial transverse perineal muscle attaches laterally to the tuberosity of iliac bone. We found, instead, that this muscle did not reach the bone in any of the specimens, but attached to the fibrous septa surrounding the small fat compartments in the ischioanal fossa. Contraction will, therefore, make the fat compartments in the ischioanal fossa more firm and may, hence, support the inferior side of the pelvic floor as an “adipose cushion pillow” [44].

## Supporting Information

S1 Fig Image of interactive 3D-PDF. The interactive 3D-PDF itself and instructions on how to use it can be found in S7 Fig.
(TIF)

S2 Fig The Amira software interface. The segmentation procedure and the color code of the structures identified are shown. The same color code is used to label these structures in Figs 1–4 and S7 Fig.
(TIF)

S3 Fig Images of sections shown in Fig 1 without contours. All Figures were magnified 1.3-fold. The panel labels are retained.
(PDF)

S4 Fig Images of sections shown in Fig 2 without contours. All Figures were magnified 1.3-fold. The panel labels are retained.
(PDF)

S5 Fig Images of sections shown in Fig 3 without contours. All Figures were magnified 1.3-fold. The panel labels are retained.
(TIF)

S6 Fig Images of sections shown in Fig 4 without contours. All Figures were magnified 1.3-fold. The panel labels are retained.
(TIF)

S7 Fig Interactive 3D rendering of the topographic anatomy of the female pelvic floor. The reconstruction (see “interactive 3D-pdf topography of ASC.pdf”), which is based on 46 structures identified in the CVH5 specimen, can be moved with the left mouse button and changed in size with the right mouse button. Structures can be activated or removed, or made transparent via the “Model Tree” option (shown on left side of image and activated via a right click in the image). Note that the levator ani muscle has a single anterior head, so that the division between the anterior portion of the pubovisceral and puborectal muscles is arbitrary and introduced only because of the requirements of the reconstruction program.
(TIF)

S8 Fig Serial sections 1–93 of the lesser pelvis of specimen CVH5. These sections were used to build the reconstruction shown in S7 Fig.
(TIF)

S1 Table A: Biometric data of the CVH bodies and details of sections; B: Biometric data of the pelvic floor in CVH specimens; C: List of structures identified and reconstructed in S2 Fig; and D: Comparison of terminology of levator and sphincter ani muscles in present study with that in Terminologia Anatomica. (DOCX)
