# Correction: 3D Topography of the Young Adult Anal Sphincter Complex Reconstructed from Undeformed Serial Anatomical Sections

**DOI:** 10.1371/journal.pone.0140736

**Published:** 2015-10-09

**Authors:** Yi Wu, Noshir F. Dabhoiwala, Jaco Hagoort, Jin-Lu Shan, Li-Wen Tan, Bin-Ji Fang, Shao-Xiang Zhang, Wouter H. Lamers

There file names for S3 Fig, S4 Fig, S5 Fig, S6 Fig, S7 Fig and S8 Fig are incorrectly switched. There is also an error in the caption for S2 Fig. Please see the corrected file names and captions for S2–S8 Figs below.

S2 Fig The Amira software interface. The segmentation procedure and the color code of the structures identified are shown. The same color code is used to label these structures in Figs 1–4 and S3 Fig.
(TIF)

S3 Fig Interactive 3D rendering of the topographic anatomy of the female pelvic floor. The reconstruction (see “interactive 3D-pdf topography of ASC.pdf”), which is based on 46 structures identified in the CVH5 specimen, can be moved with the left mouse button and changed in size with the right mouse button. Structures can be activated or removed, or made transparent via the “Model Tree” option (shown on left side of image and activated via a right click in the image). Note that the levator ani muscle has a single anterior head, so that the division between the anterior portion of the pubovisceral and puborectal muscles is arbitrary and introduced only because of the requirements of the reconstruction program.
(TIF)

S4 Fig Serial sections 1–93 of the lesser pelvis of specimen CVH5. These sections were used to build the reconstruction shown in S3 Fig.
(TIF)

S5 Fig Images of sections shown in Fig 1 without contours. All Figures were magnified 1.3-fold. The panel labels are retained.
(PDF)

S6 Fig Images of sections shown in Fig 2 without contours. All Figures were magnified 1.3-fold. The panel labels are retained.
(PDF)

S7 Fig Images of sections shown in Fig 3 without contours. All Figures were magnified 1.3-fold. The panel labels are retained.
(TIF)

S8 Fig Images of sections shown in Fig 4 without contours. All Figures were magnified 1.3-fold. The Spanel labels are retained.
(TIF)
