# Supplementary figures and images for: 3D Topography of the Young Adult Anal Sphincter Complex Reconstructed from Undeformed Serial Anatomical Sections
Source: PLoS One. 2015 Aug 25;10(8):e0132226. doi: 10.1371/journal.pone.0132226 (PMC4549266; doi:10.1371/journal.pone.0132226)

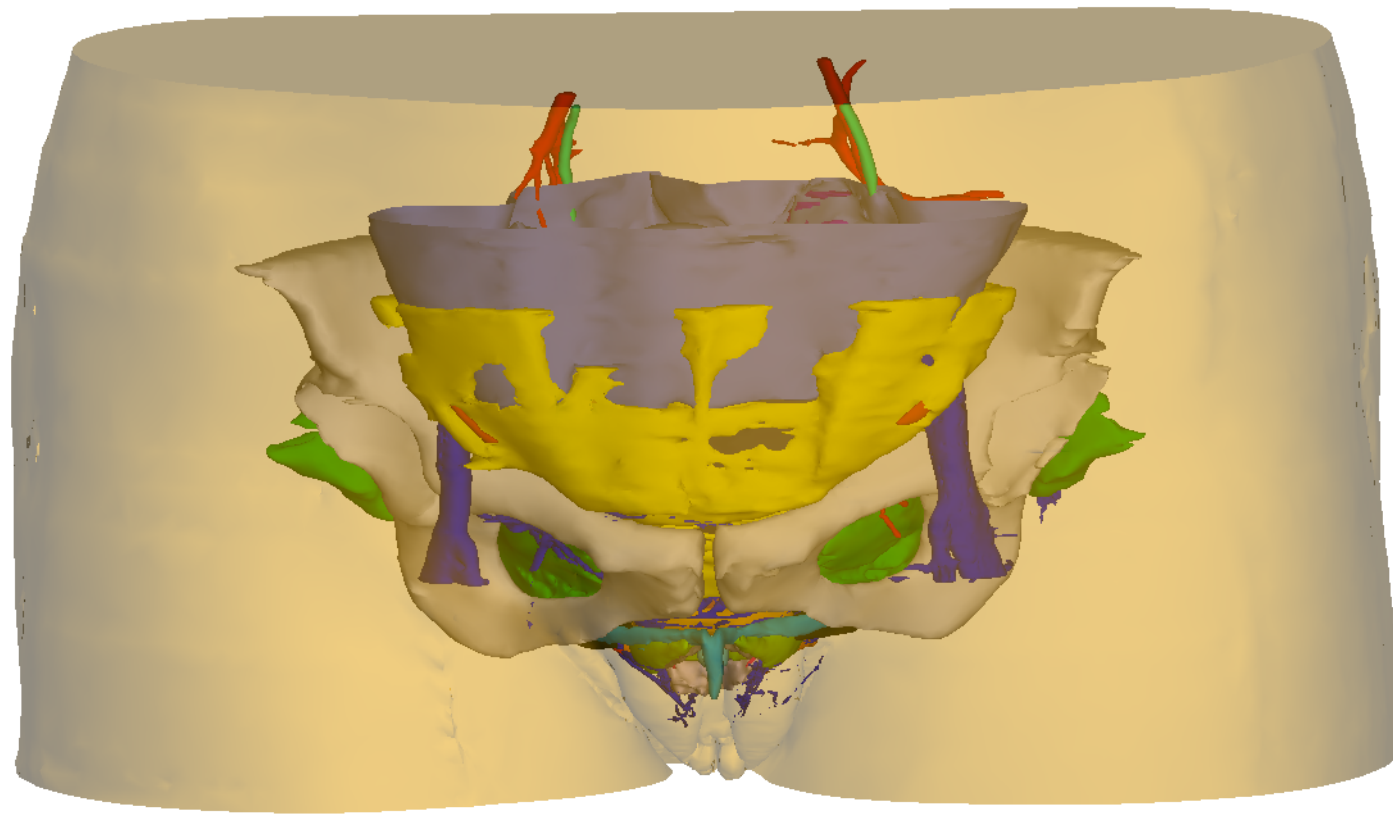

Supplement: S3 Fig — All Figures were magnified 1.3-fold. The panel labels are retained. (PDF) [file pone.0132226.s003.pdf]

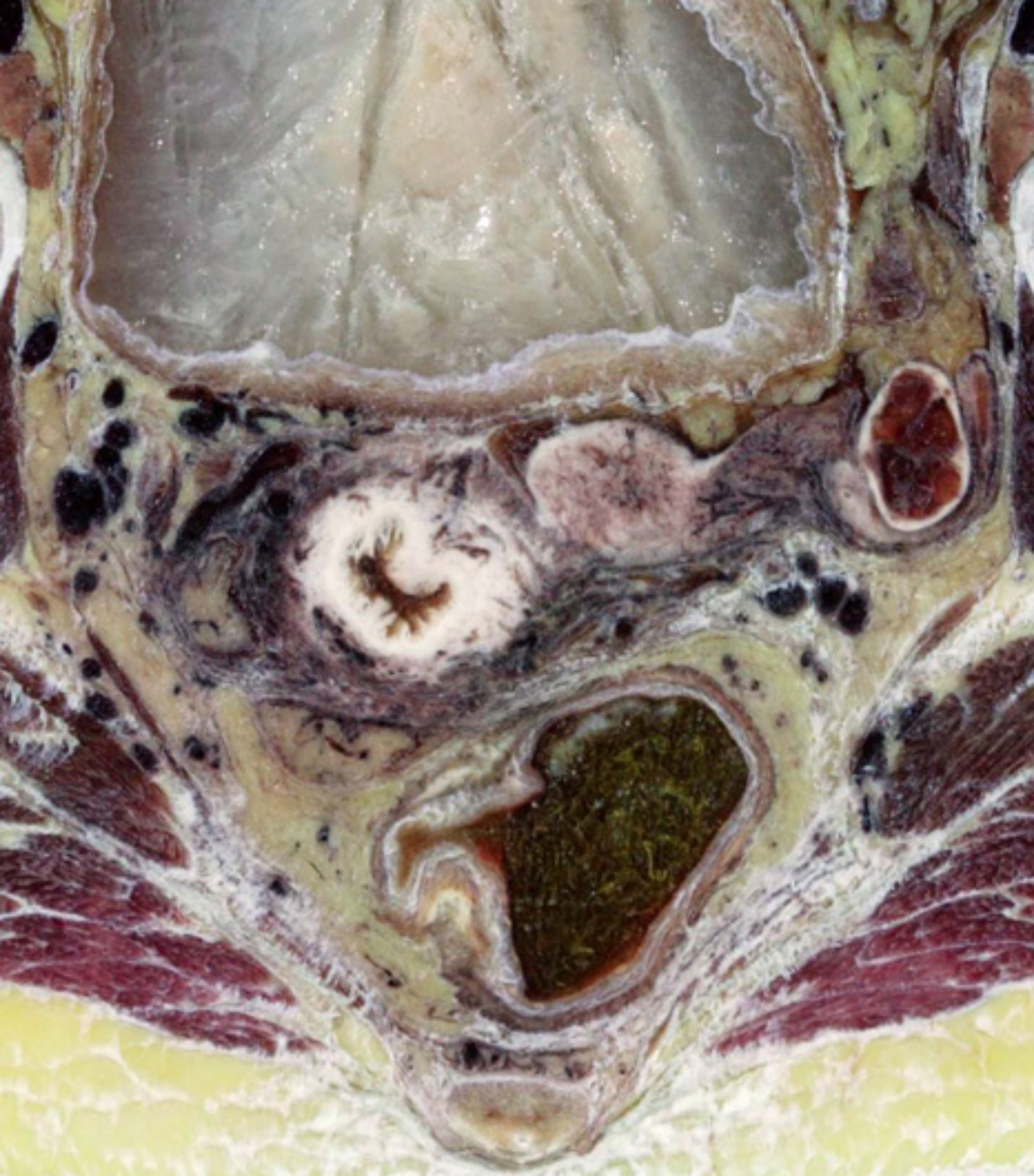

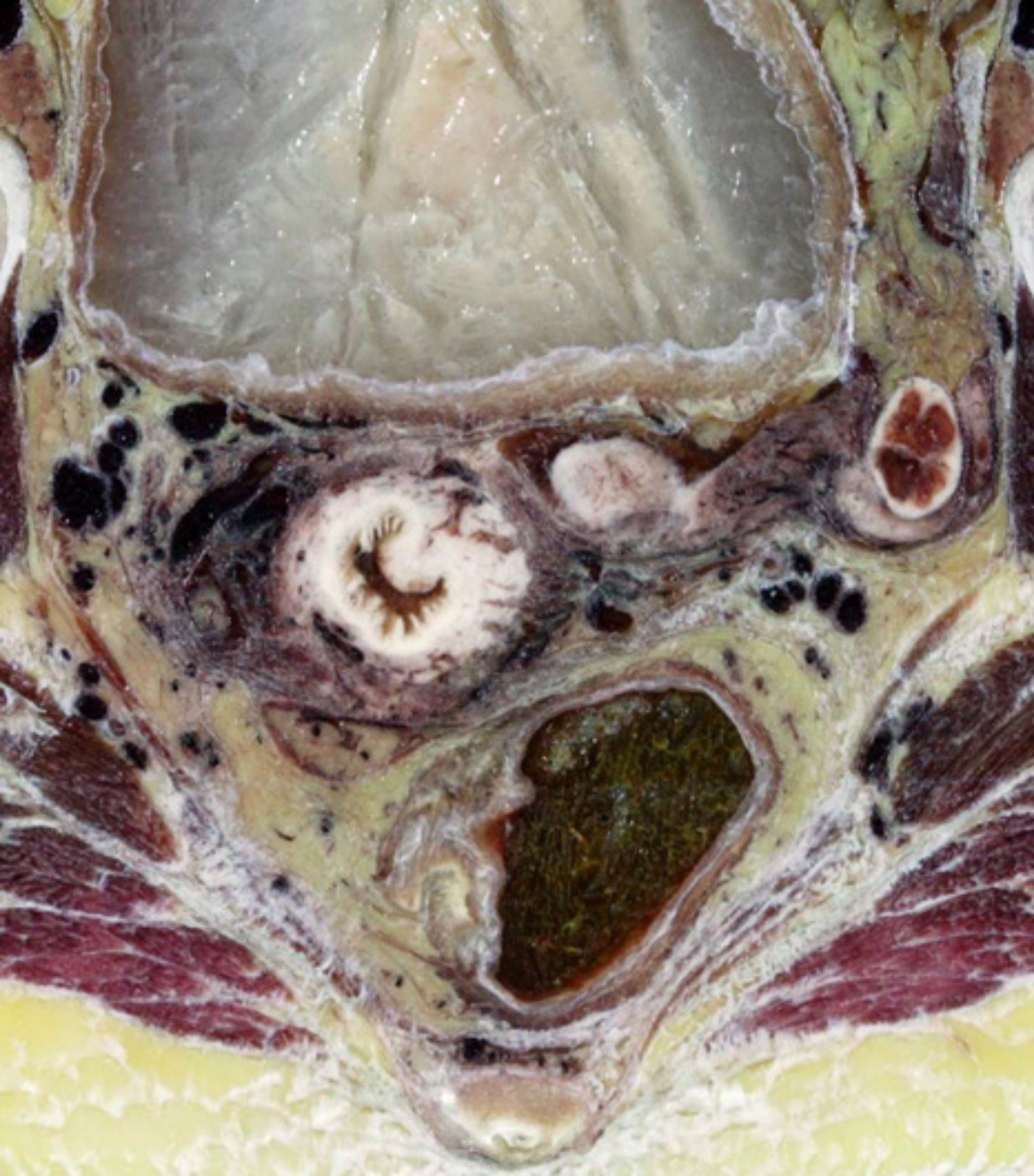

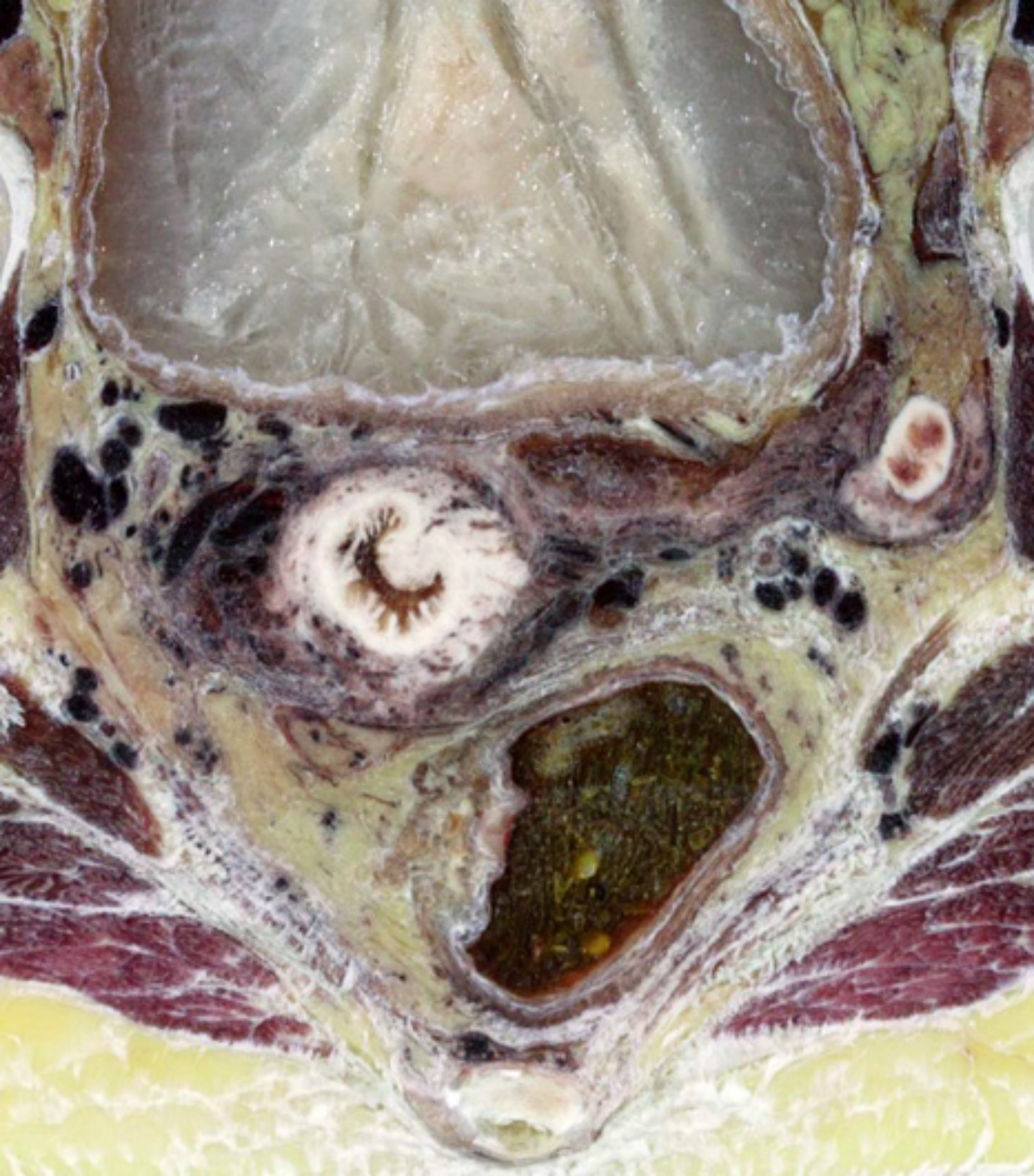

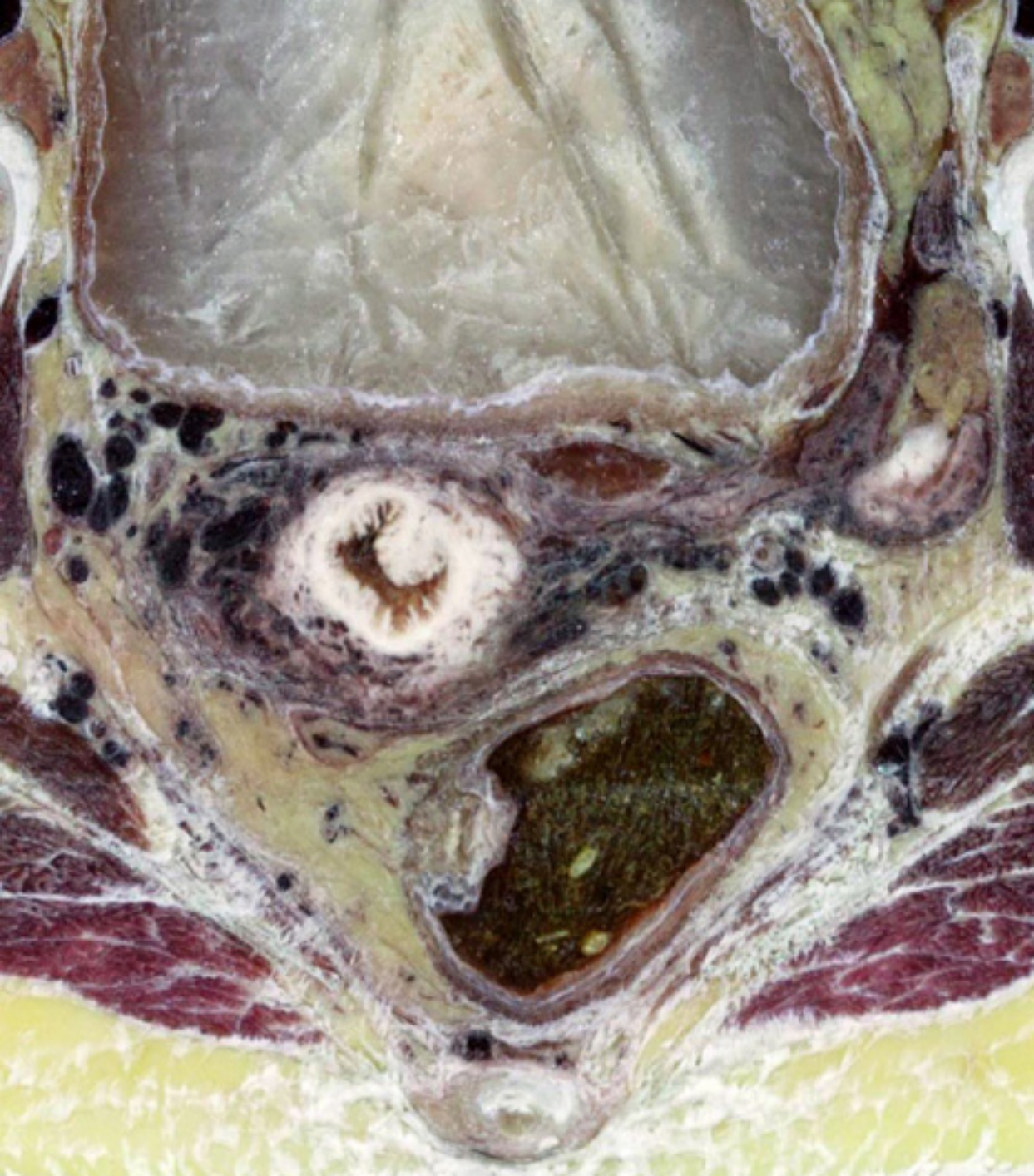

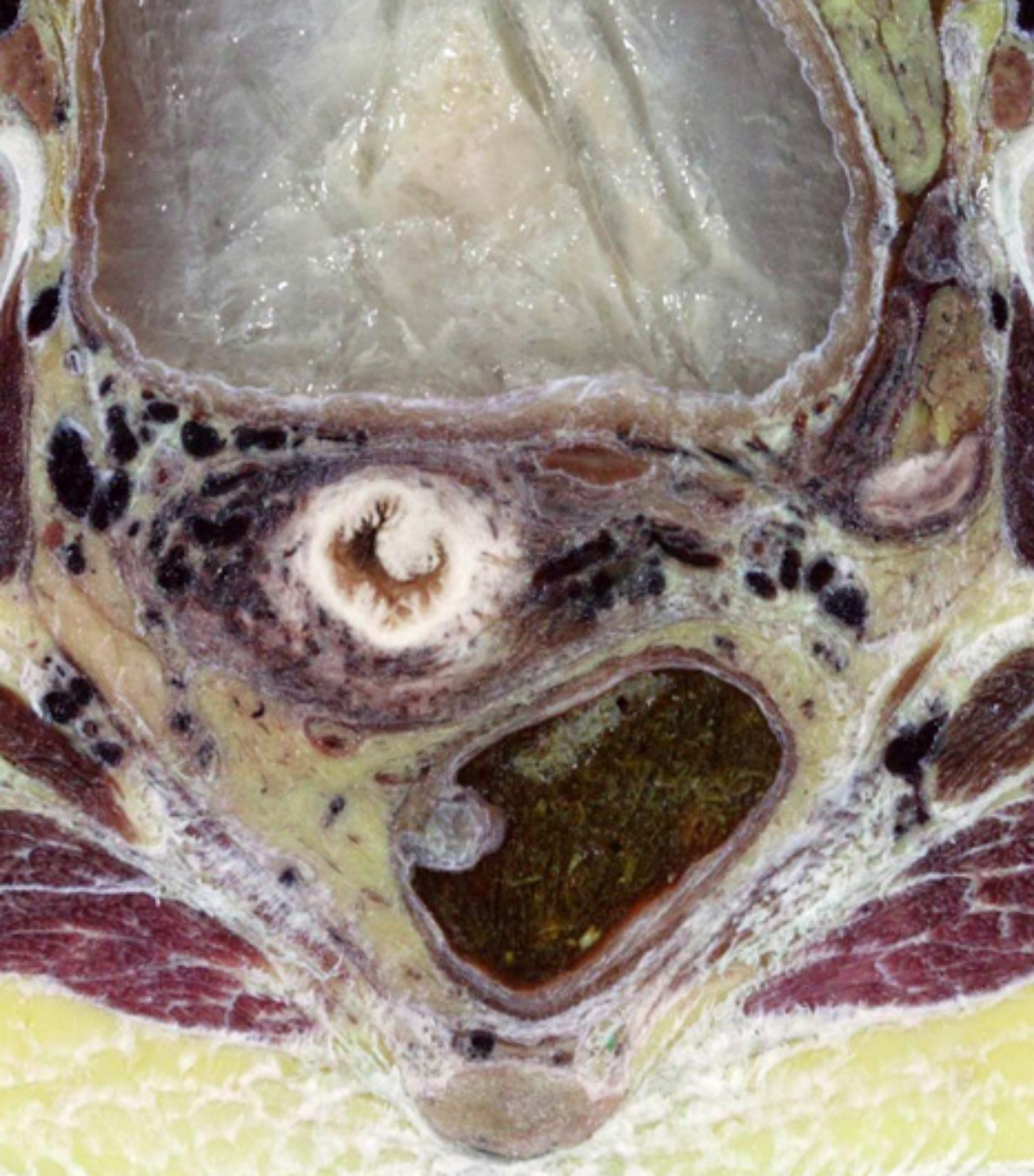

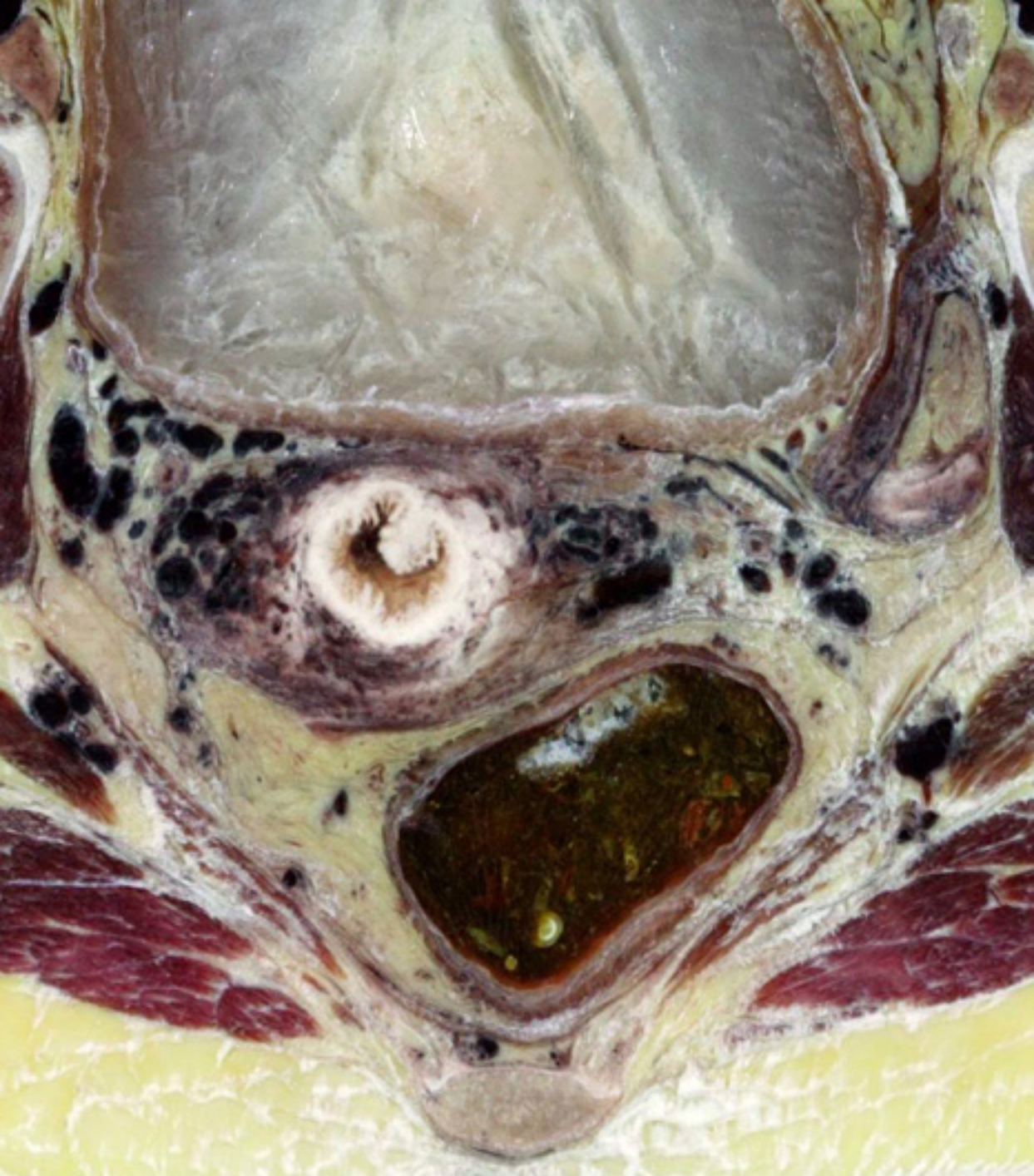

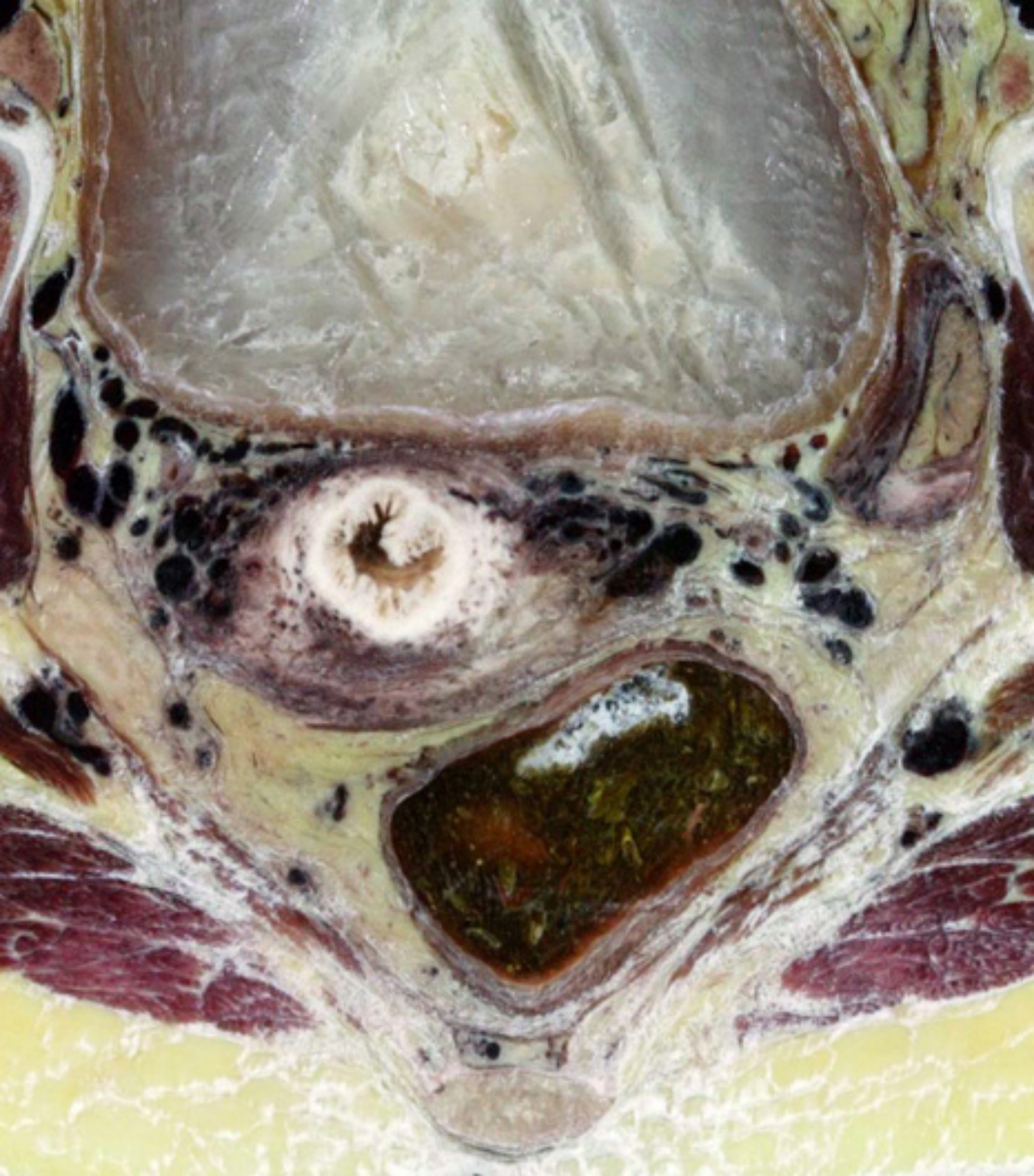

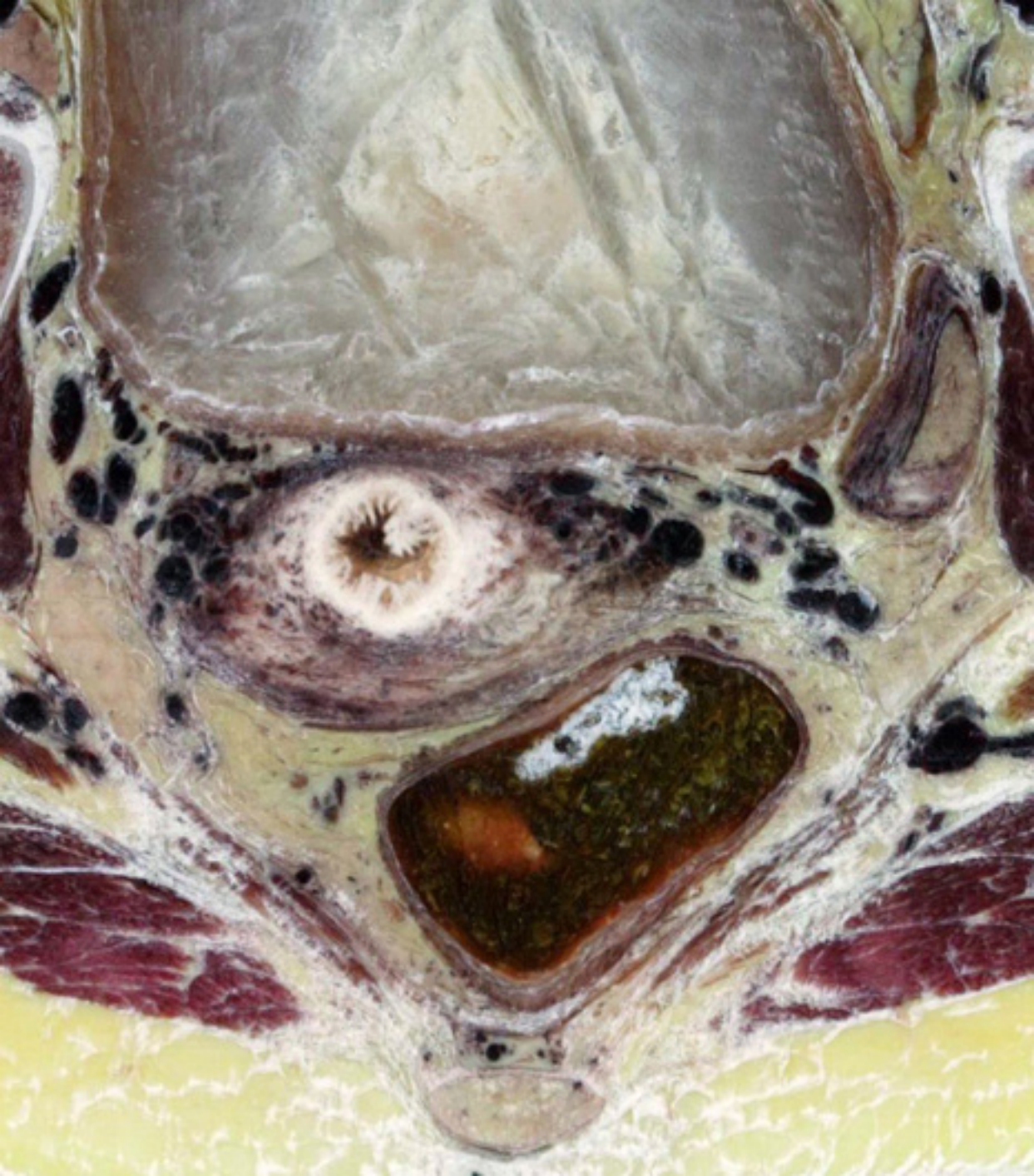

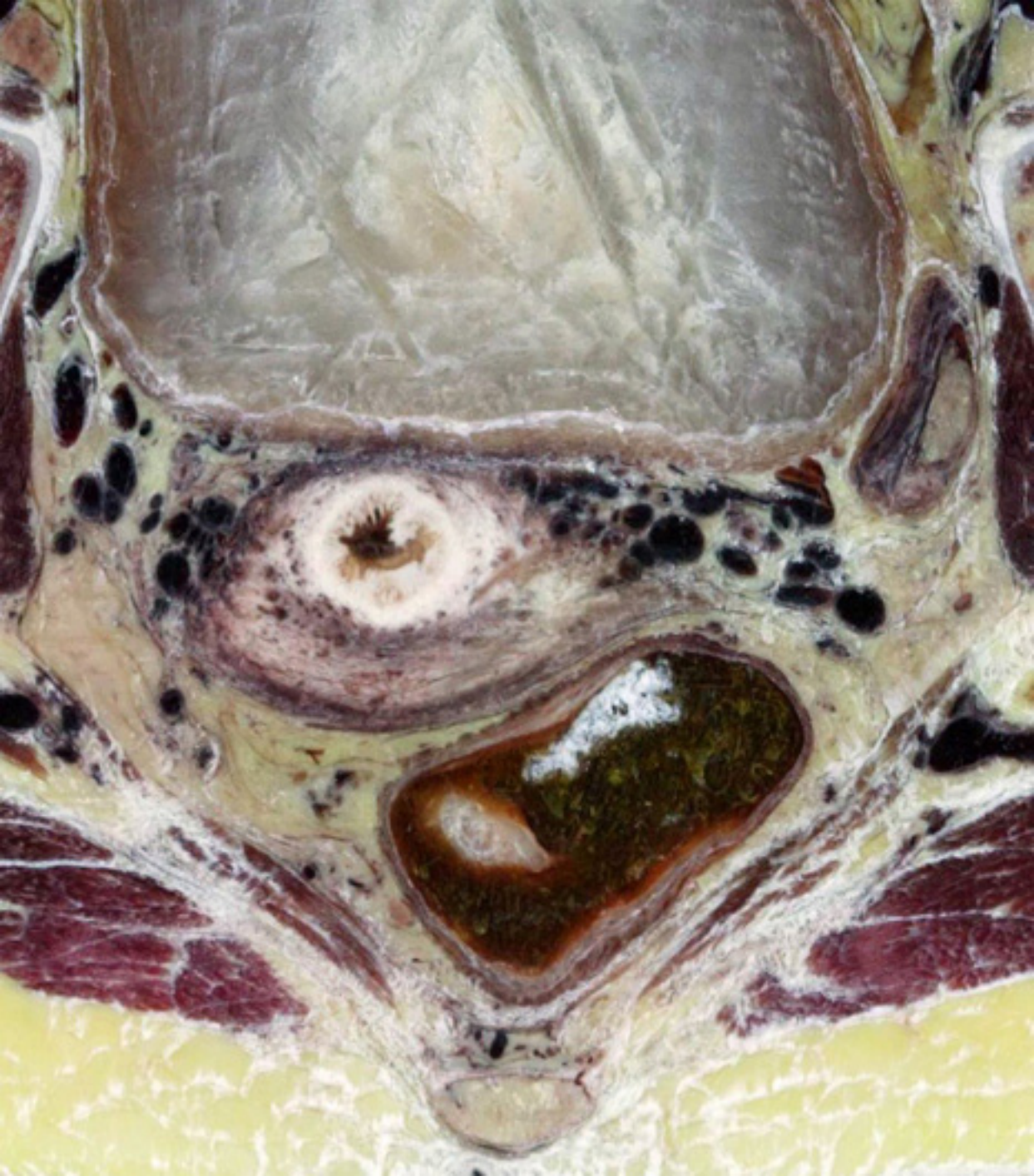

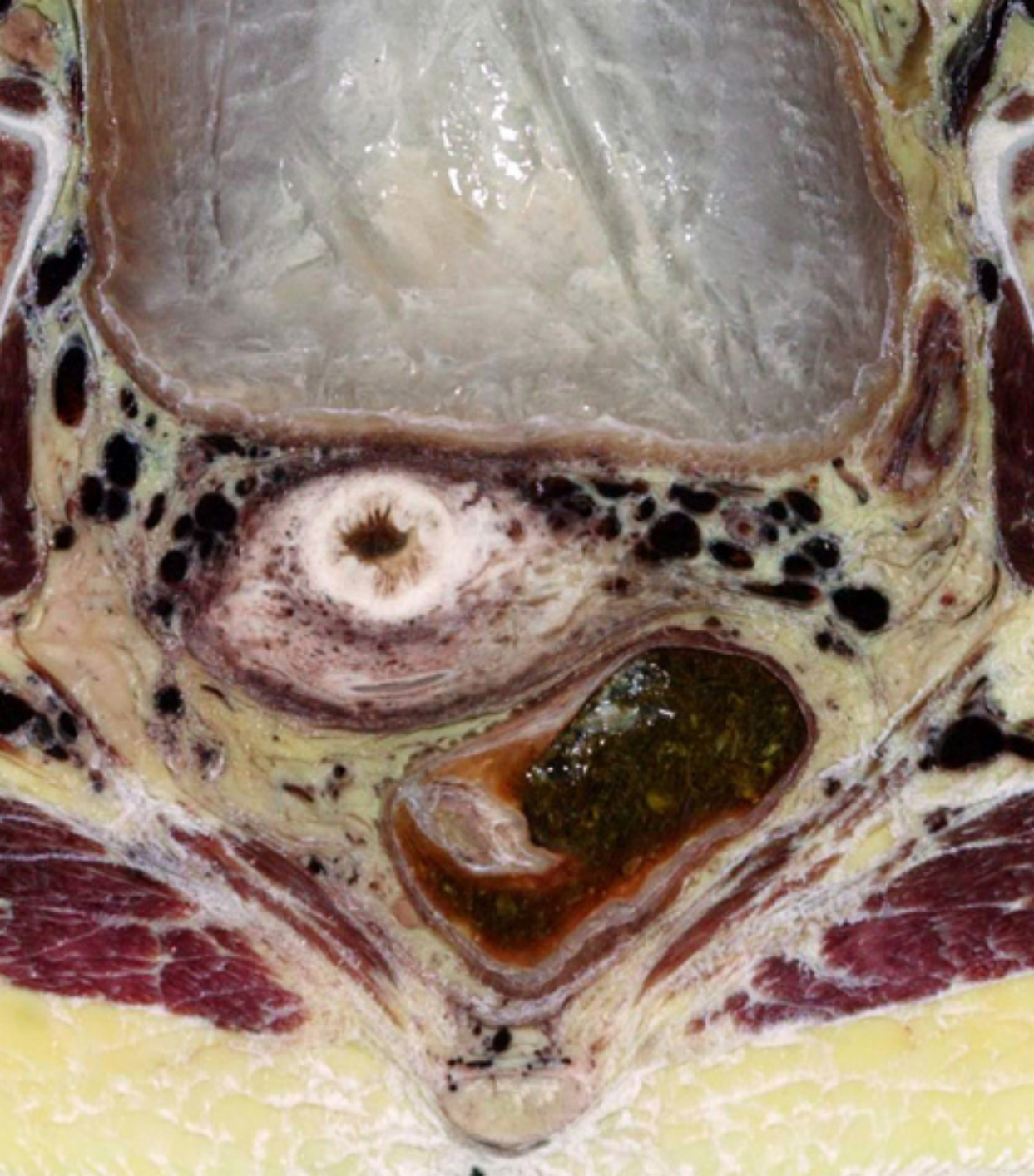

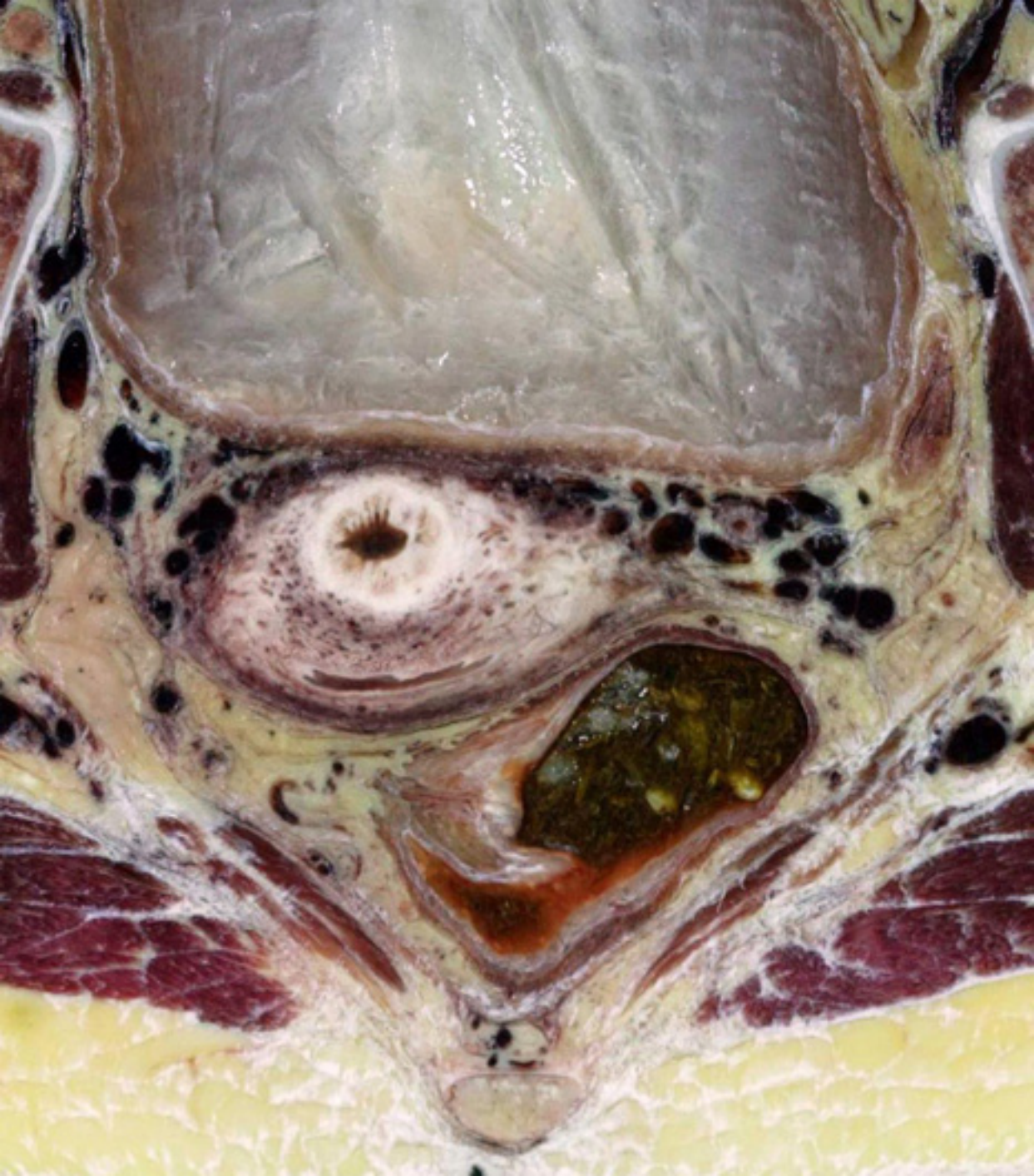

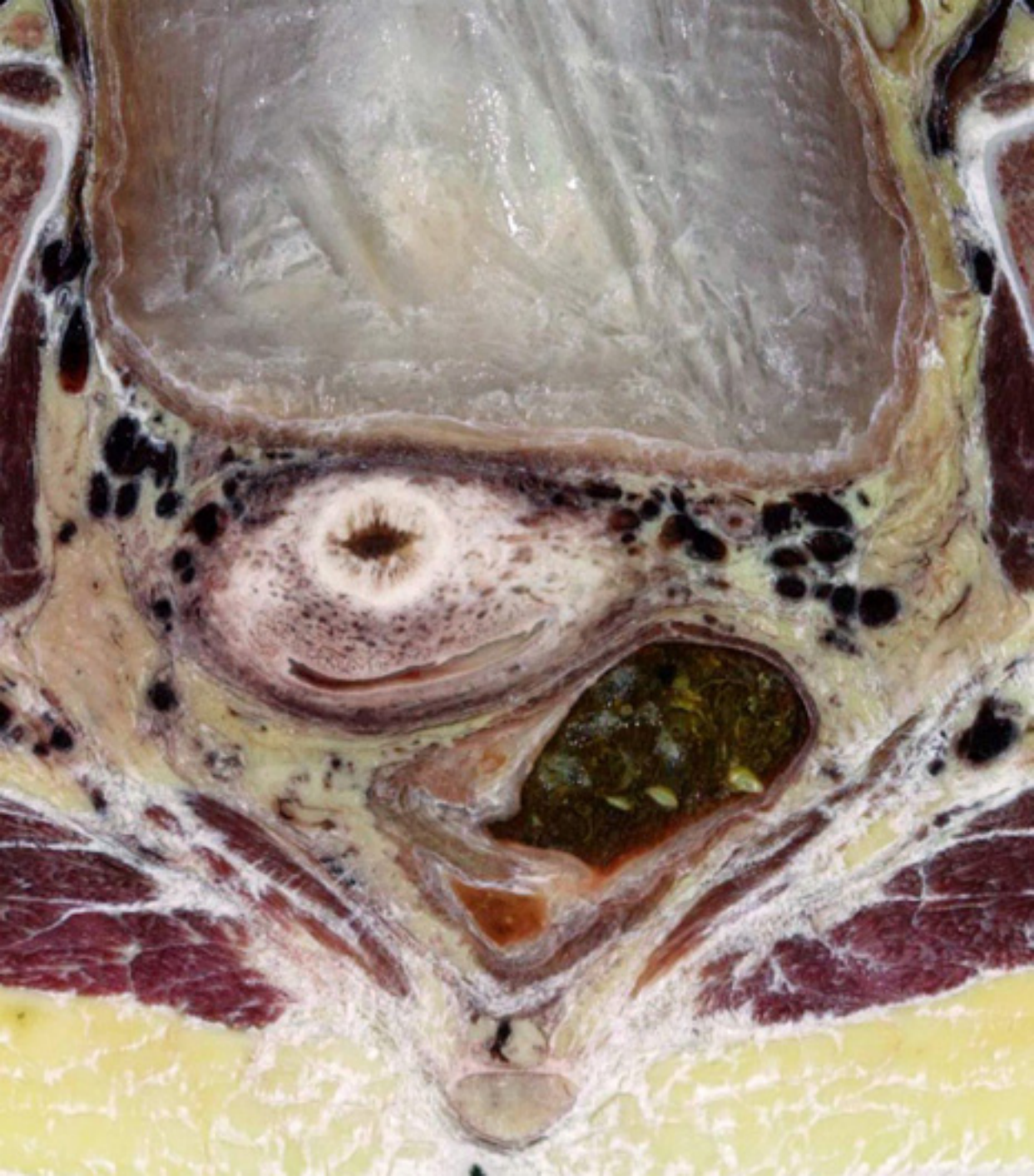

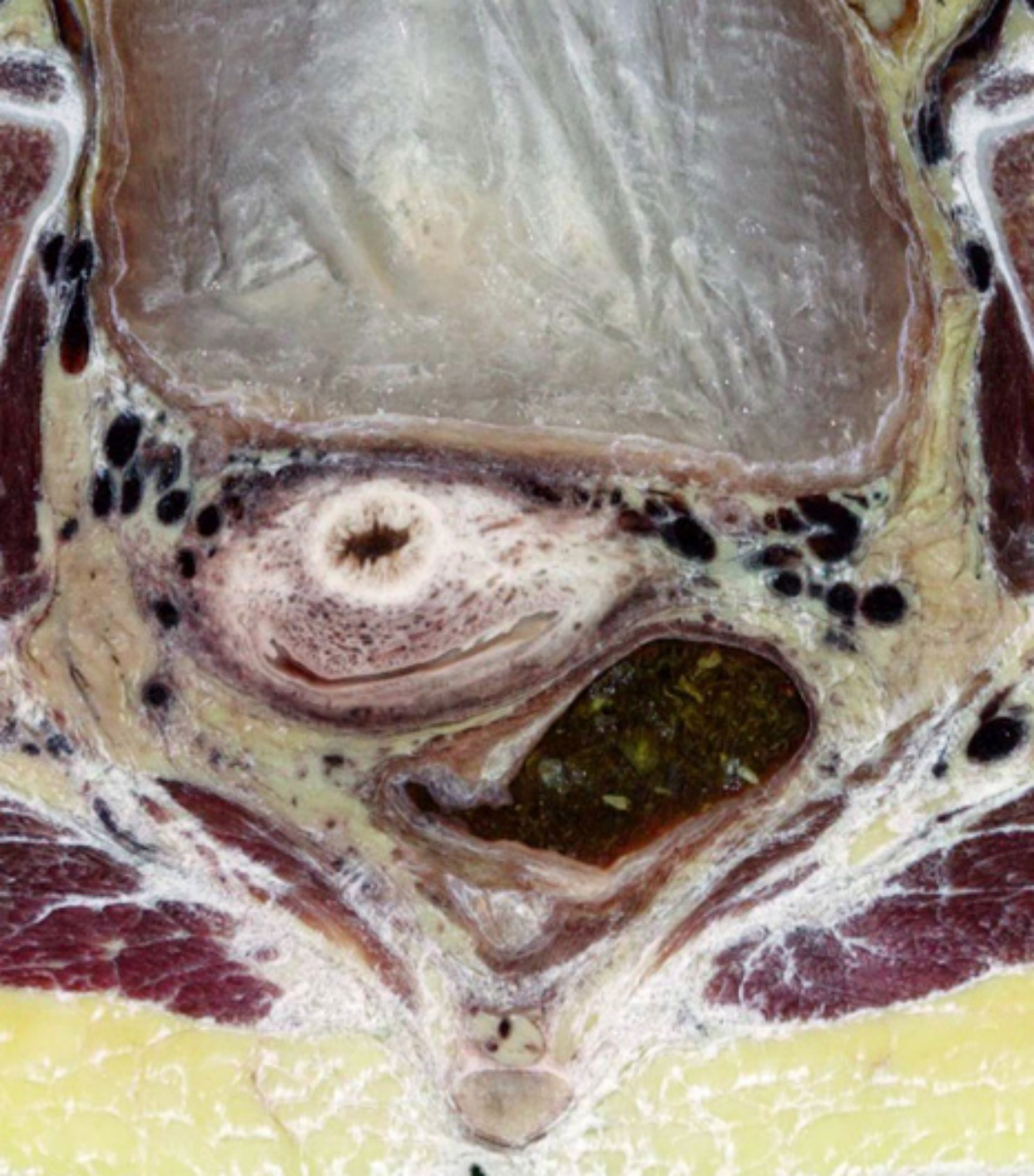

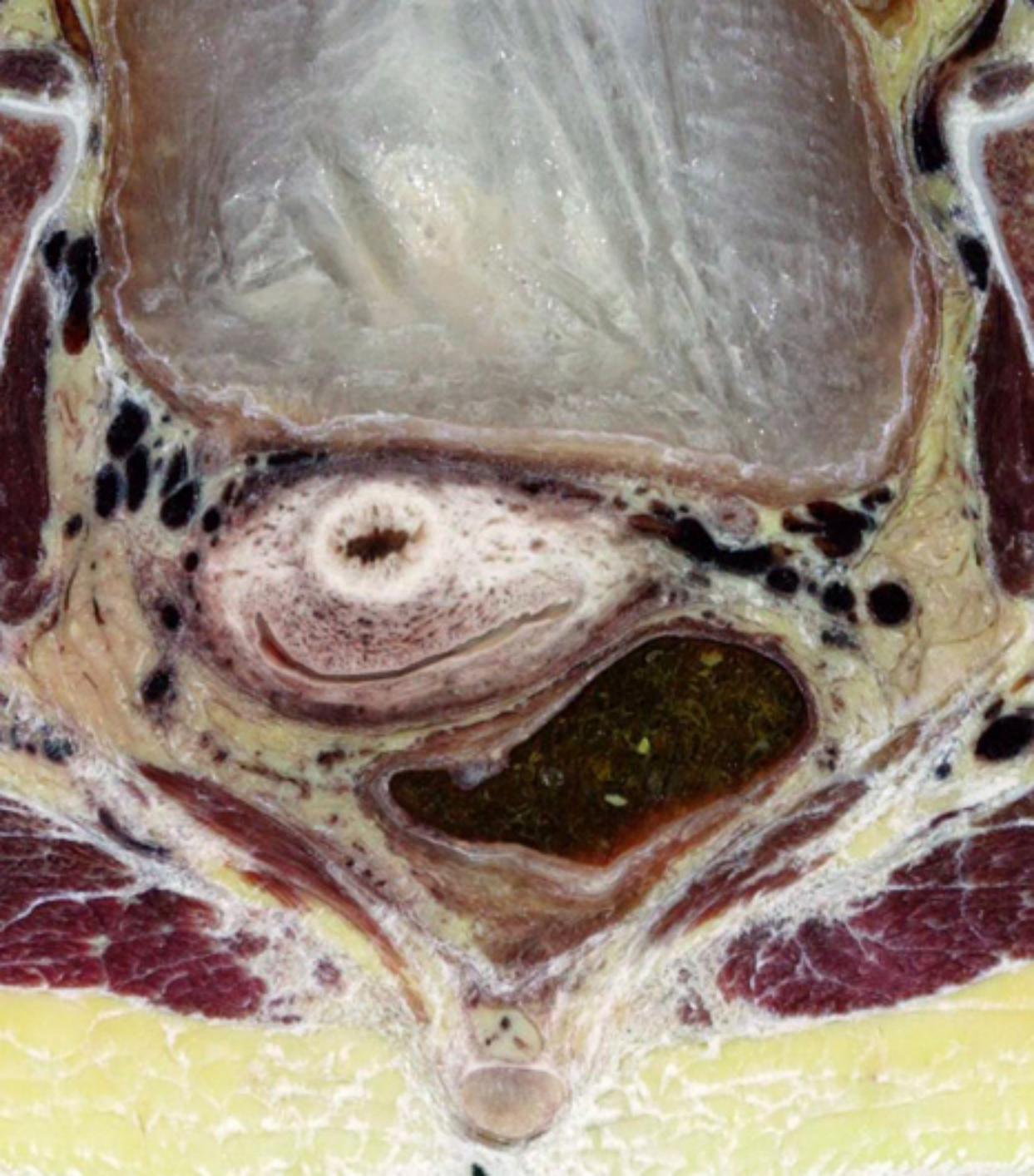

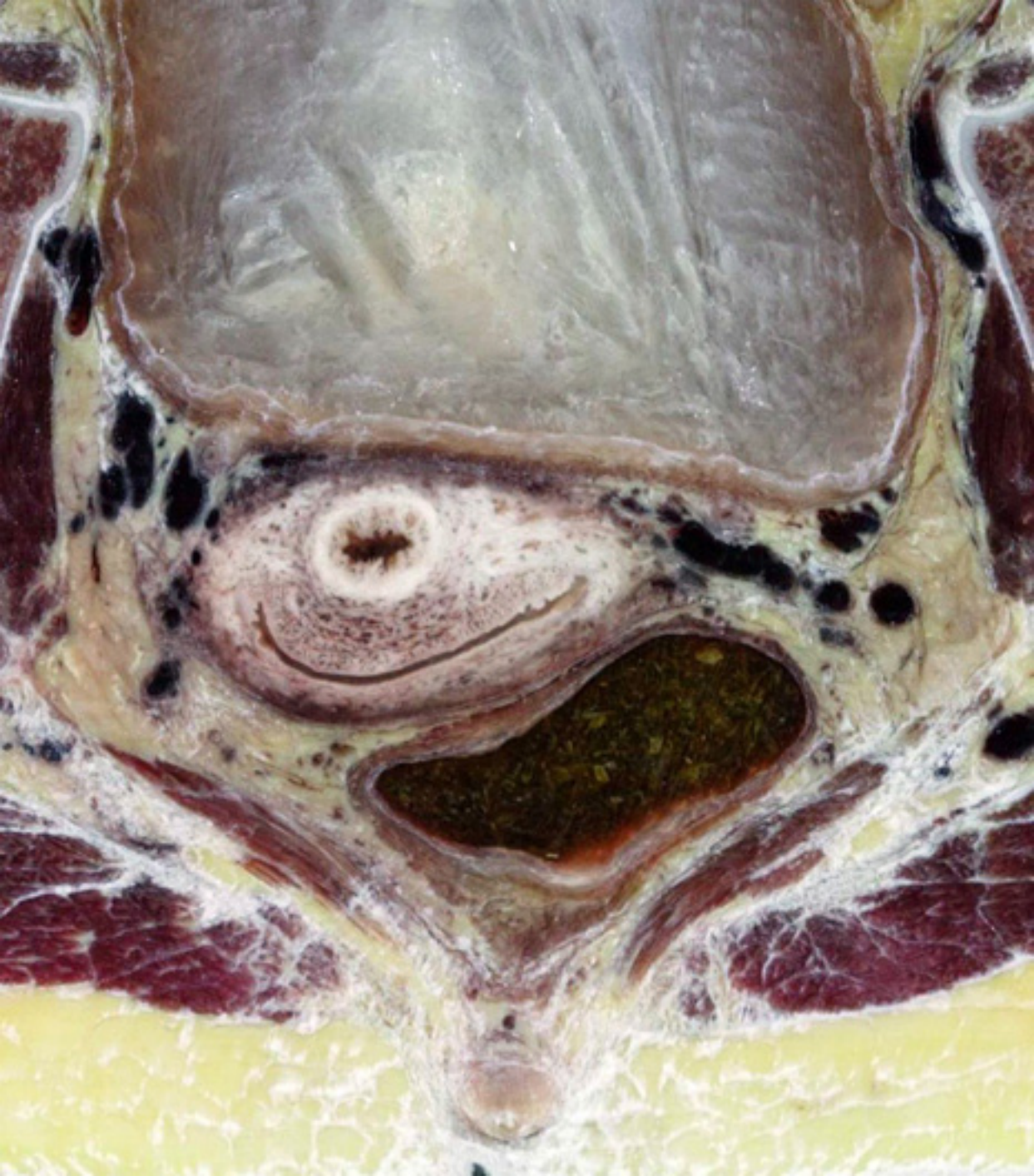

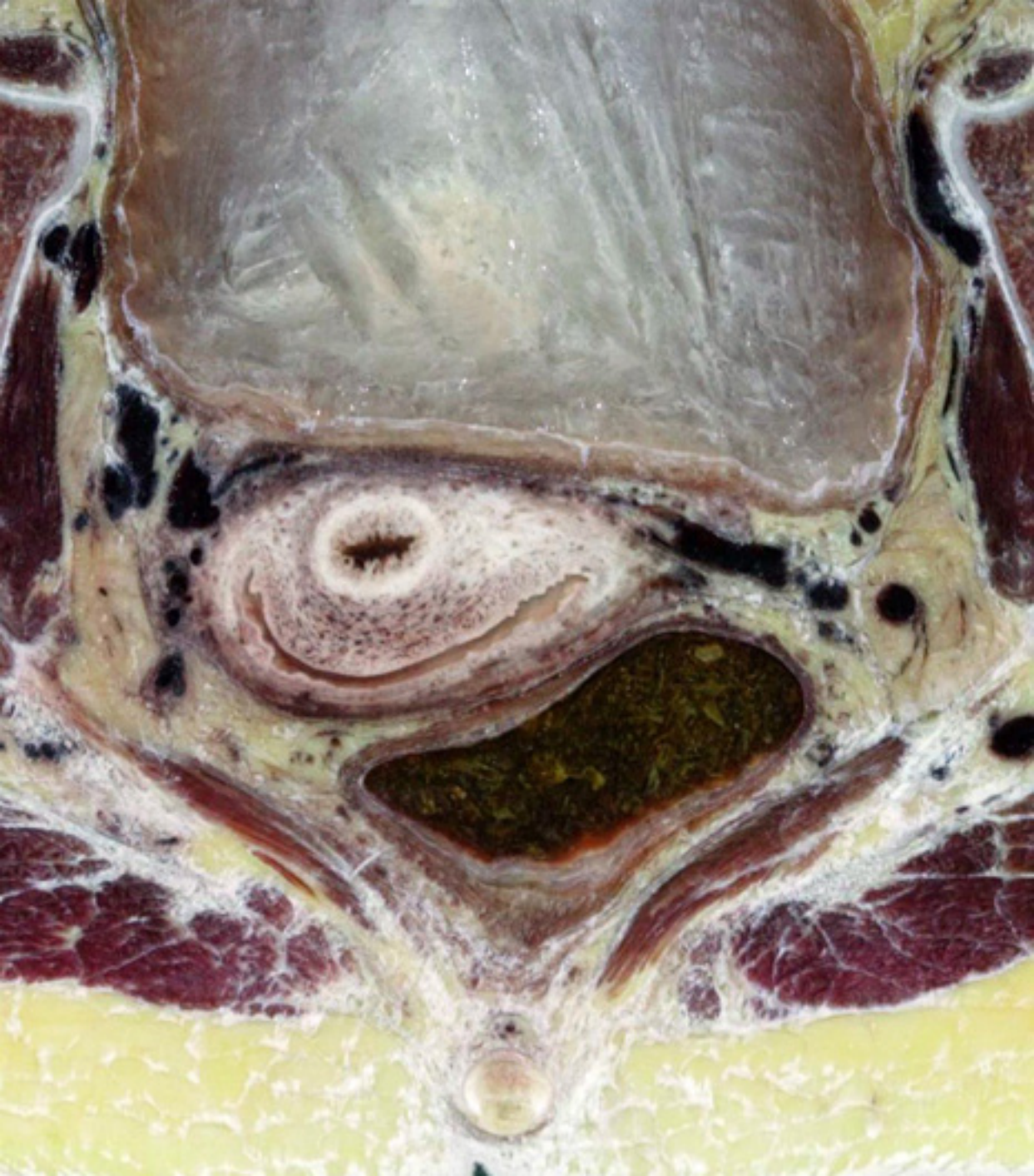

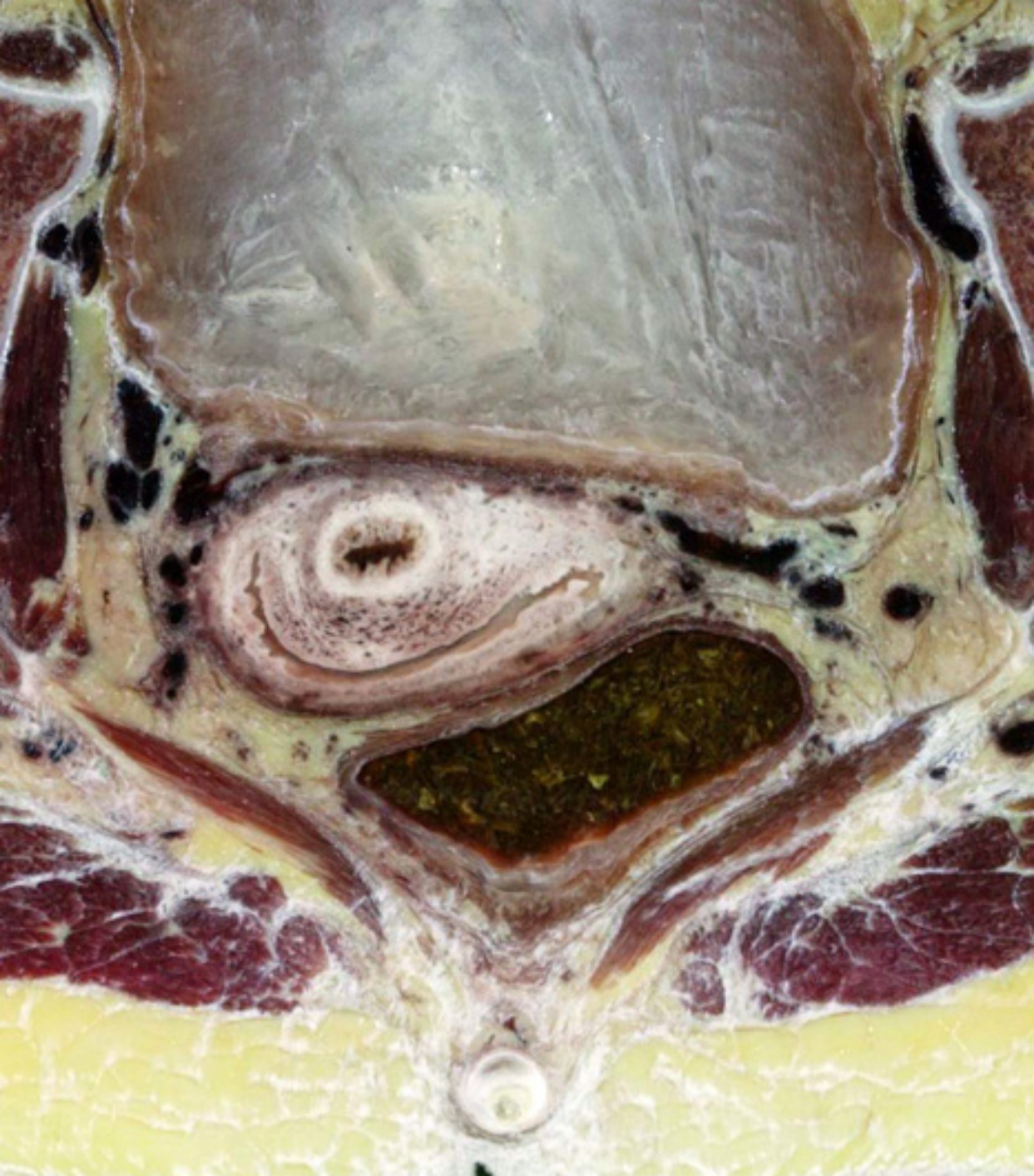

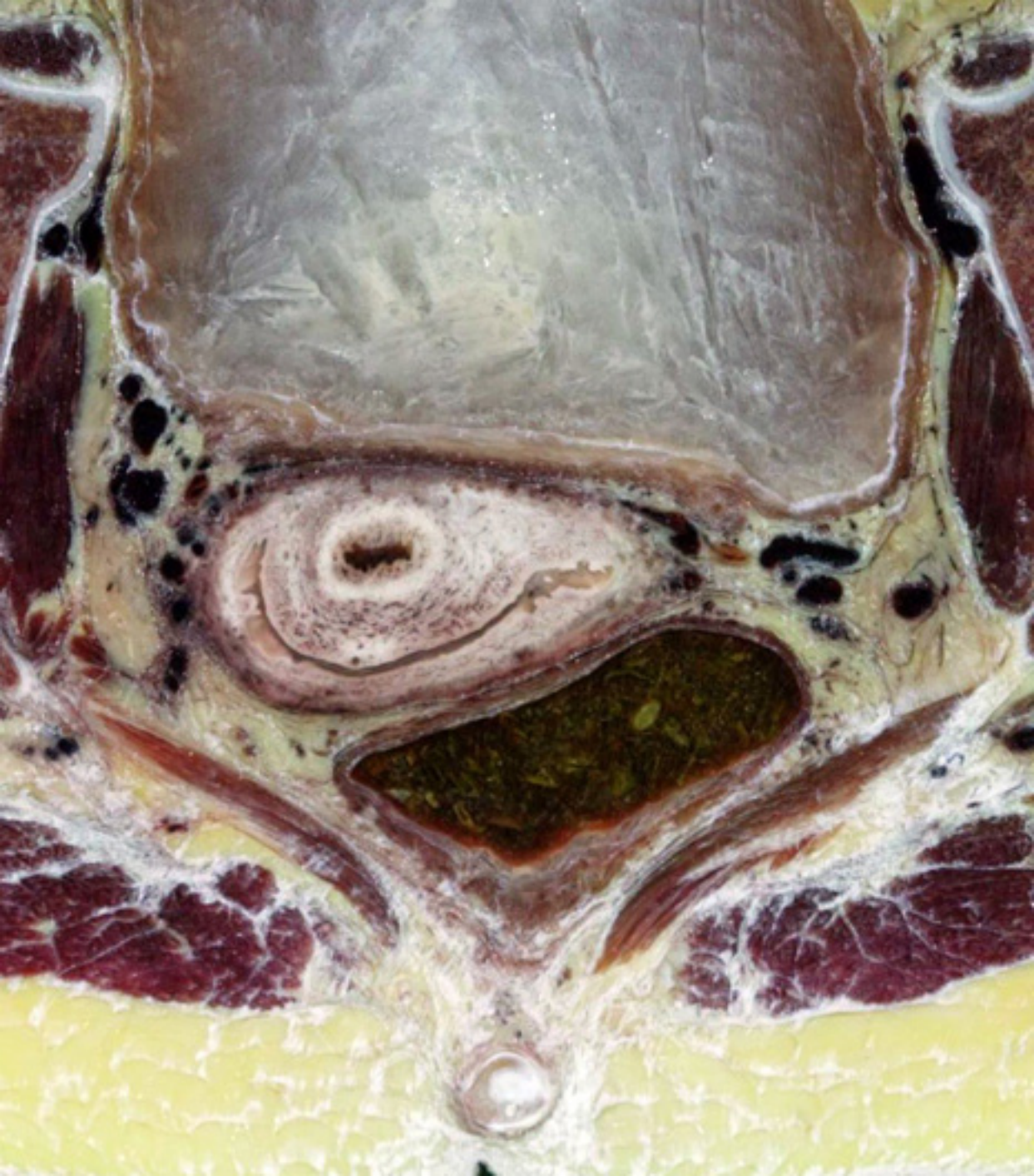

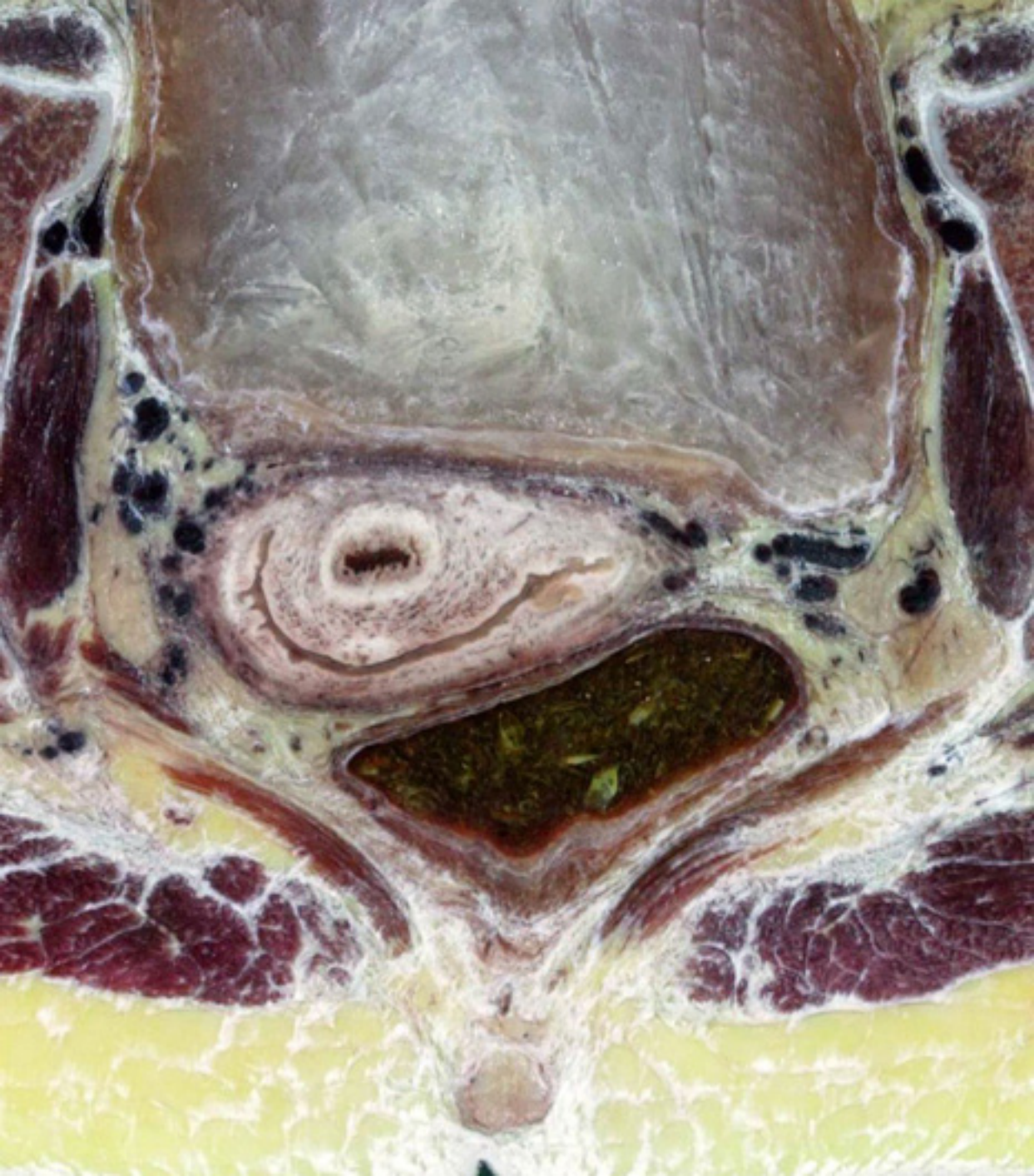

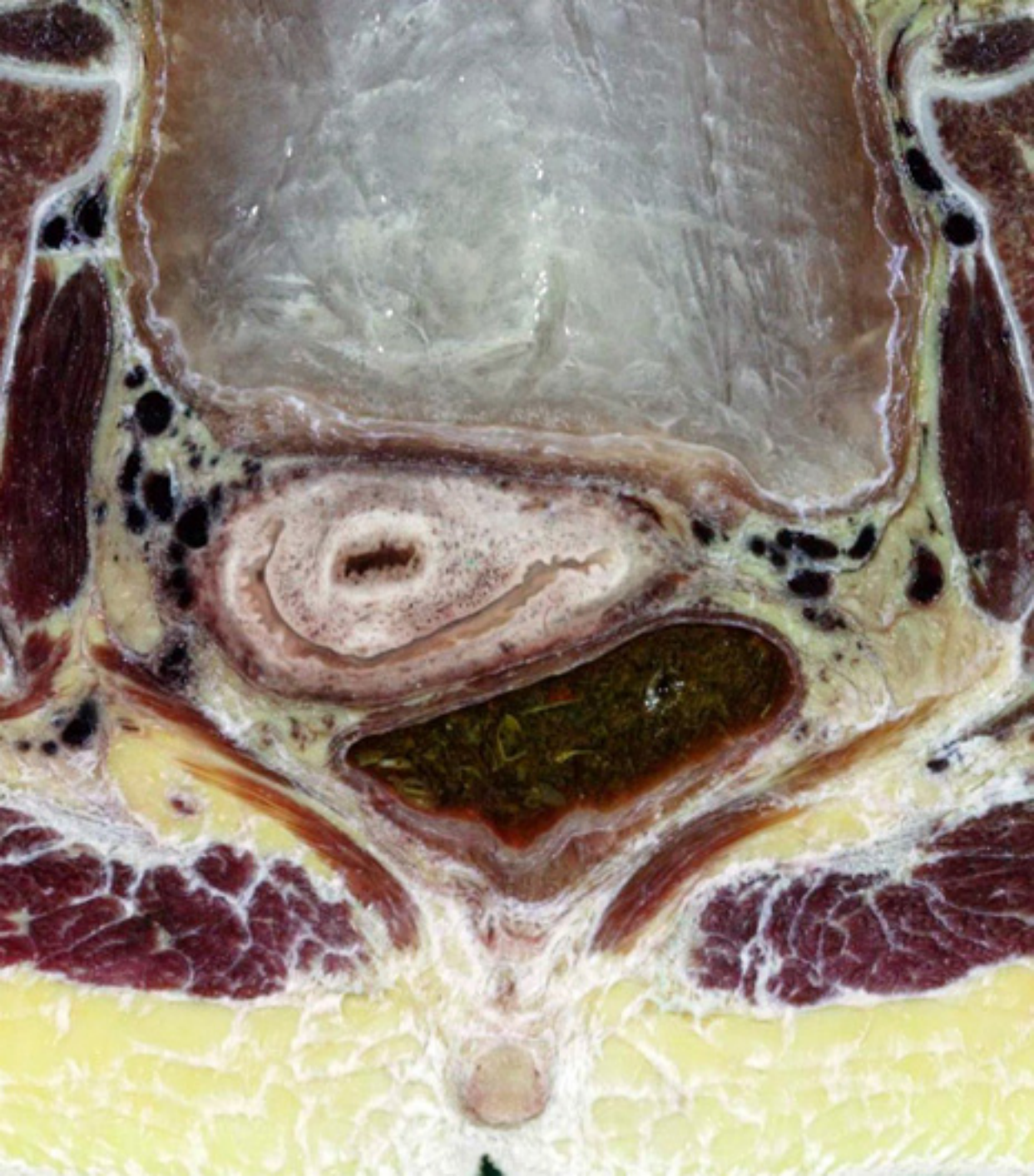

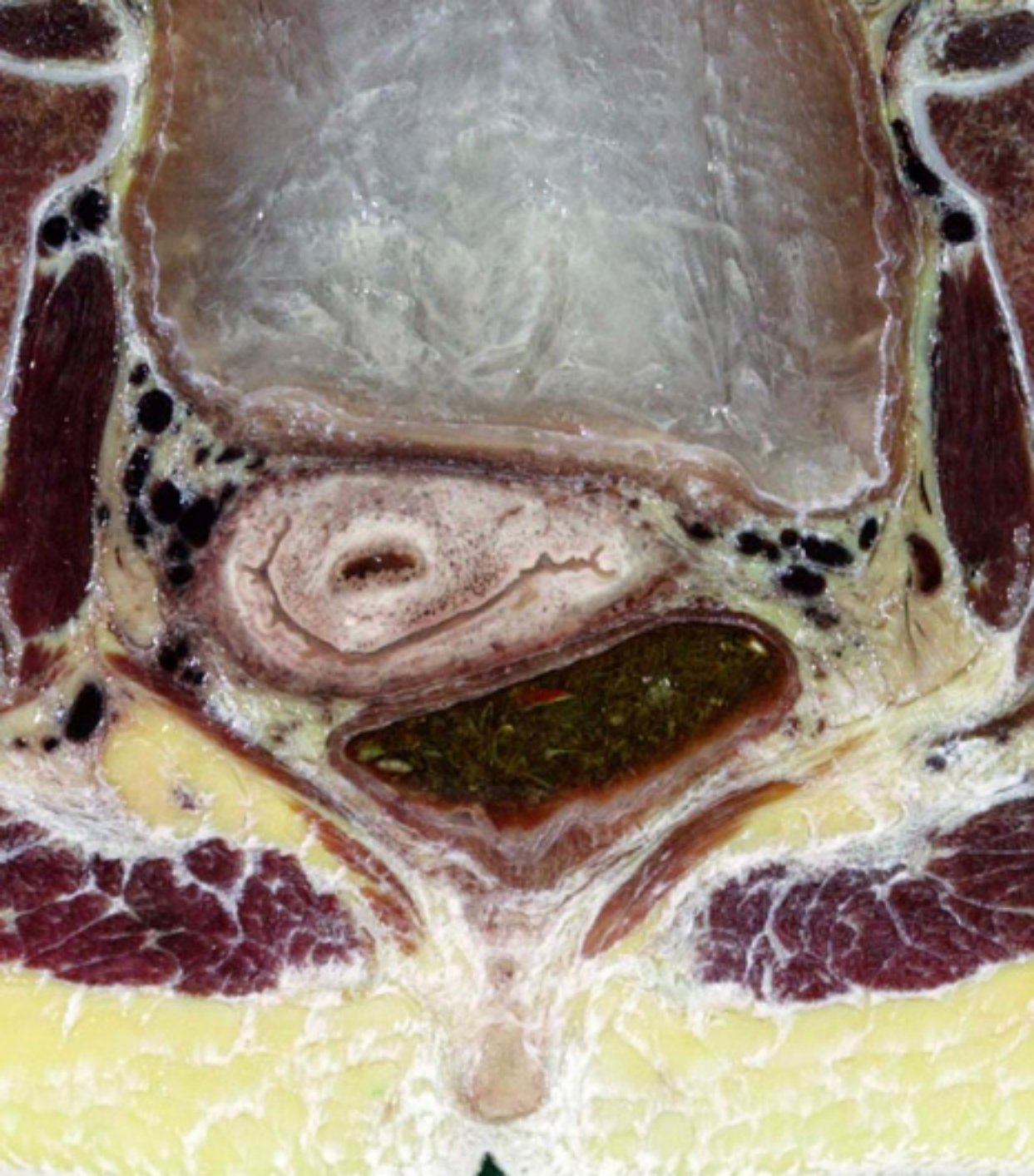

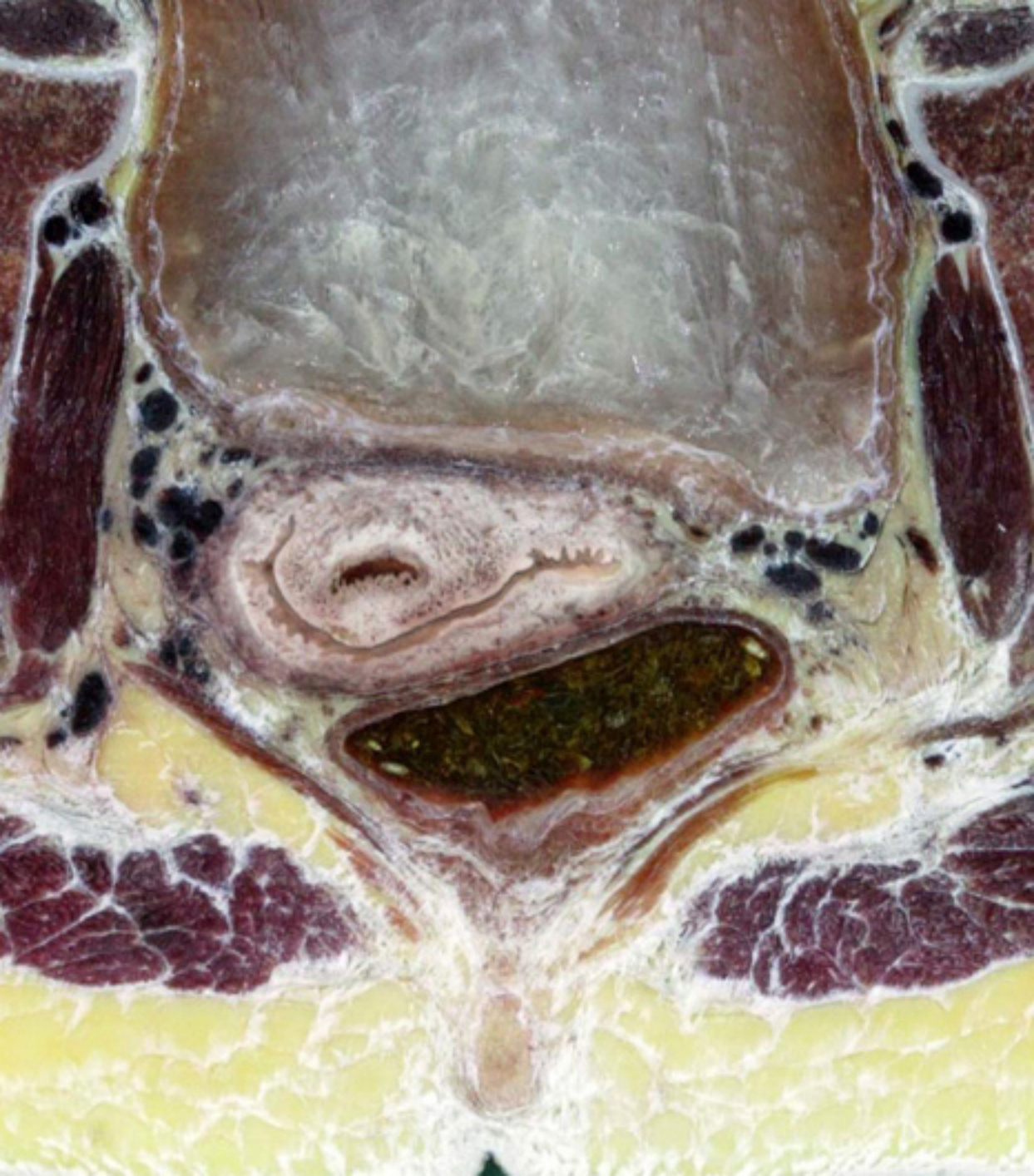

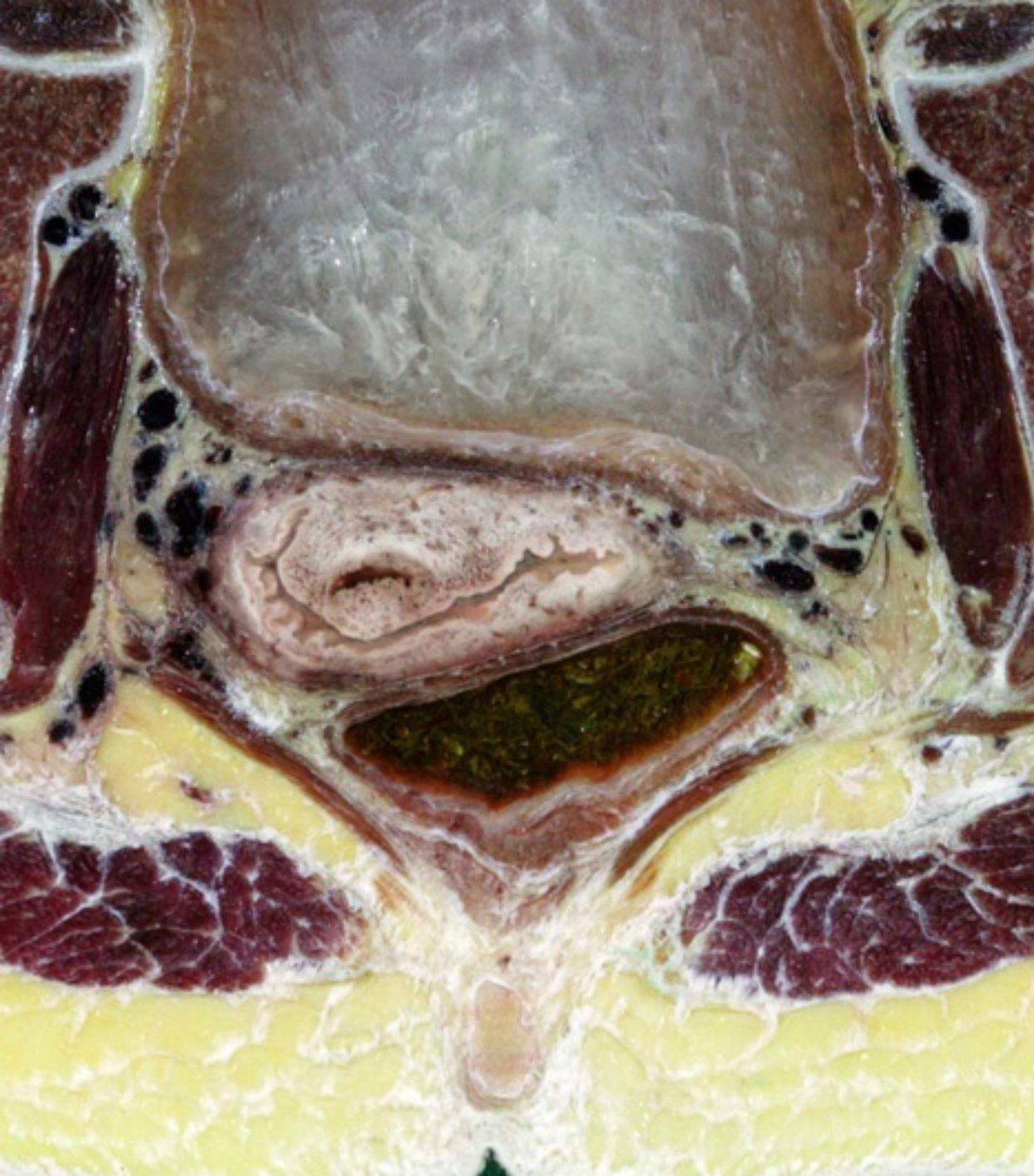

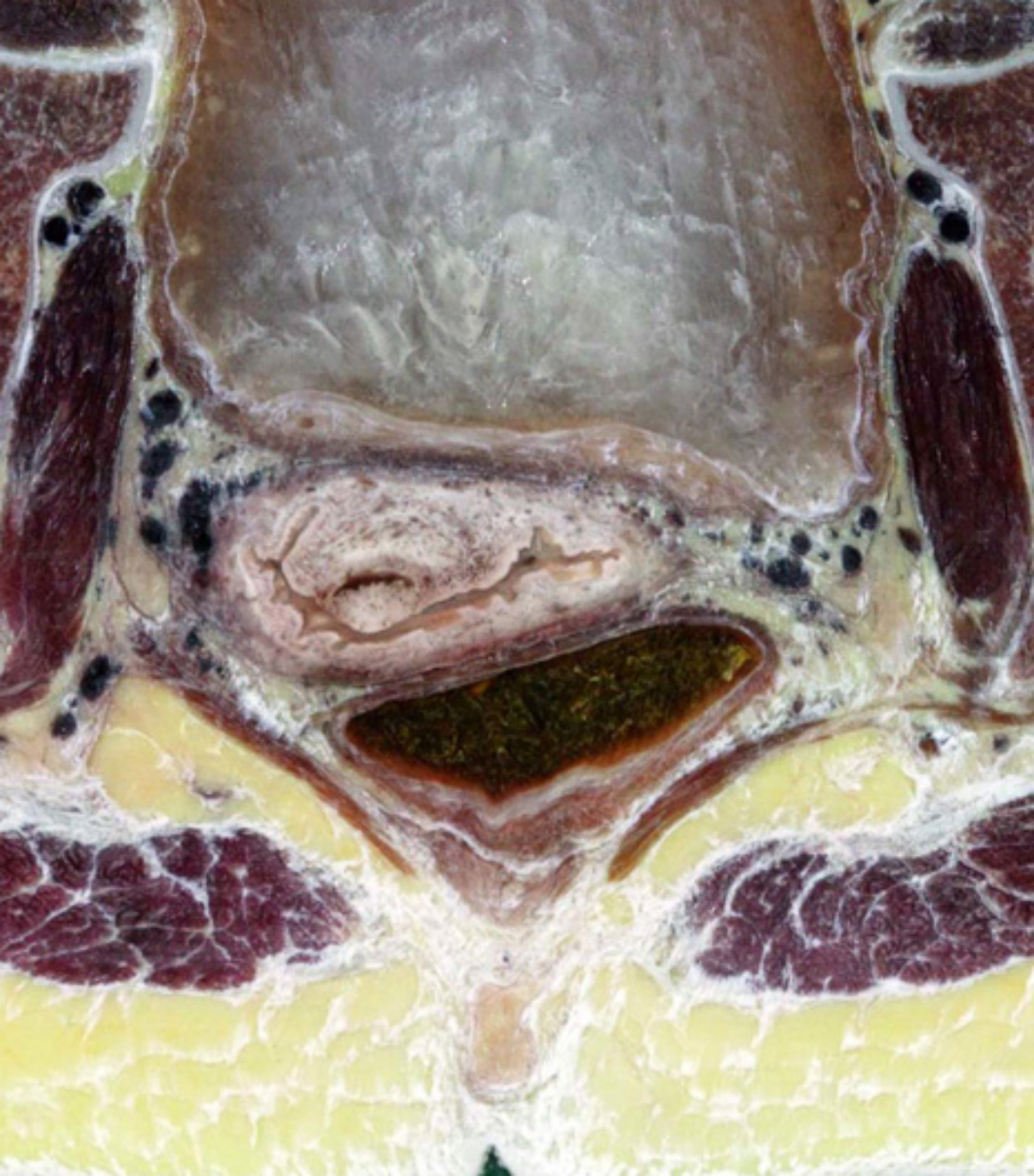

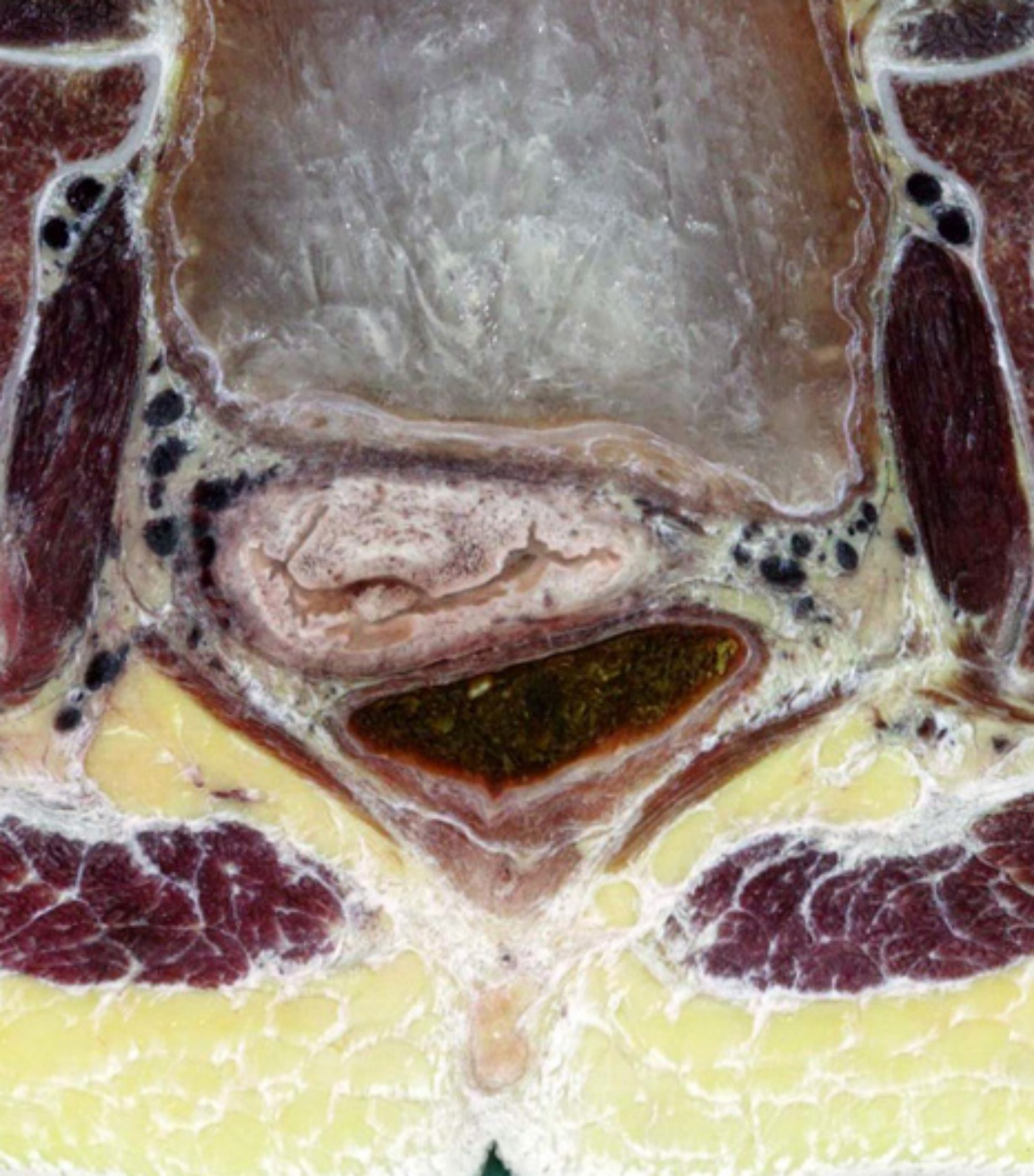

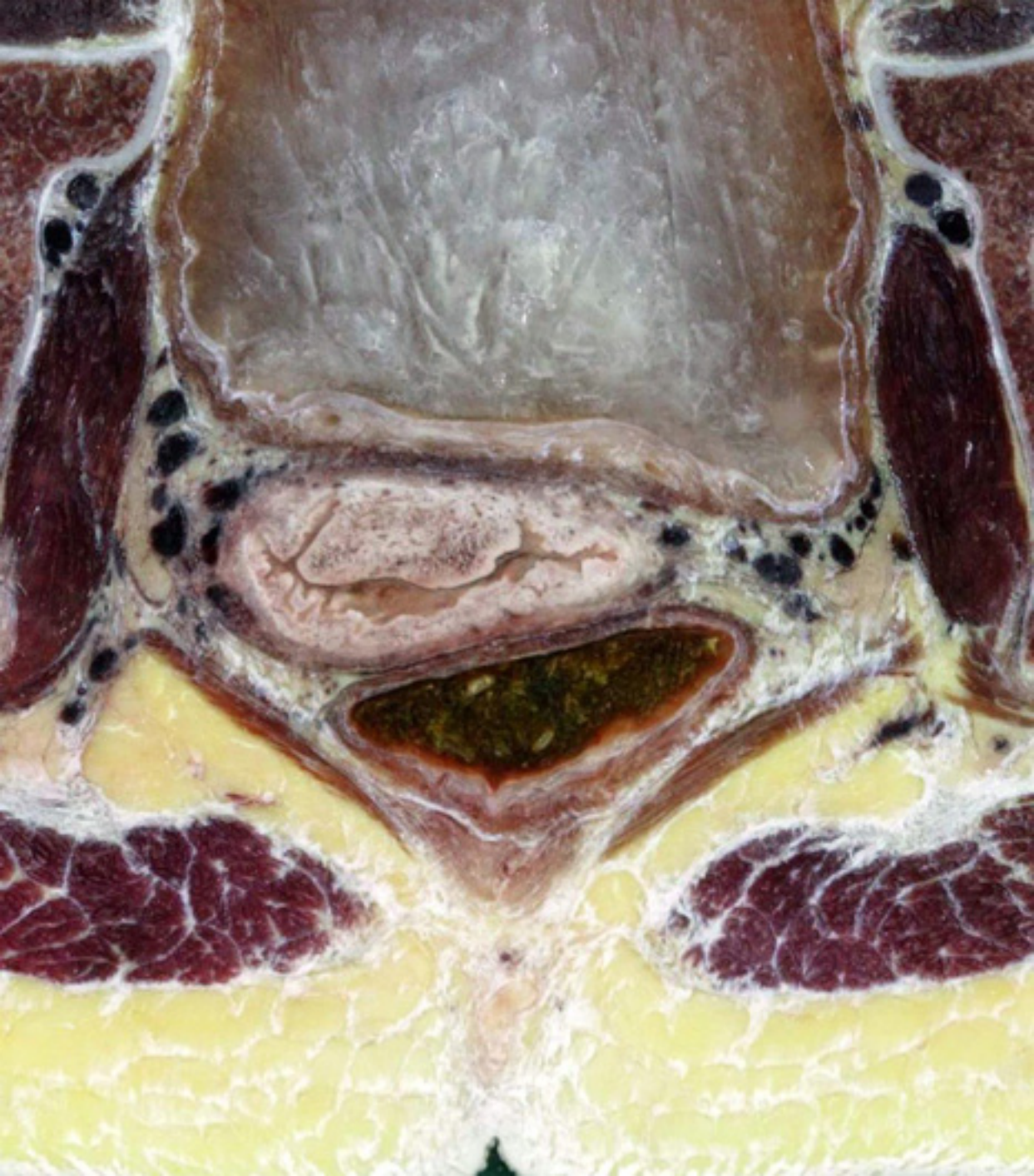

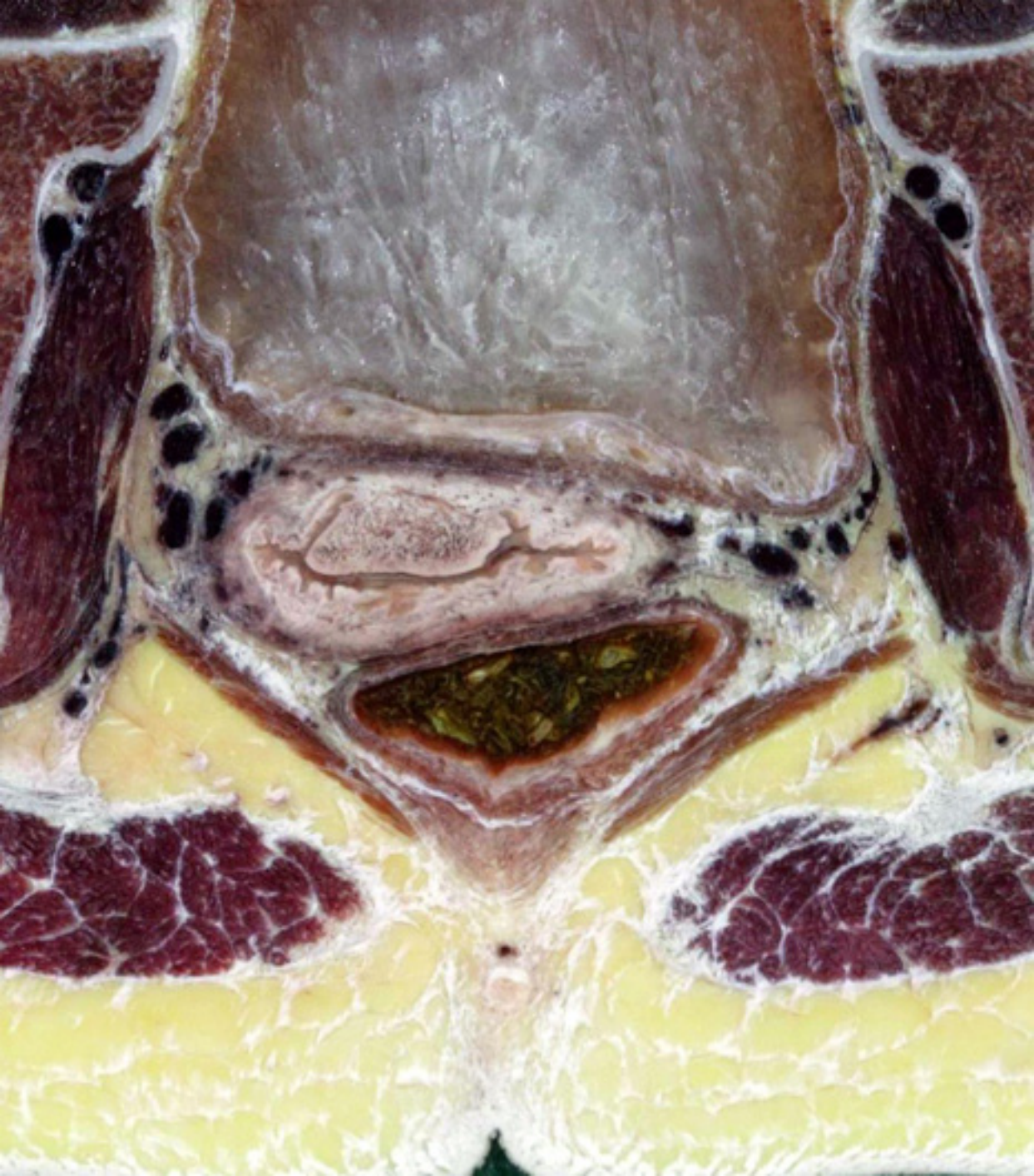

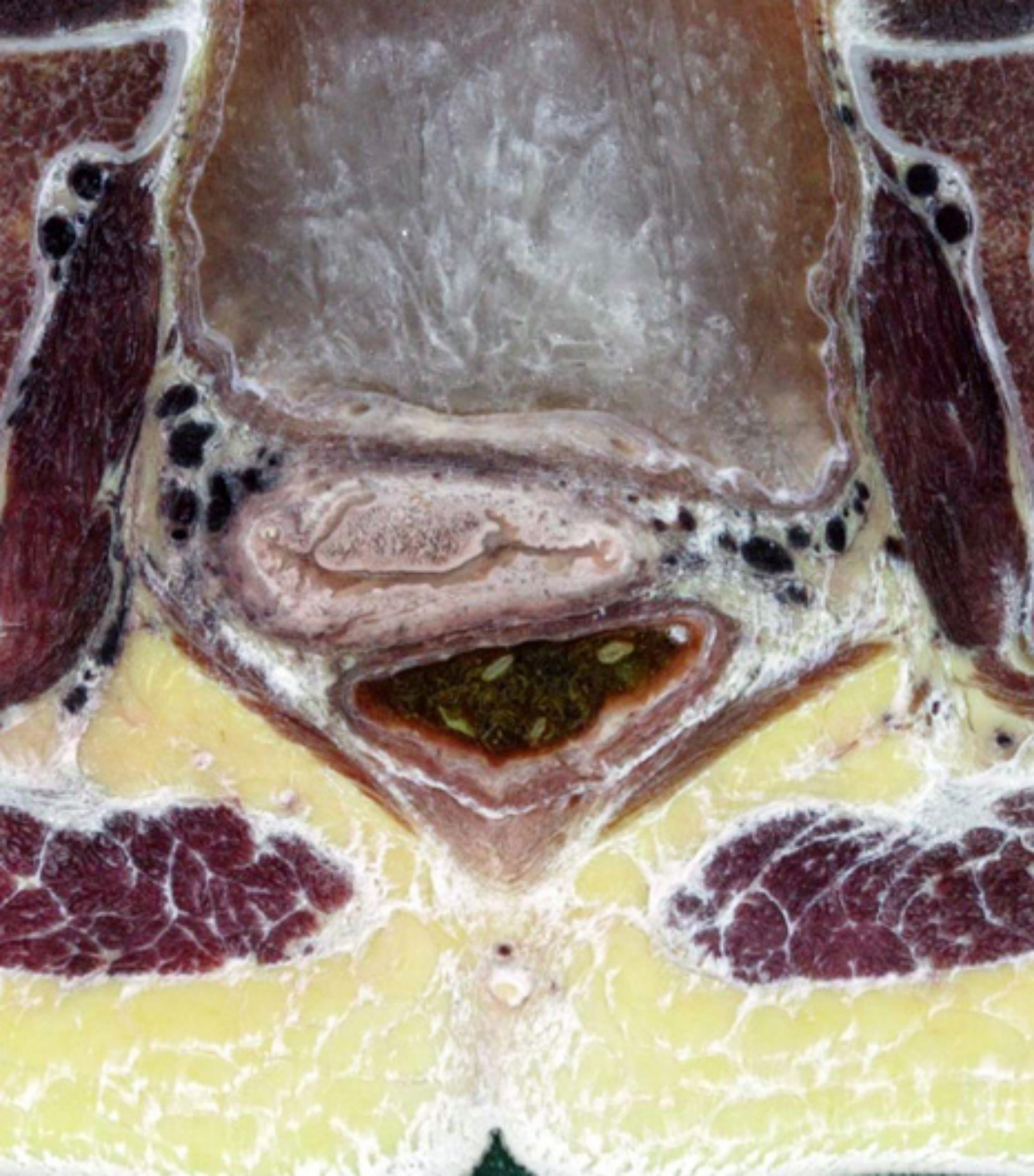

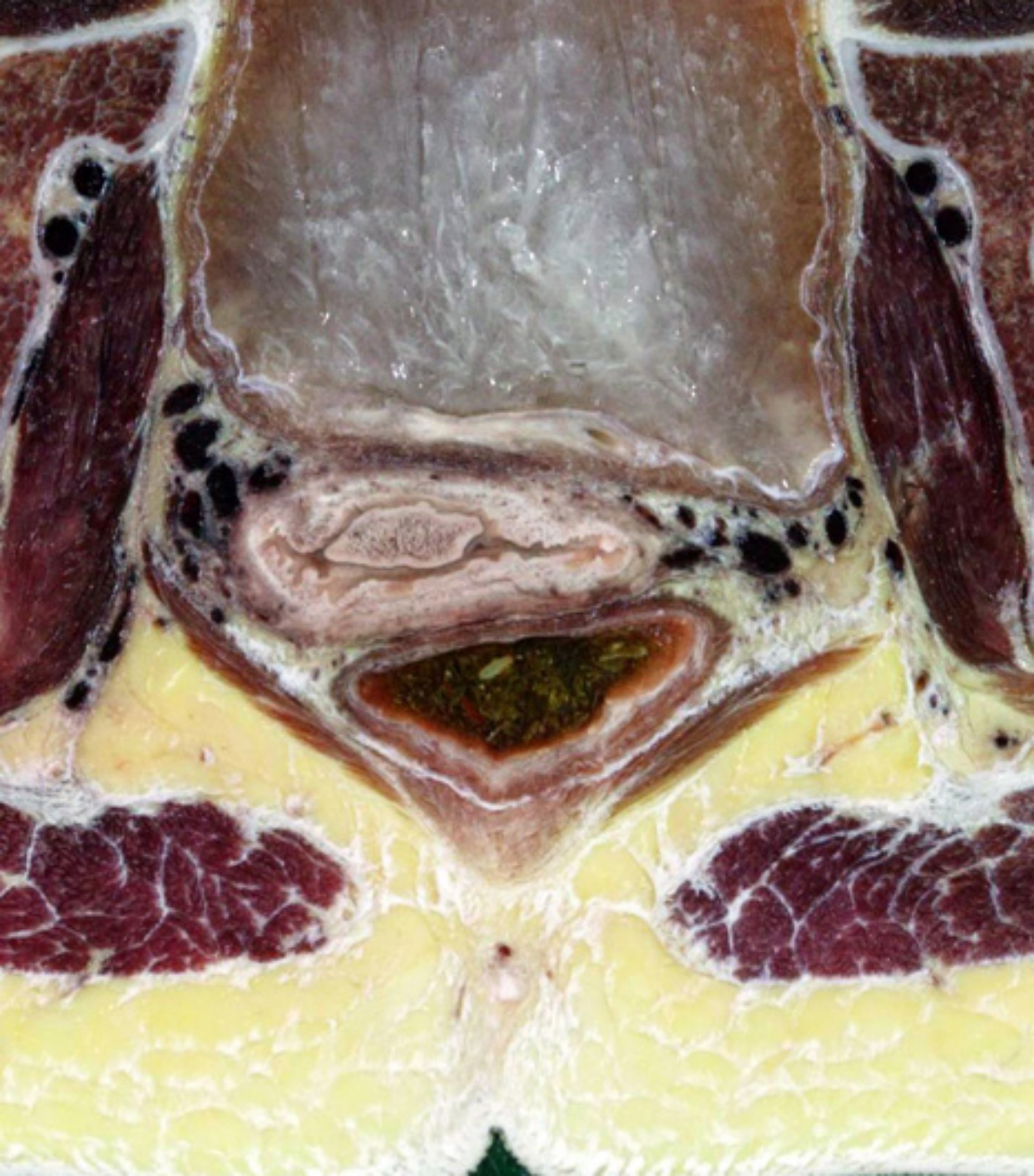

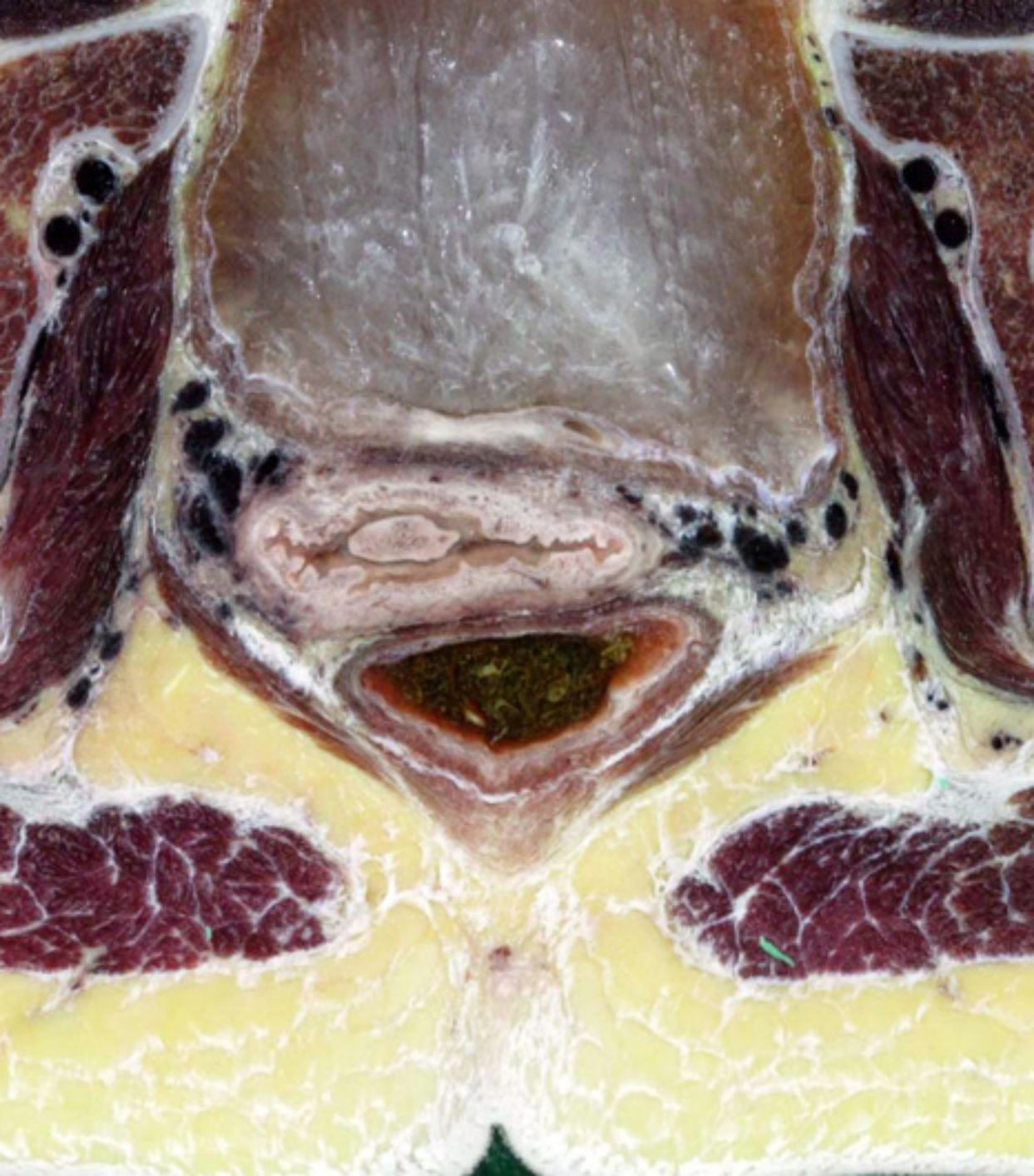

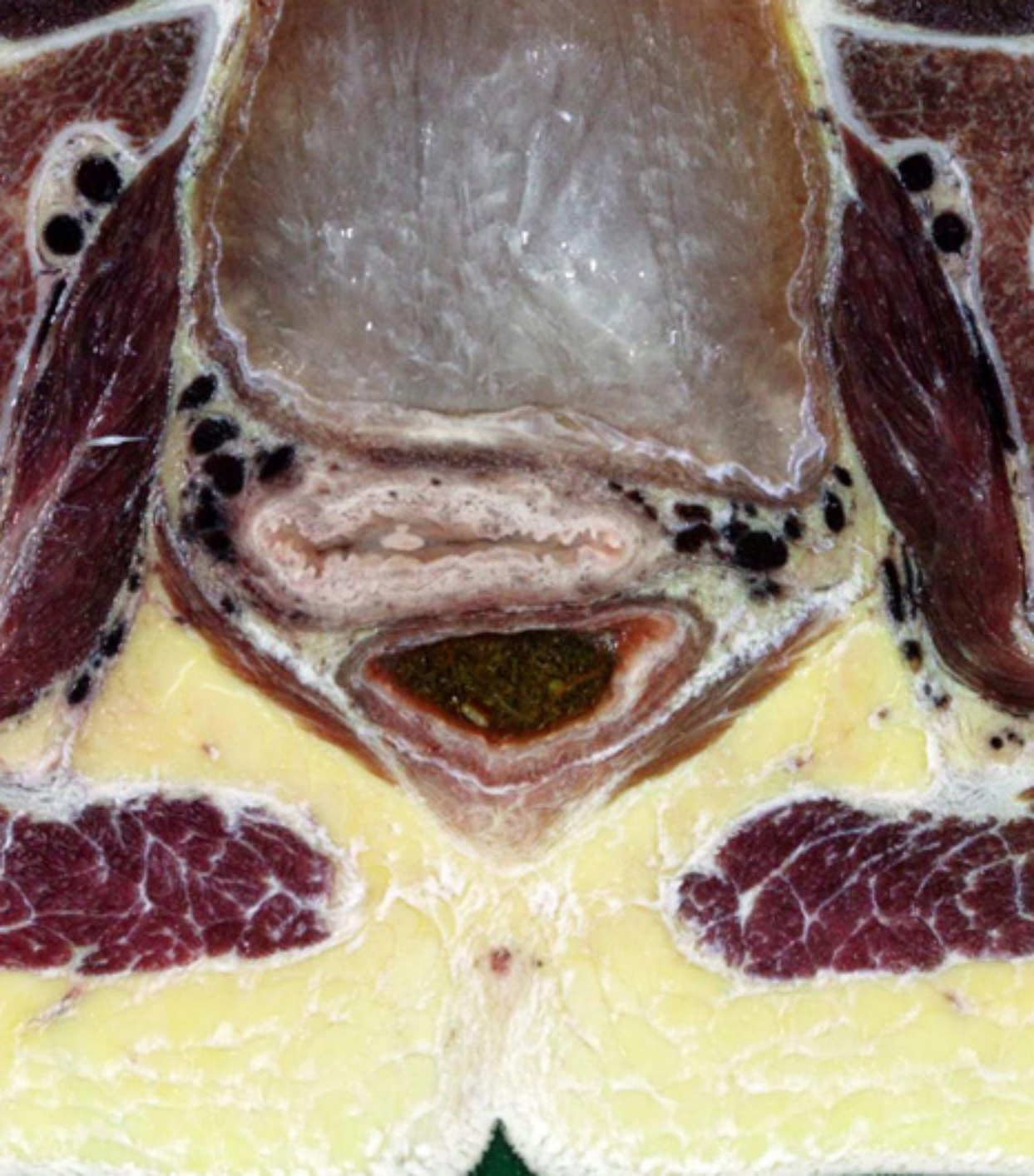

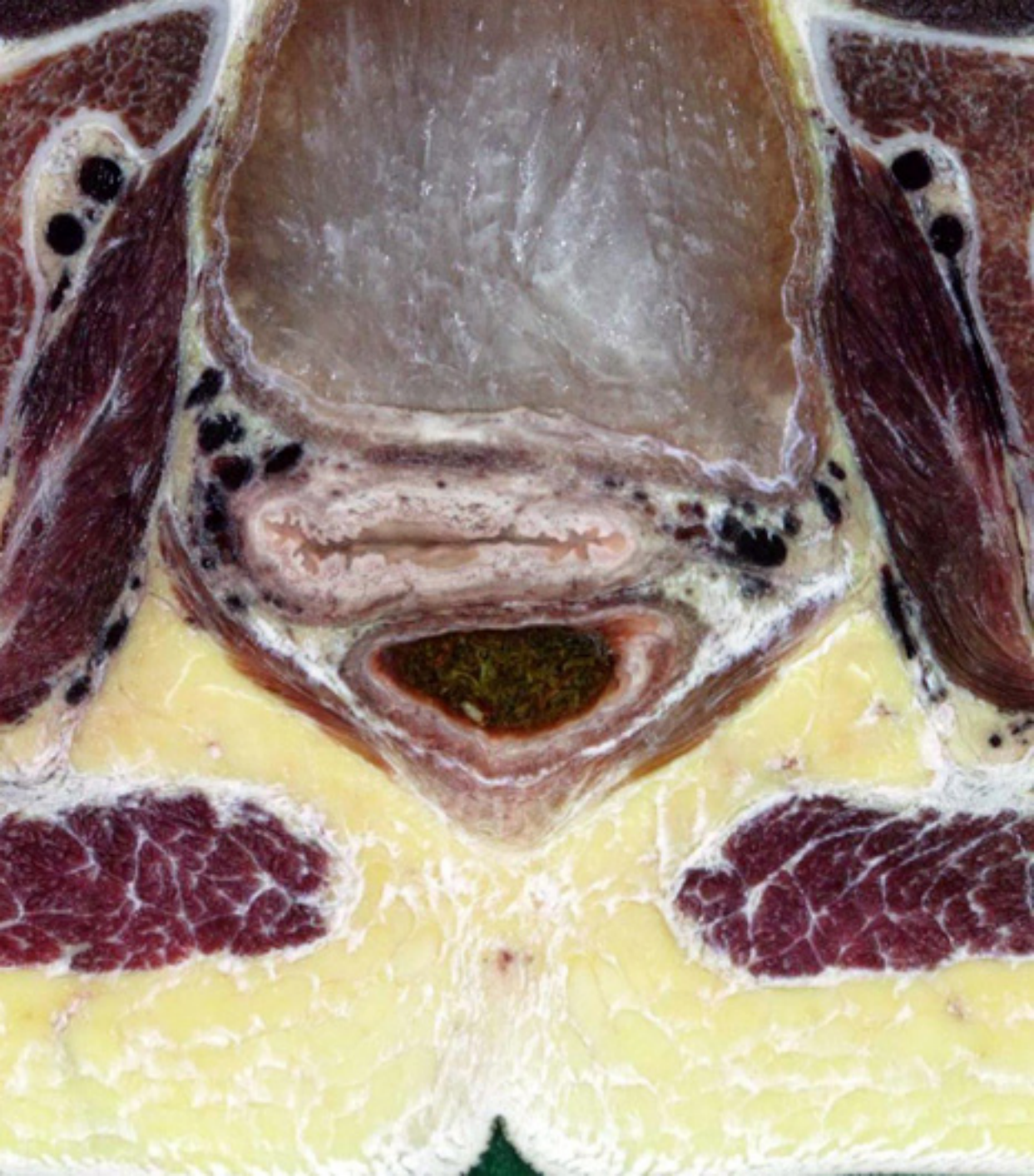

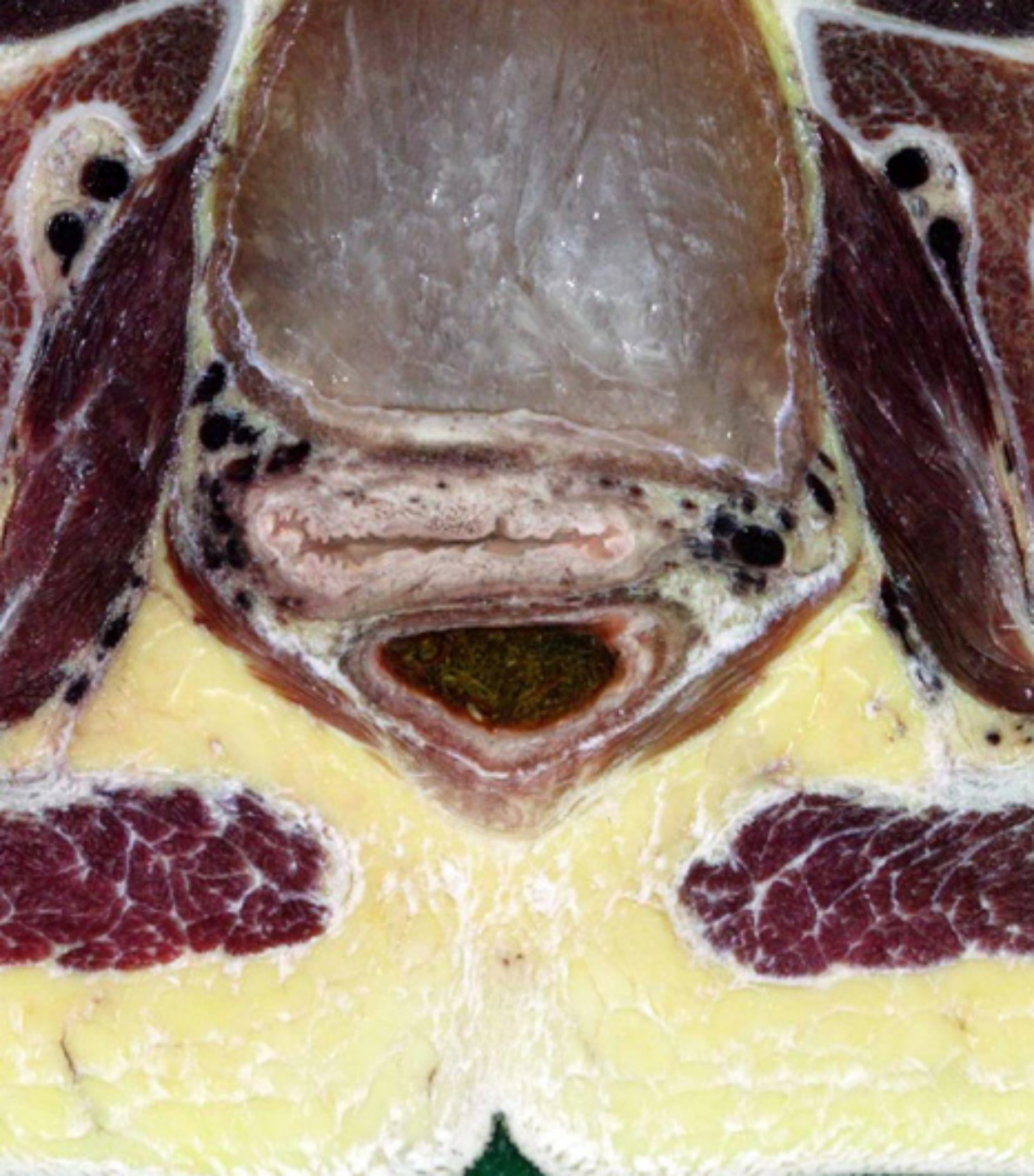

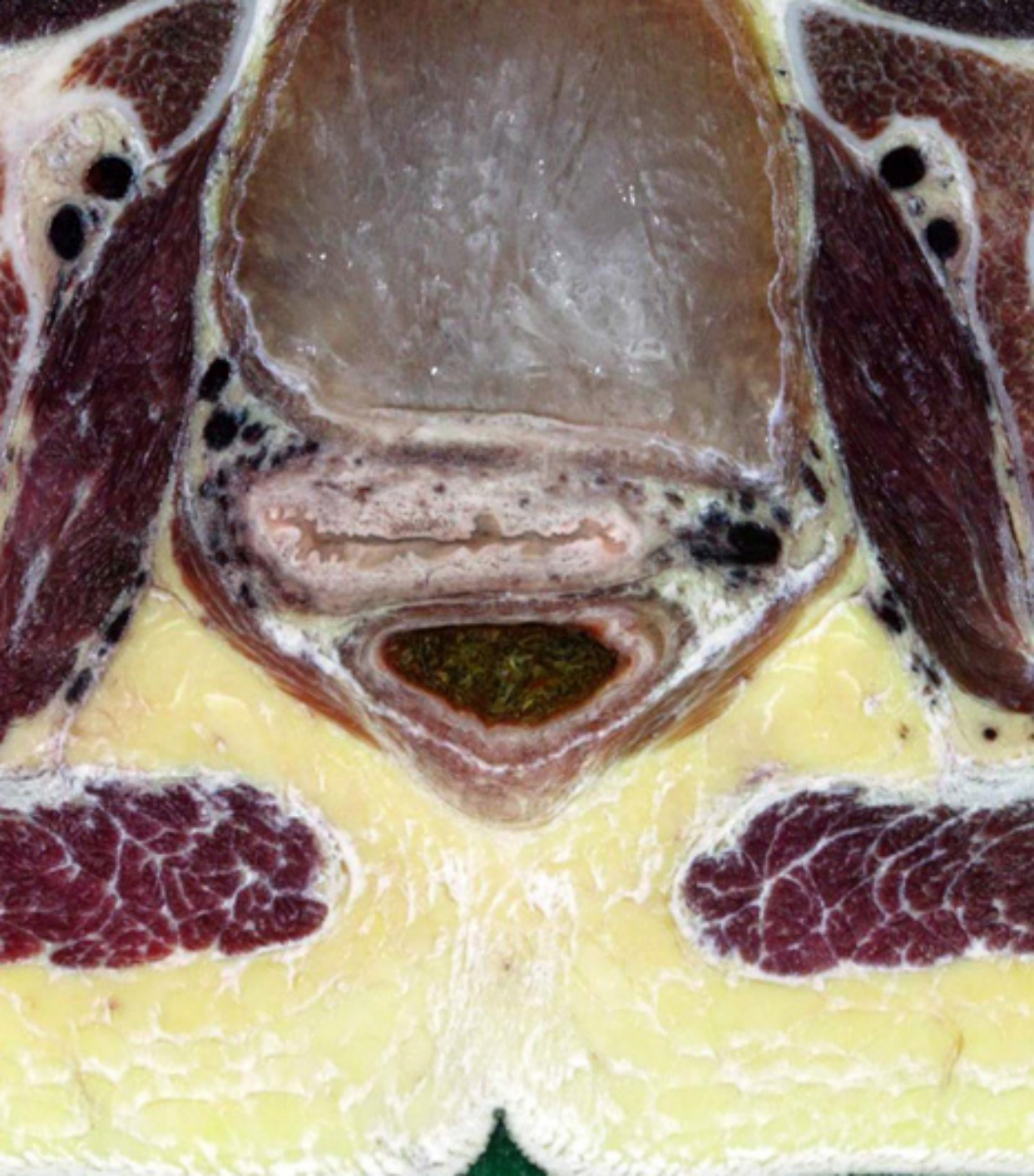

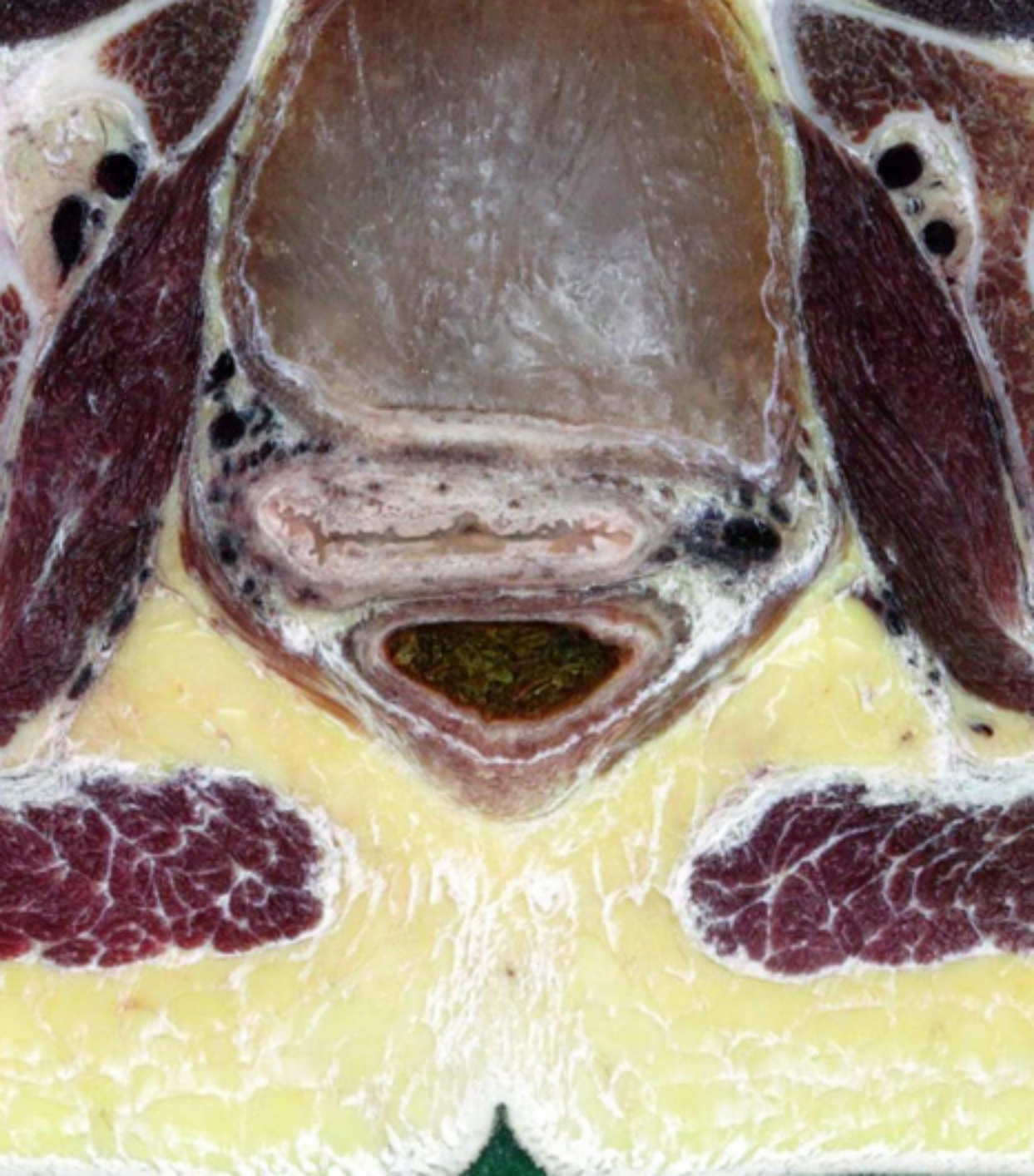

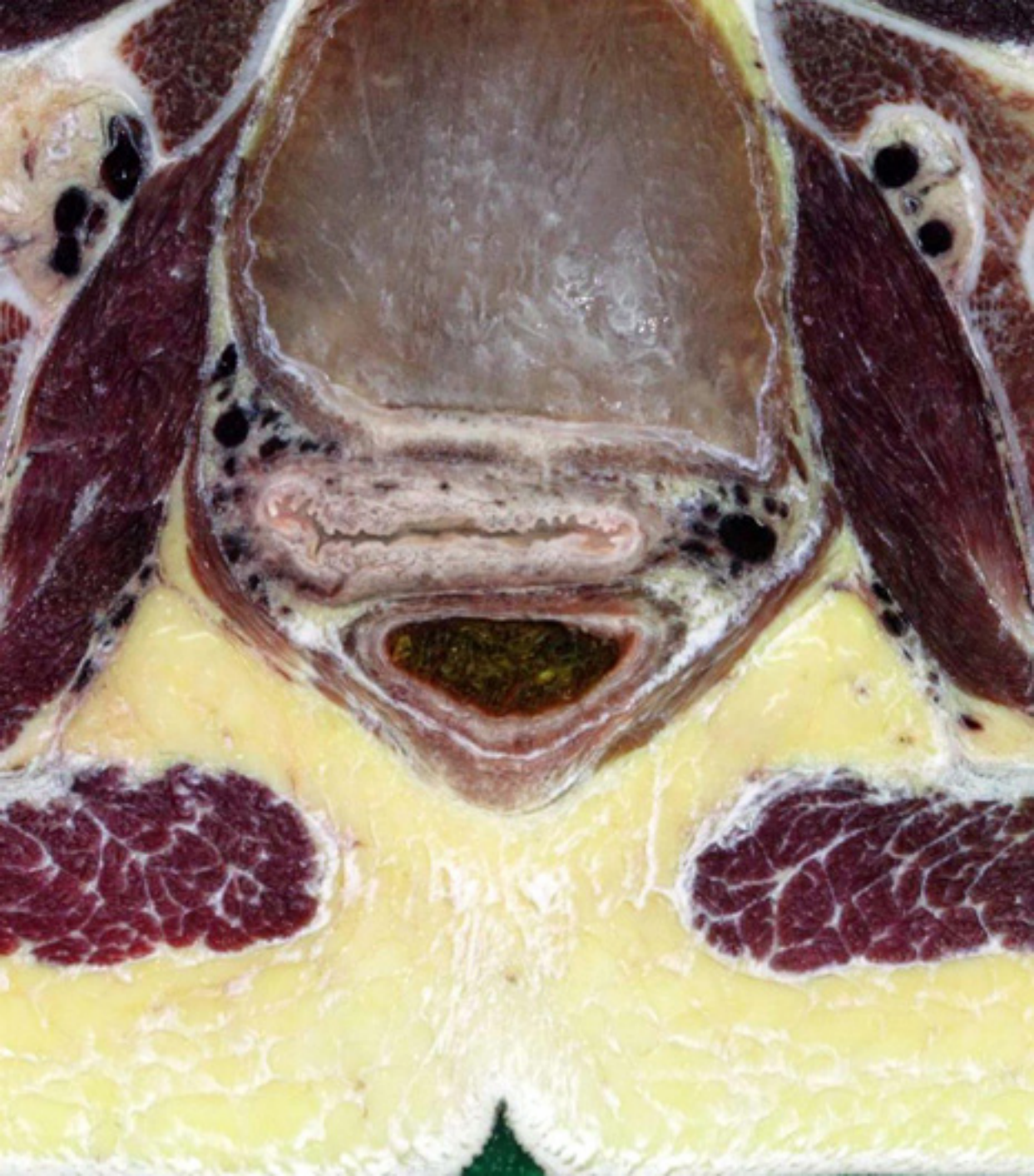

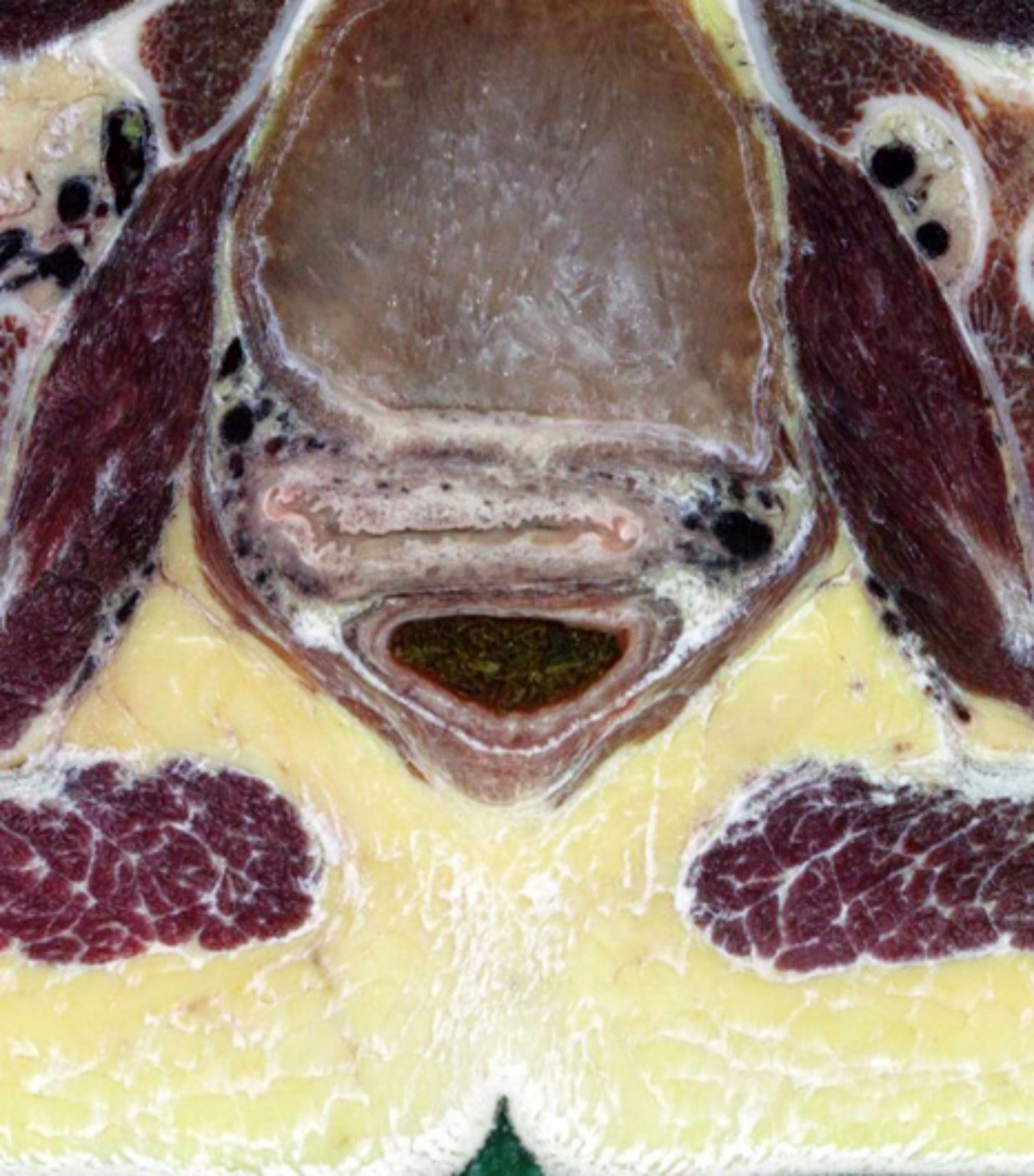

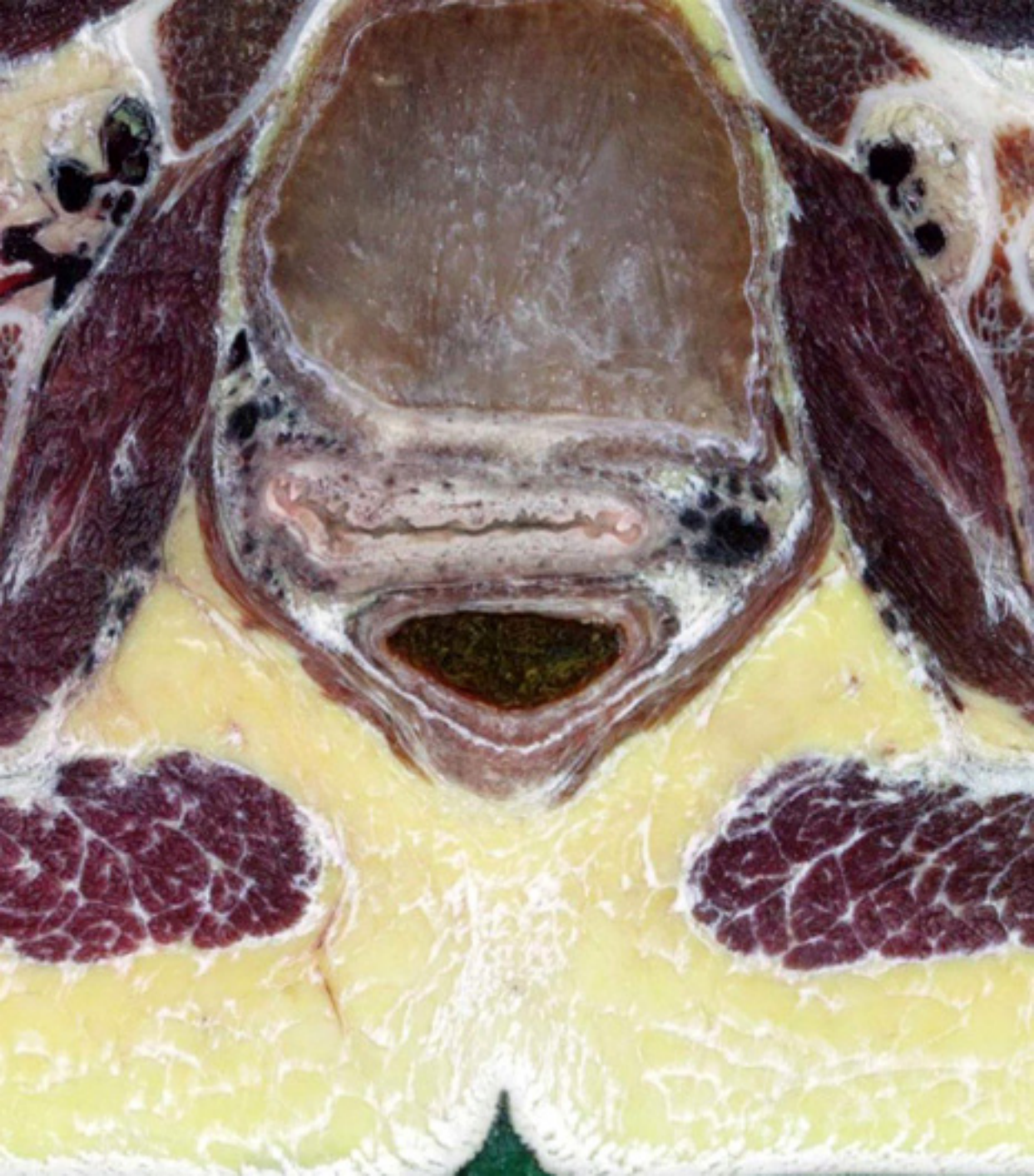

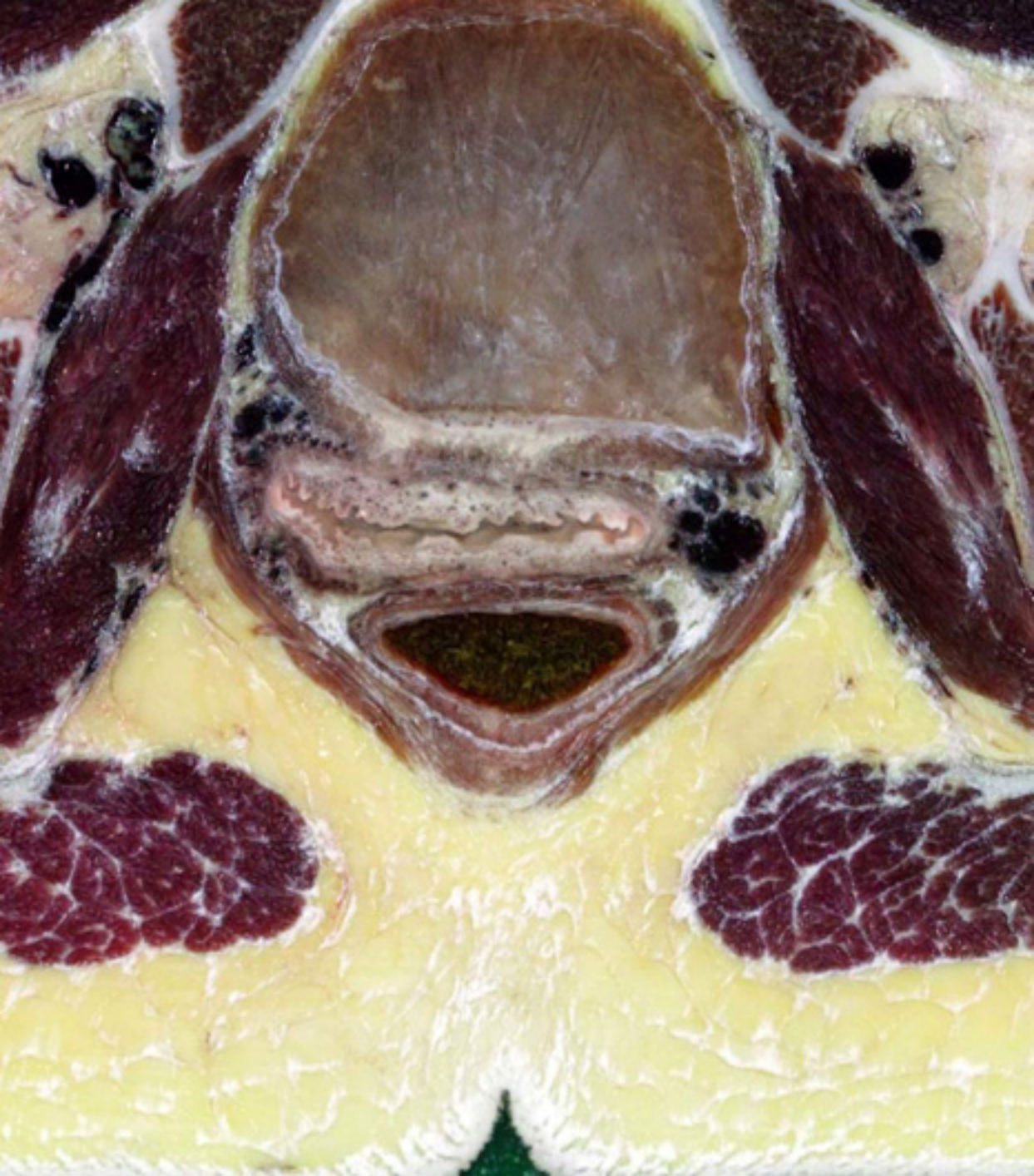

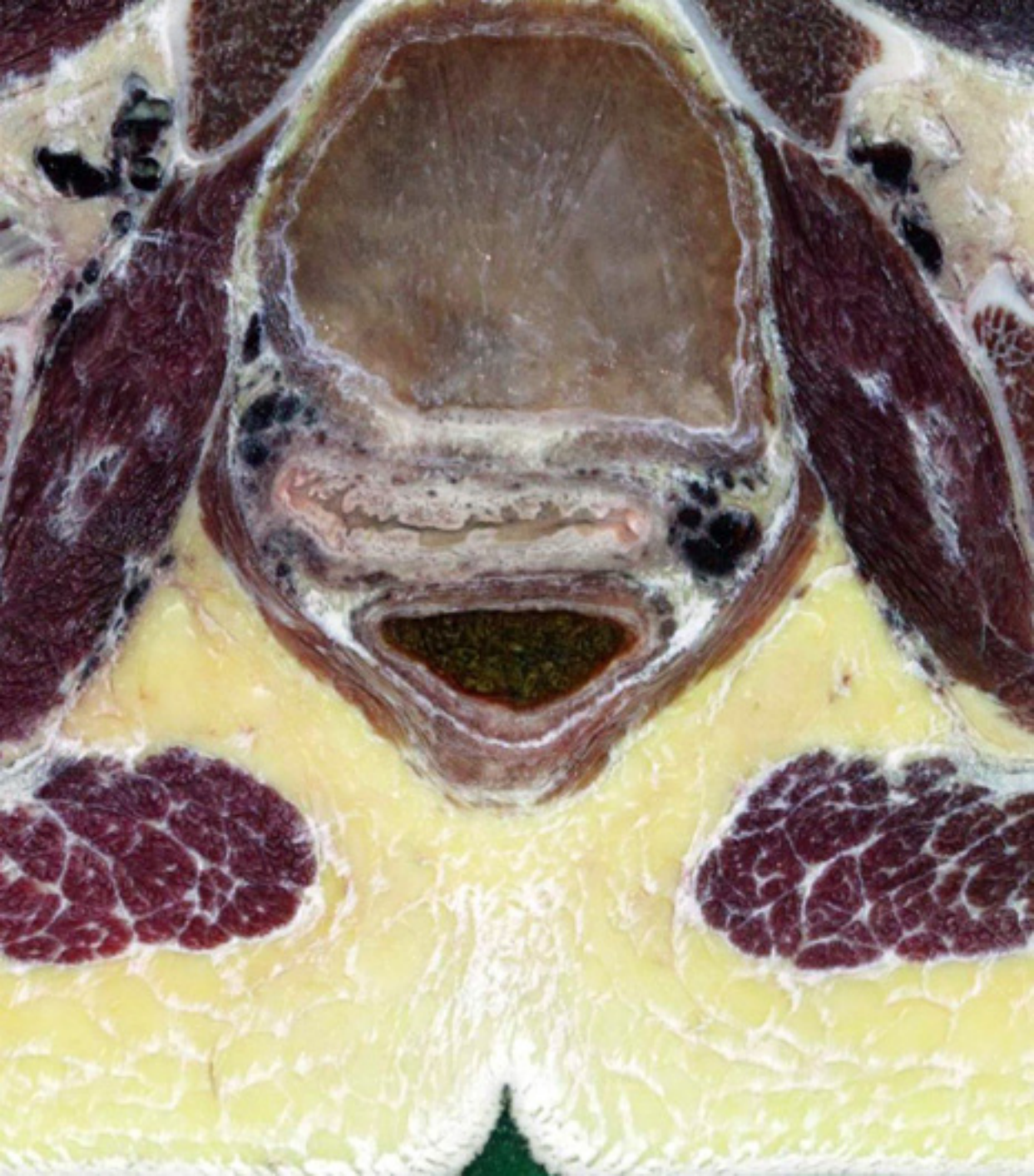

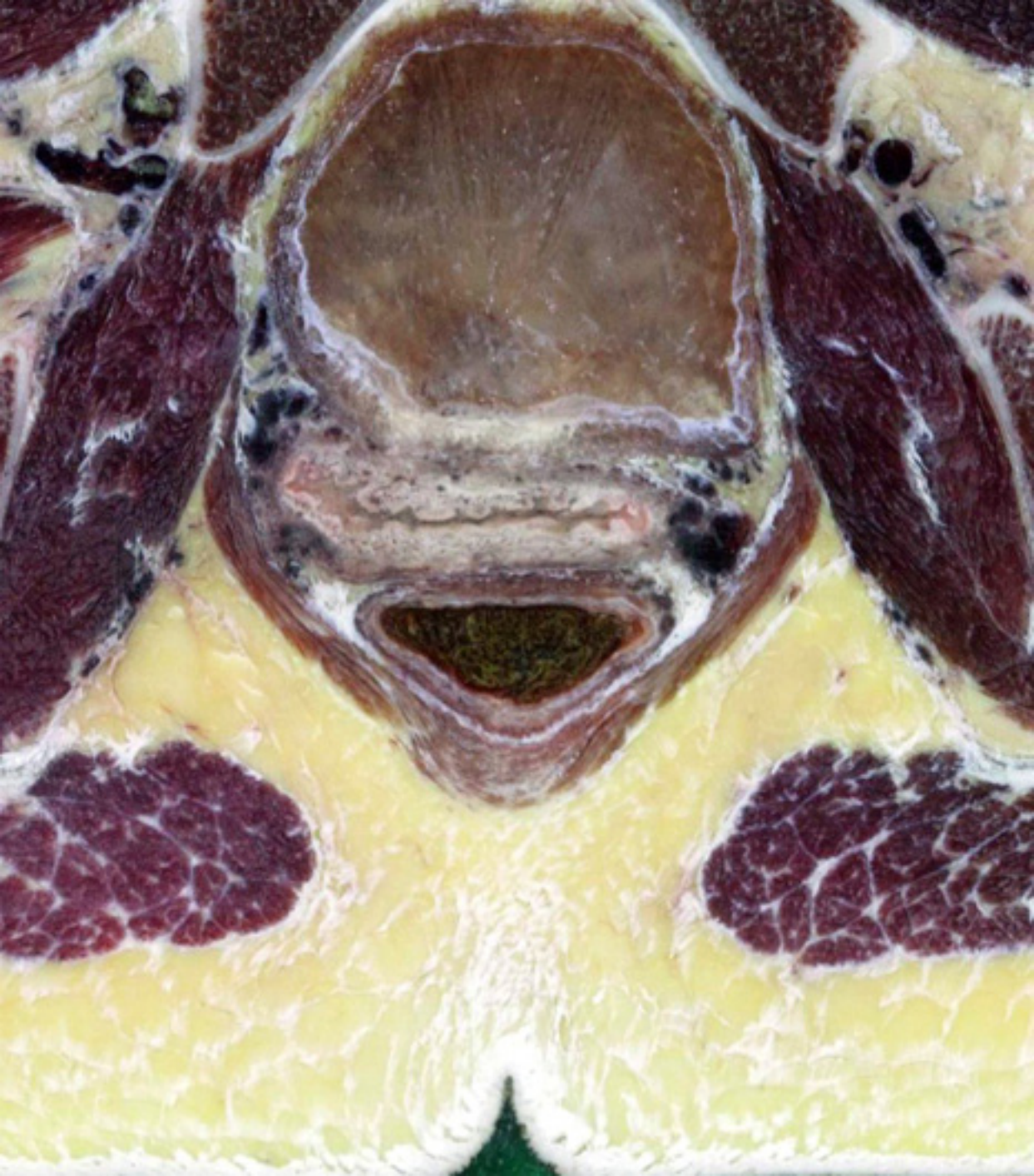

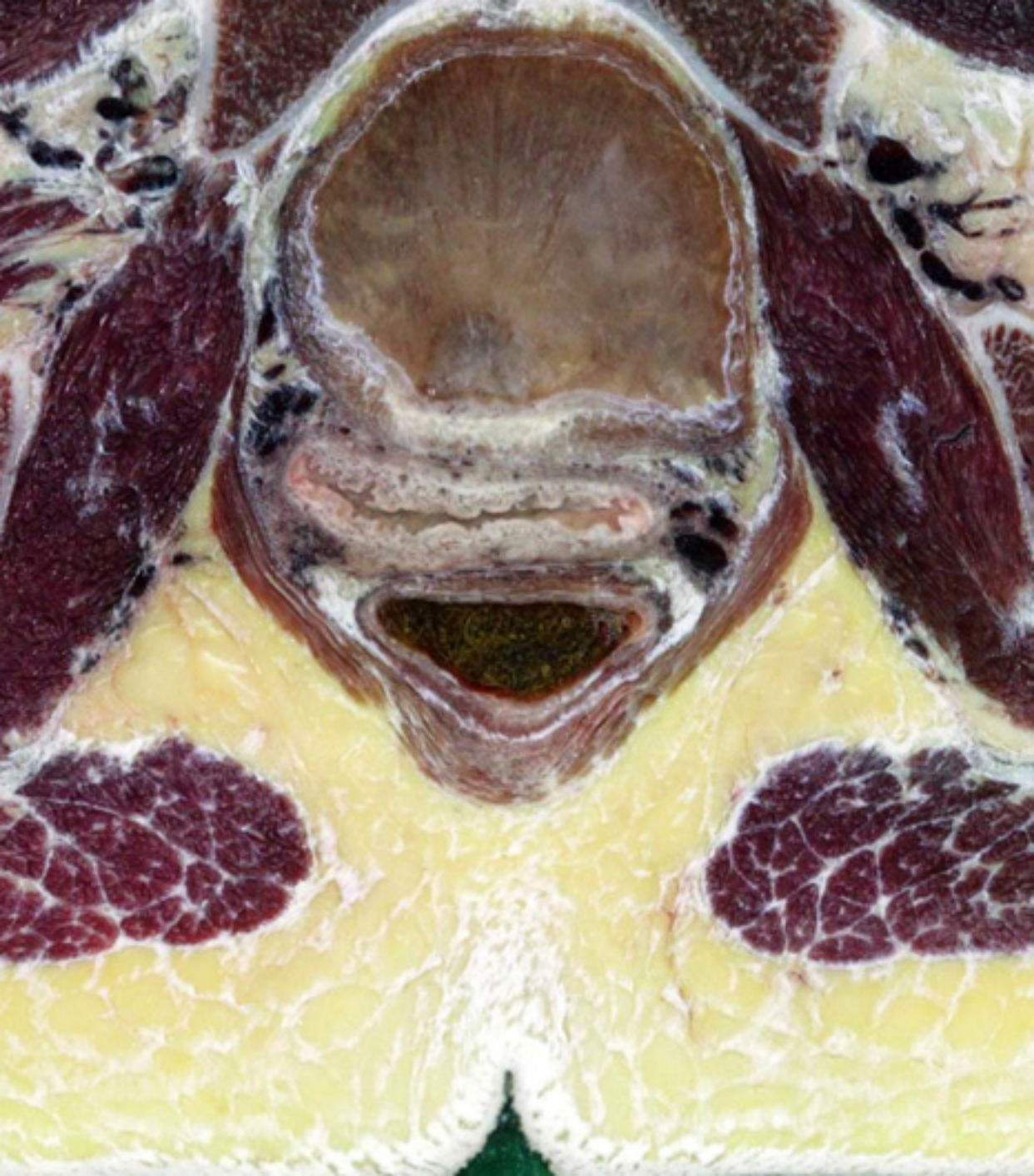

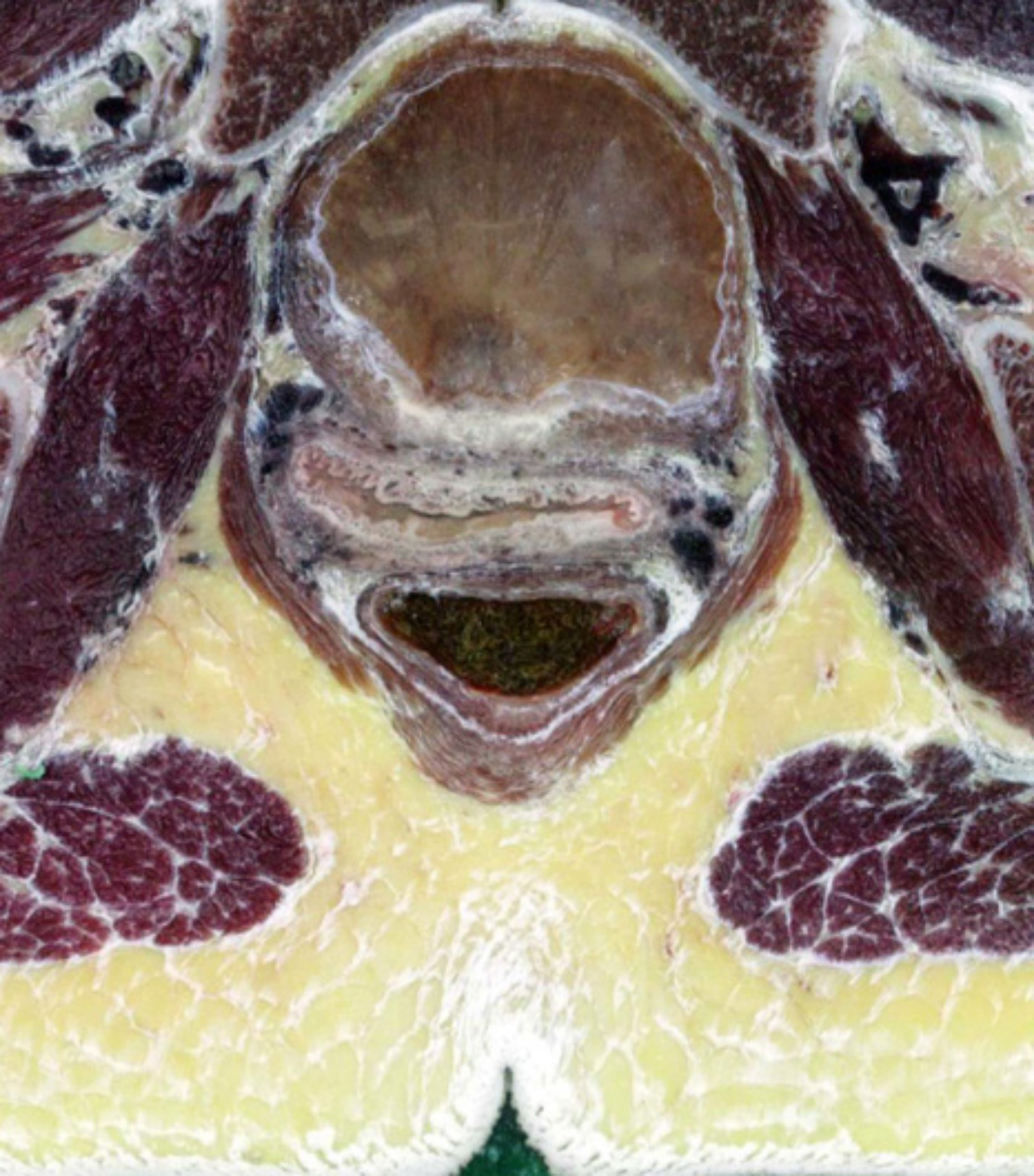

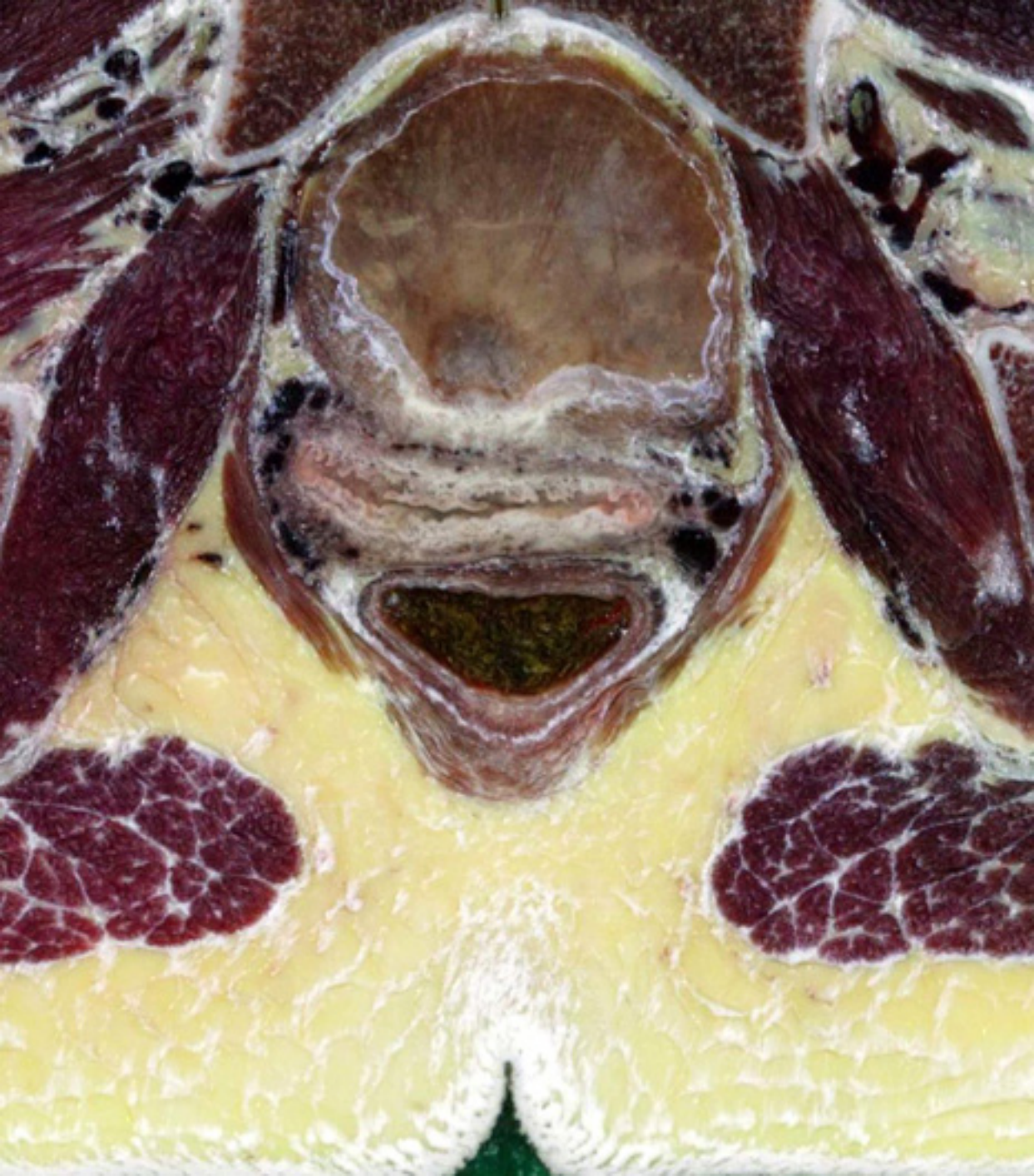

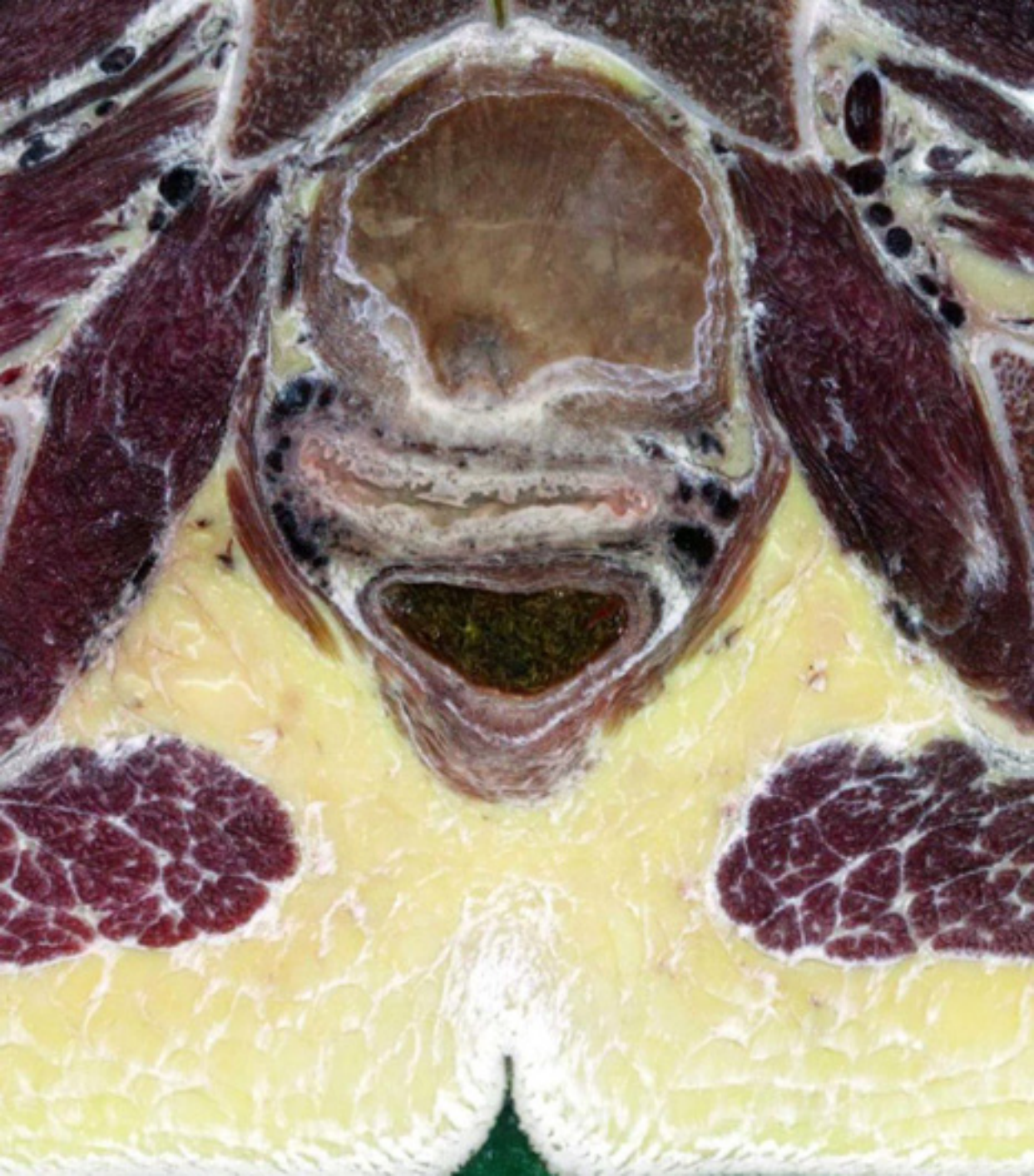

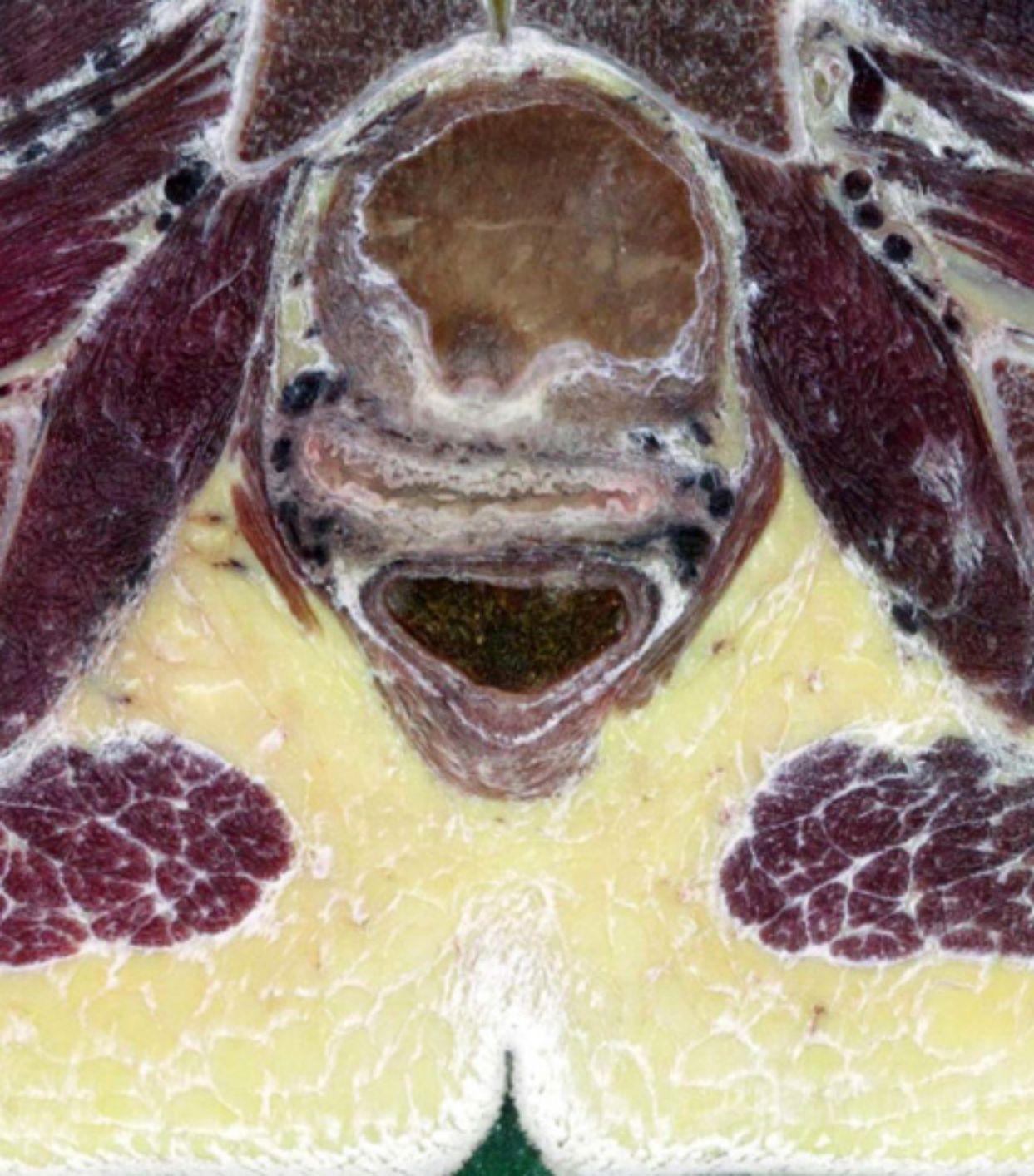

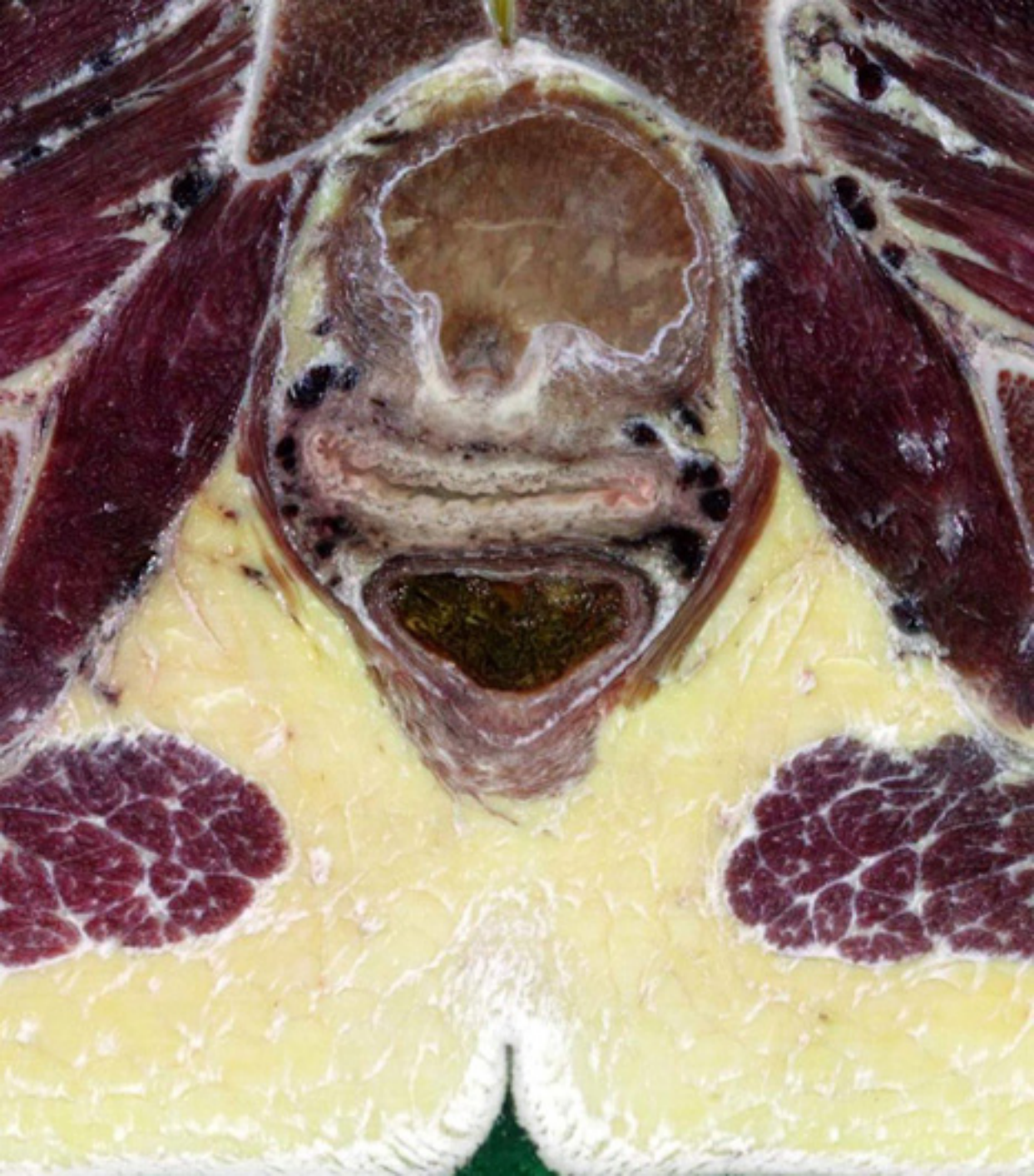

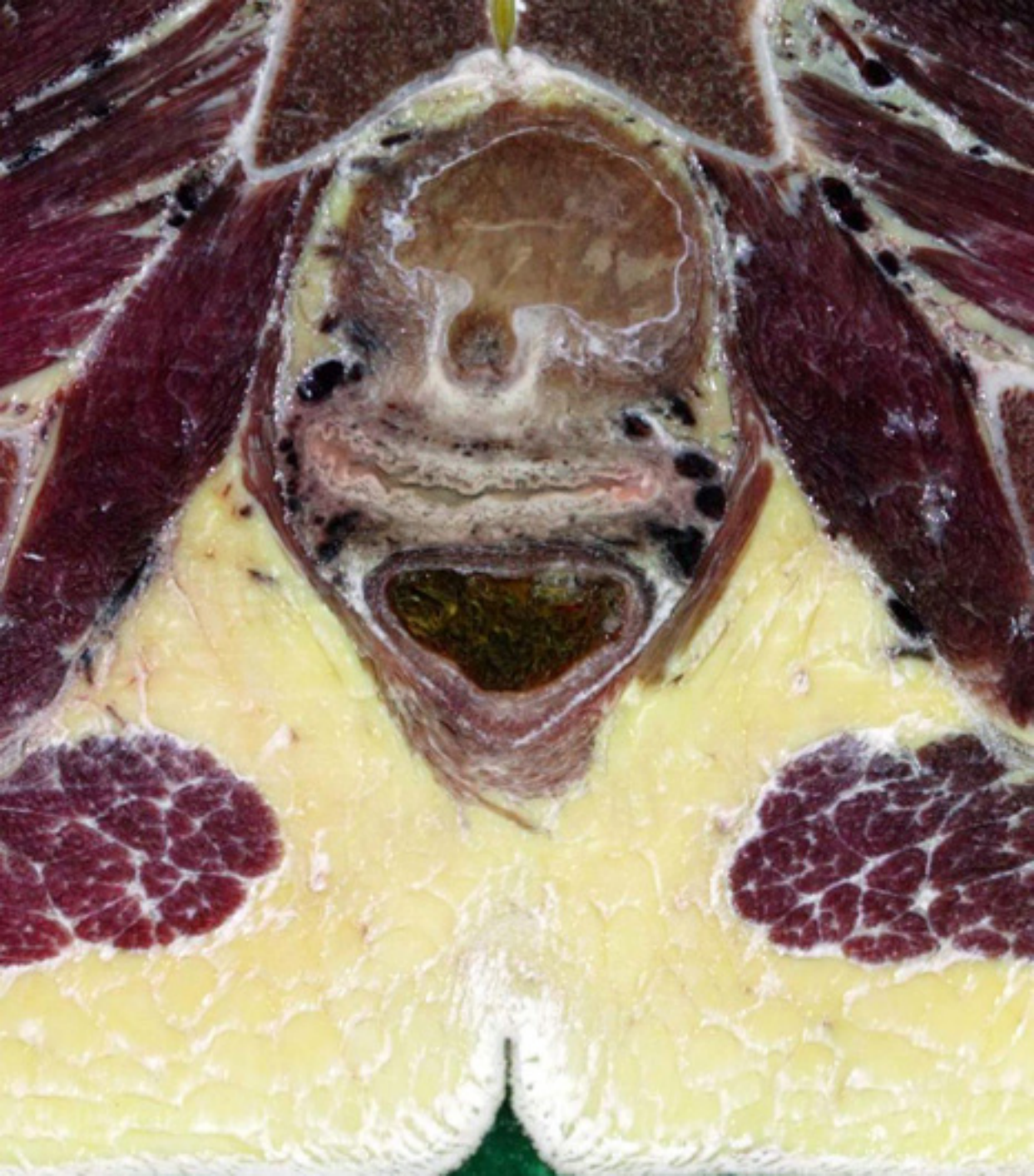

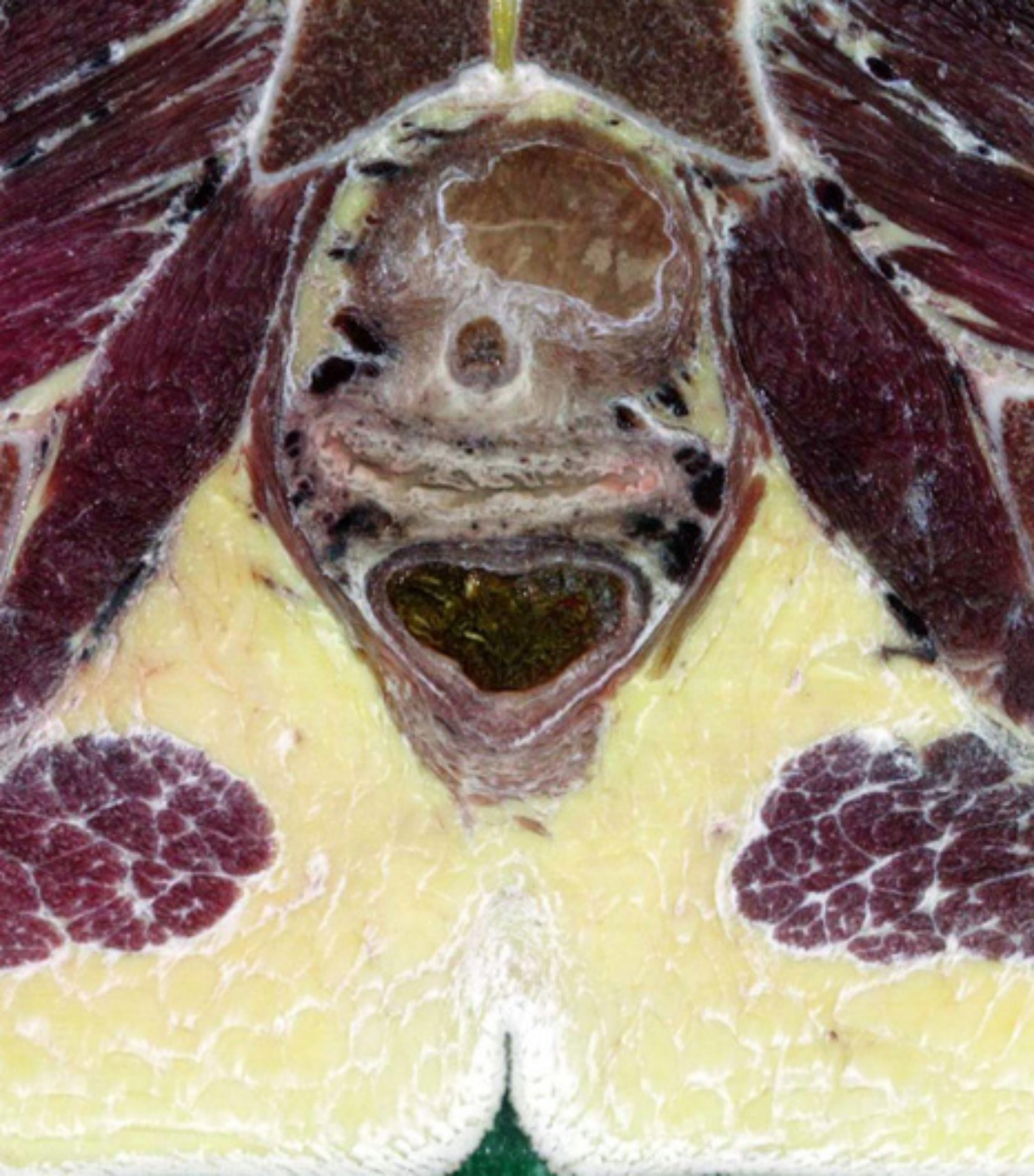

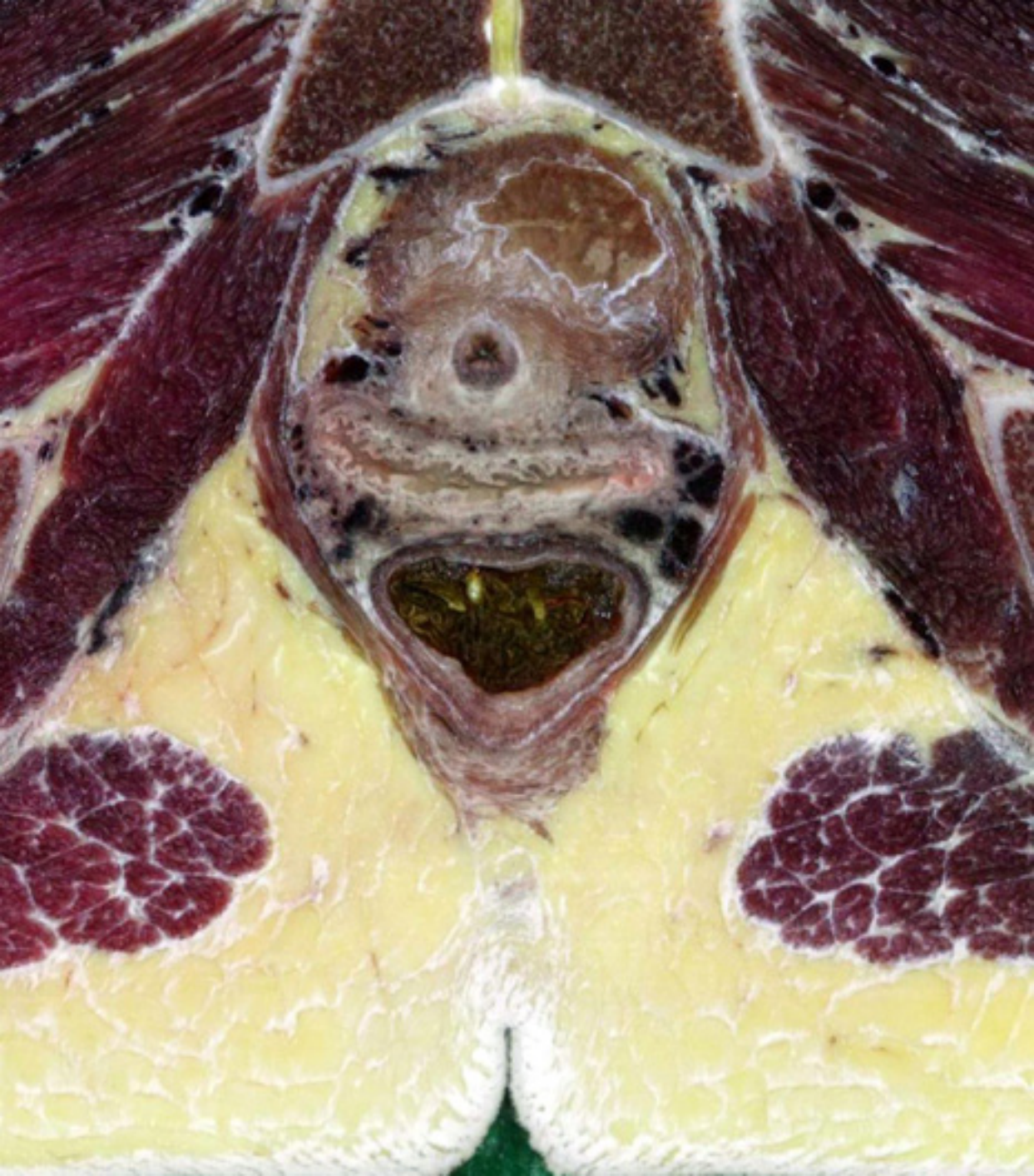

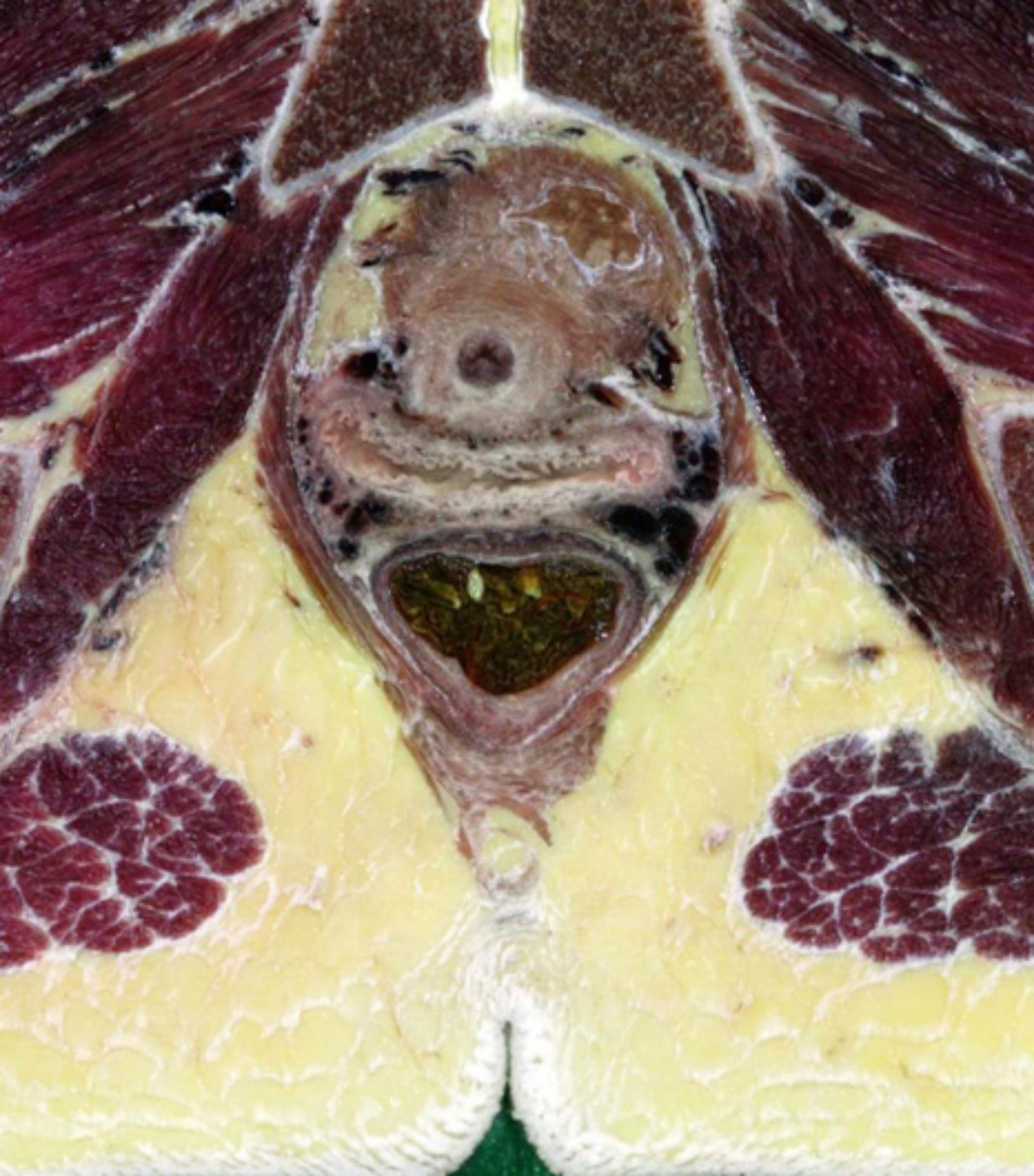

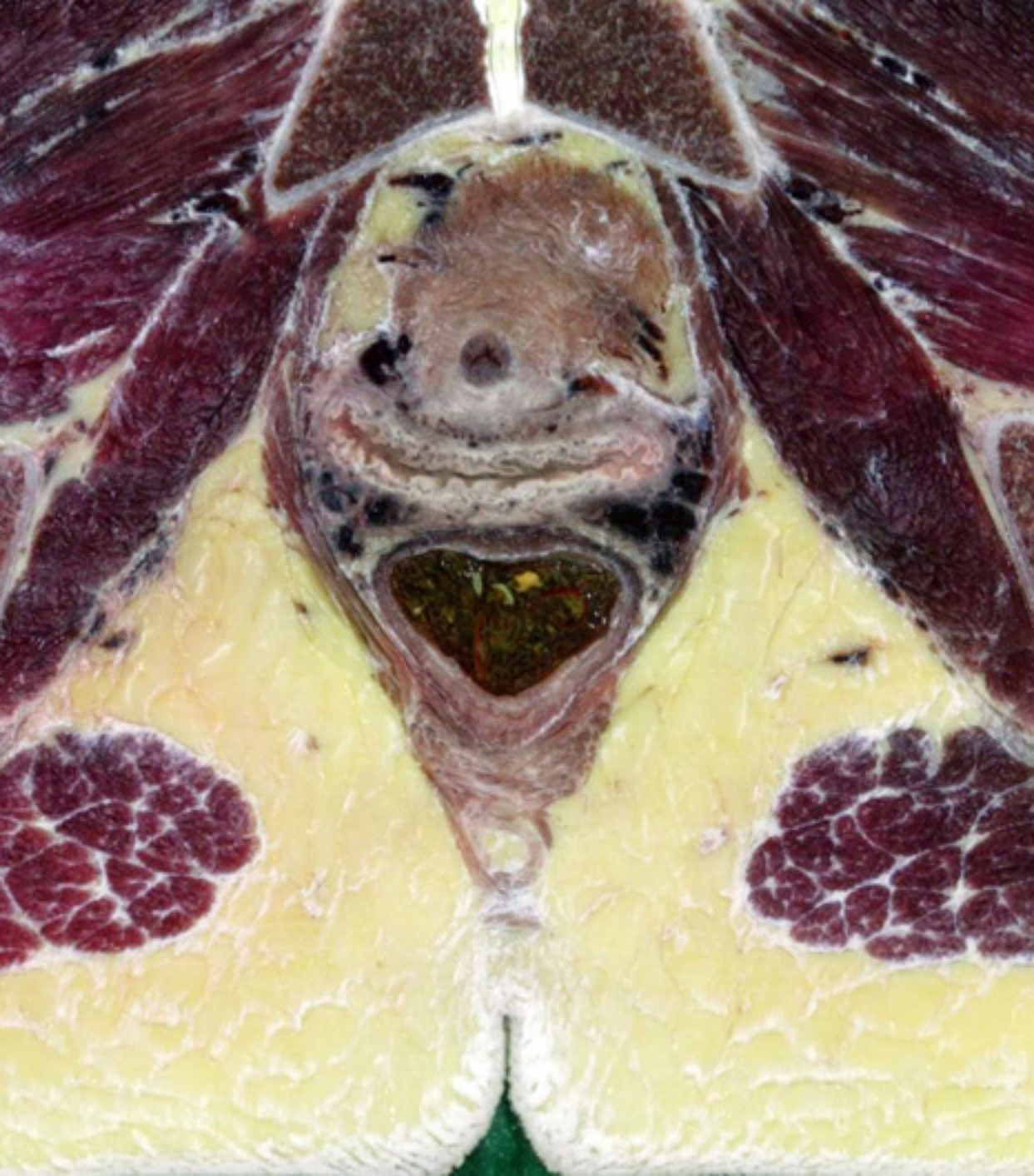

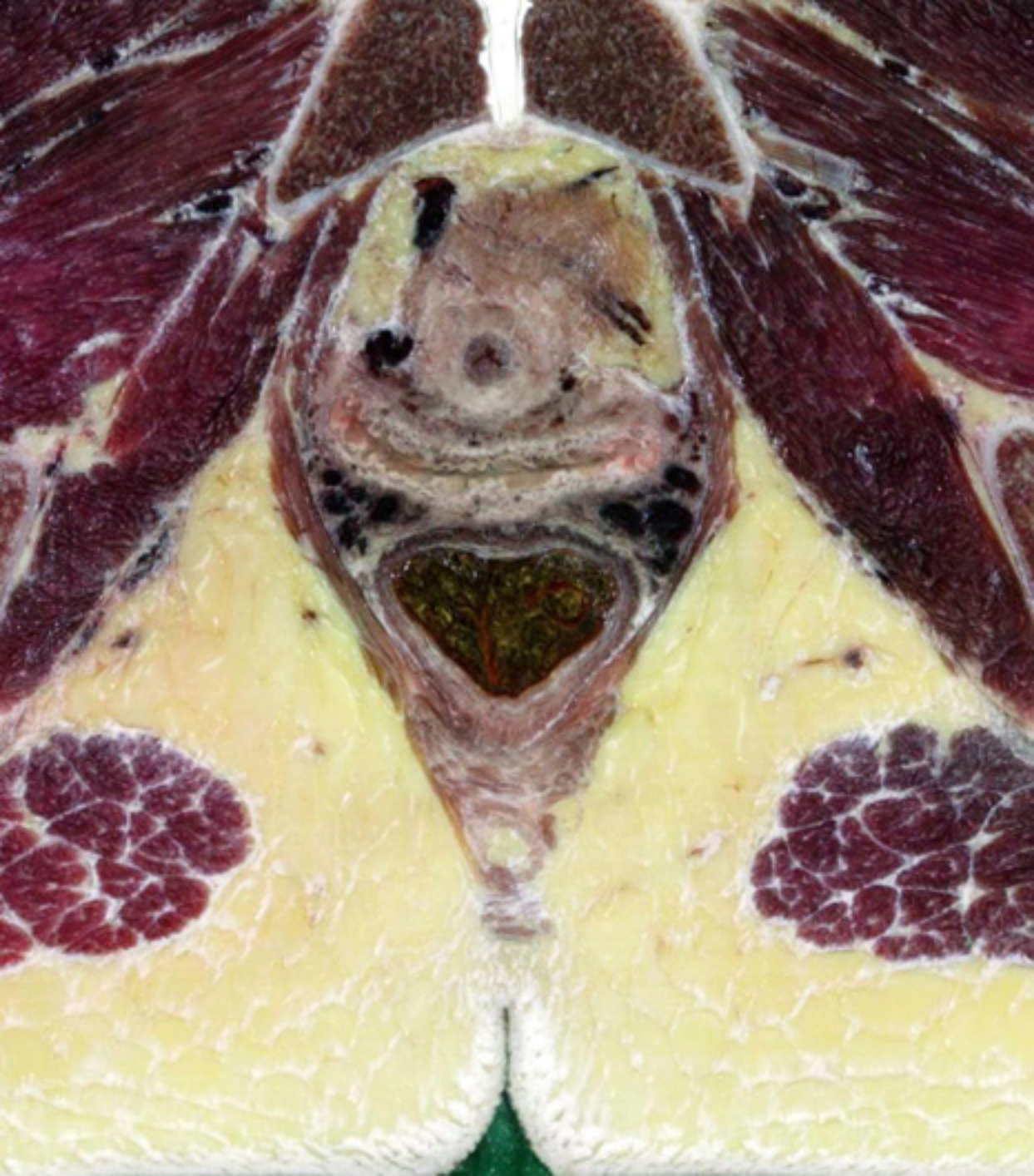

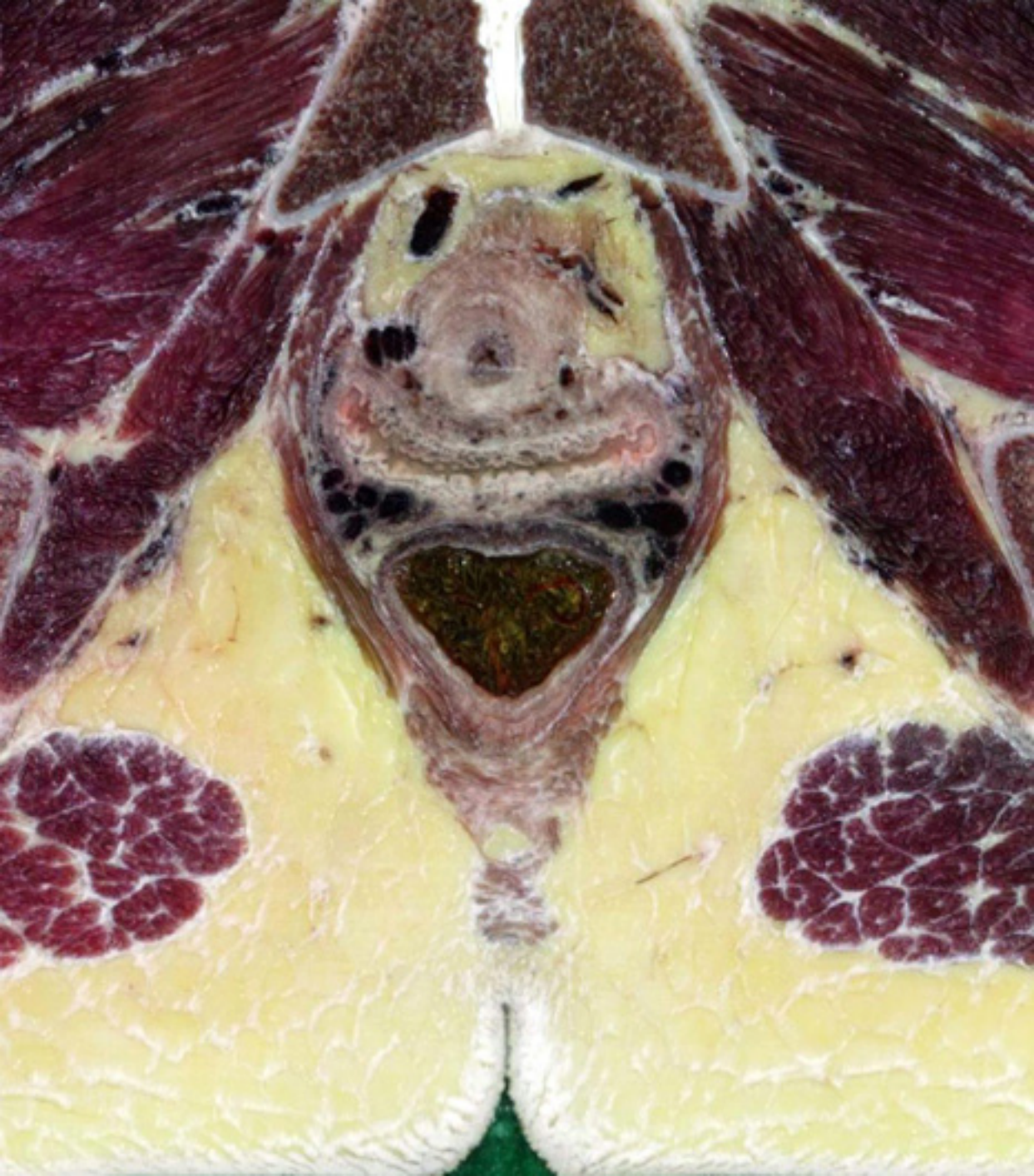

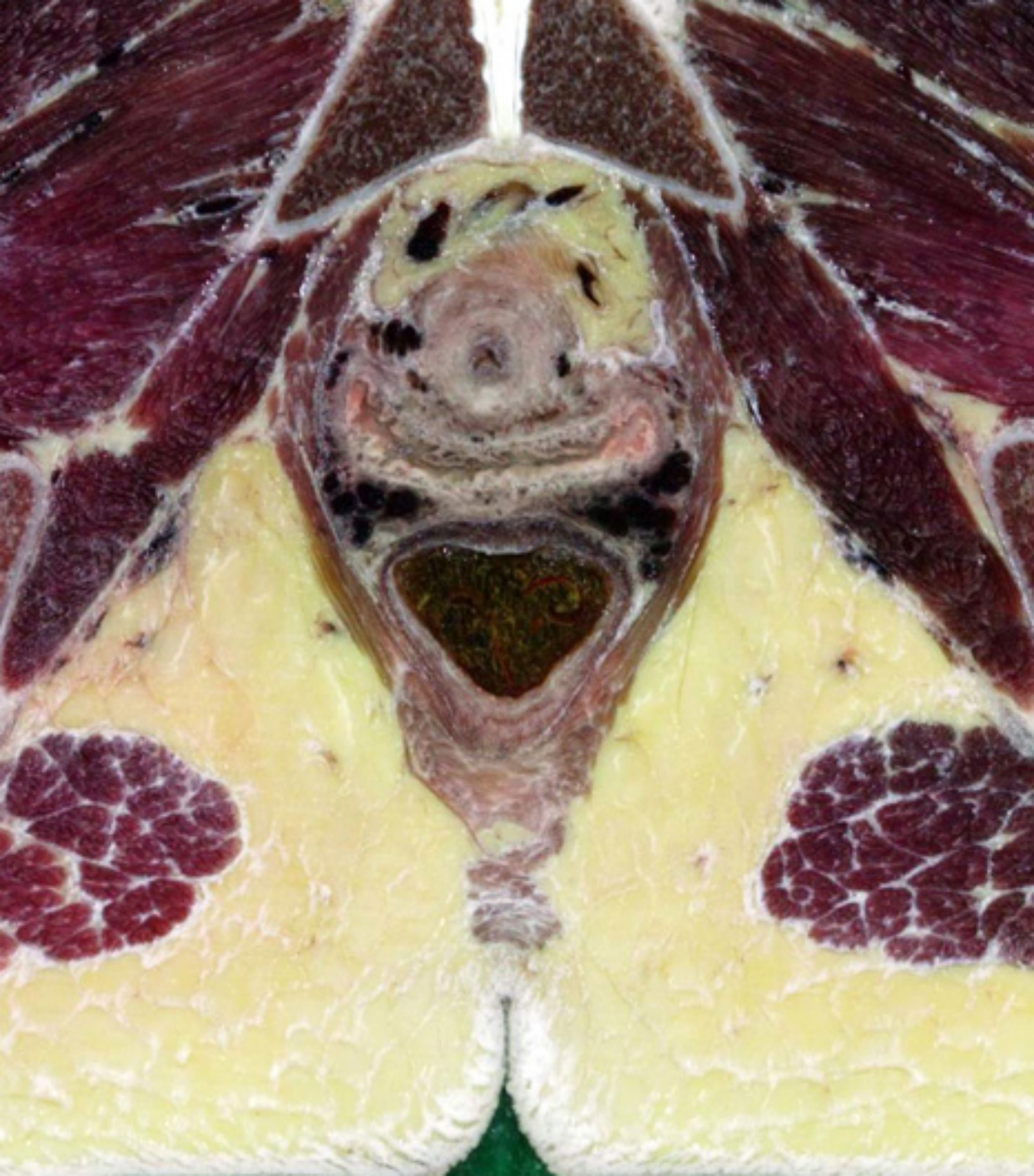

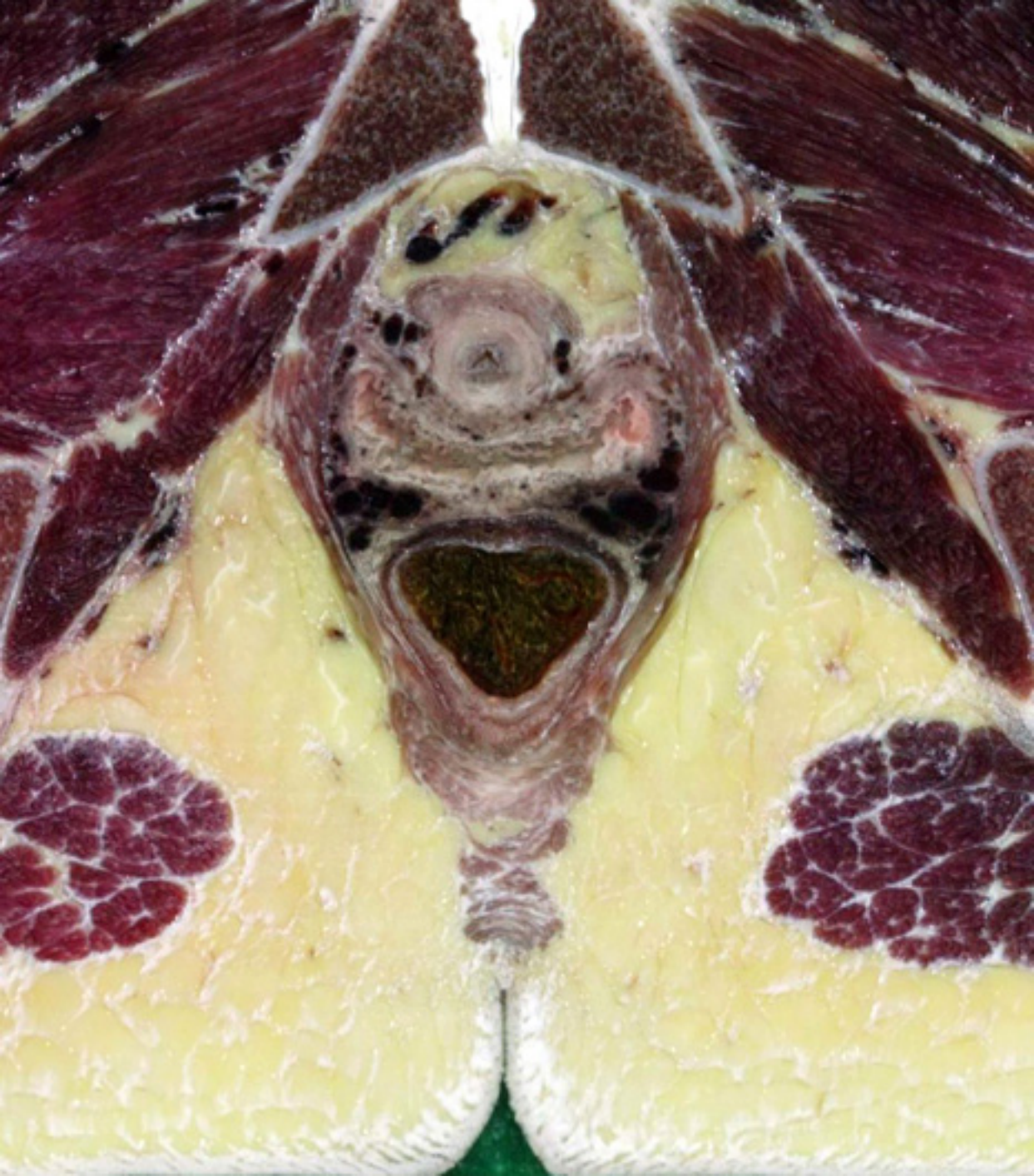

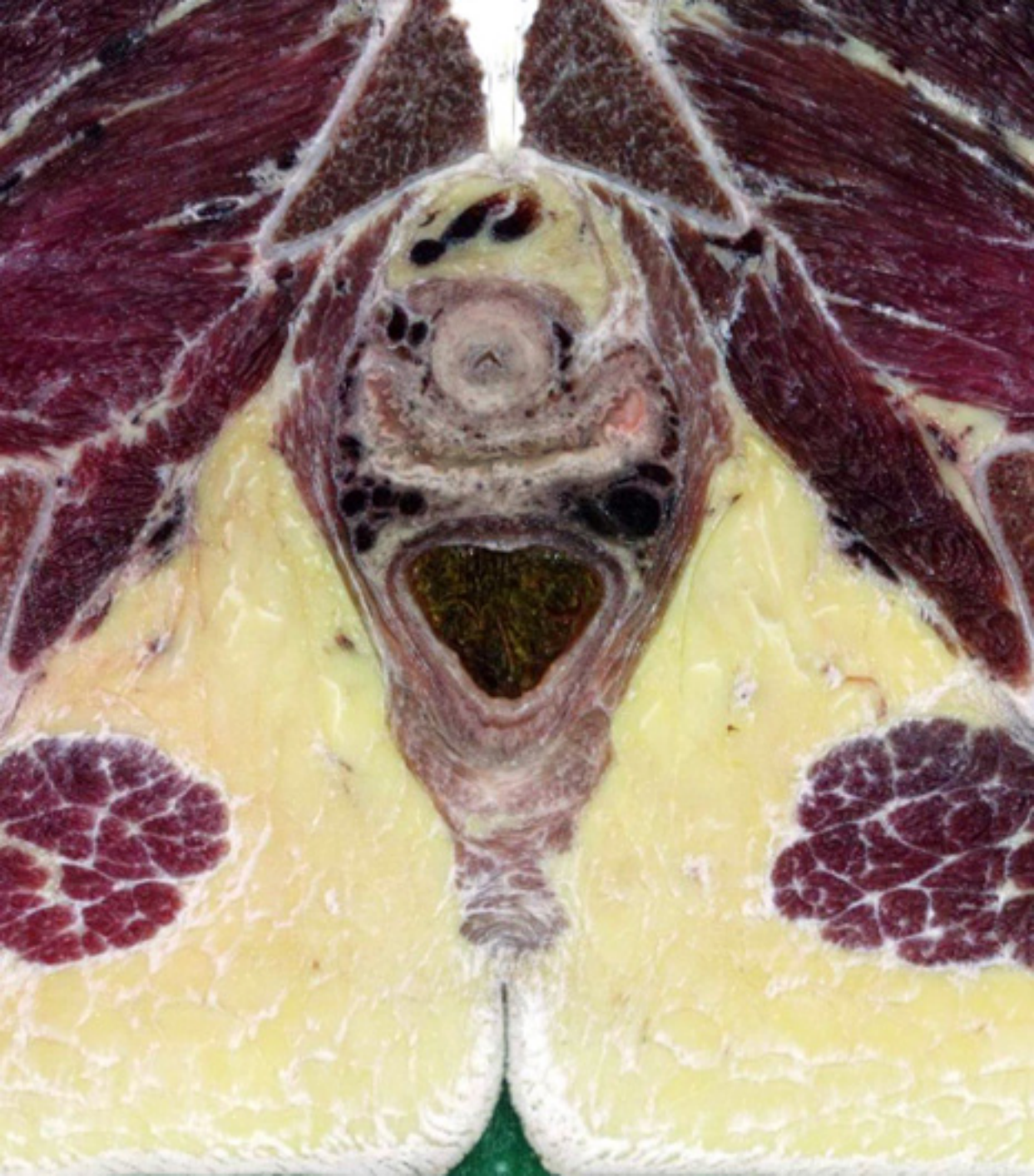

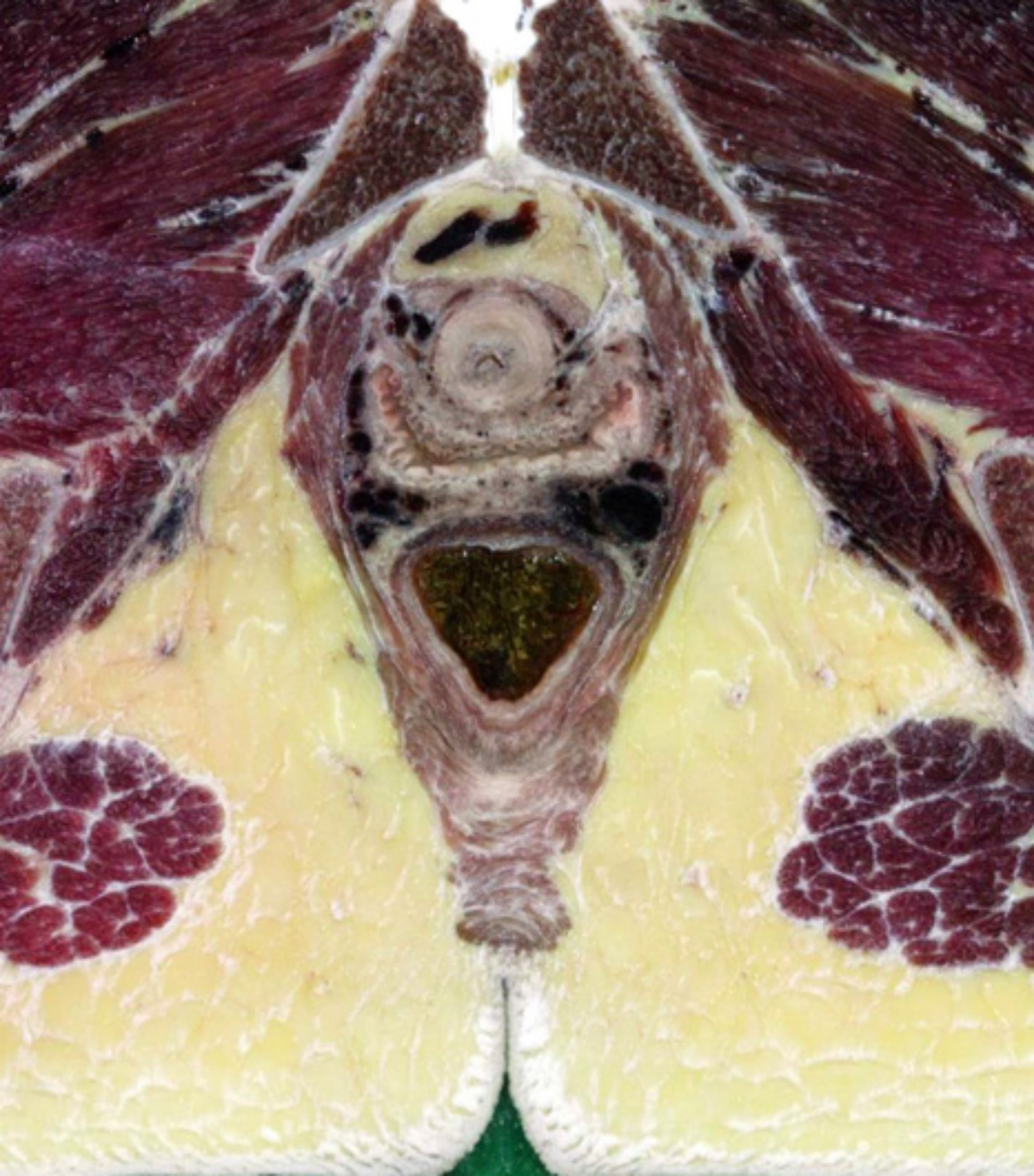

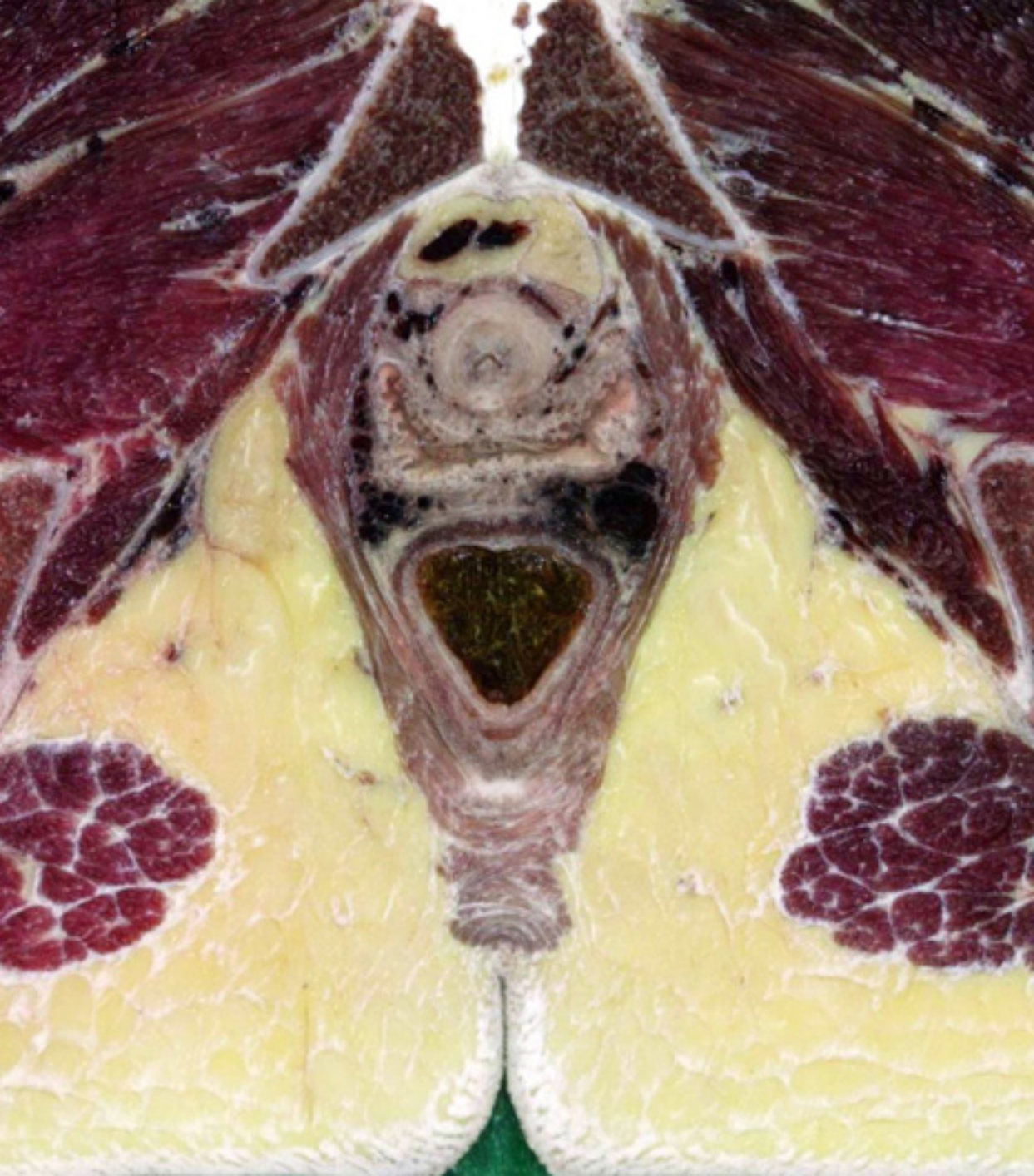

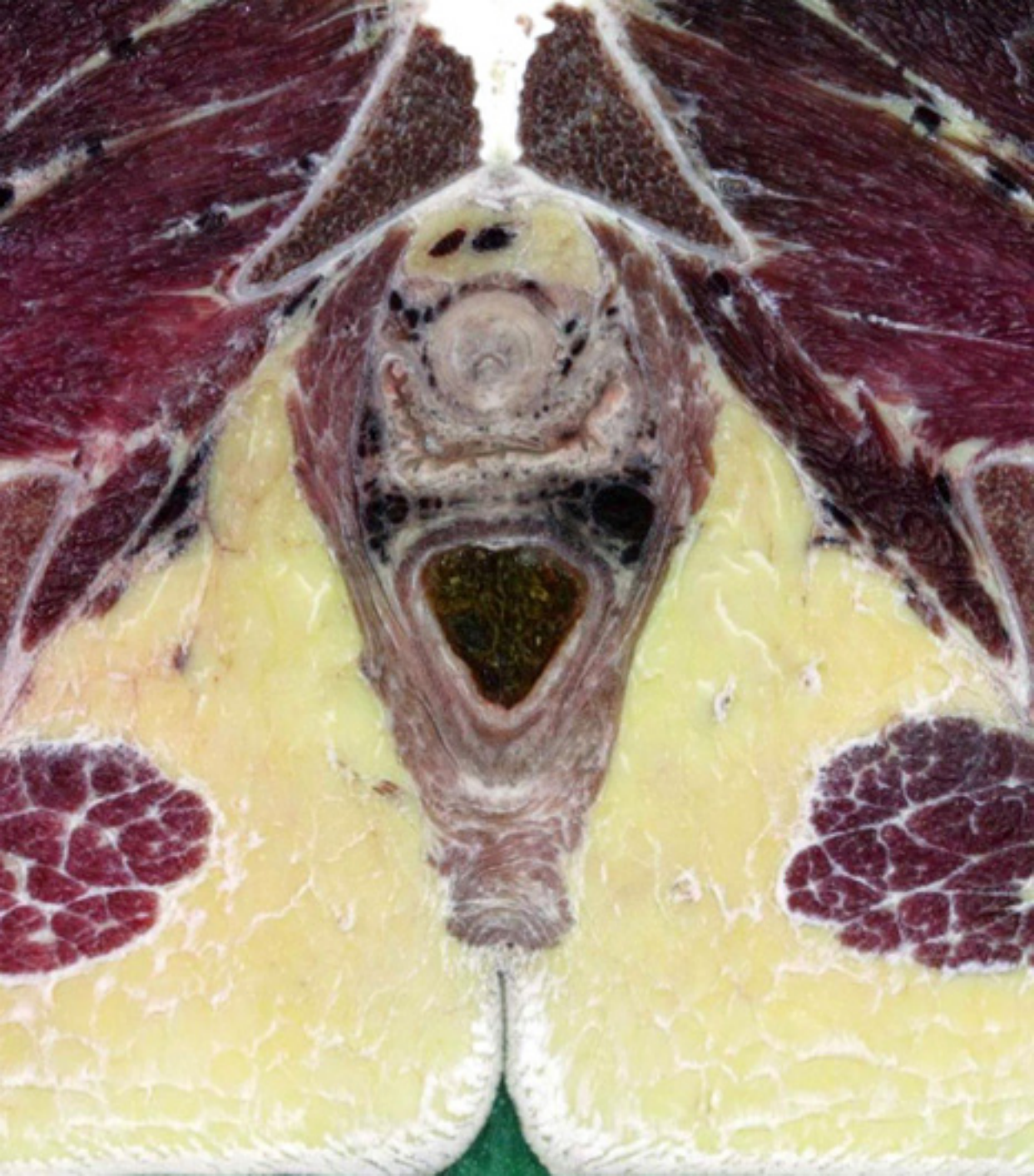

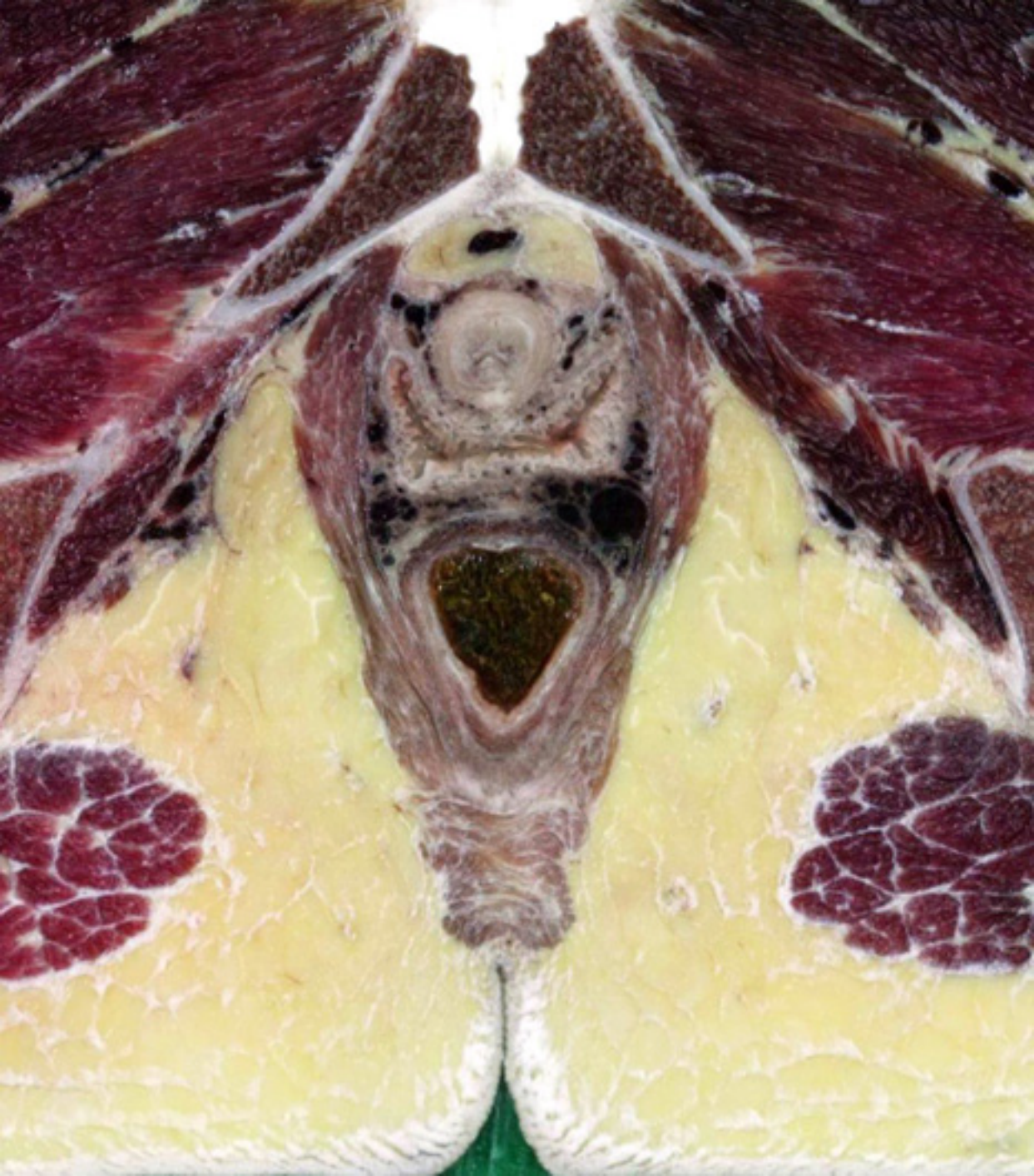

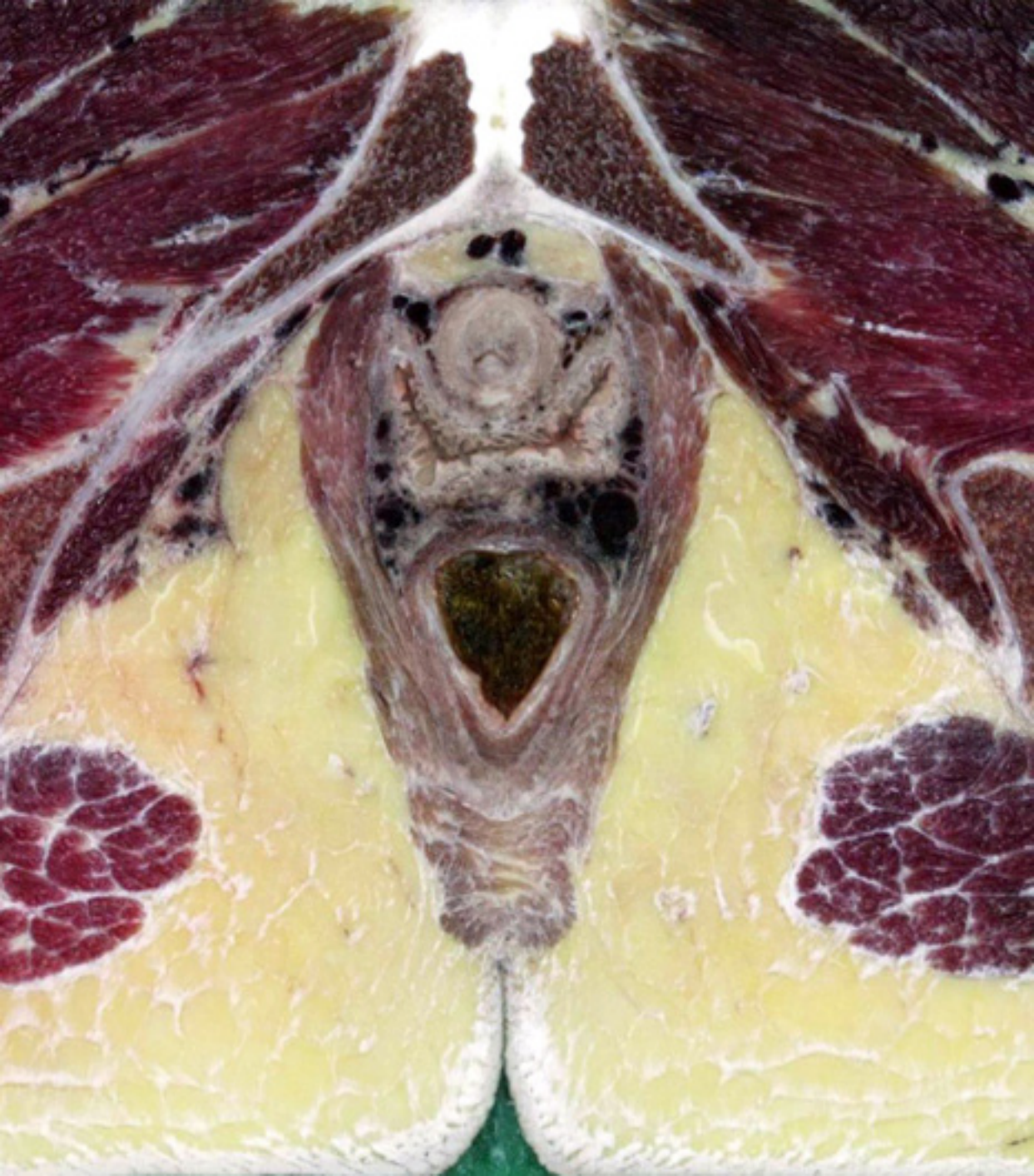

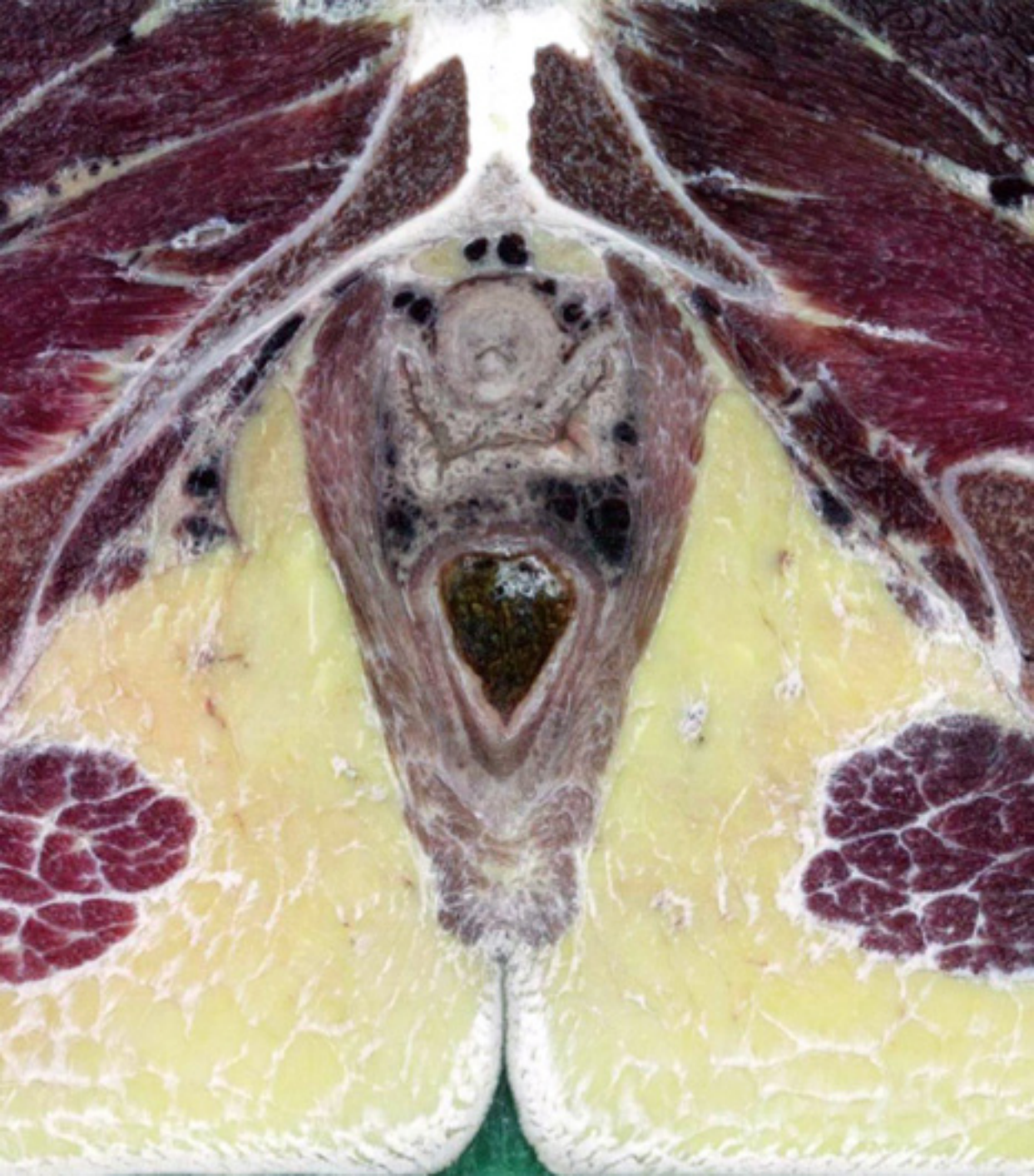

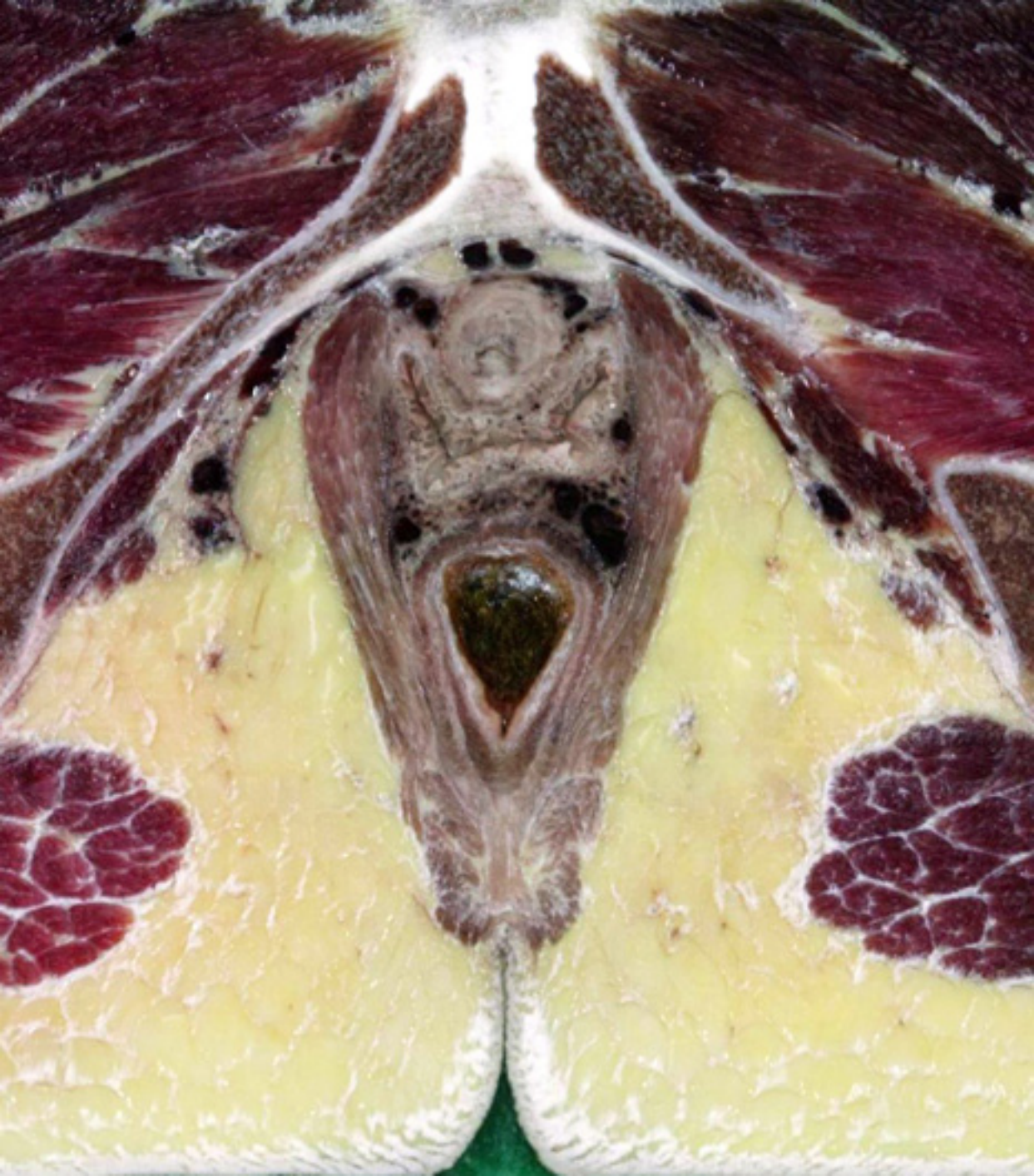

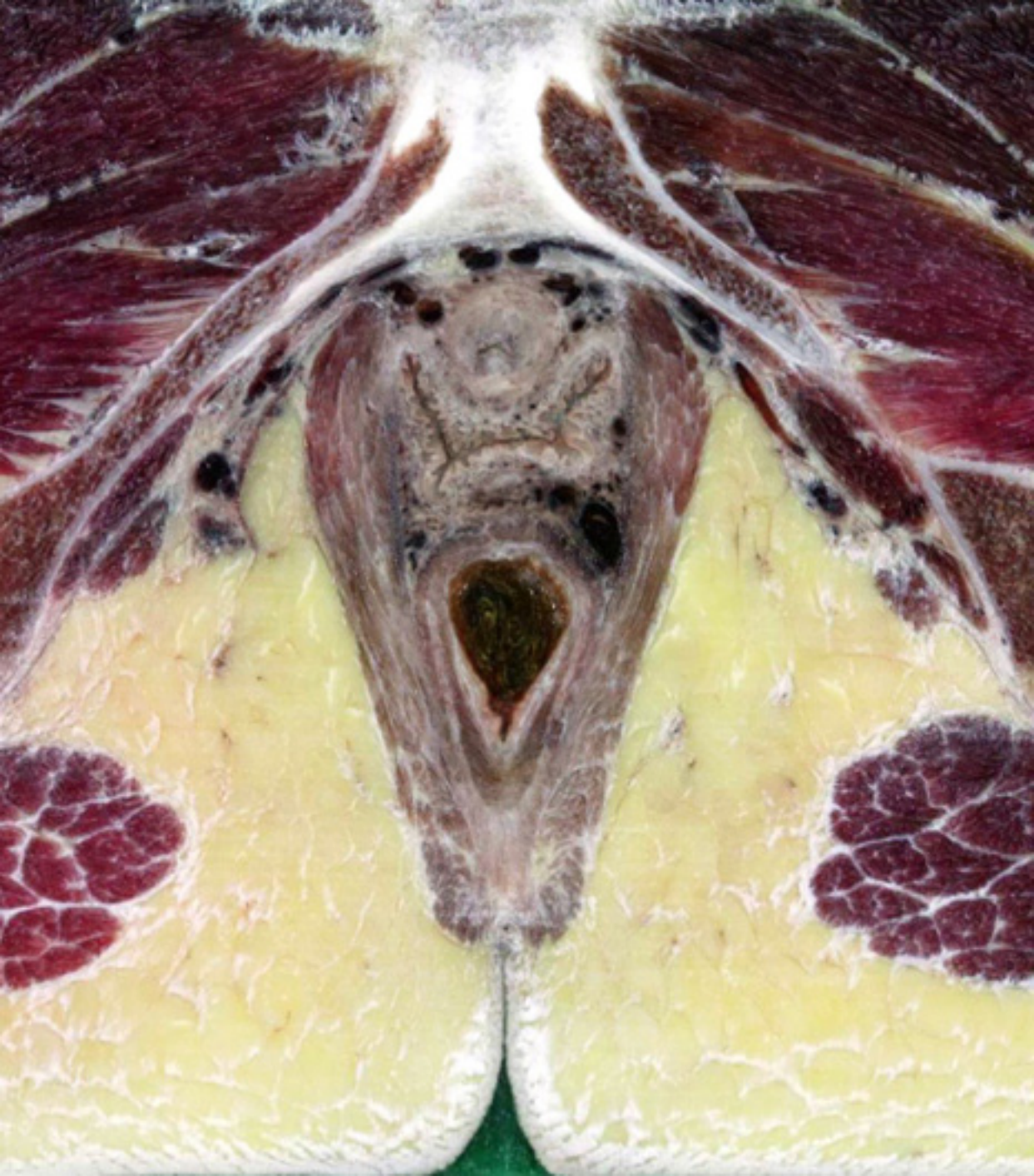

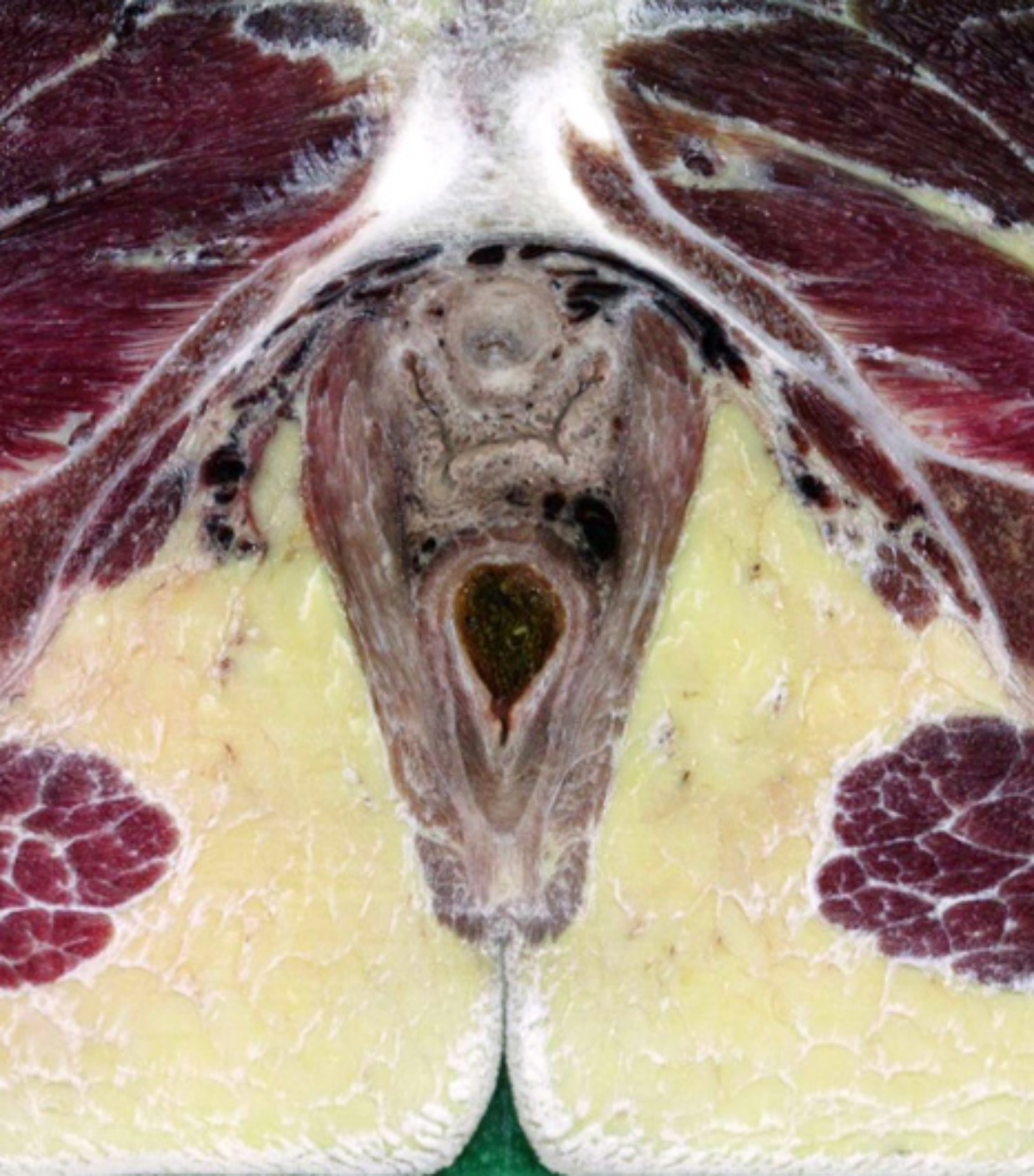

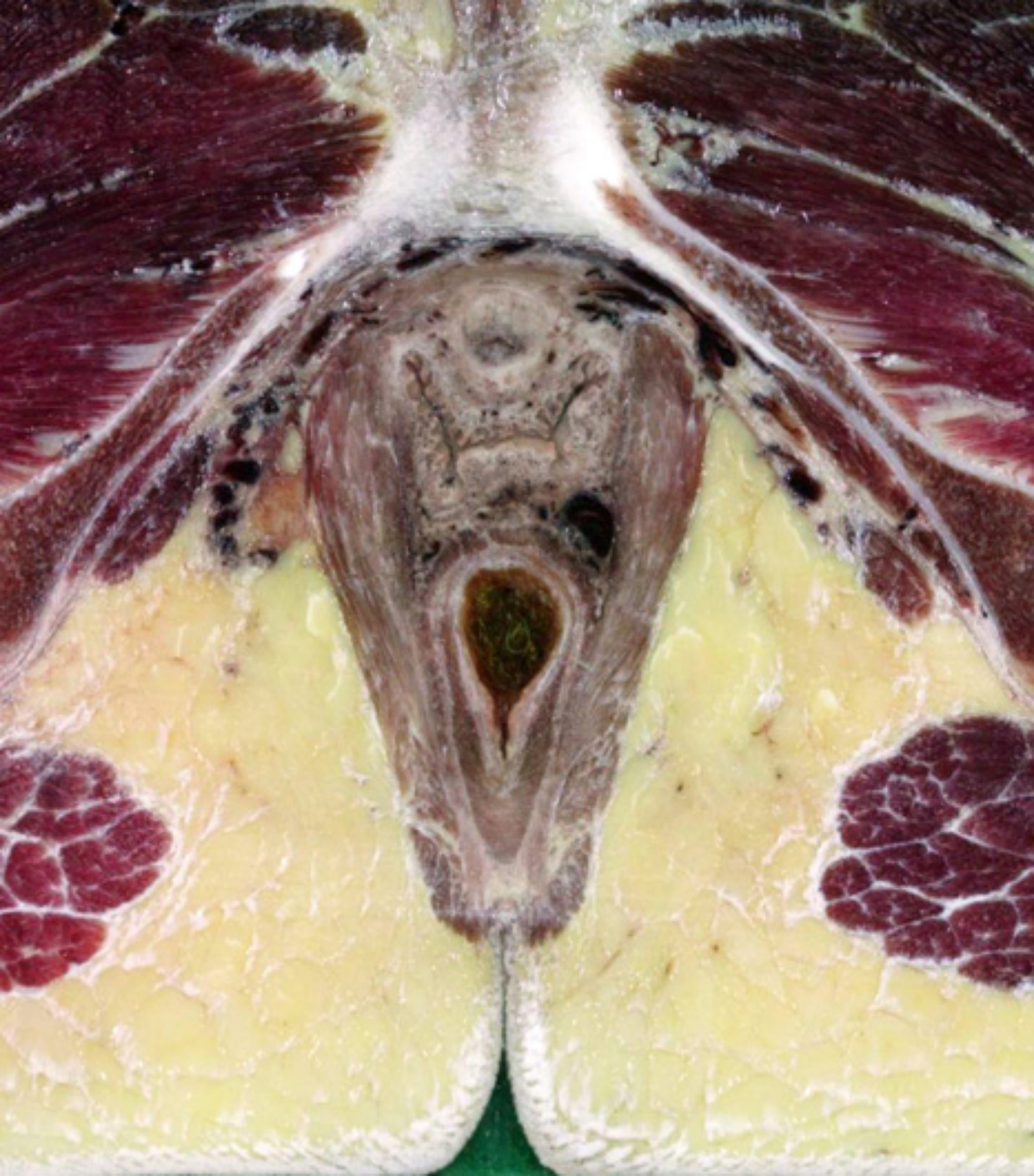

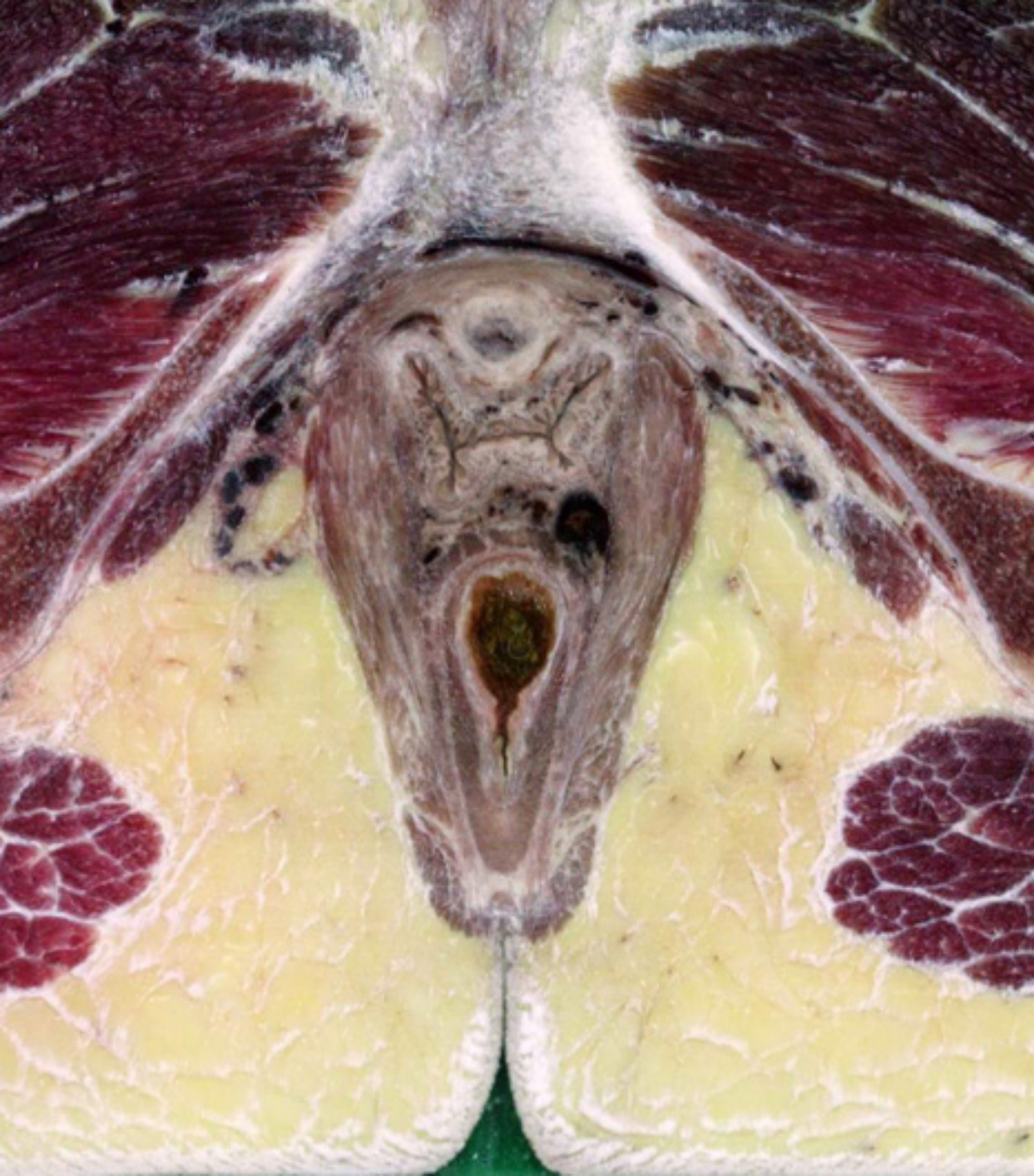

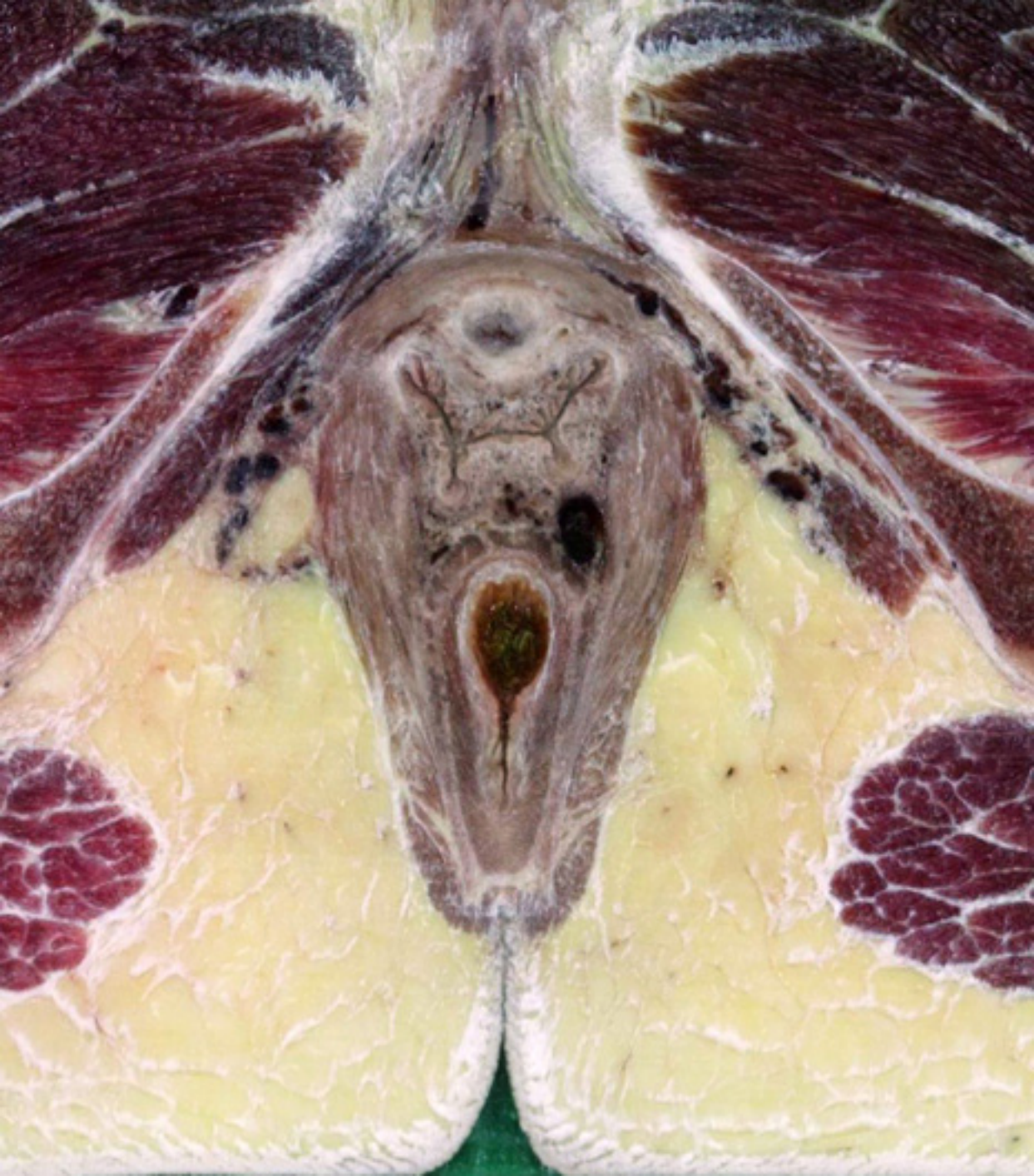

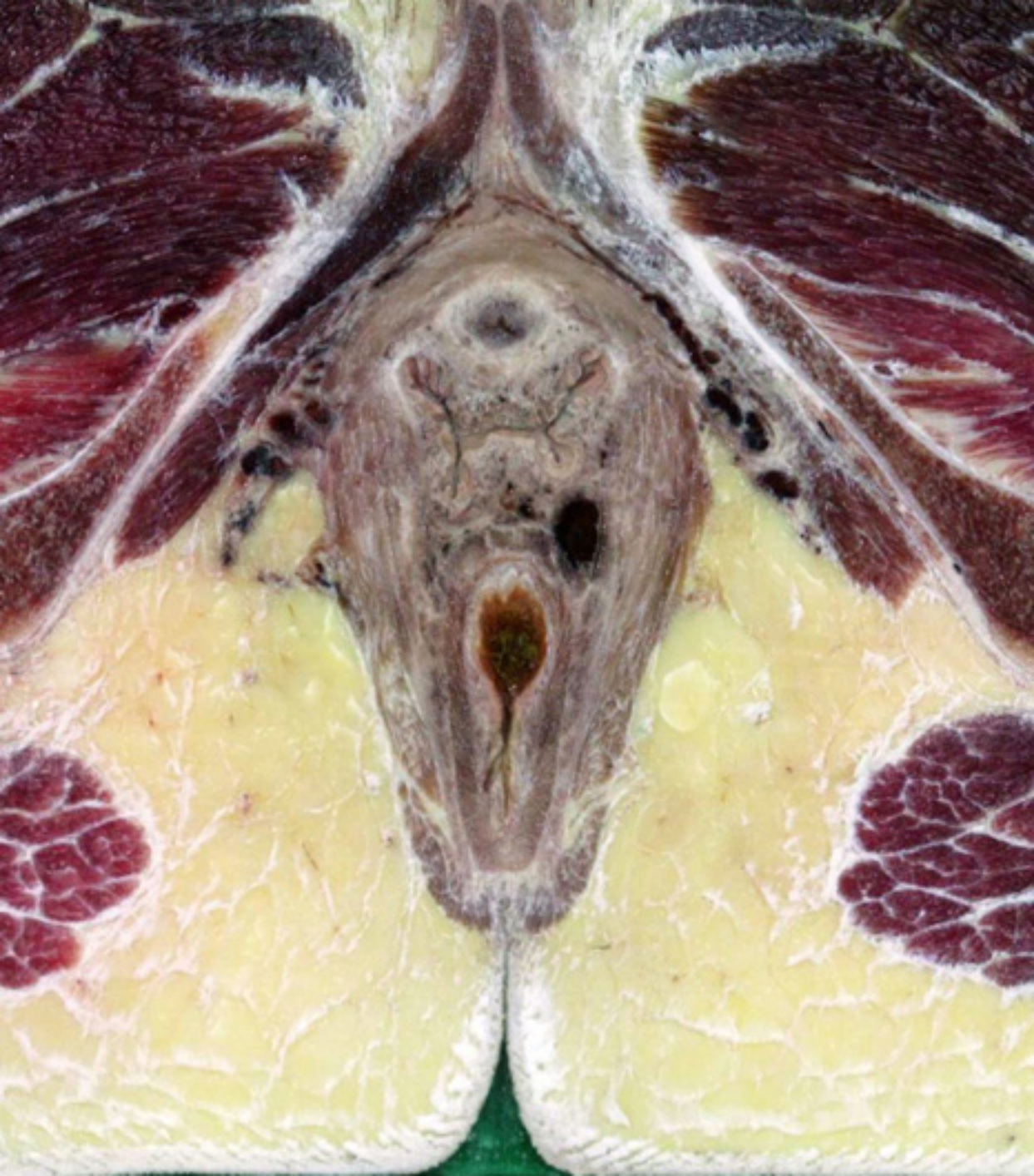

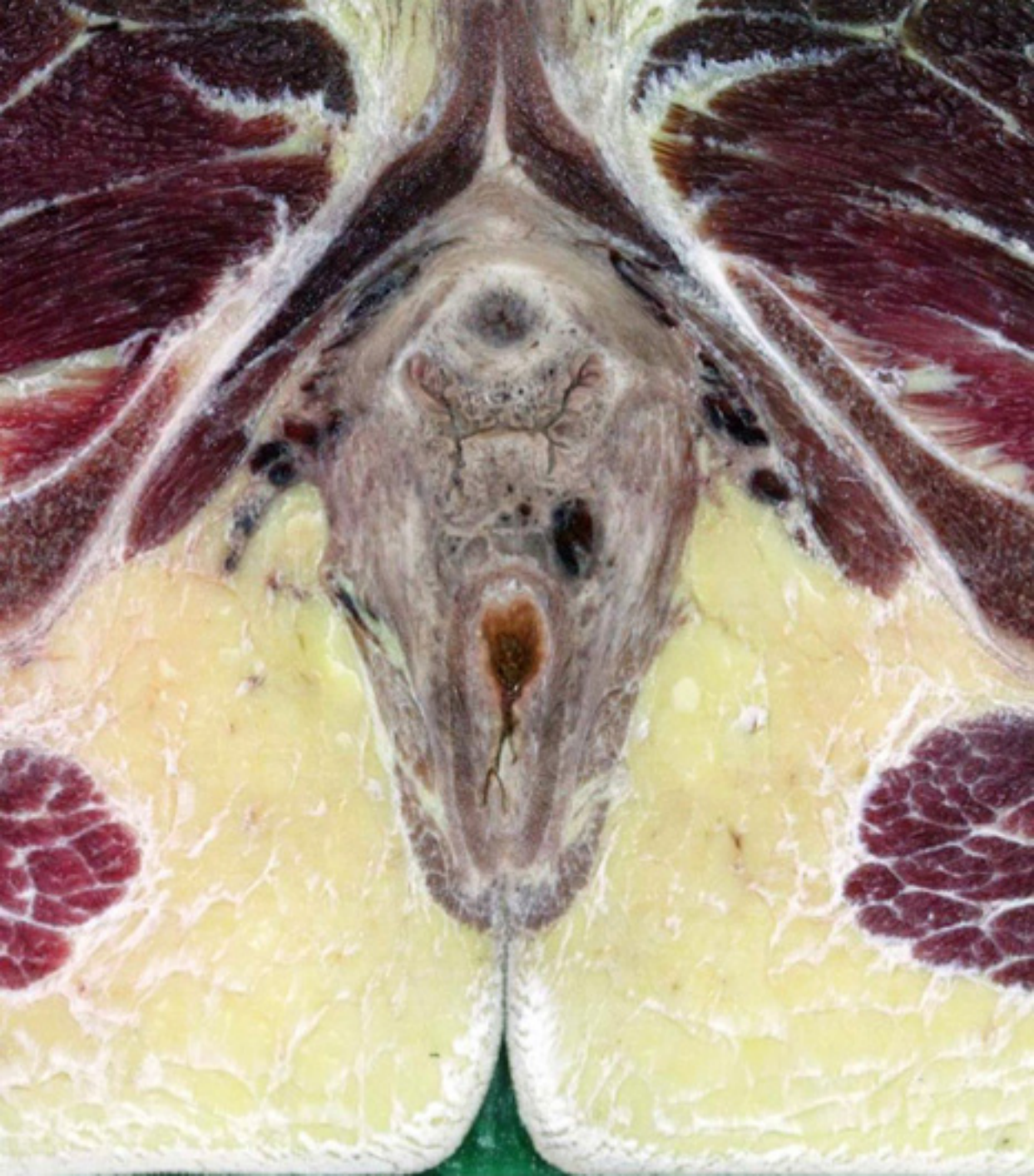

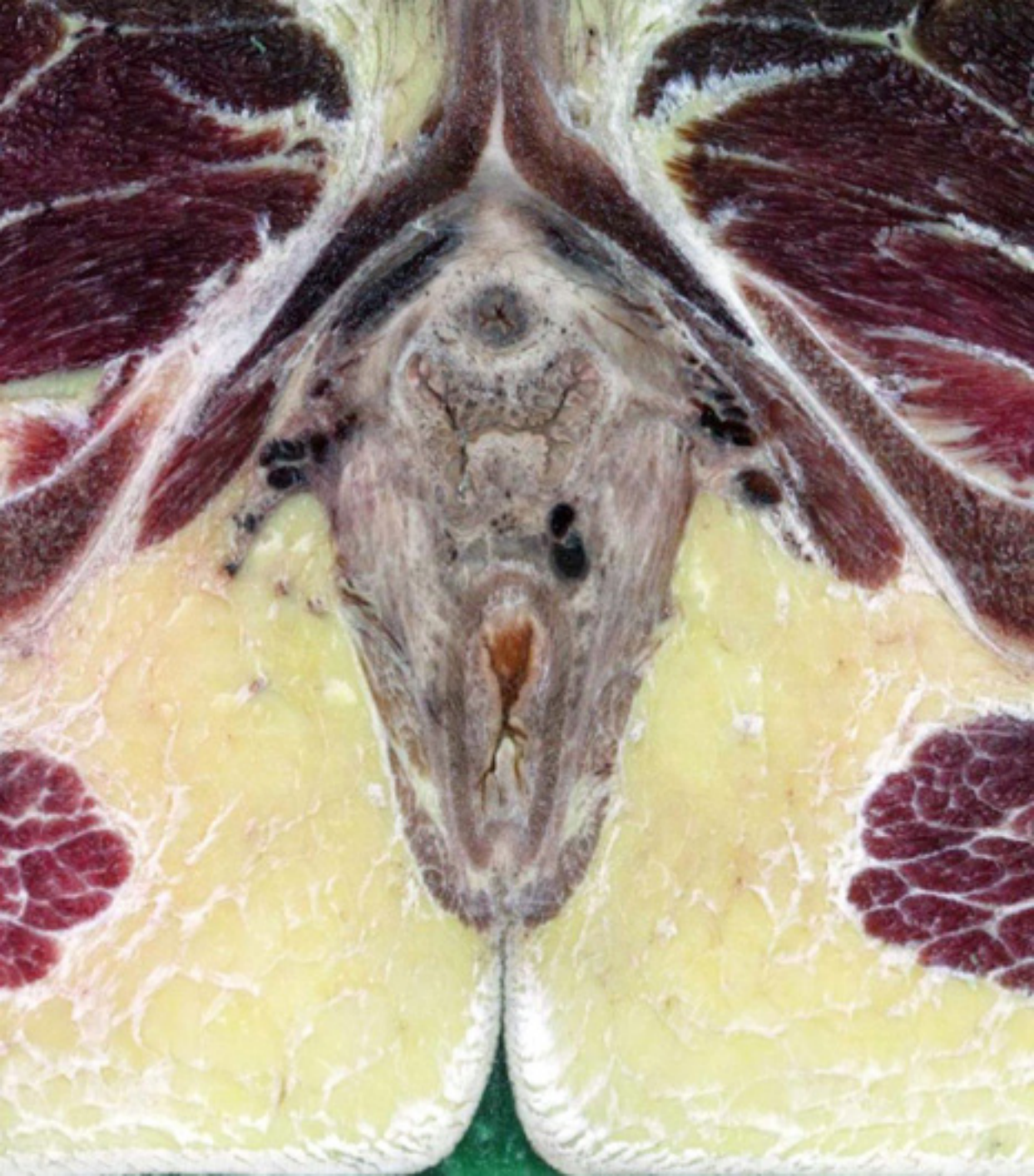

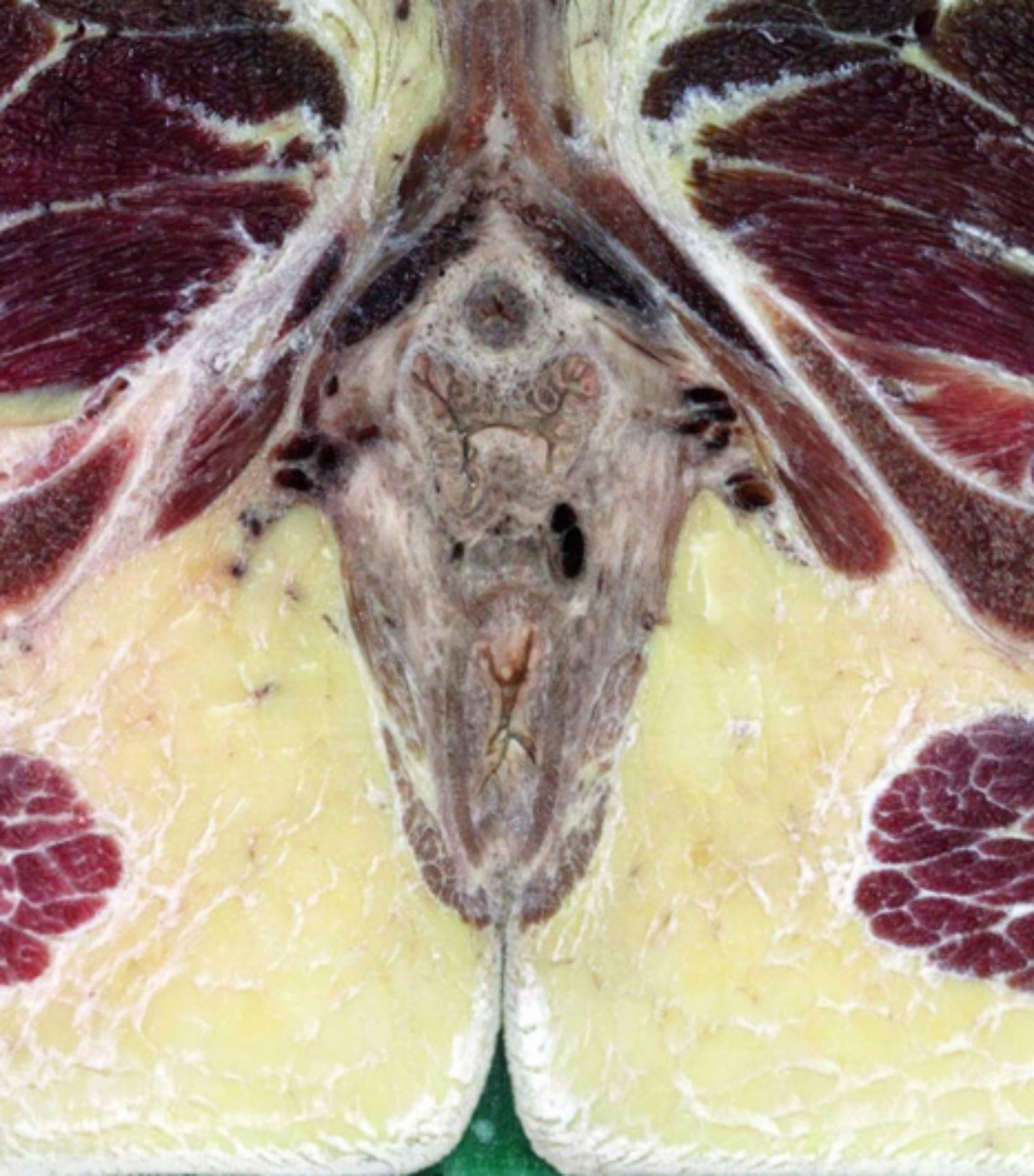

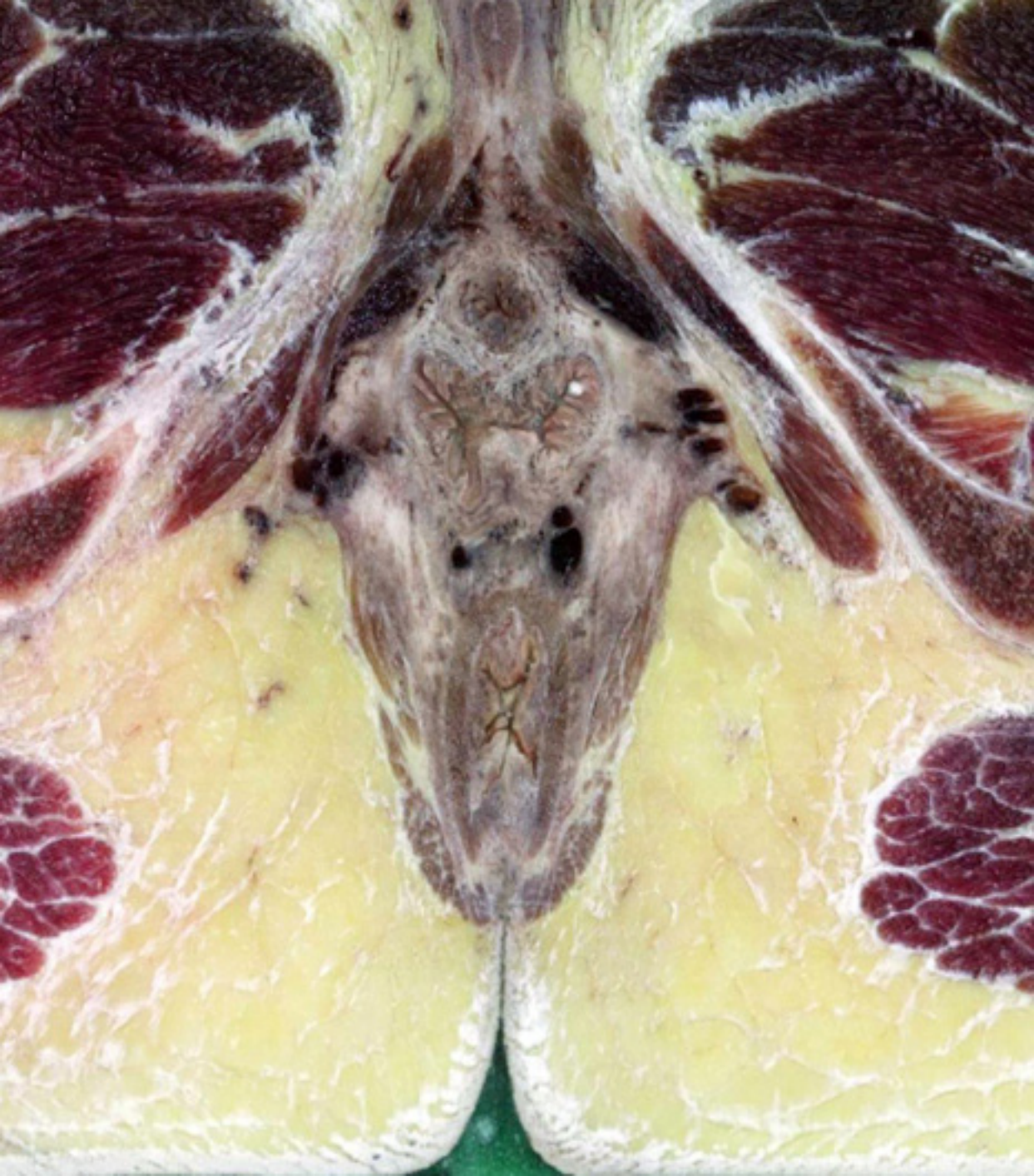

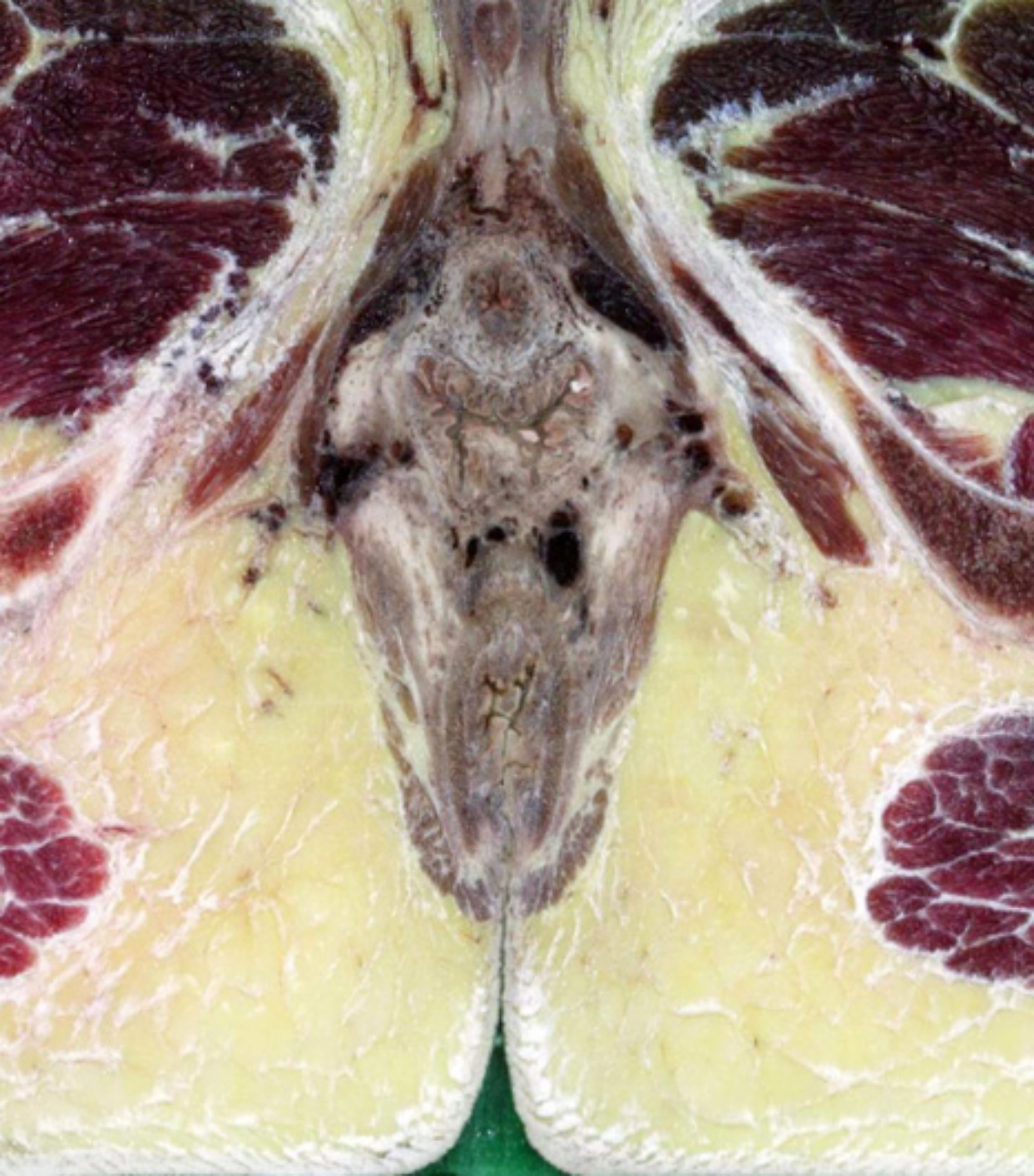

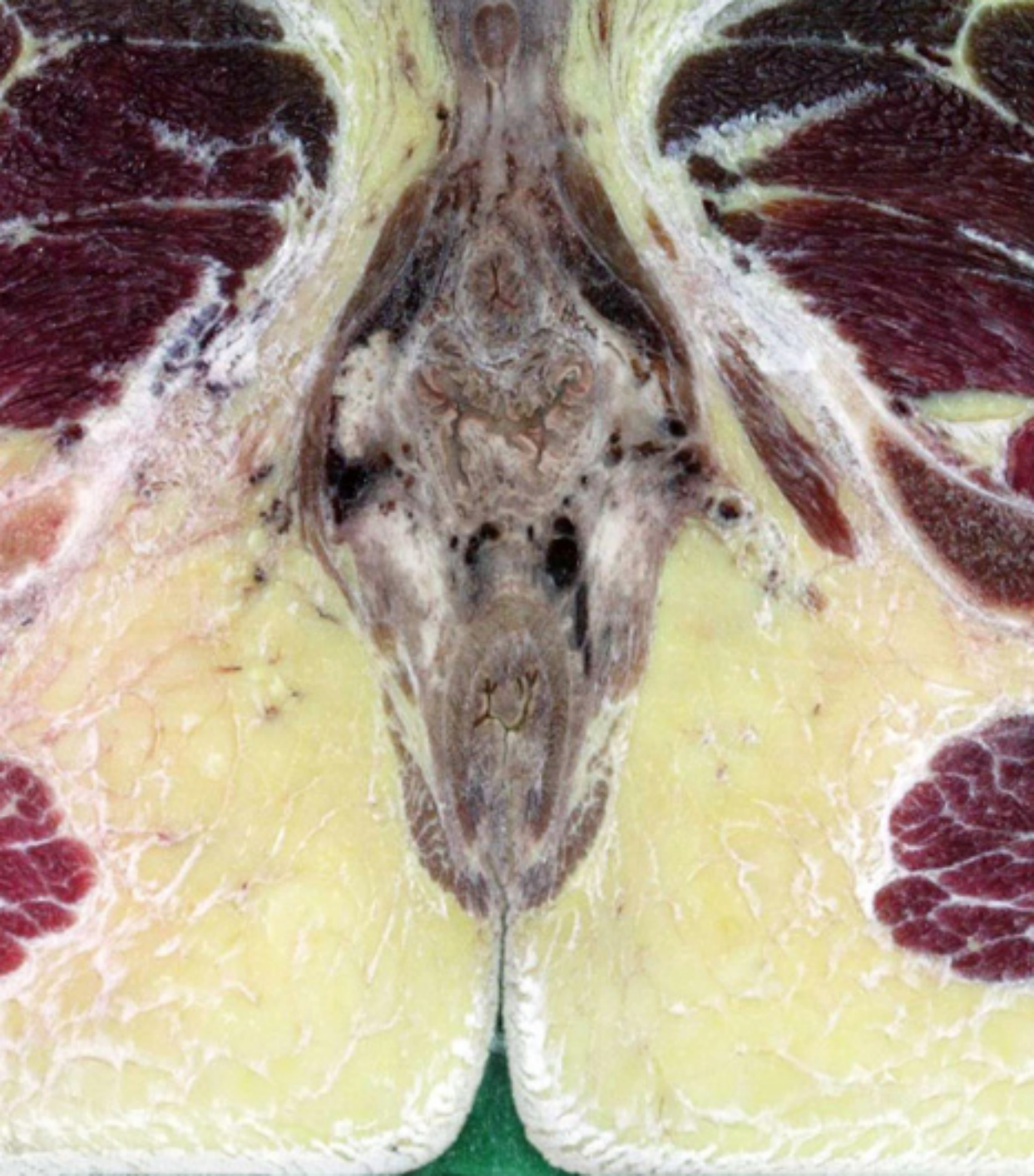

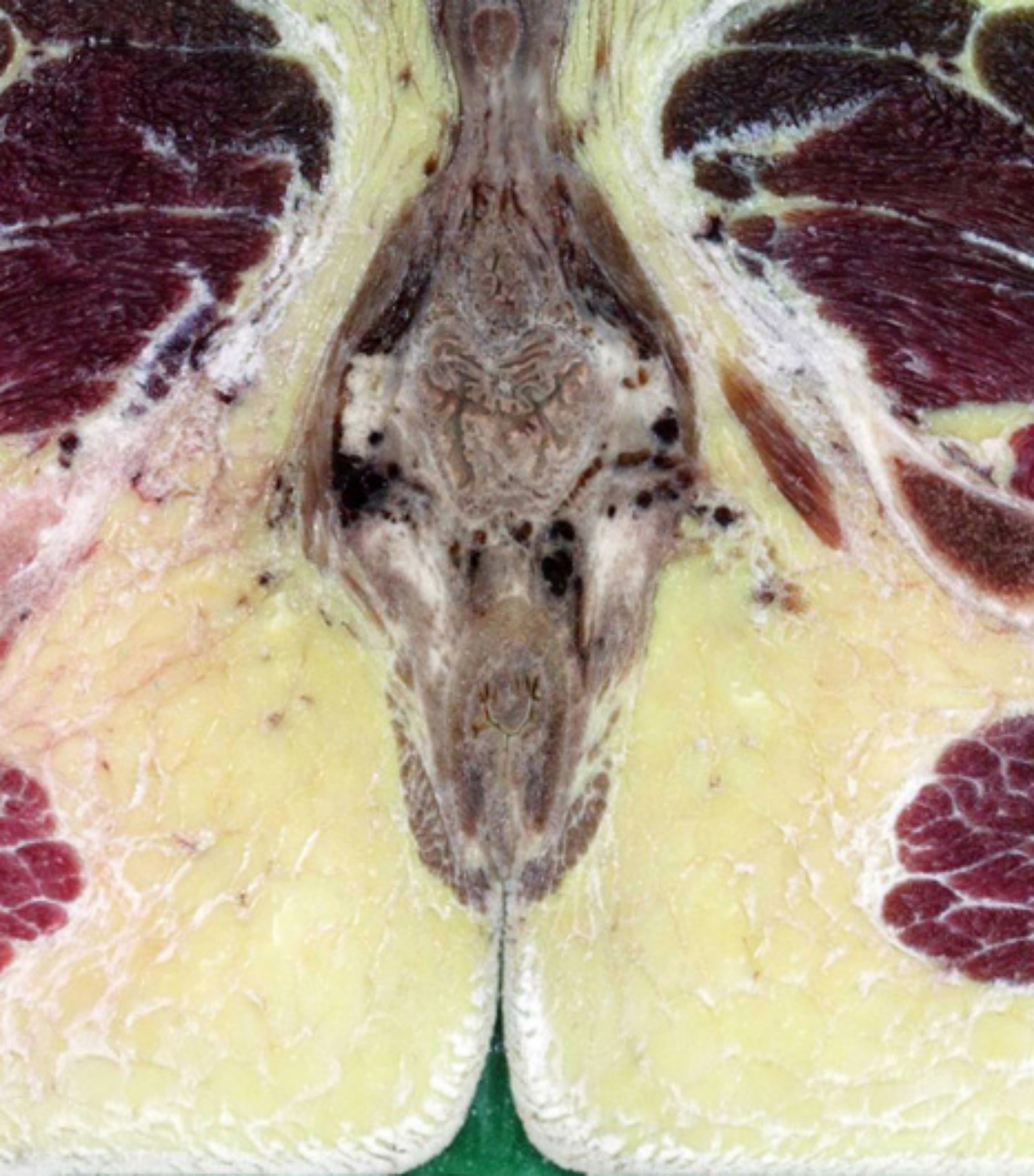

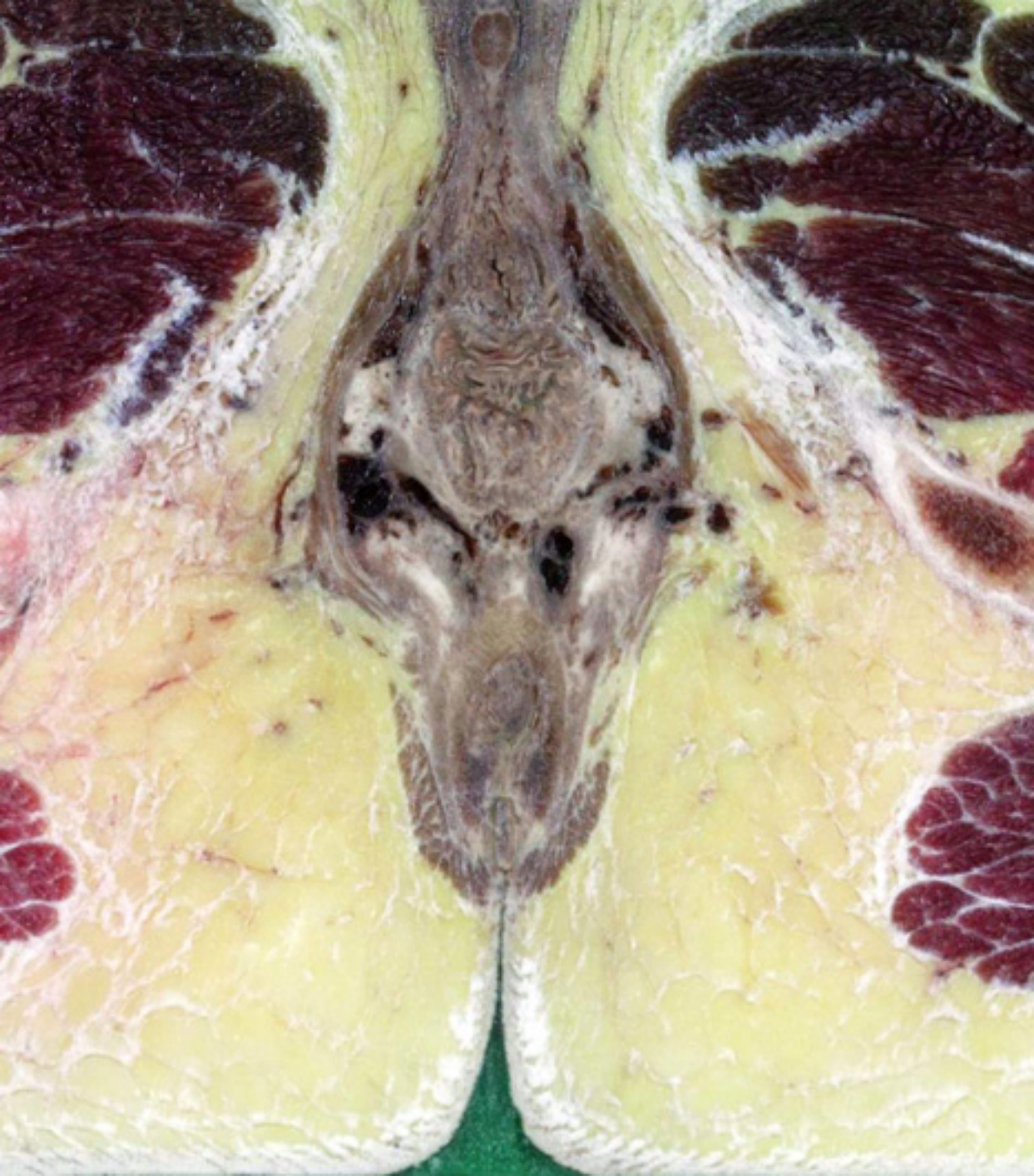

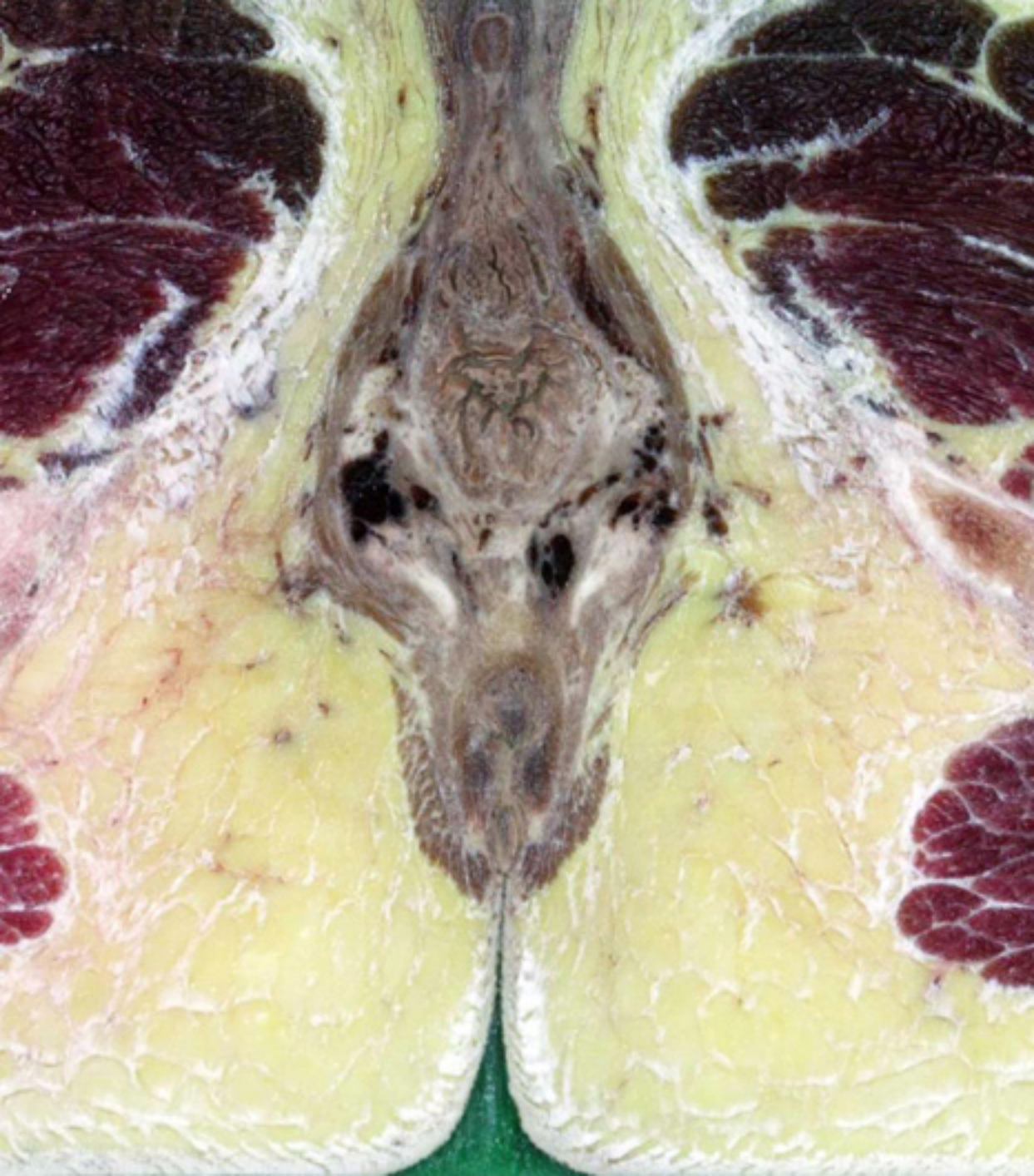

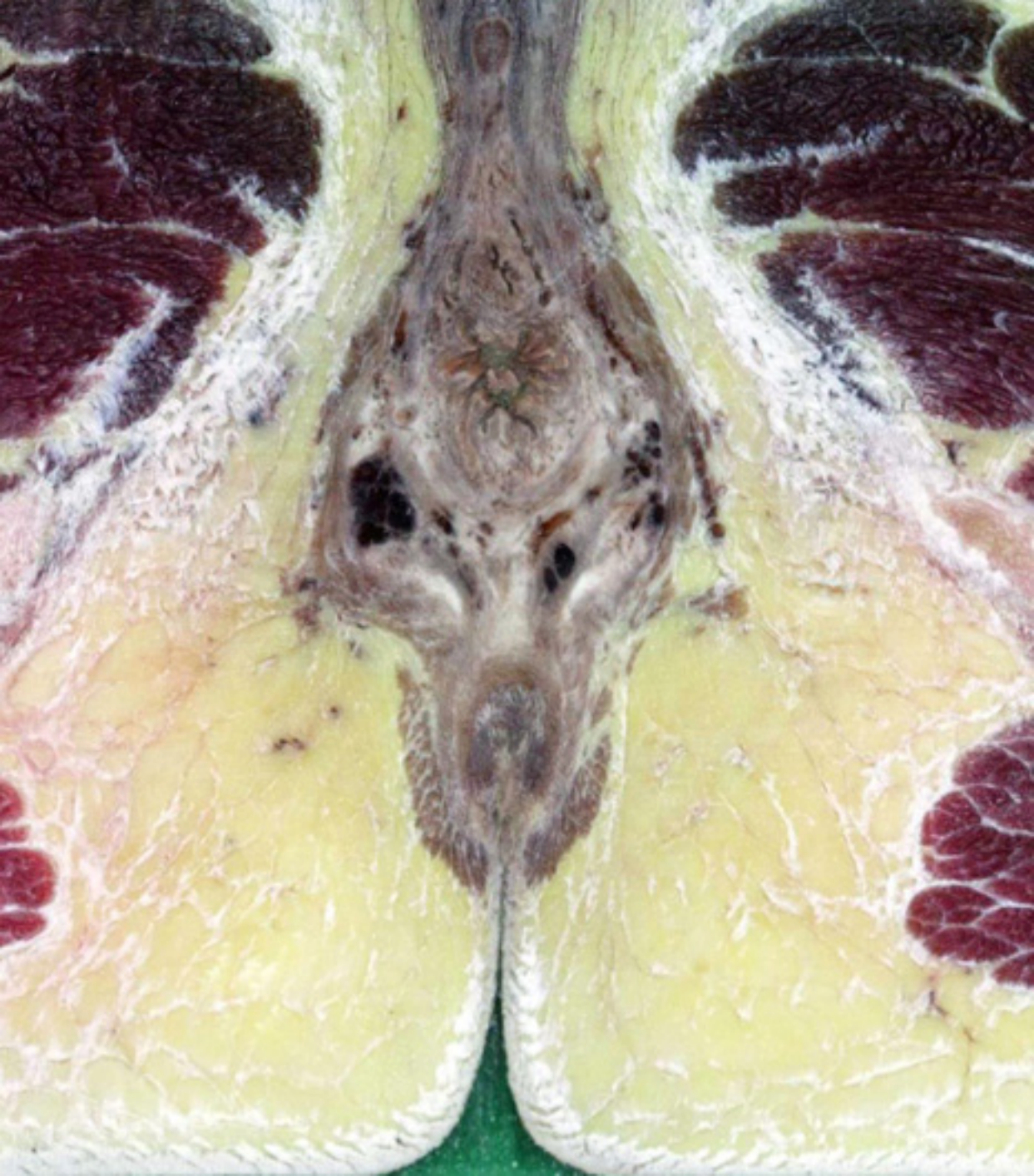

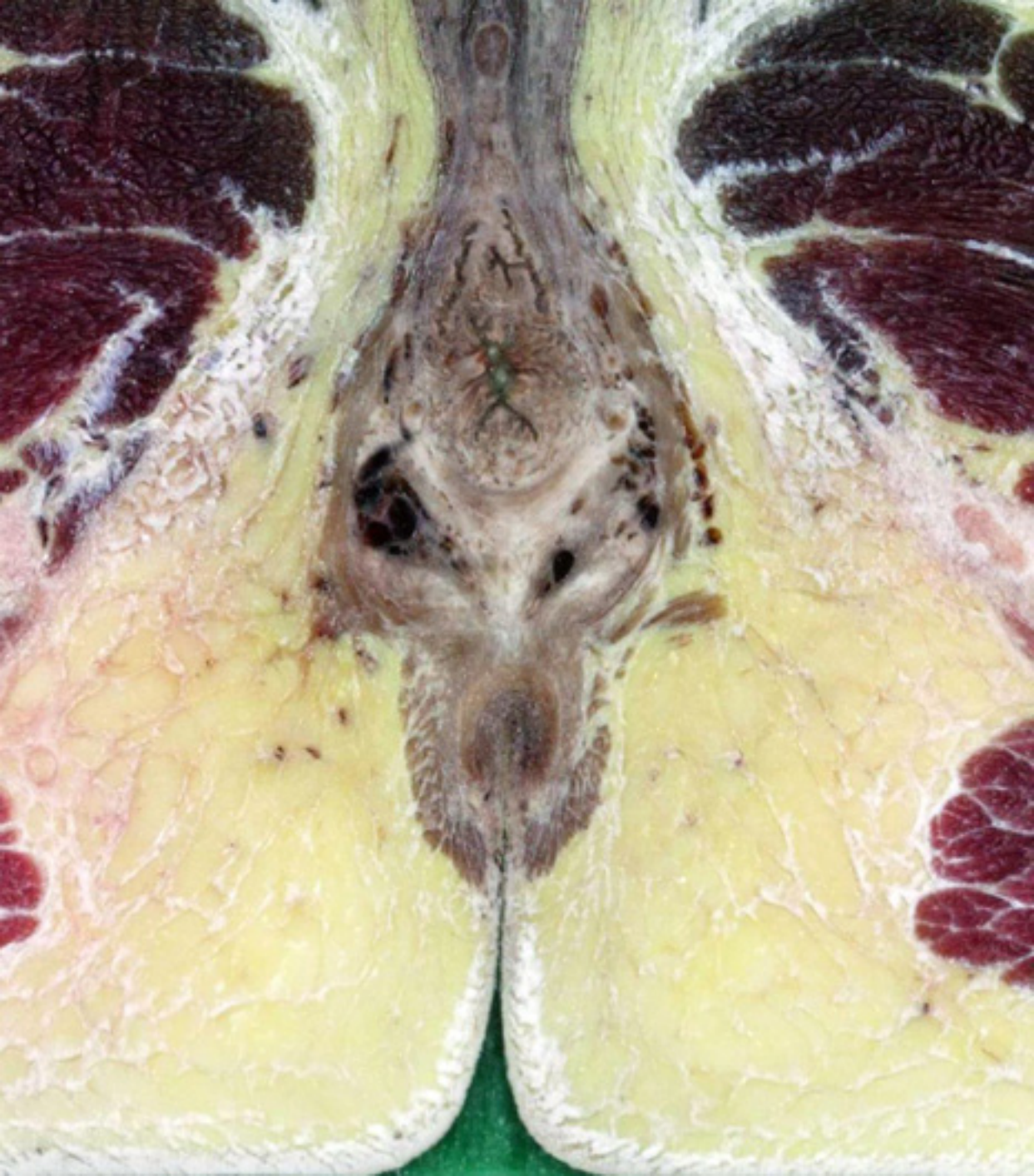

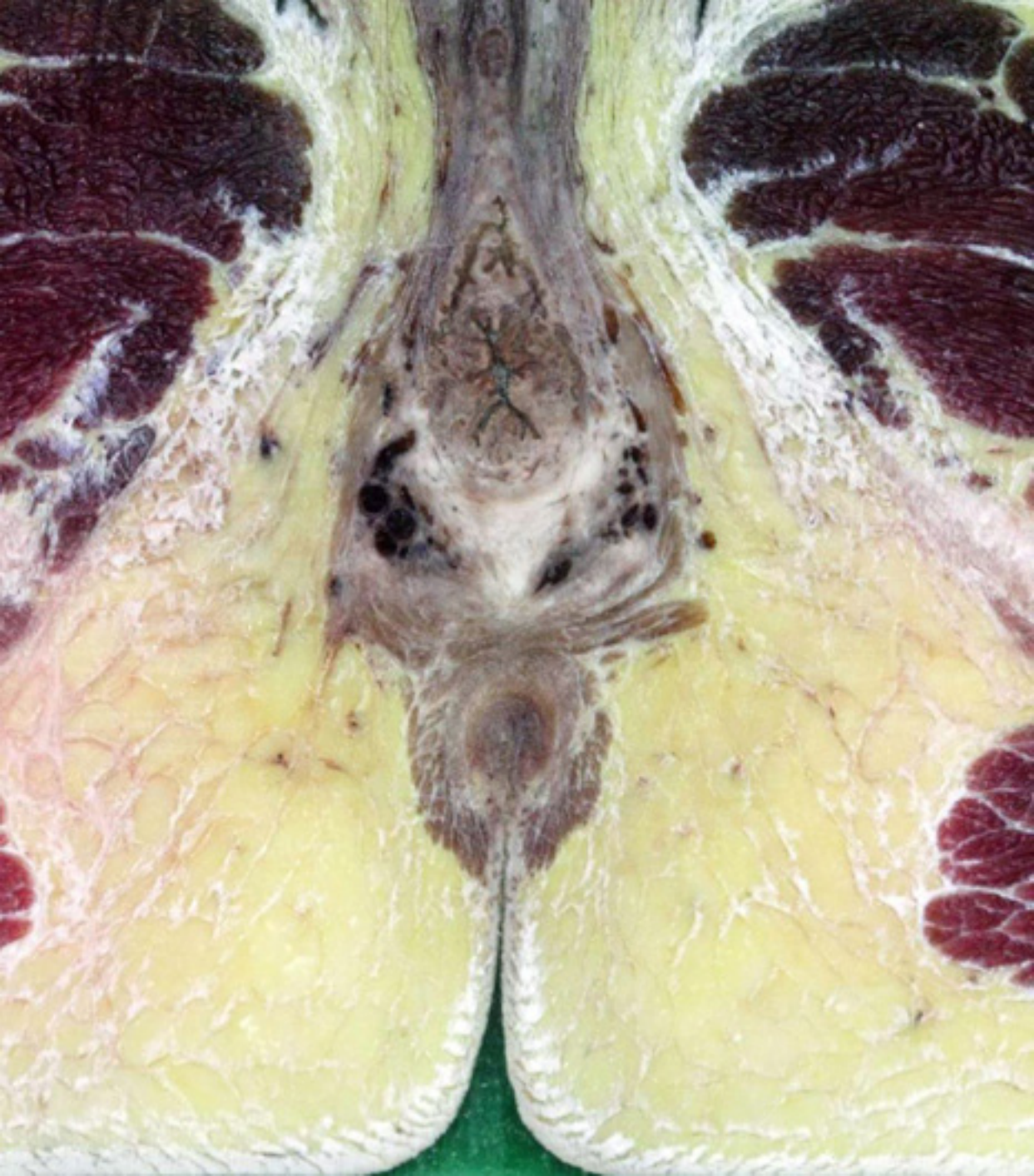

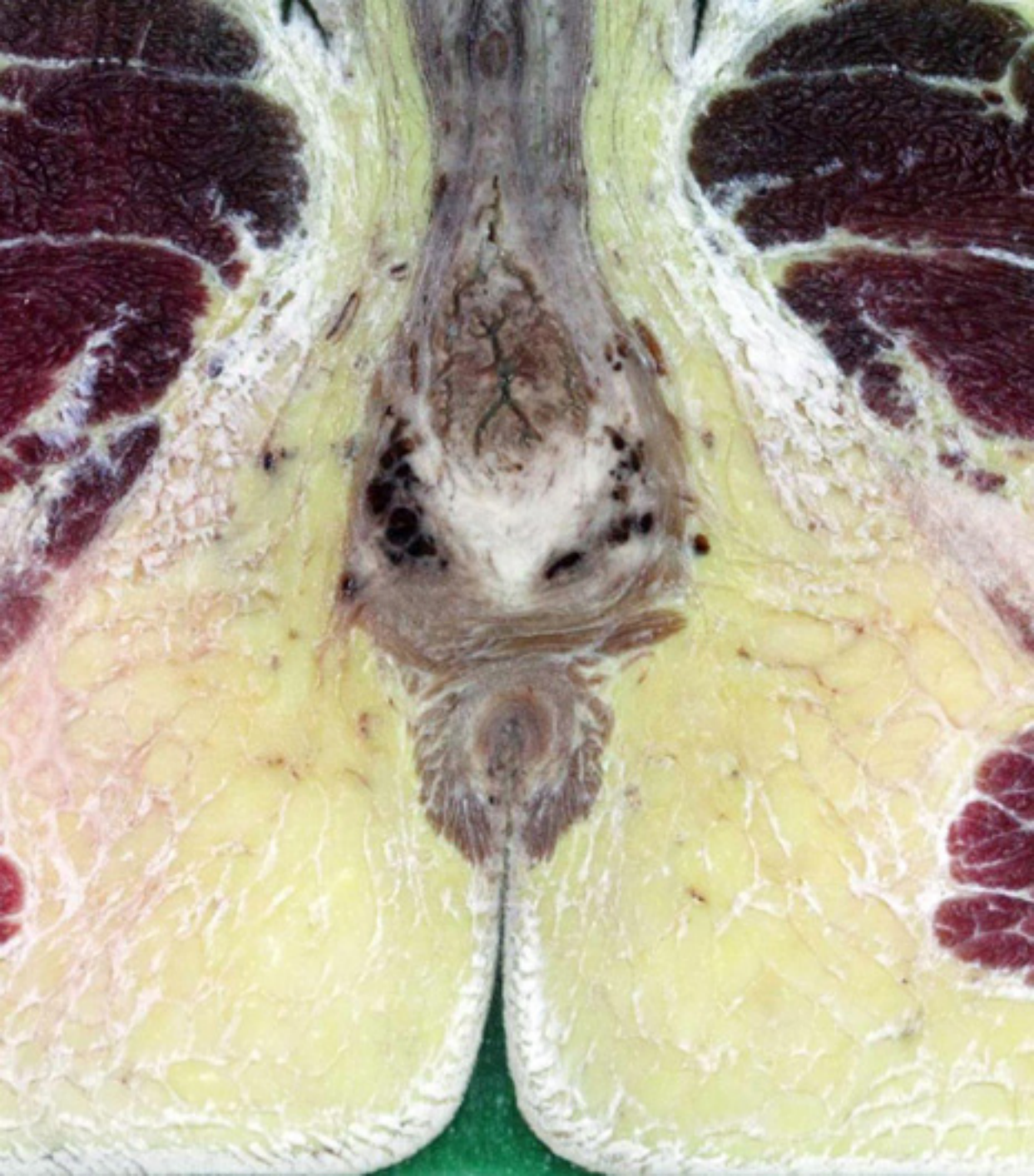

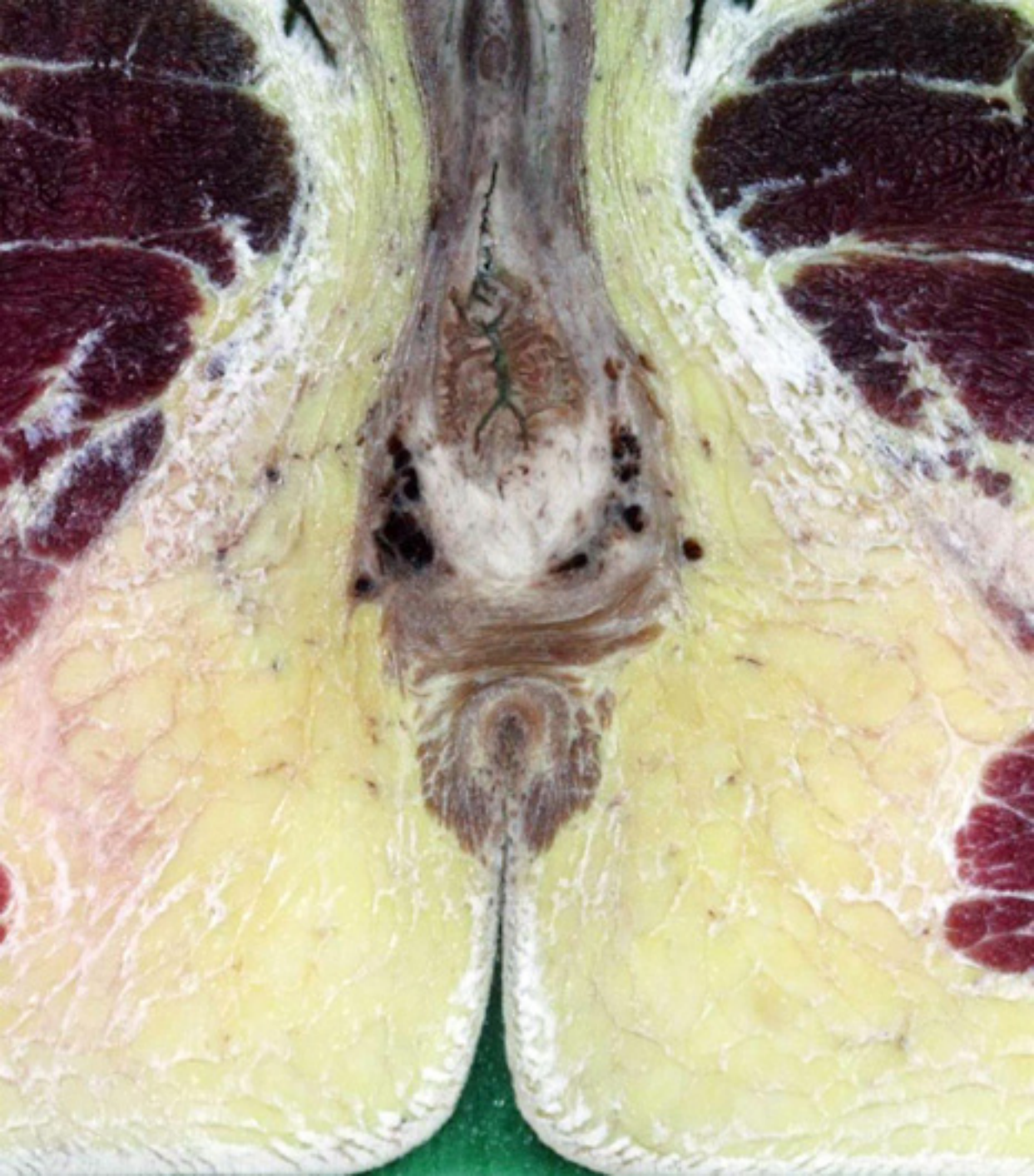

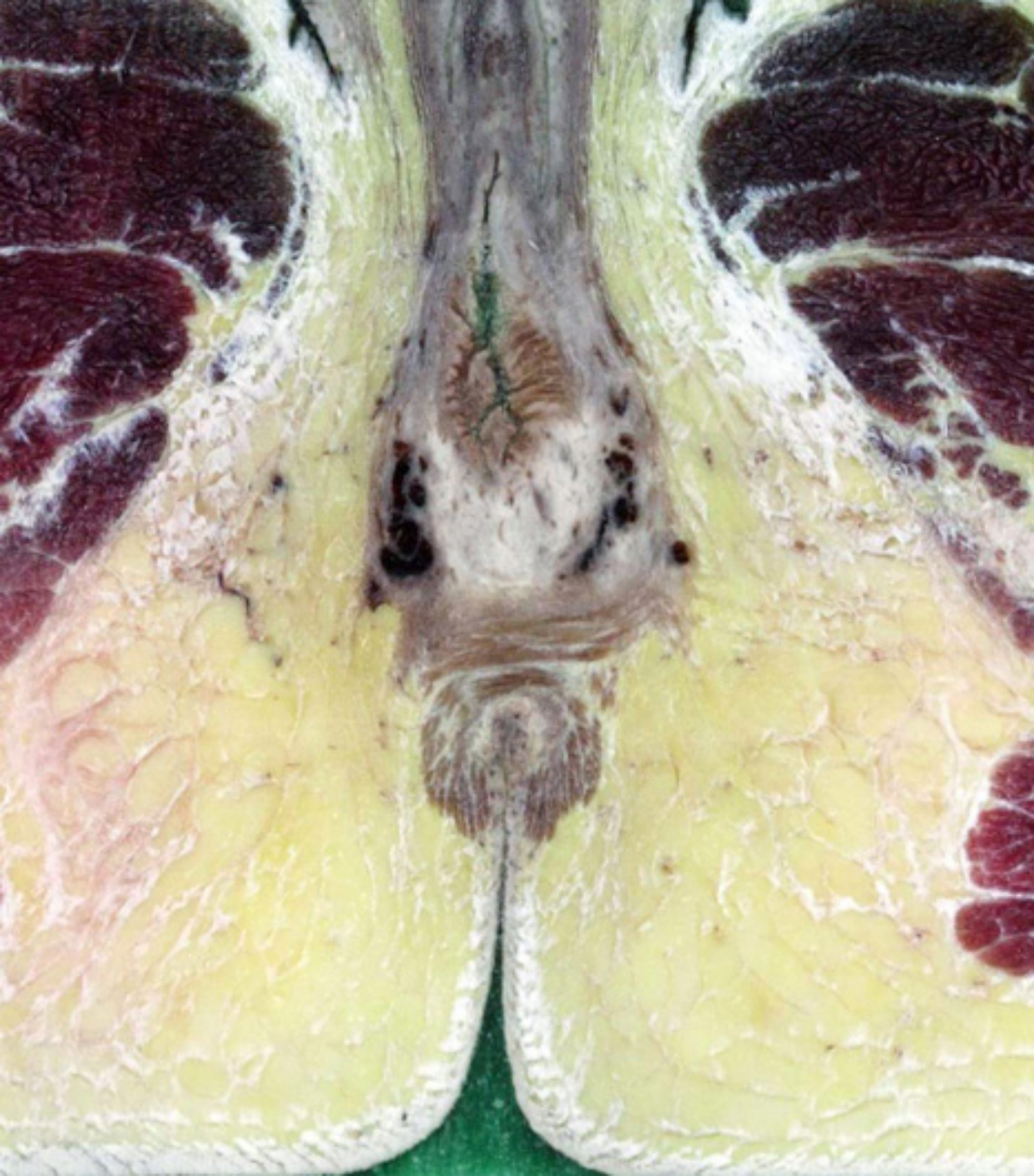

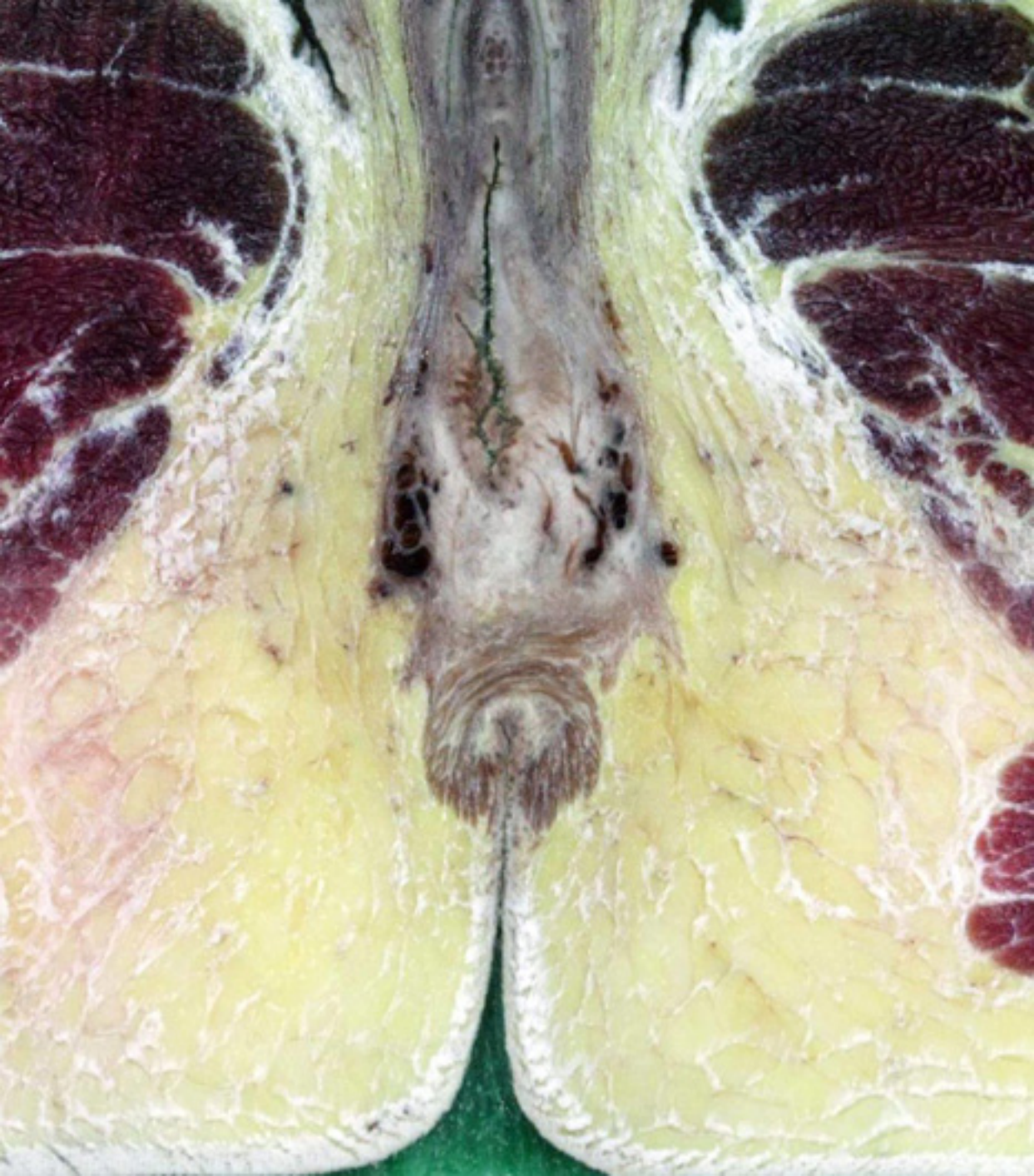

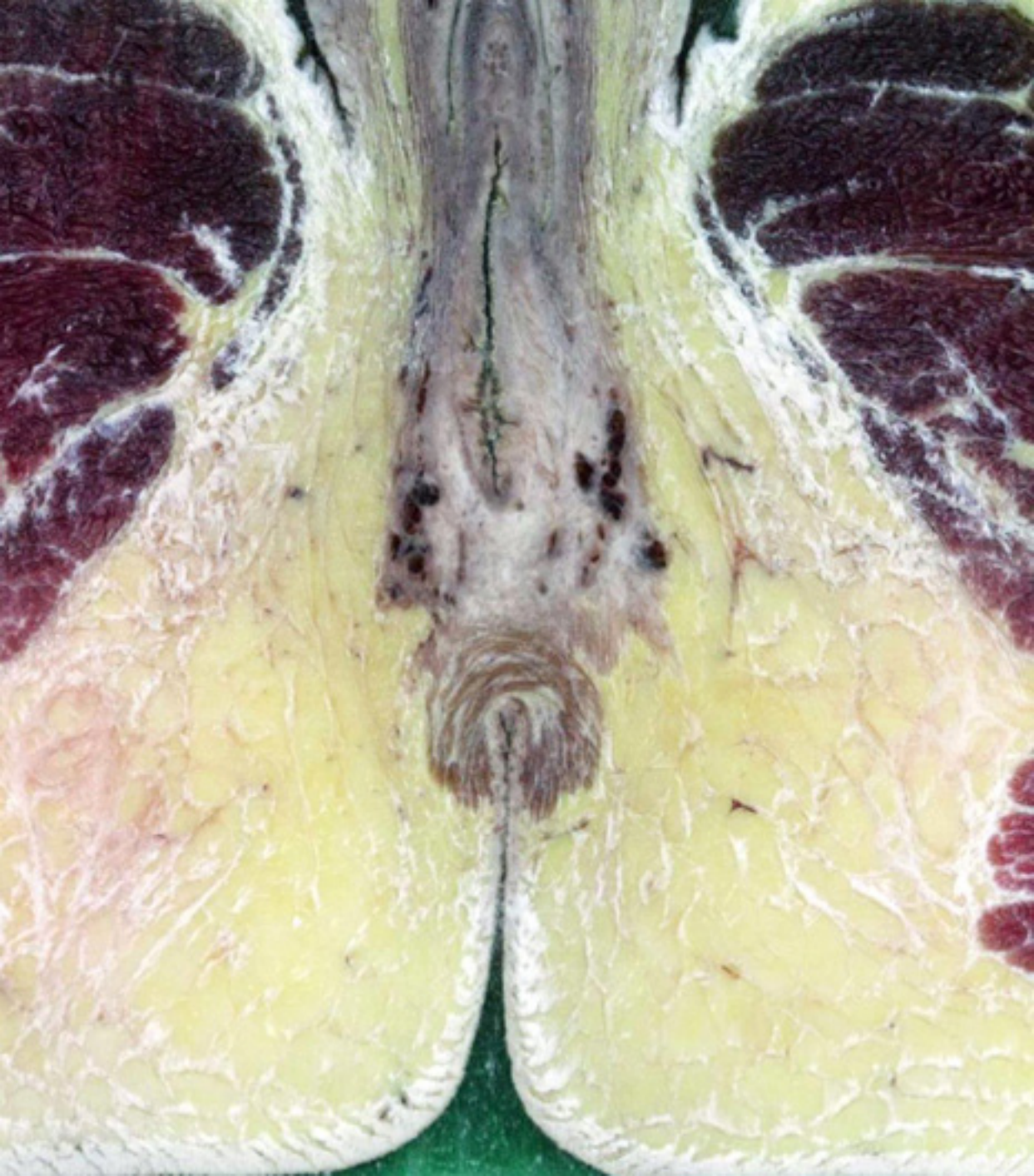

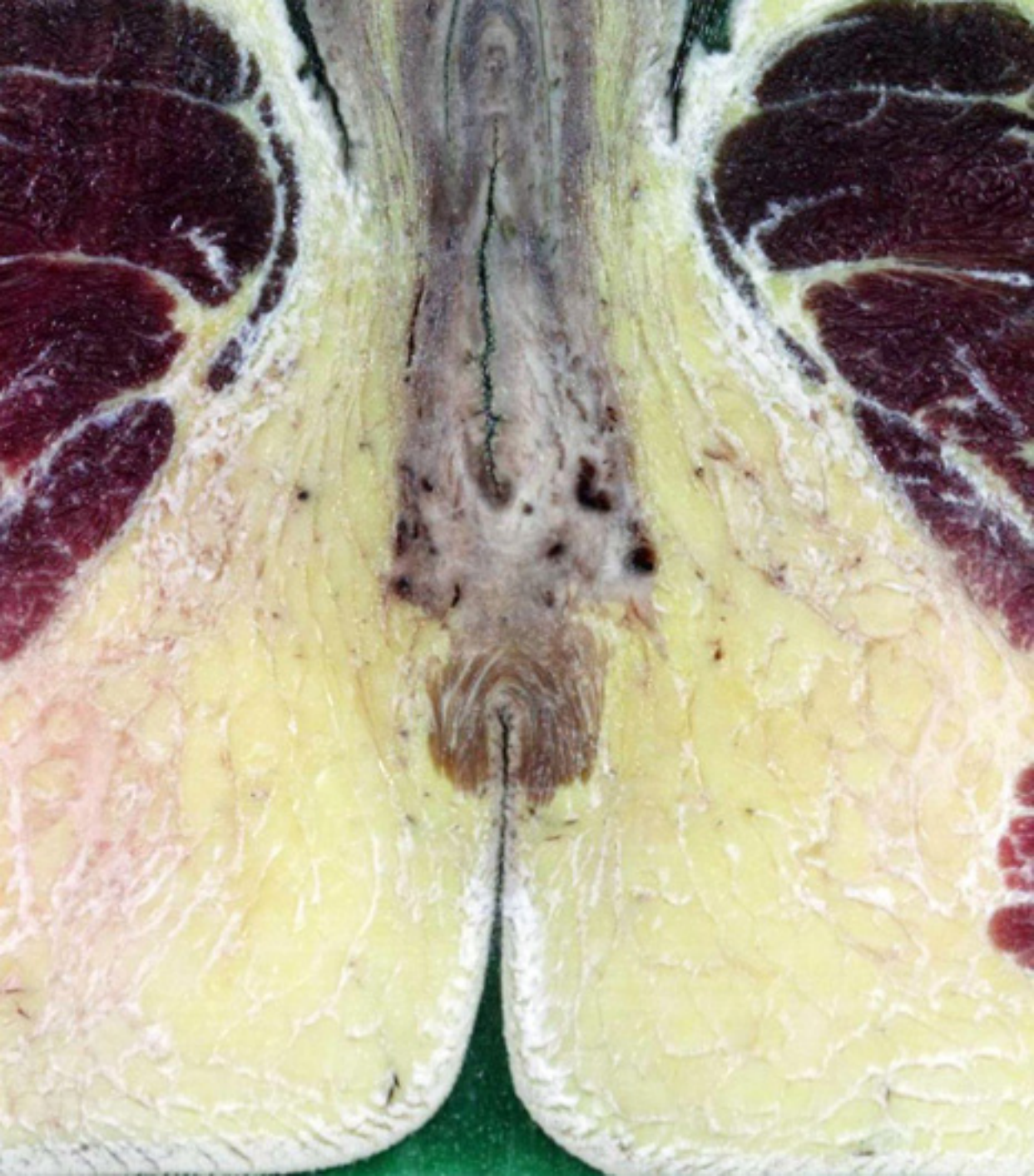

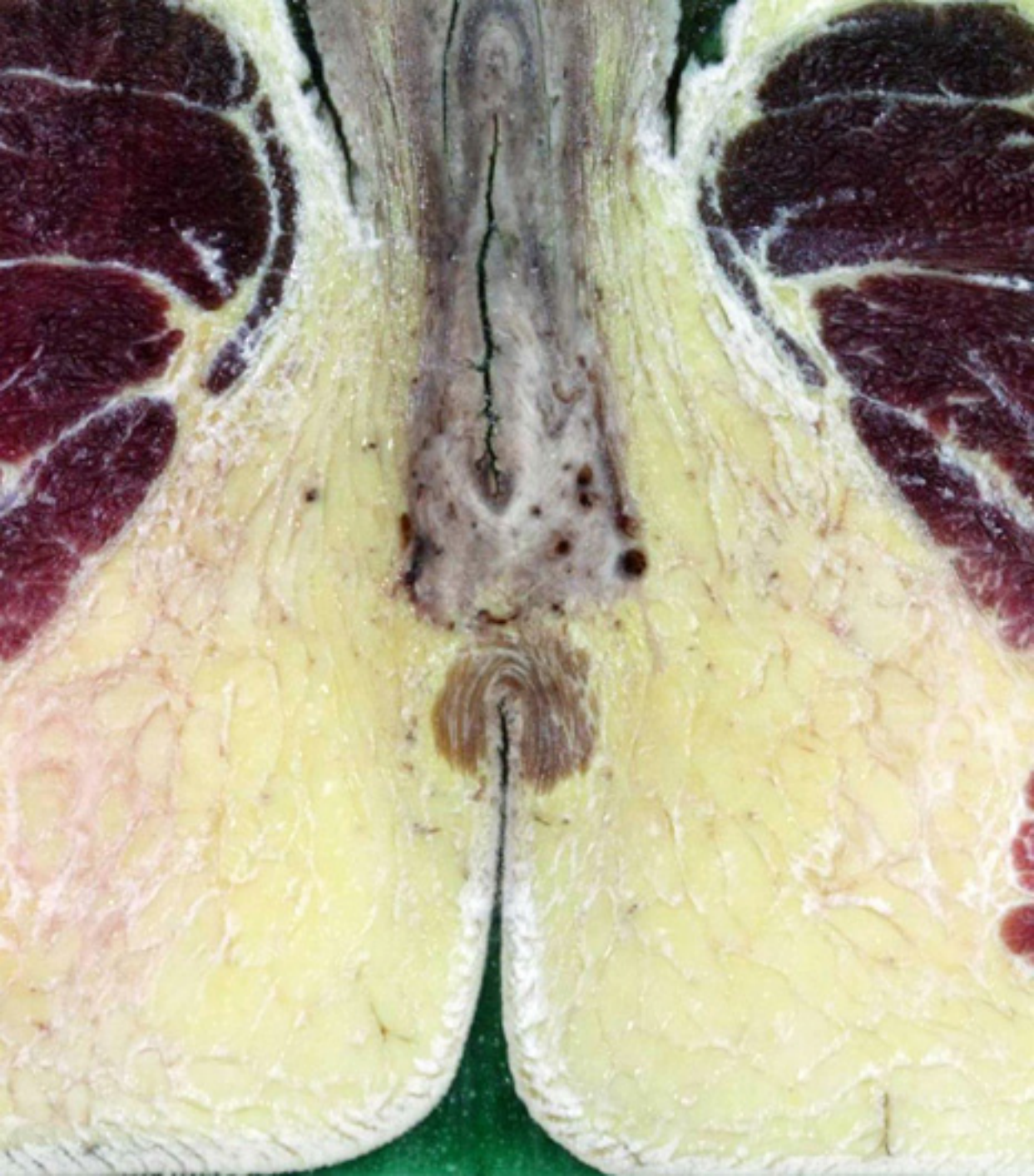

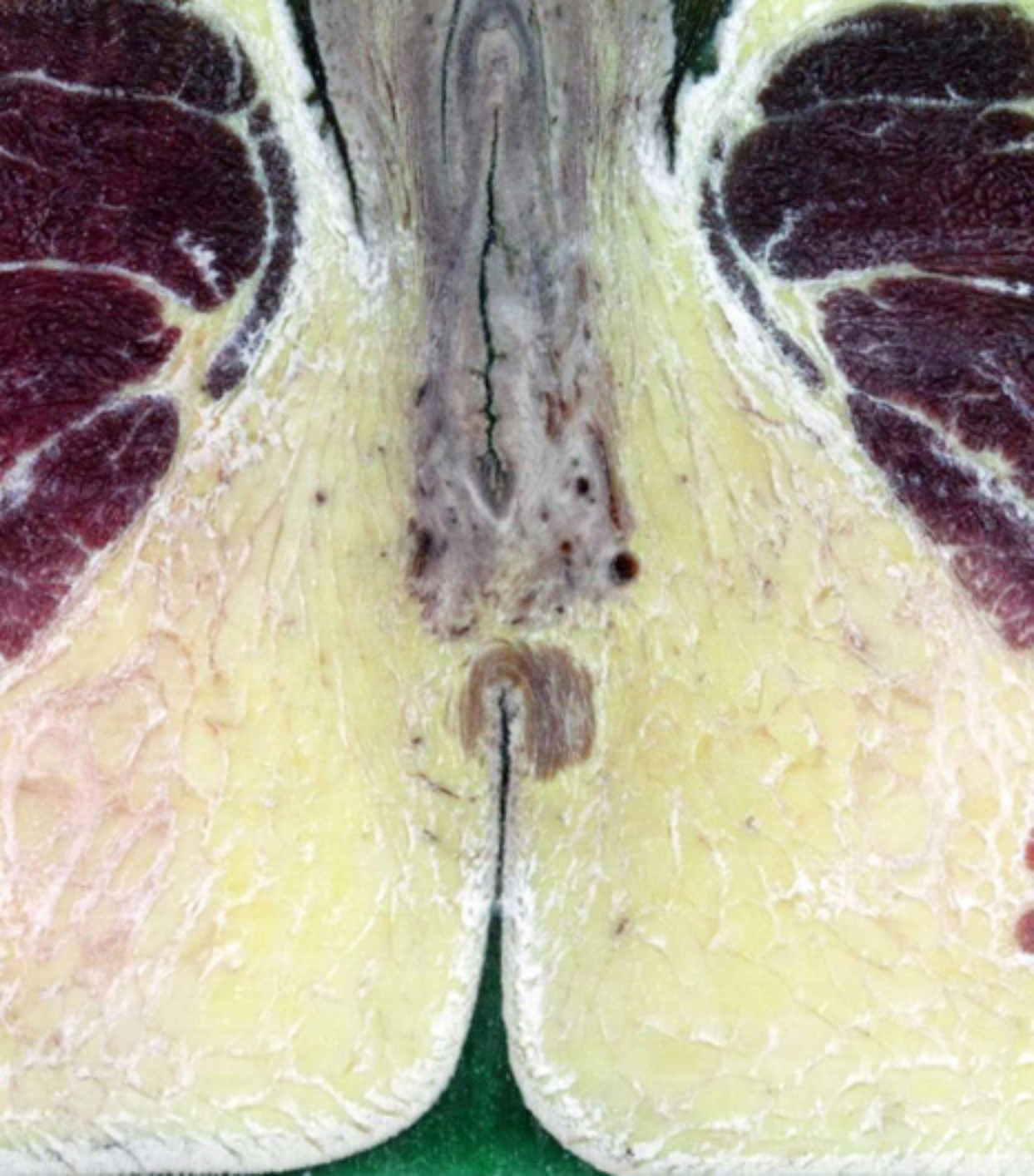

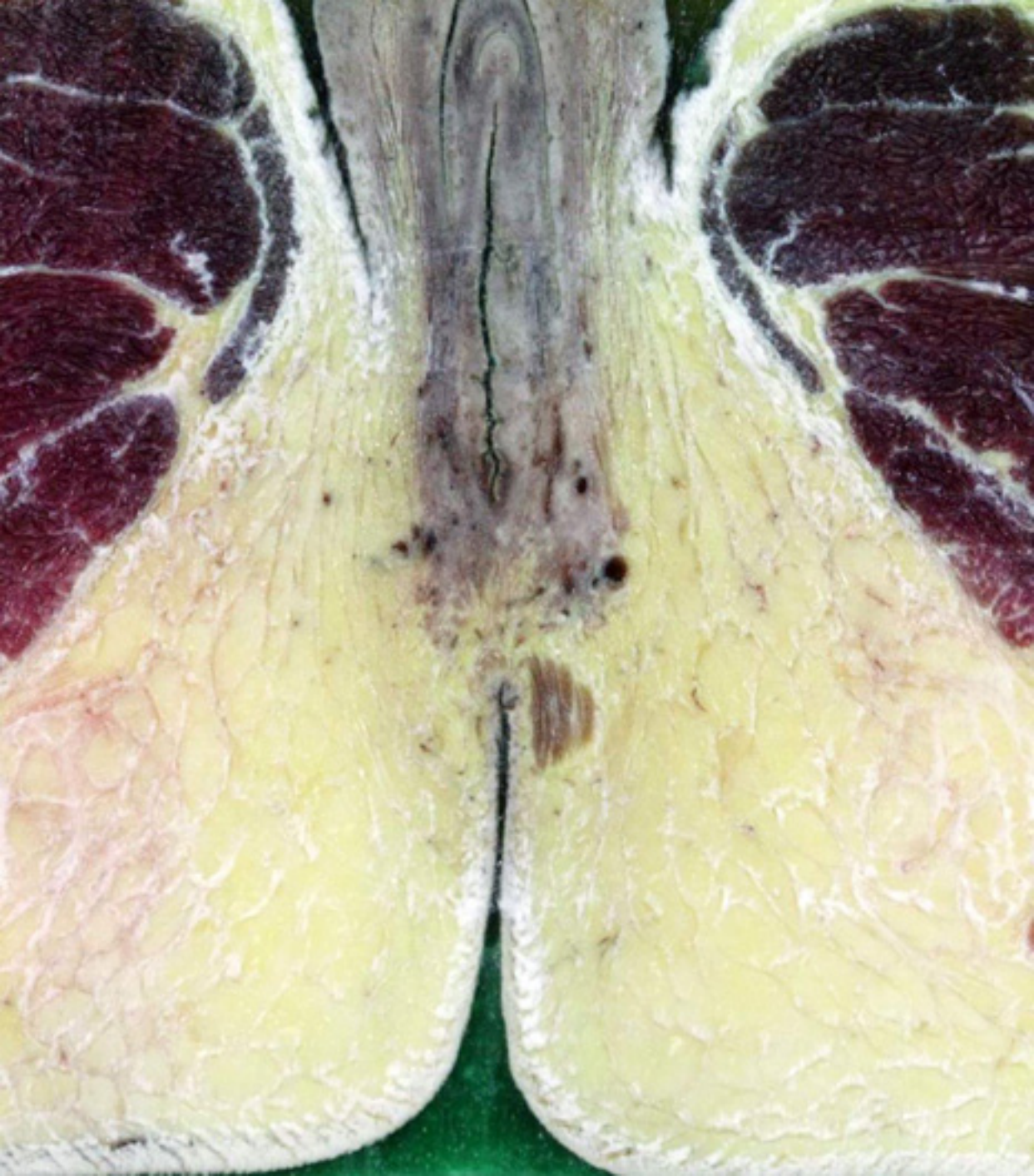

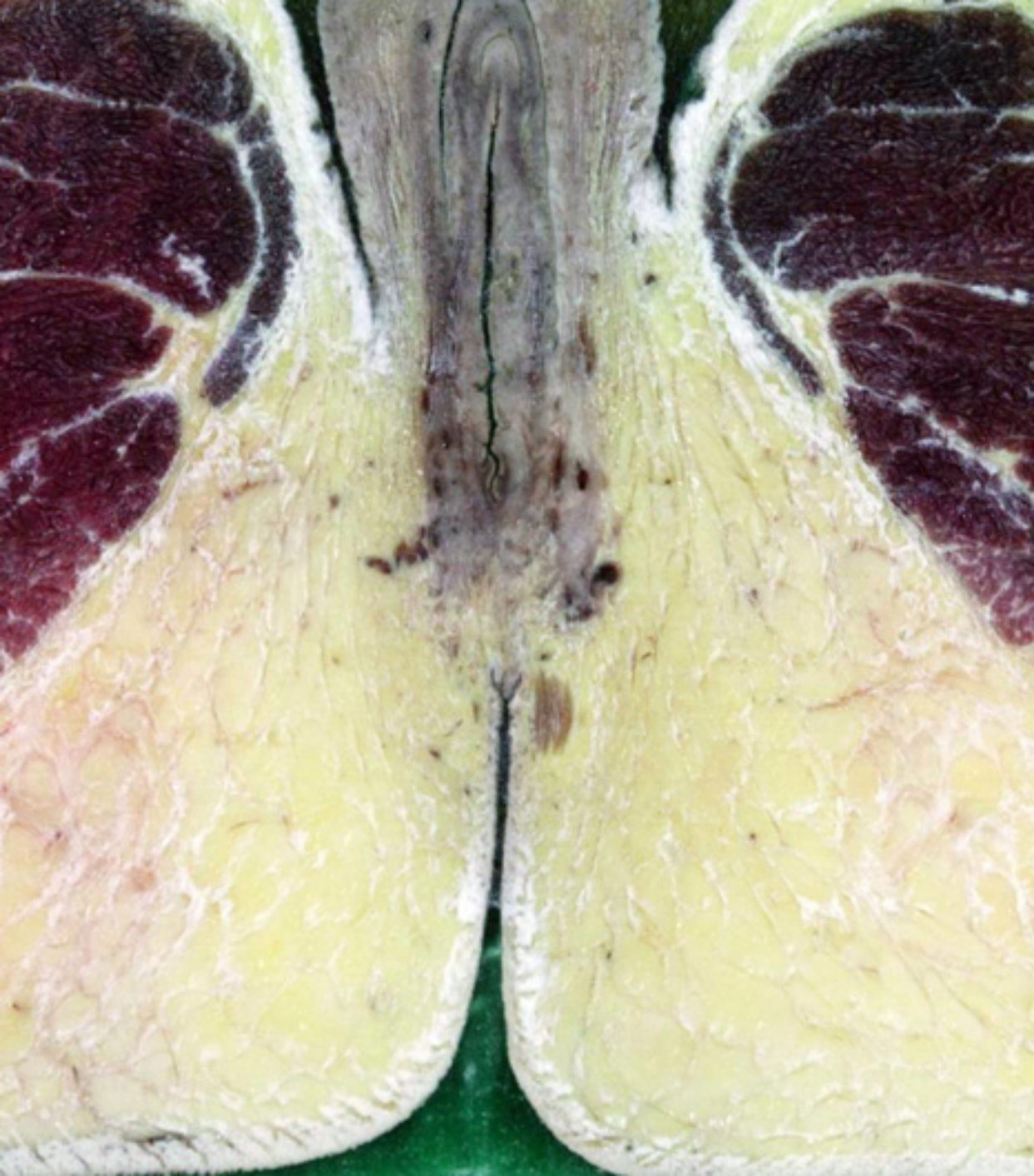

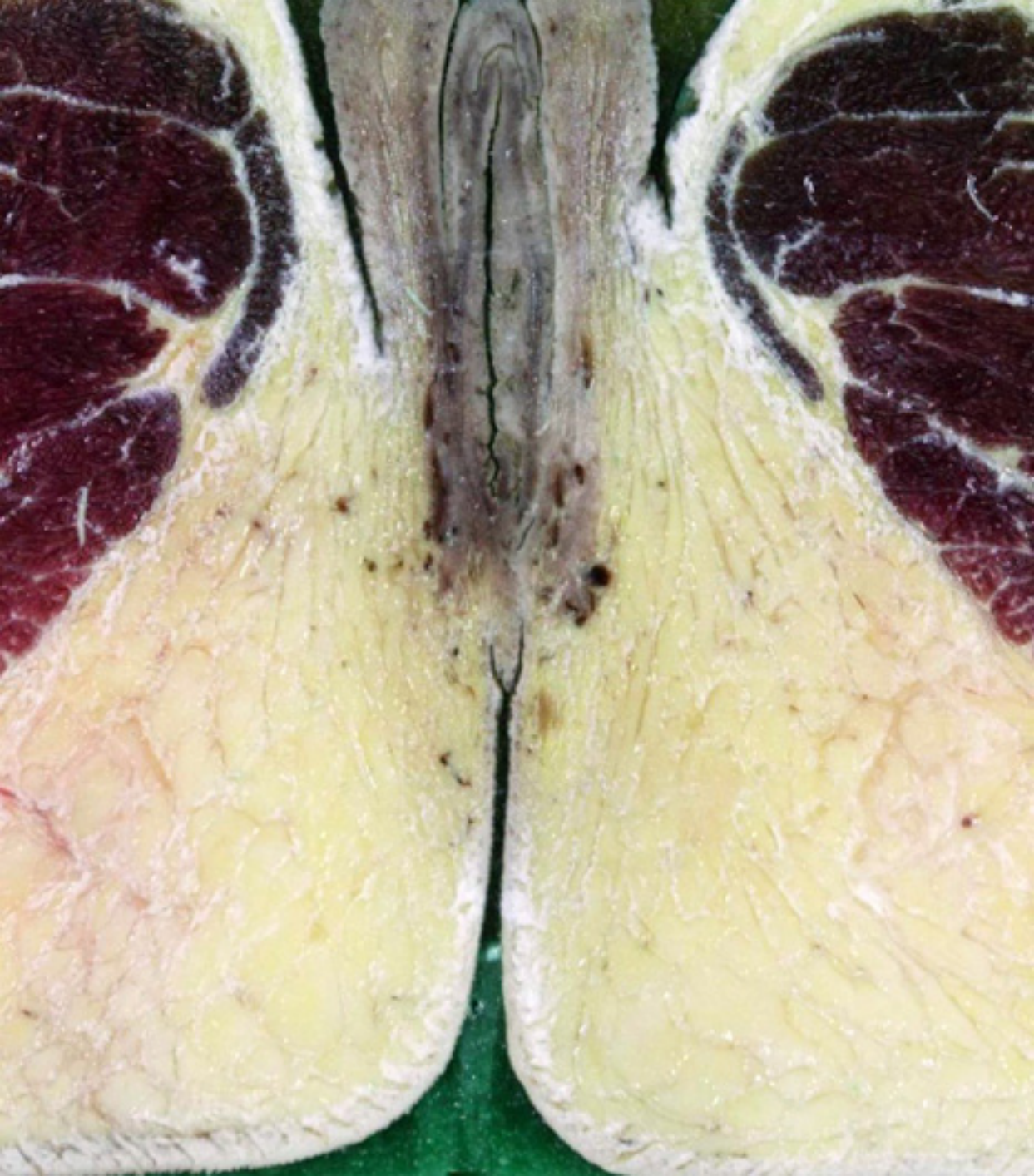

Supplement: S4 Fig — All Figures were magnified 1.3-fold. The panel labels are retained. (PDF) [file pone.0132226.s004.pdf]
