# Supplementary material for: 3D Topography of the Young Adult Anal Sphincter Complex Reconstructed from Undeformed Serial Anatomical Sections
Source: PLoS One. 2015 Aug 25;10(8):e0132226. doi: 10.1371/journal.pone.0132226 (PMC4549266; doi:10.1371/journal.pone.0132226)
Supplement: S1 Table — (DOCX) [file pone.0132226.s009.docx]

**S1 Table A. Biometric data of the CVH bodies and details of sections.**

|  | **CVH1** | **CVH2** | **CVH3** | **CVH4** | **CVH5** | **CVO** |
| --- | --- | --- | --- | --- | --- | --- |
|  | male | female | male | female | female | female |
| Age | 35 | 22 | 21 | 25 | 25 | 35 |
| Height (mm) | 1700 | 1620 | 1820 | 1620 | 1700 | * |
| Weight (kg) | 65 | 54 | 66 | 57.5 | 59 | * |
| Sectioning direction | transverse | transverse | transverse | transverse | transverse | sagittal |
| Section thickness (mm) | 1.0 | 0.5 | 0.1 | 0.5 | 0.2 | 0.2 |
| Image Resolution | 3073×2048 | 3072×2048 | 4064×2704 | 4064×2704 | 4064×2704 | 4064×2704 |

*: only lower abdomen and pelvis were sectioned.

**S1 Table B. Biometric data of the pelvic floor in CVH specimens.**

|  | **females** | **males** |
| --- | --- | --- |
| Pubococcygeal line | 94±3 | 85±12 |
| “H”-line | 61±4 | 60±10 |
| “M”-line | 15±3 | 9±3 |
| “WLH”-line | 23±3 | 21±4 |
| length IAS | 26±2 | 31±4 |
| thickness IAS | ~3 | ~3 |
| volume LAM | 26±1 | 21±4 |
| volume perineal body | 1.0-4.4 | 0.6-1.2 |

Length is given in mm; volume in cm^3^. Pubococcygeal line: pubic arch to coccyx; “H”-line: pubic arch to posterior anorectal junction (~ anteroposterior length of levator hiatus); “M”-line: posterior anorectal junction to pubococcygeal line (~ descent of levator hiatus); “WLH”-line: width of levator hiatus.

**S1 Table C. List of structures identified and reconstructed in S1 Fig.**

Name used in present study Terminologia Anatomica^[1](#_ENREF_1)^

1. Internal obturator muscle
2. Coccygeal muscle
3. Pubovisceral muscle (inner layer) Pubococcygeal and iliococcygeal muscles
4. Pubovisceral muscle (external layer) Pubococcygeal and iliococcygeal muscles
5. Puborectal muscle (deep part) Puborectal muscle / Deep layer external anal sphincter
6. Puborectal muscle (superficial part) Superficial layer external anal sphincter
7. Rectal lumen
8. Smooth muscle of rectum
9. Mesorectum
10. Rectococcygeal muscle
11. Rectoperineal muscle
12. Internal anal sphincter
13. Conjoint longitudinal muscle of rectum
14. External anal sphincter Subcutaneous layer external anal sphincter
15. Anal intermuscular septum
16. Anococcygeal ligament
17. Ovary
18. Uterine wall
19. Uterine tube
20. Uterine and vaginal lumen
21. Round ligament of uterus
22. Vaginal wall
23. Bartholin’s gland
24. Denonvilliers’ fascia
25. Perineal body
26. Deep perineal muscle Deep transverse perineal muscle
27. Superficial transverse perineal muscle
28. Bladder wall
29. Ureter
30. Pubovesical ligament
31. Bladder and urethral lumen
32. Urethral sphincter proper External urethral sphincter
33. Raphe of urethral sphincter
34. Urethral compressor muscle External urethral sphincter
35. Urethro-vaginal sphincter External urethral sphincter
36. Submucous layer of urethra
37. Ischiocavernous muscle
38. Bulbospongiosus muscle
39. Vestibular bulb
40. Cavernous body of clitoris
41. Peritoneum
42. Fat tissue
43. Arteries
44. Veins
45. Pelvic bone
46. Sacral and coccygeal bone
47. Skin

**S1 Table D. Terminology of muscles in present study compared to that in Terminologia Anatomica^1^ (TA; levator and sphincter ani muscles)**

| **Present study** | **LAM (TA)** | **EAS (TA)** |
| --- | --- | --- |
| pubovisceral | pubococcygeal + iliococcygeal | -- |
| levator ani muscle | (pubo- & iliococcygeal) + puborectal | -- |
| puborectal (deep portion) | puborectal | deep portion |
| puborectal (superficial portion) | -- | superficial portion |
| external anal sphincter (proper) | -- | subcutaneous portion |

^1^ Federative Committee on Anatomical Terminology (1998) Terminologia Anatomica.

Thieme, New York.
