# Supplementary figures and images for: Correction: 3D Topography of the Young Adult Anal Sphincter Complex Reconstructed from Undeformed Serial Anatomical Sections
Source: PLoS One. 2015 Oct 9;10(10):e0140736. doi: 10.1371/journal.pone.0140736 (PMC4599863; doi:10.1371/journal.pone.0140736)

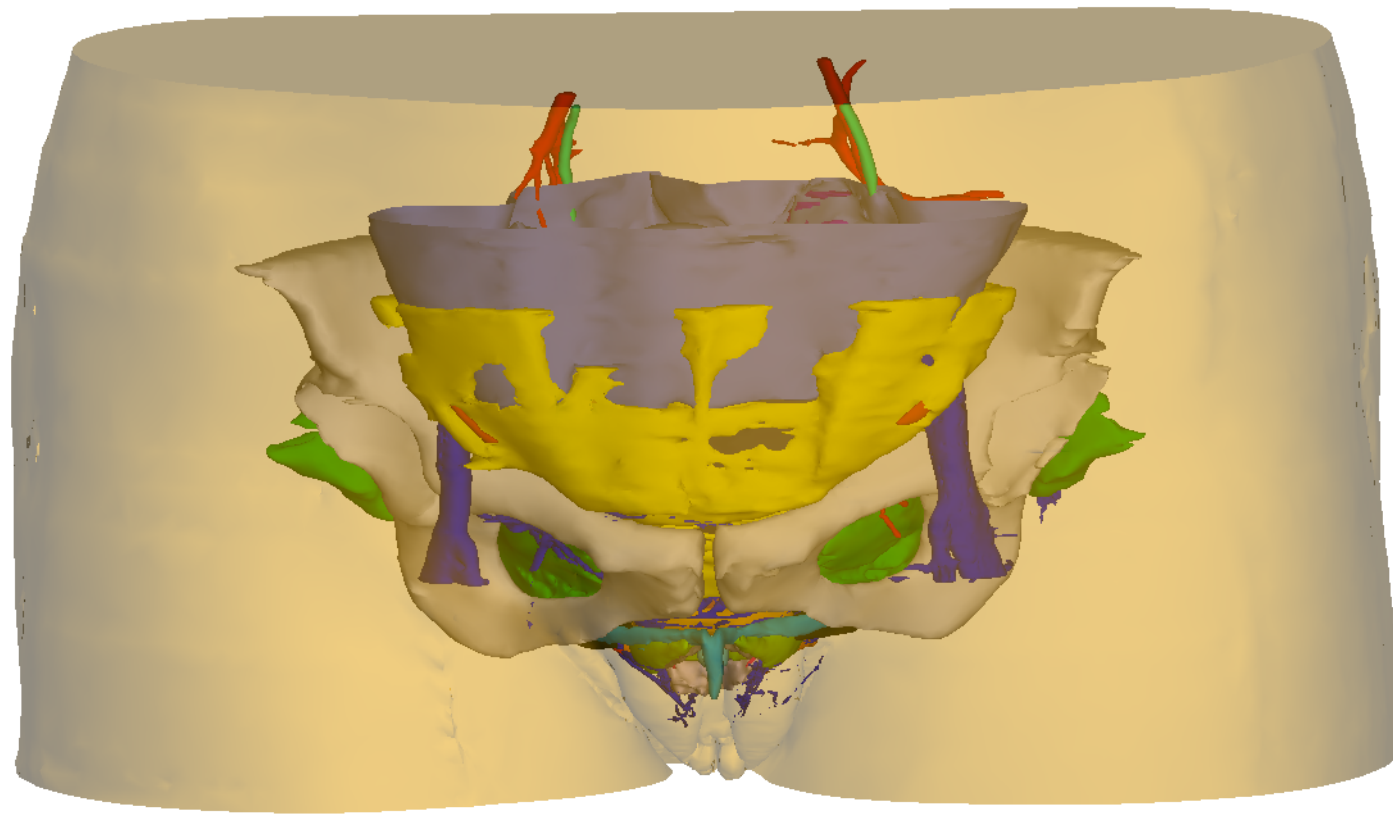

Supplement: S5 Fig — All Figures were magnified 1.3-fold. The panel labels are retained. (PDF) [file pone.0140736.s004.PDF]

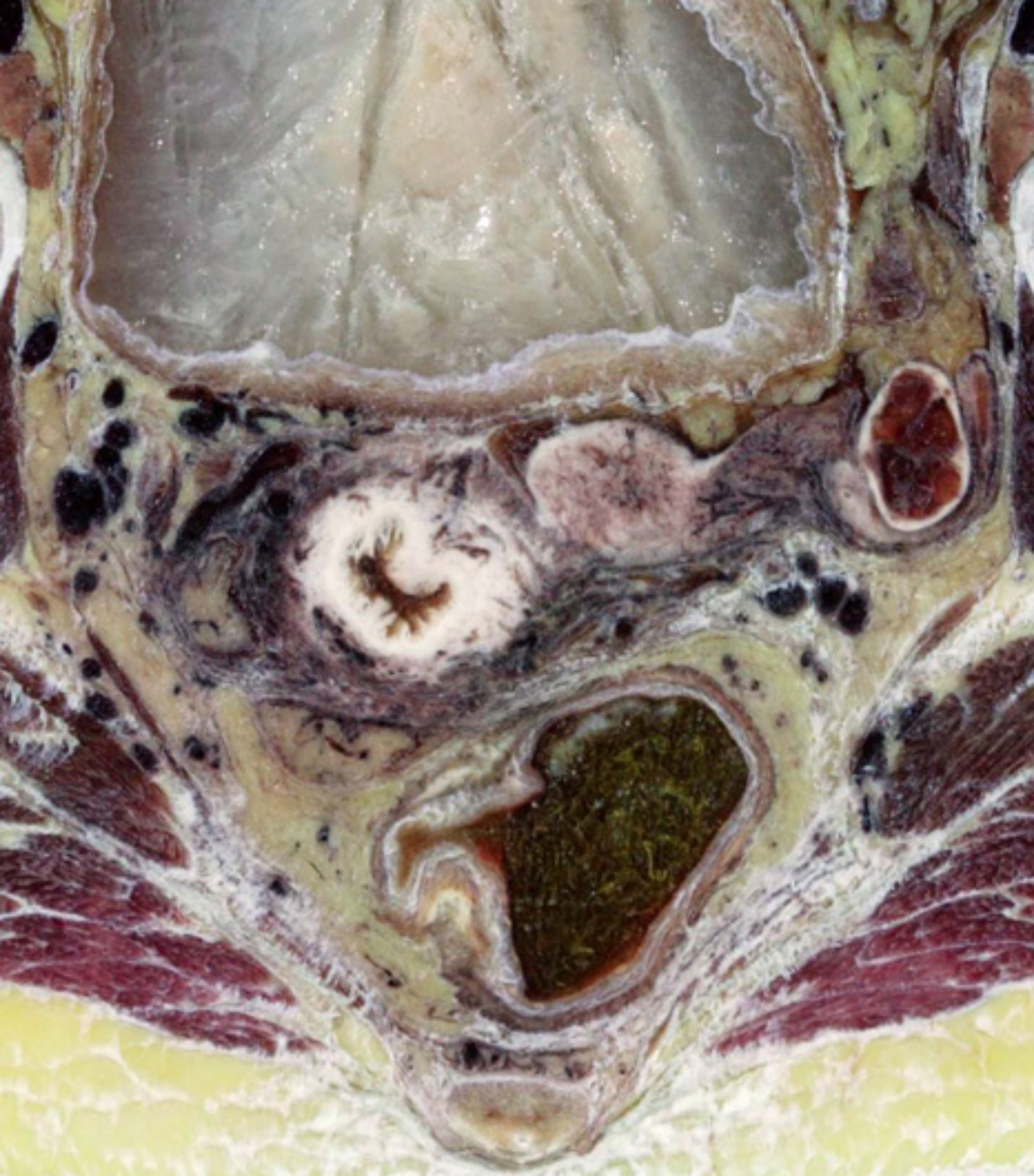

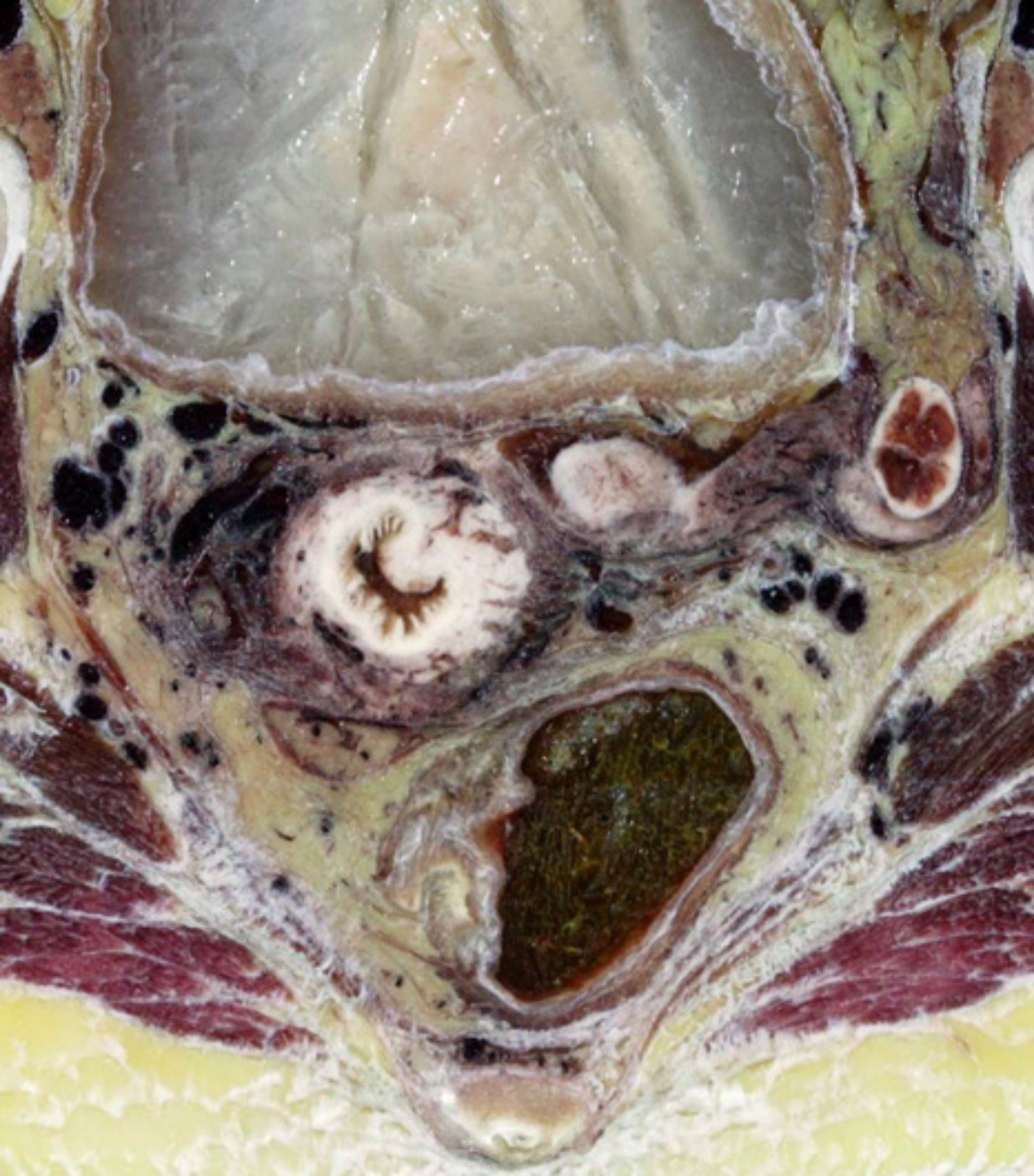

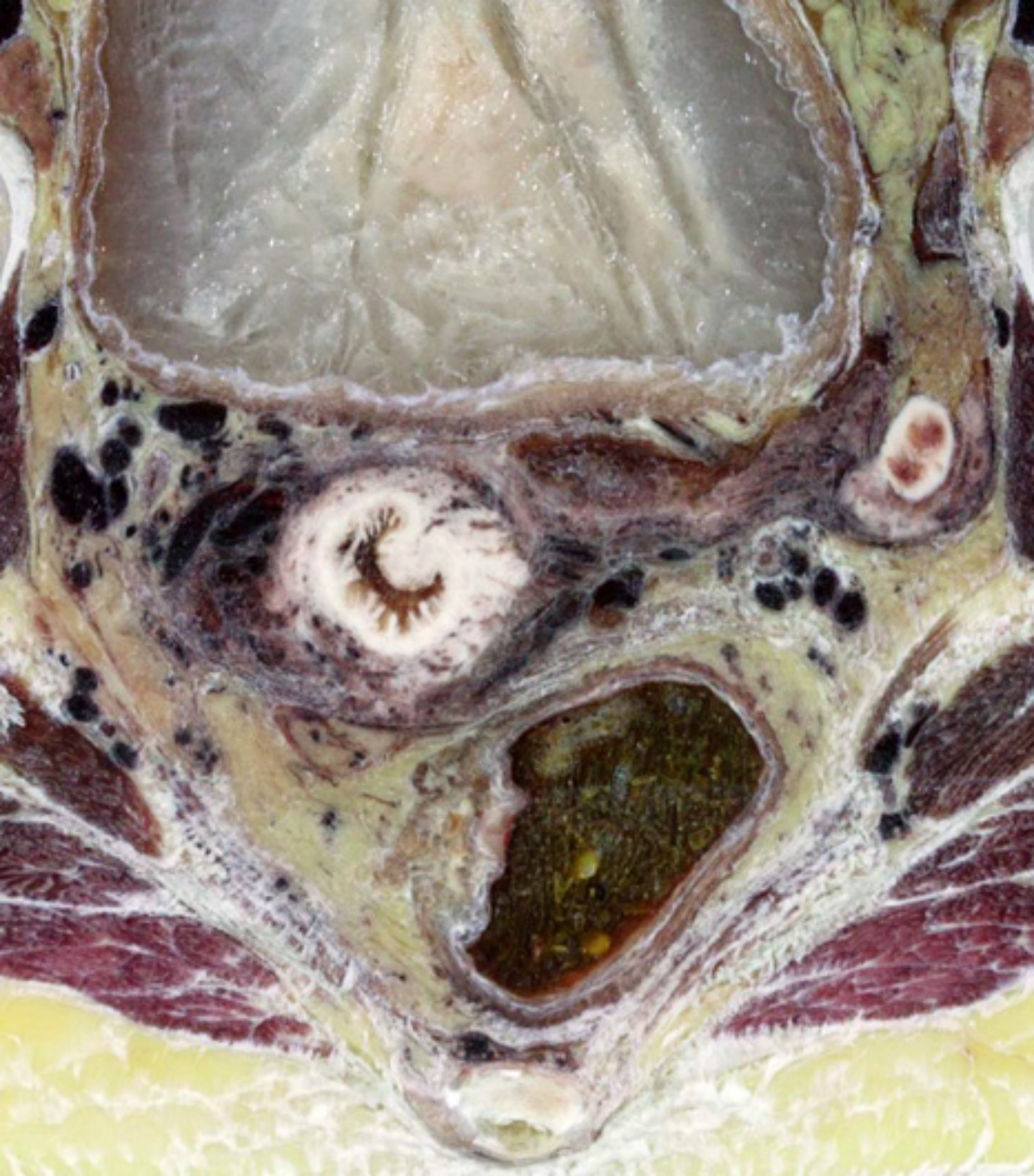

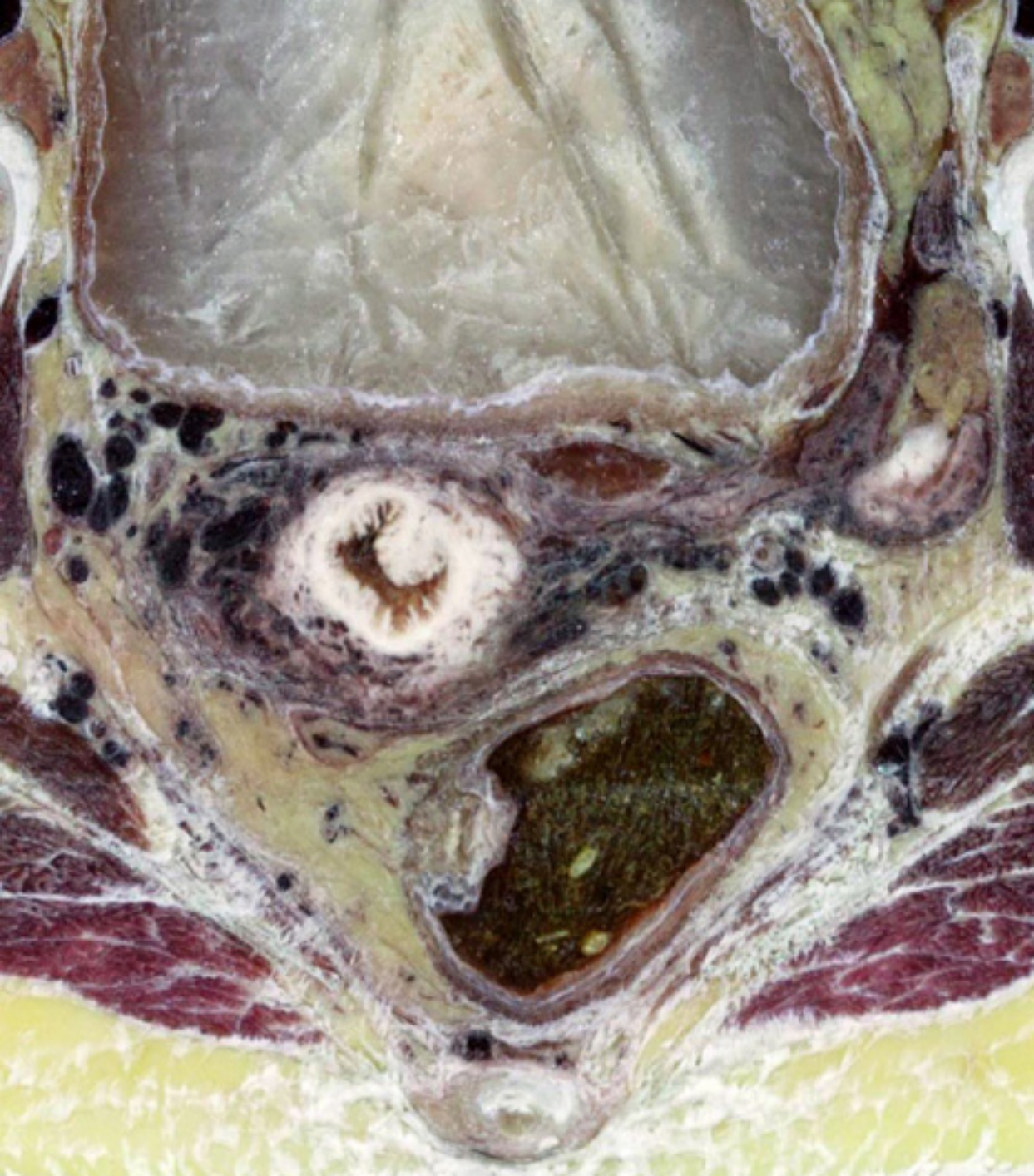

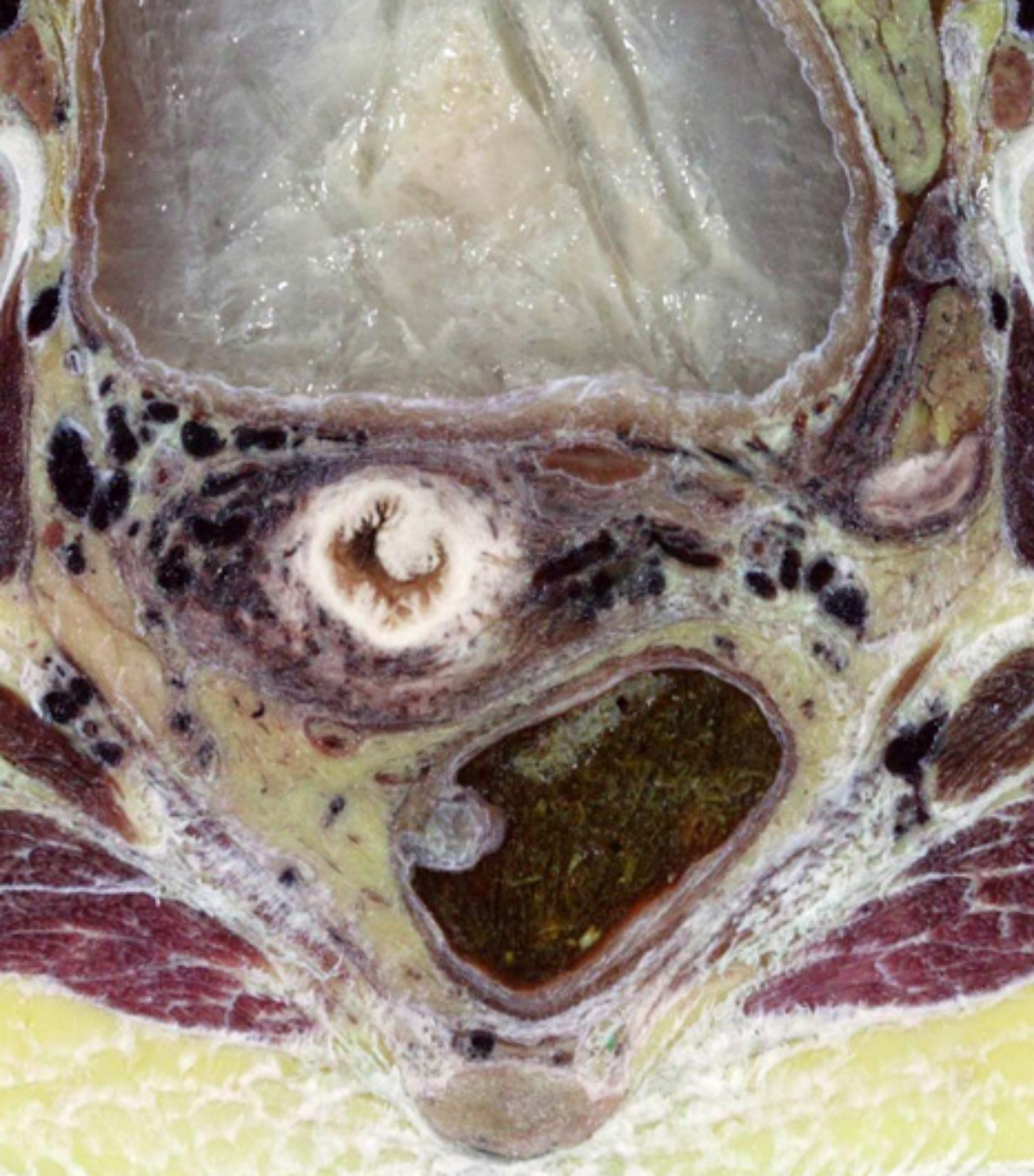

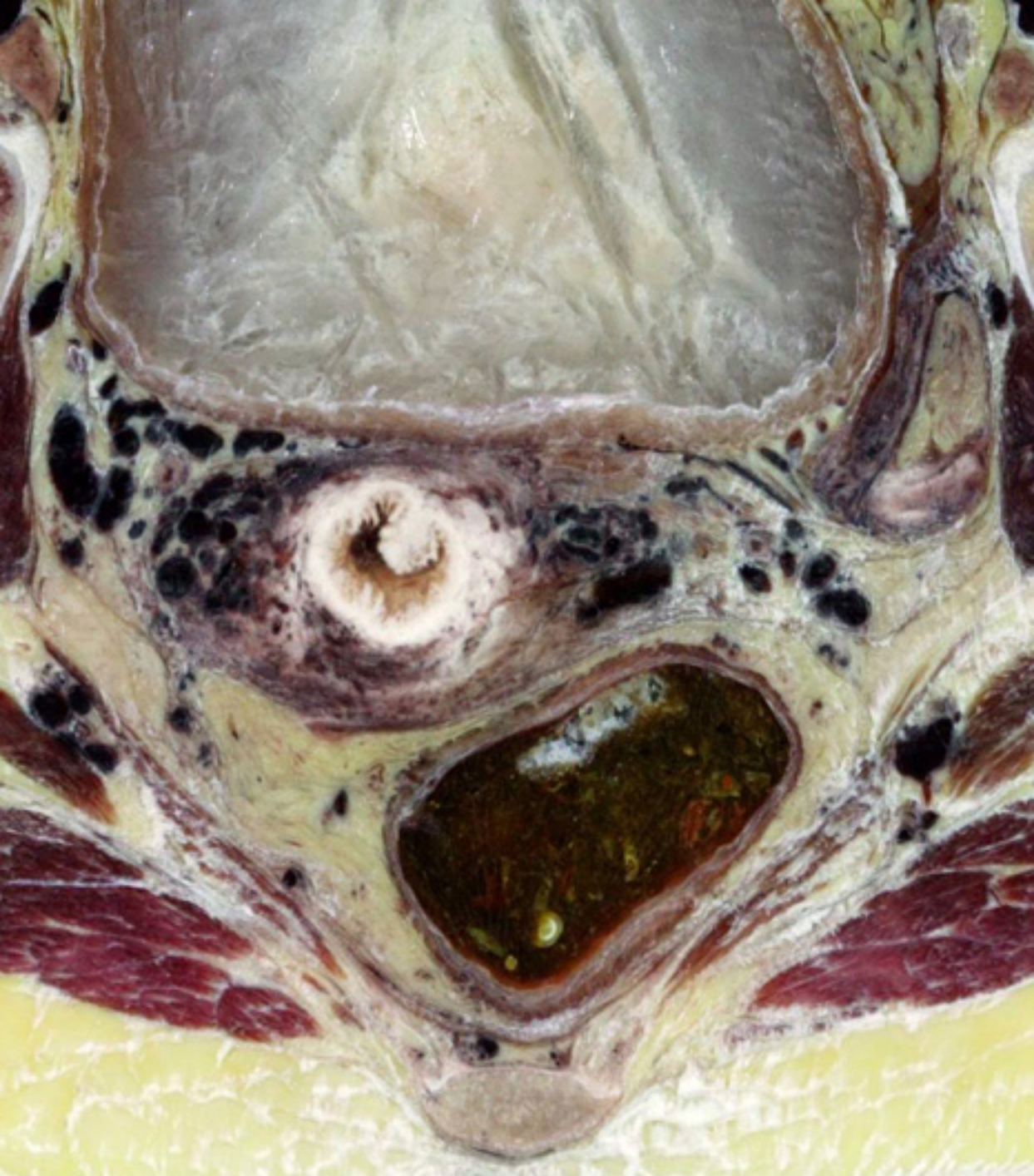

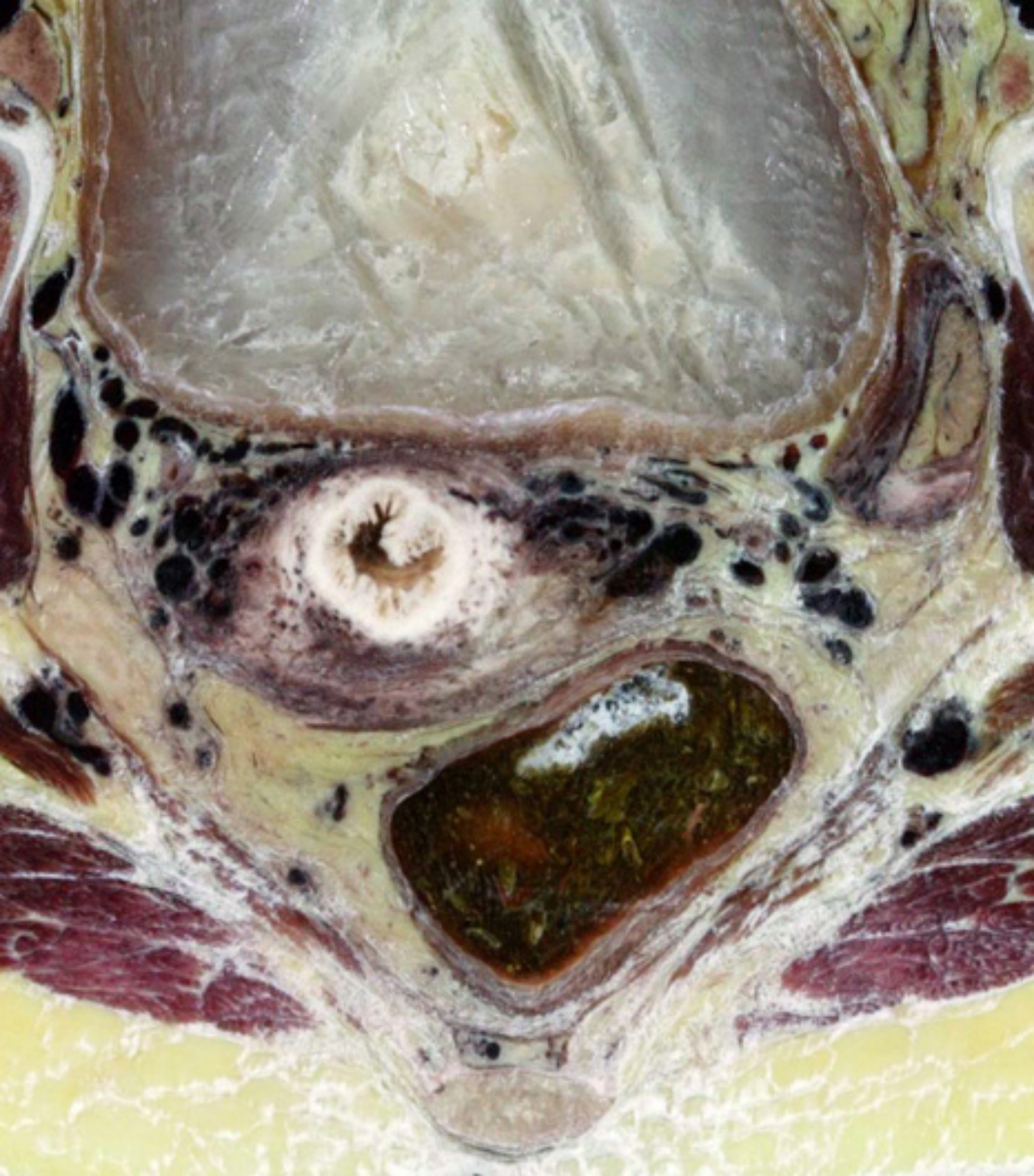

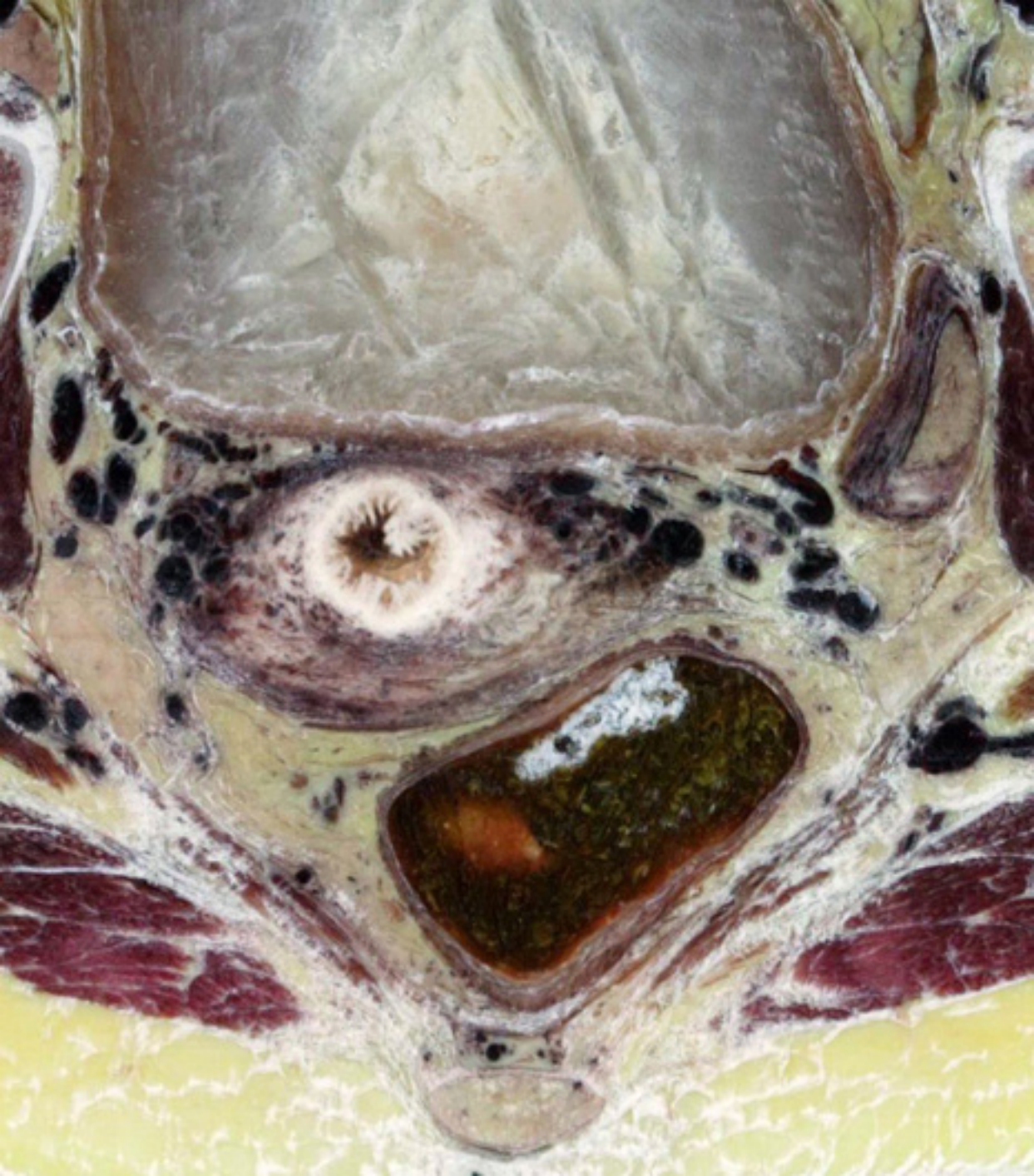

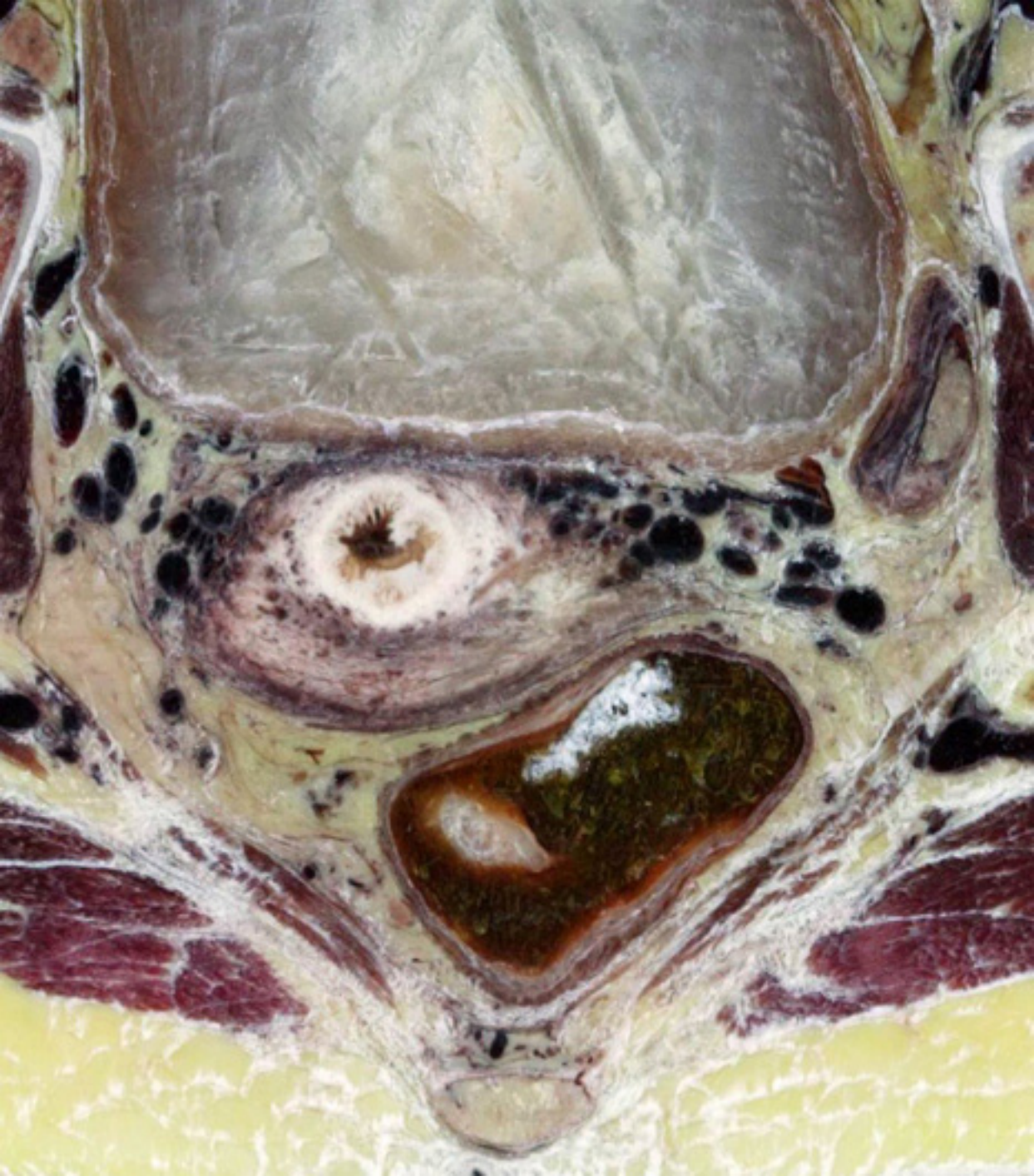

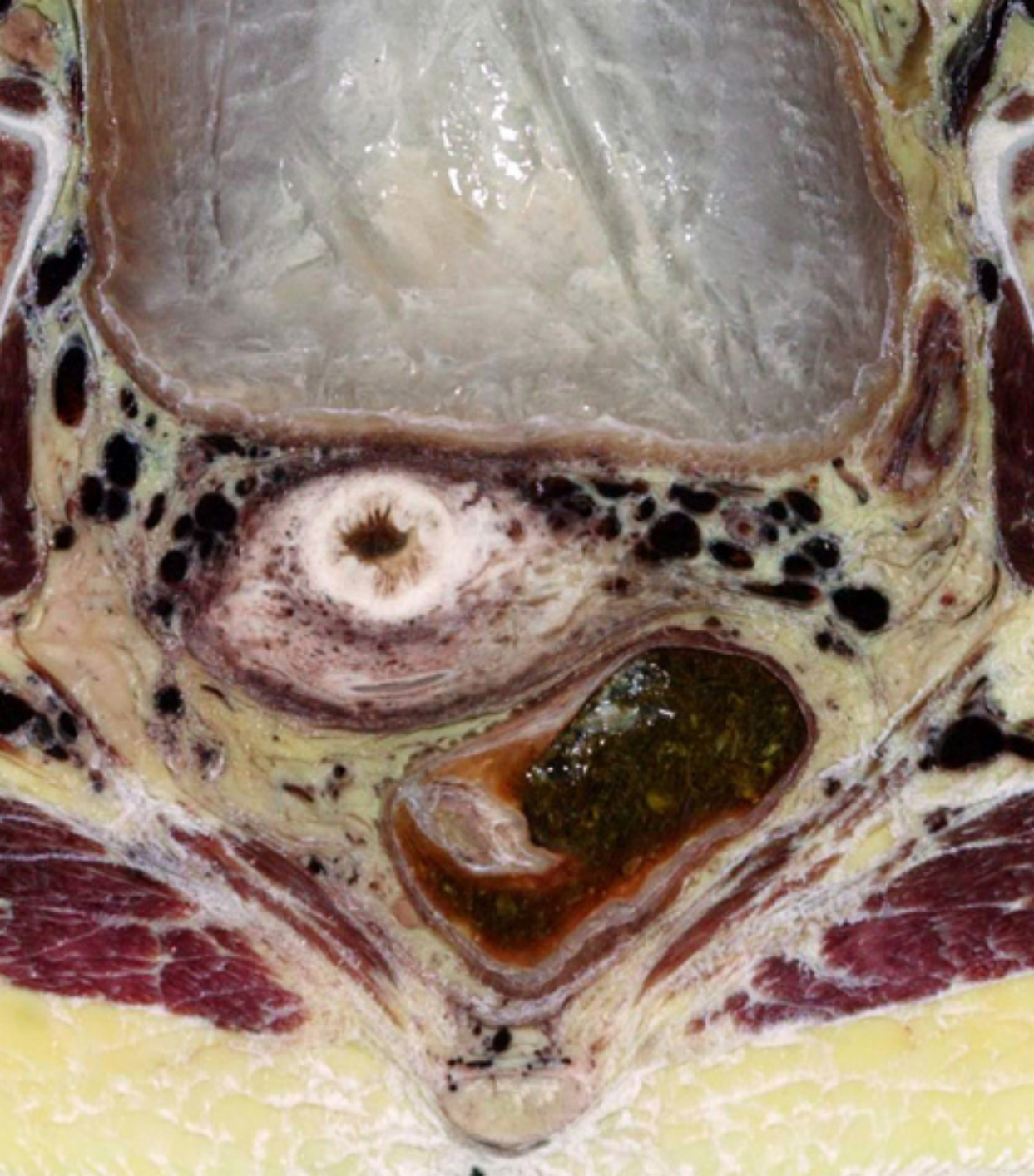

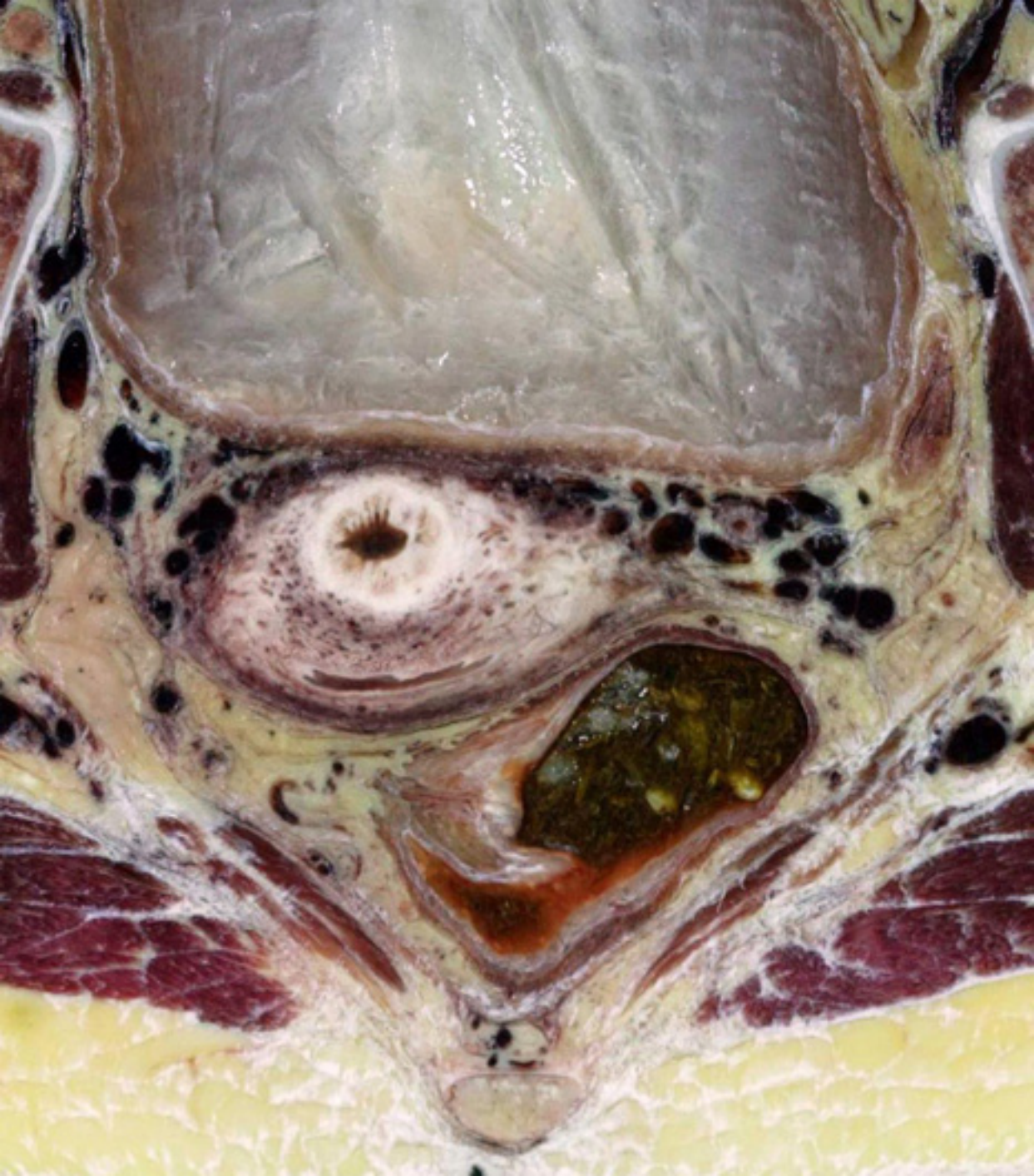

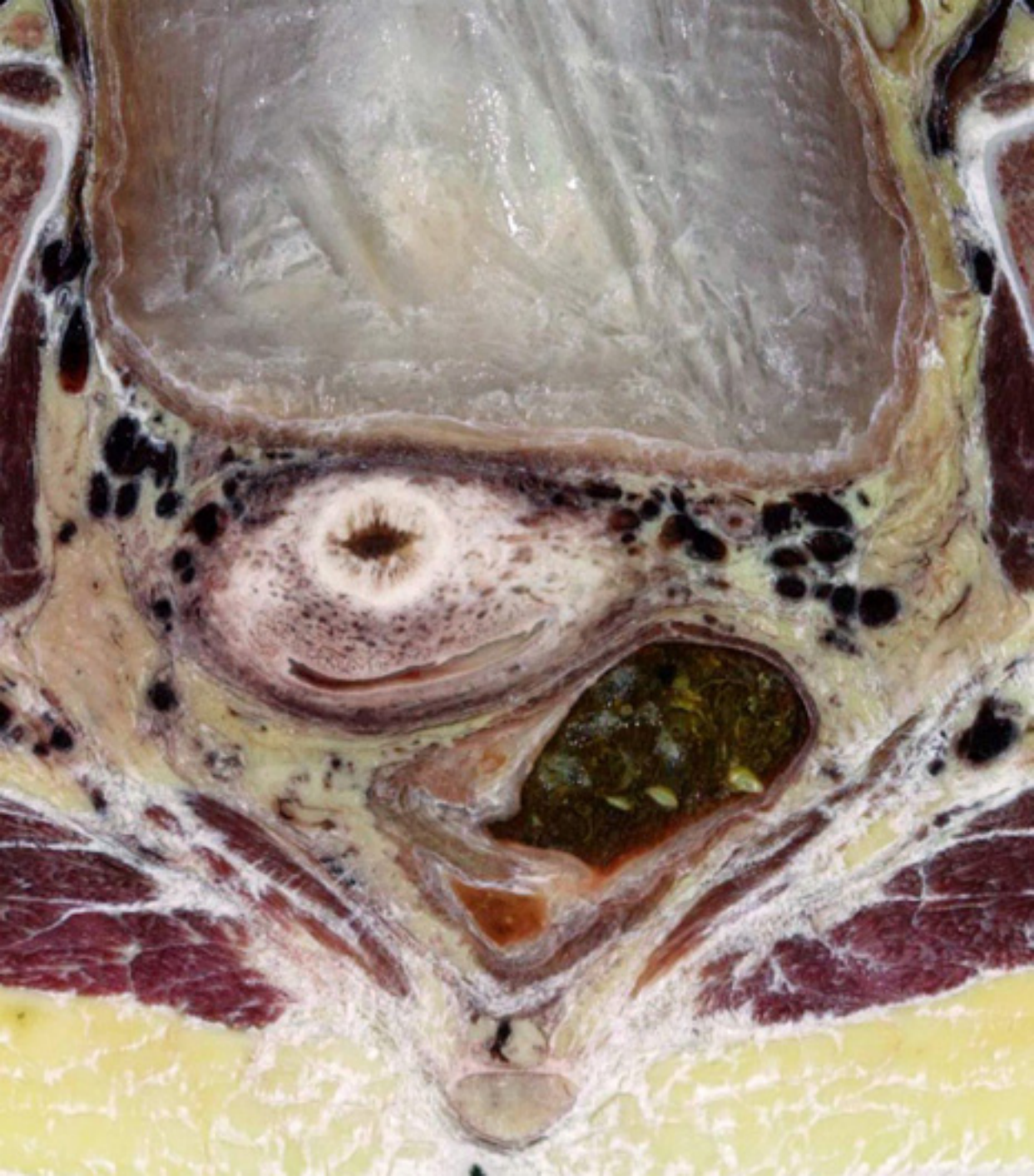

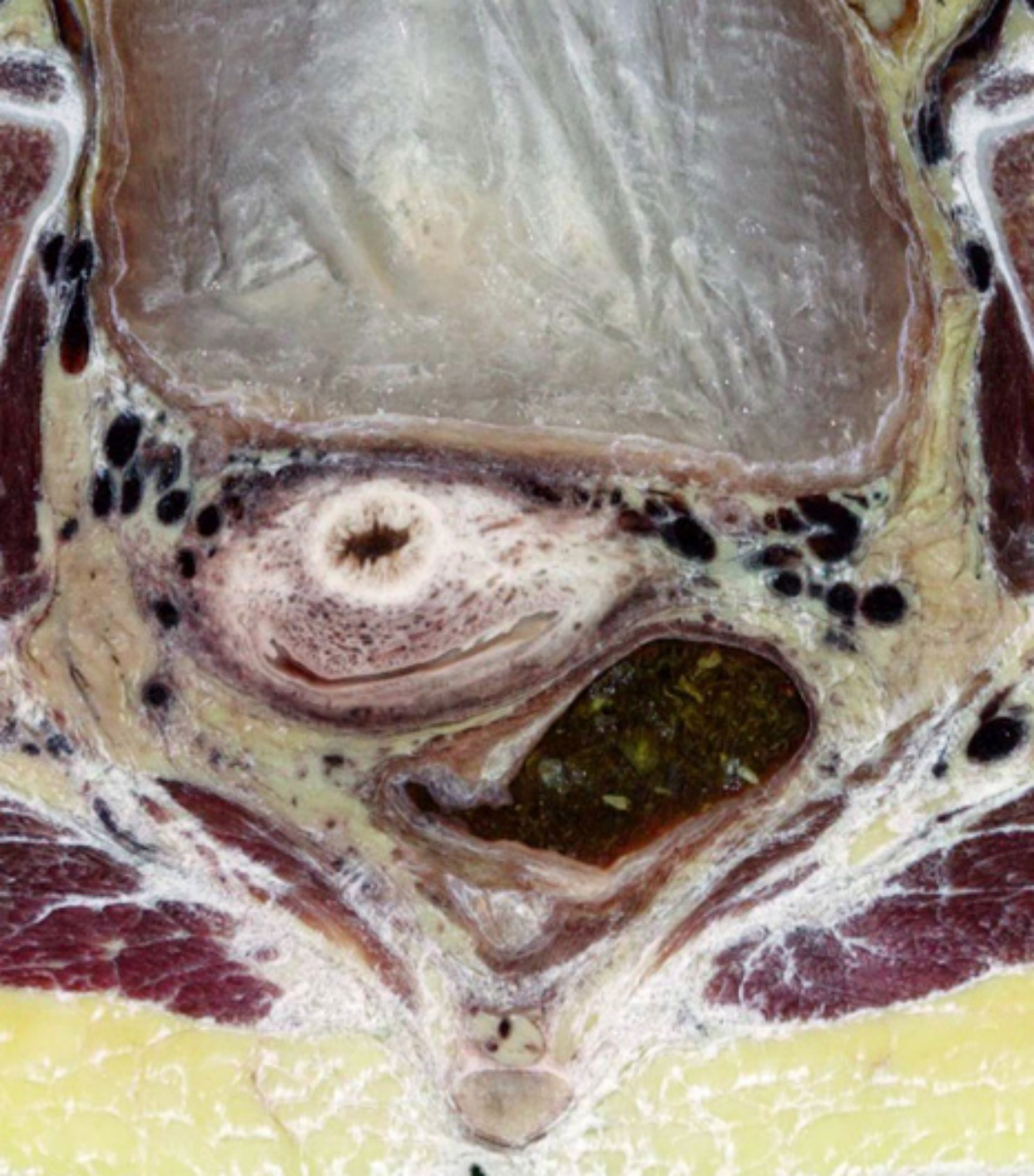

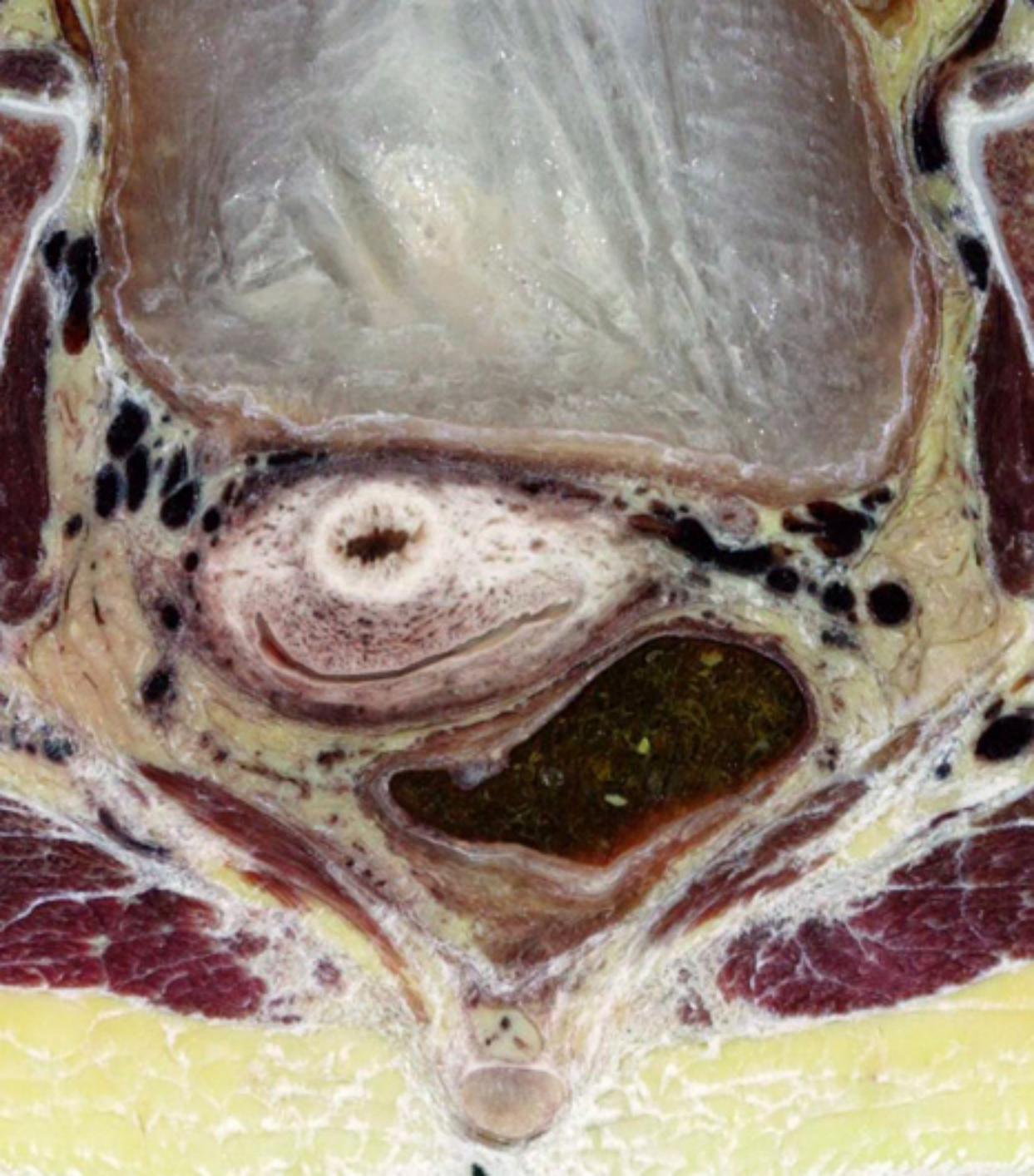

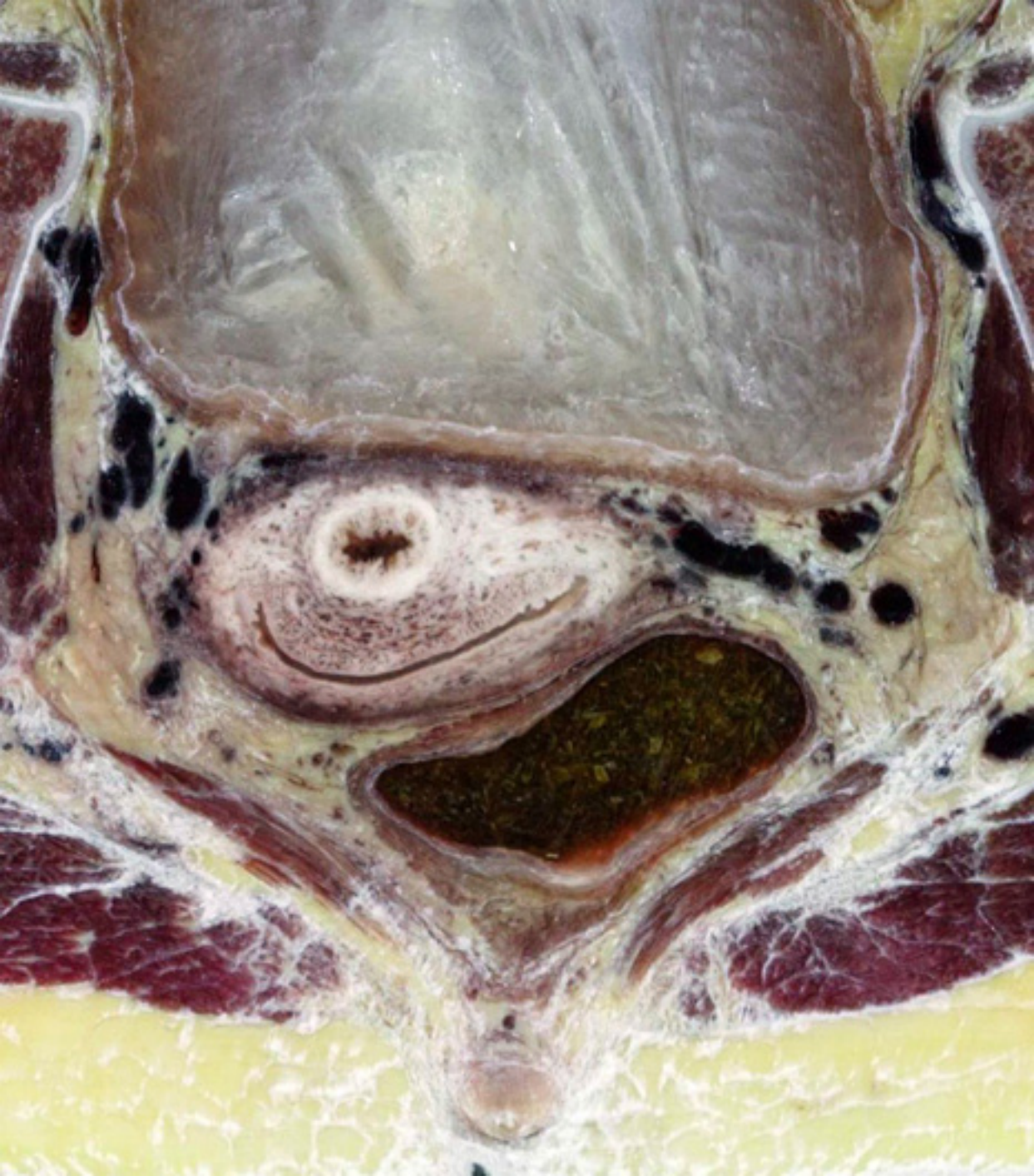

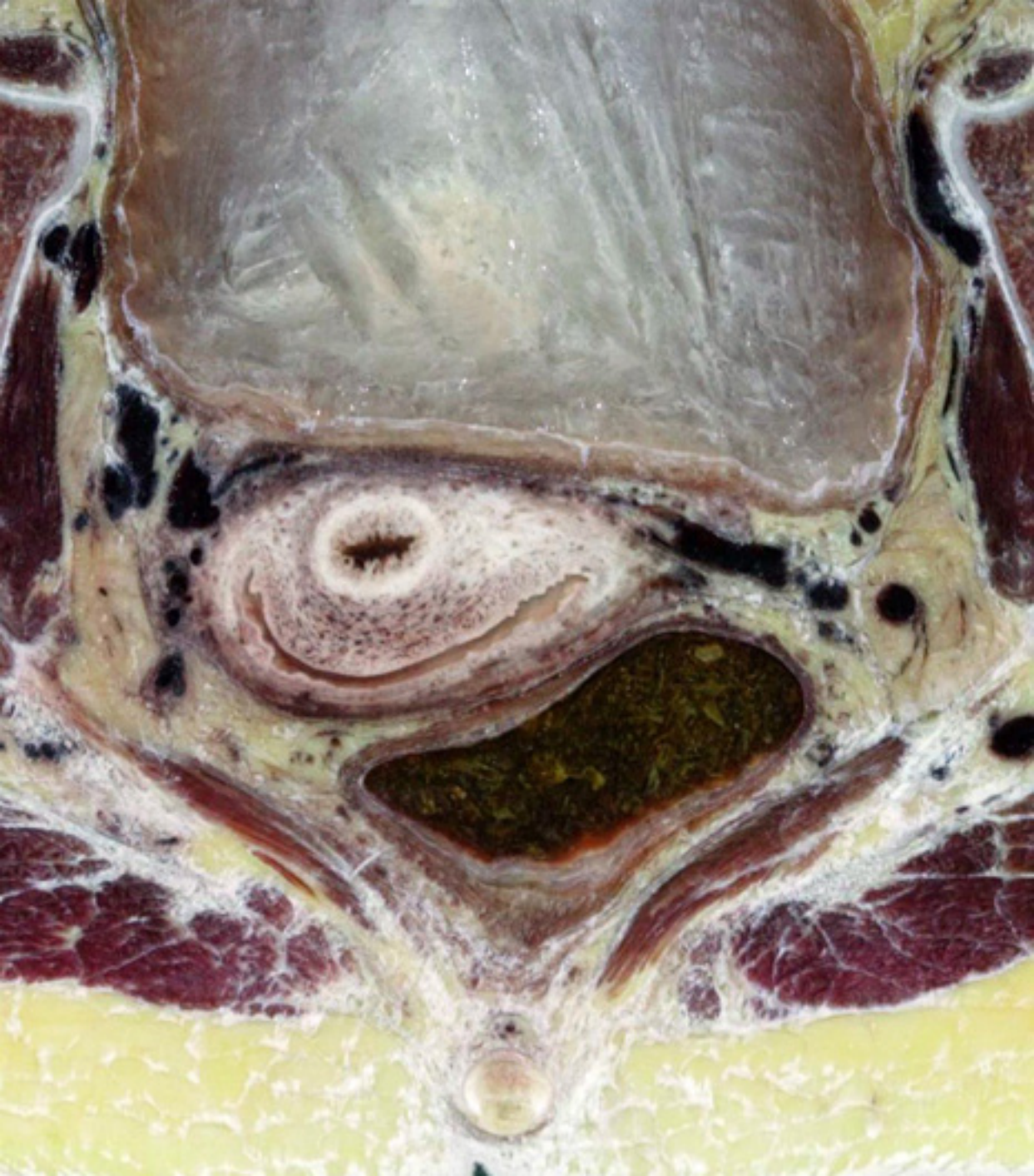

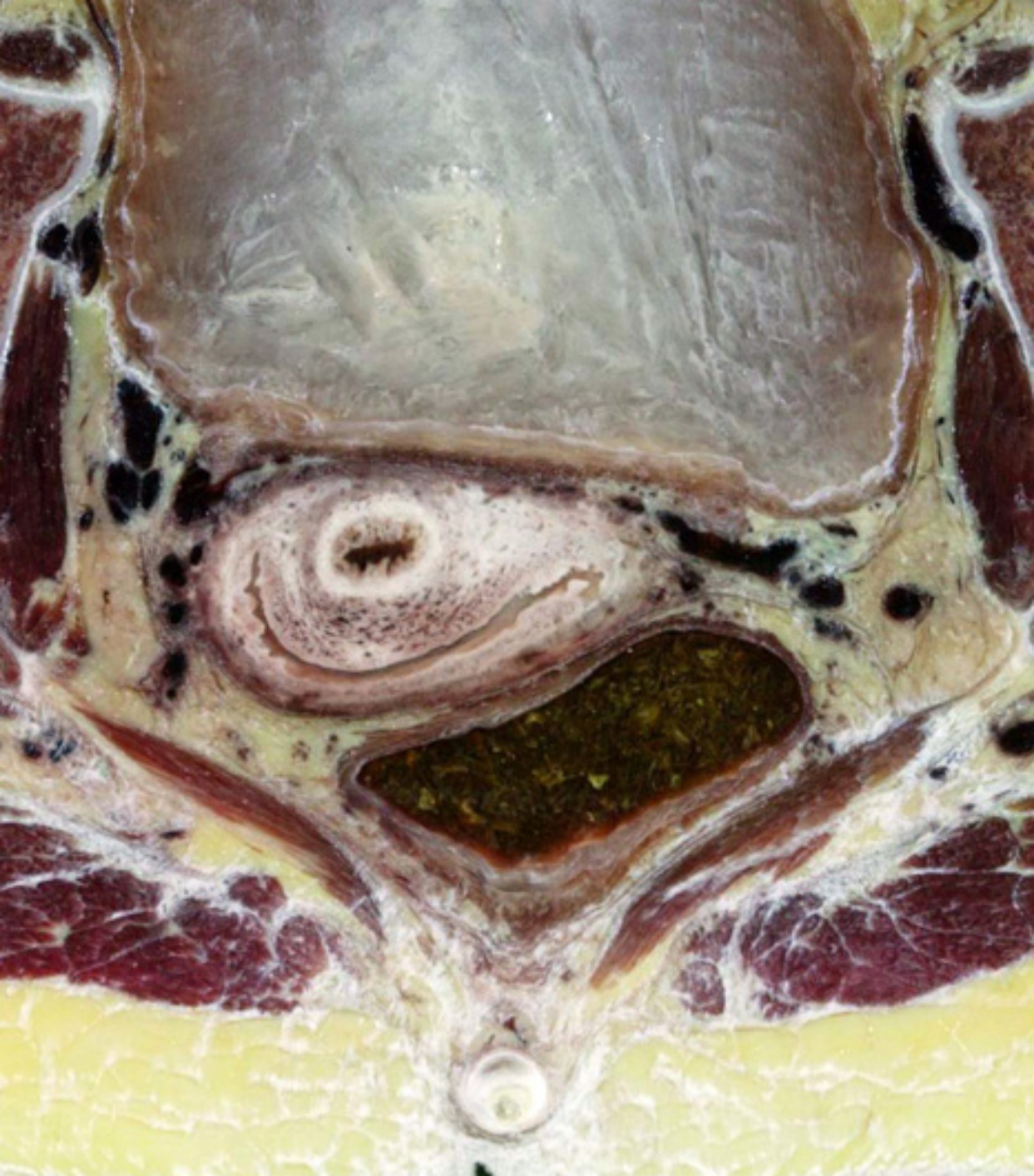

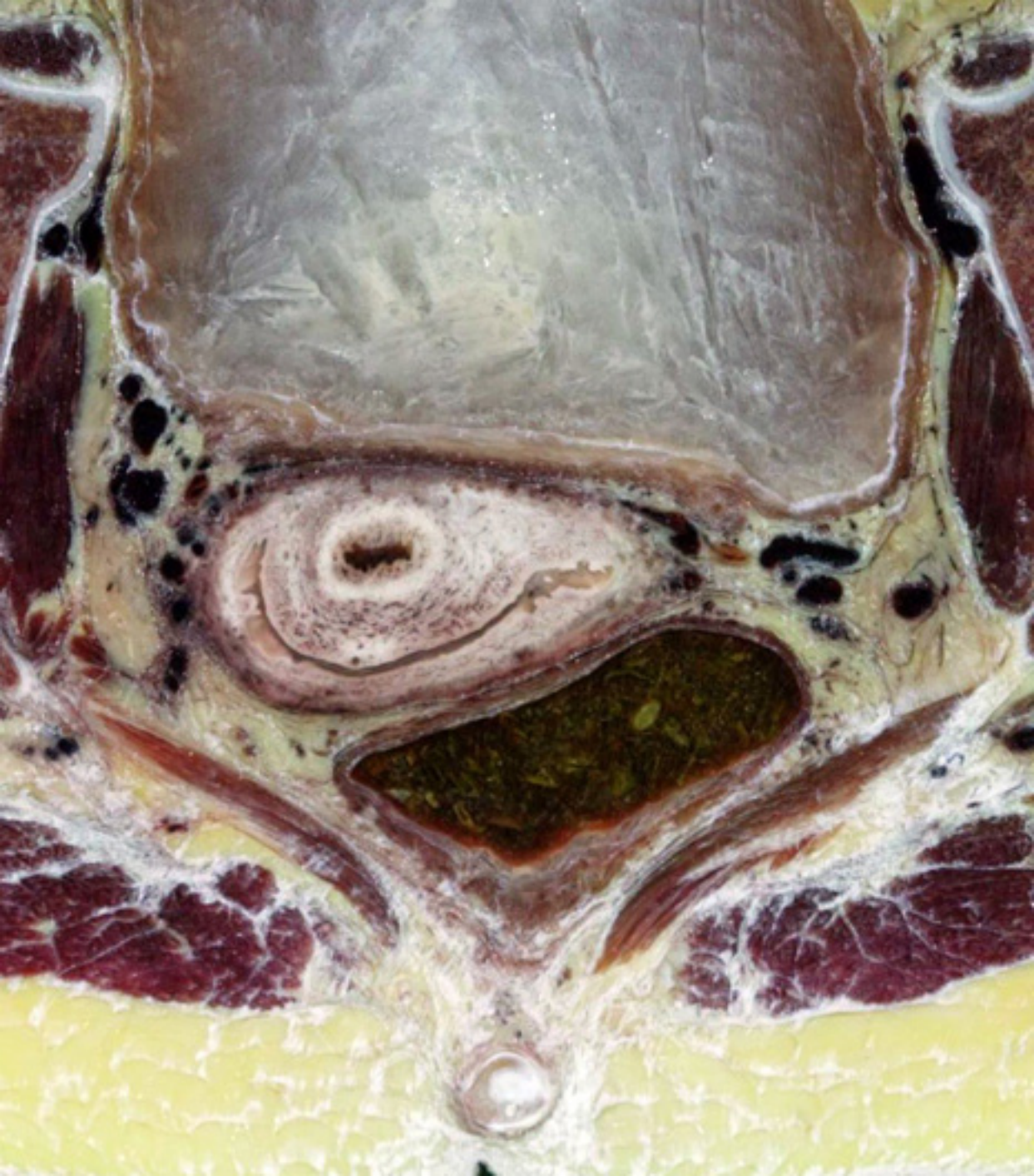

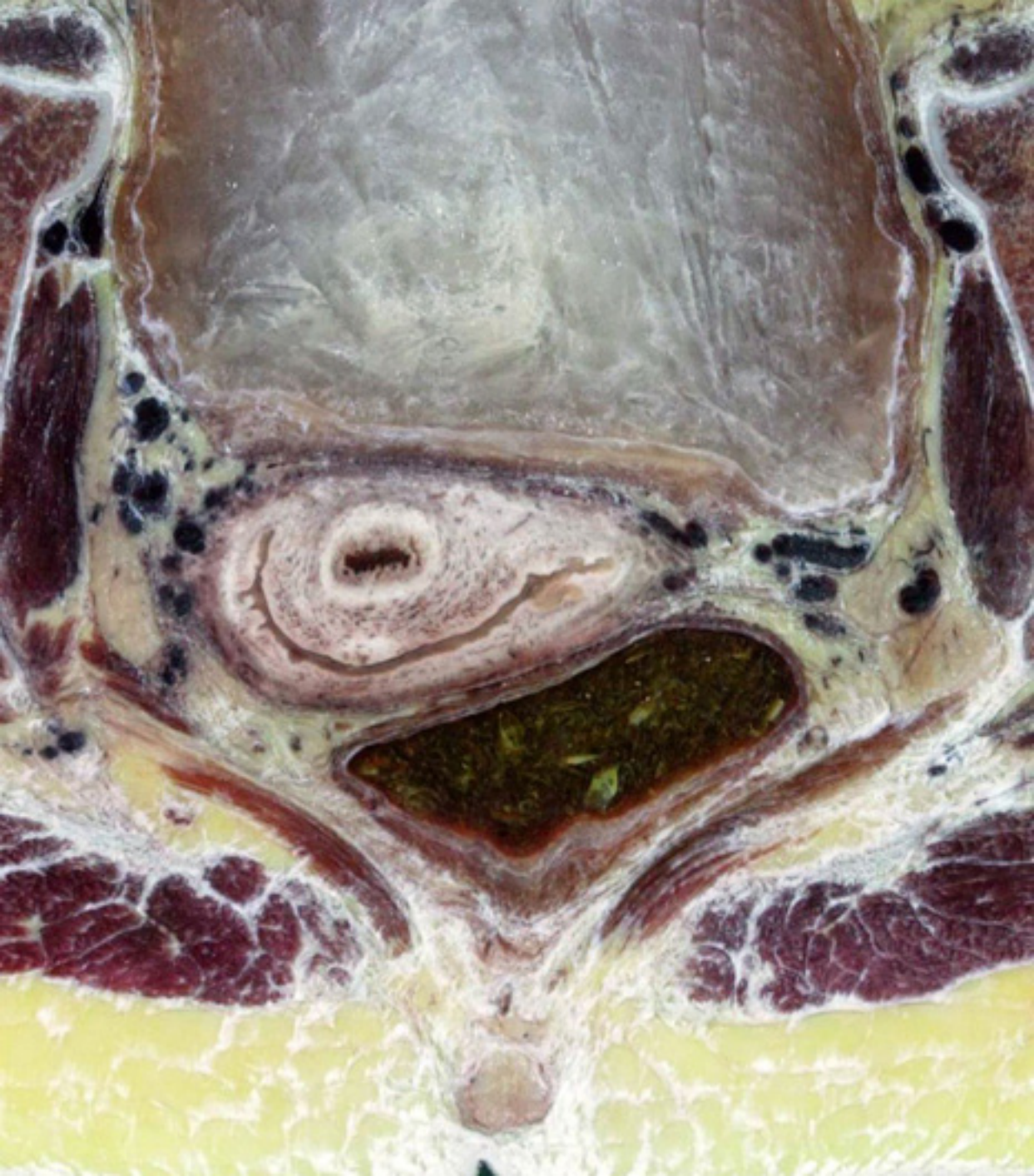

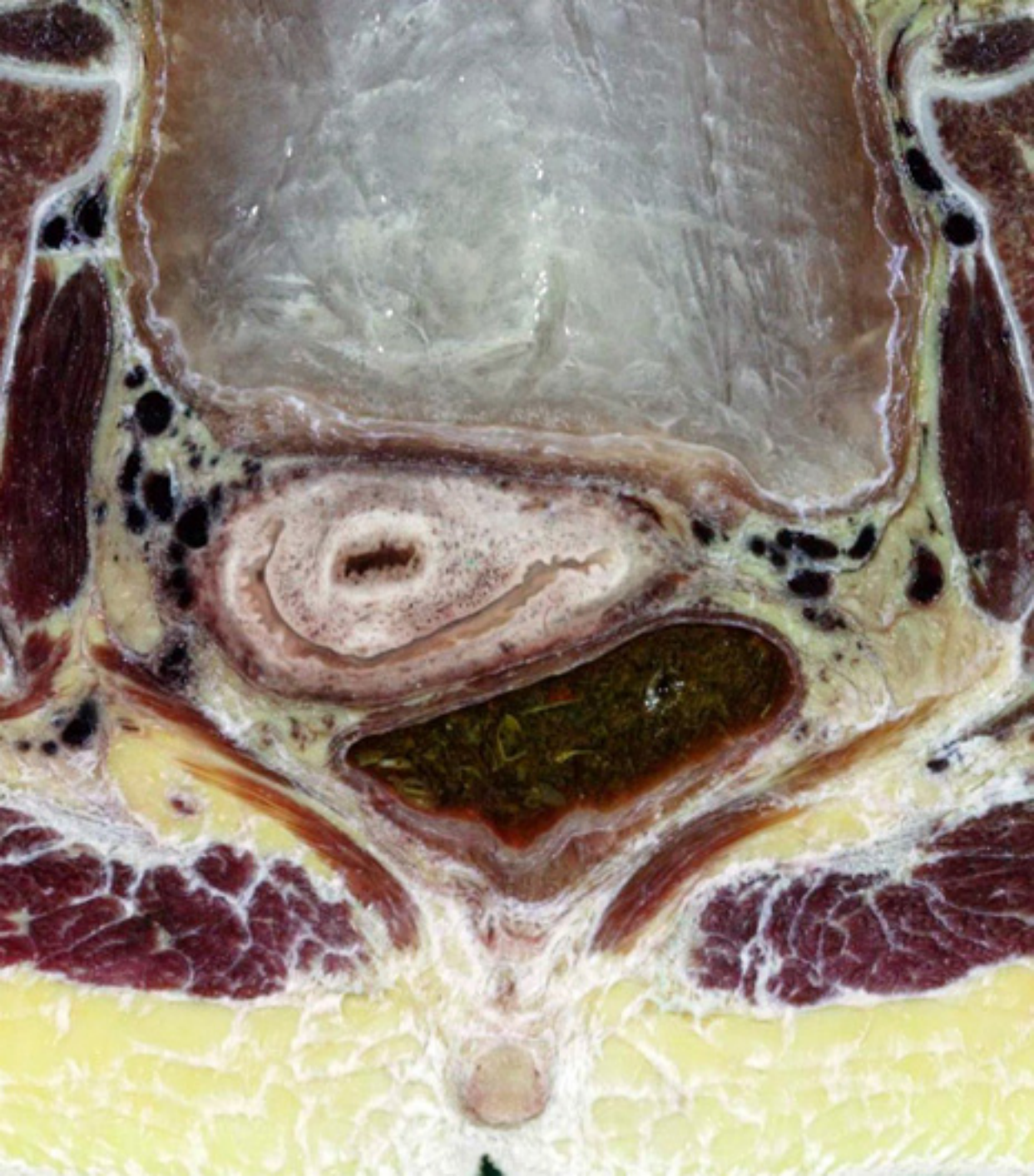

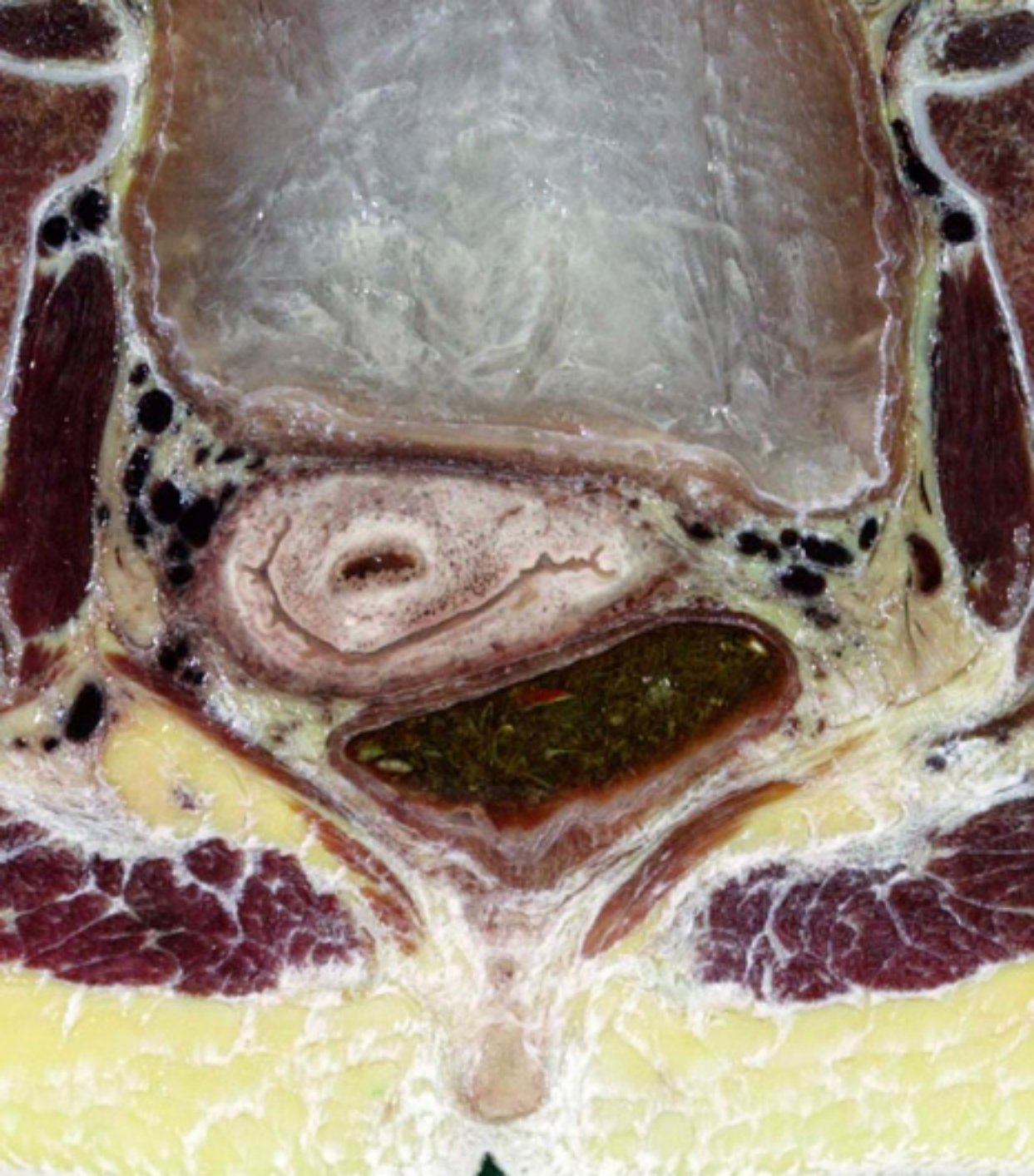

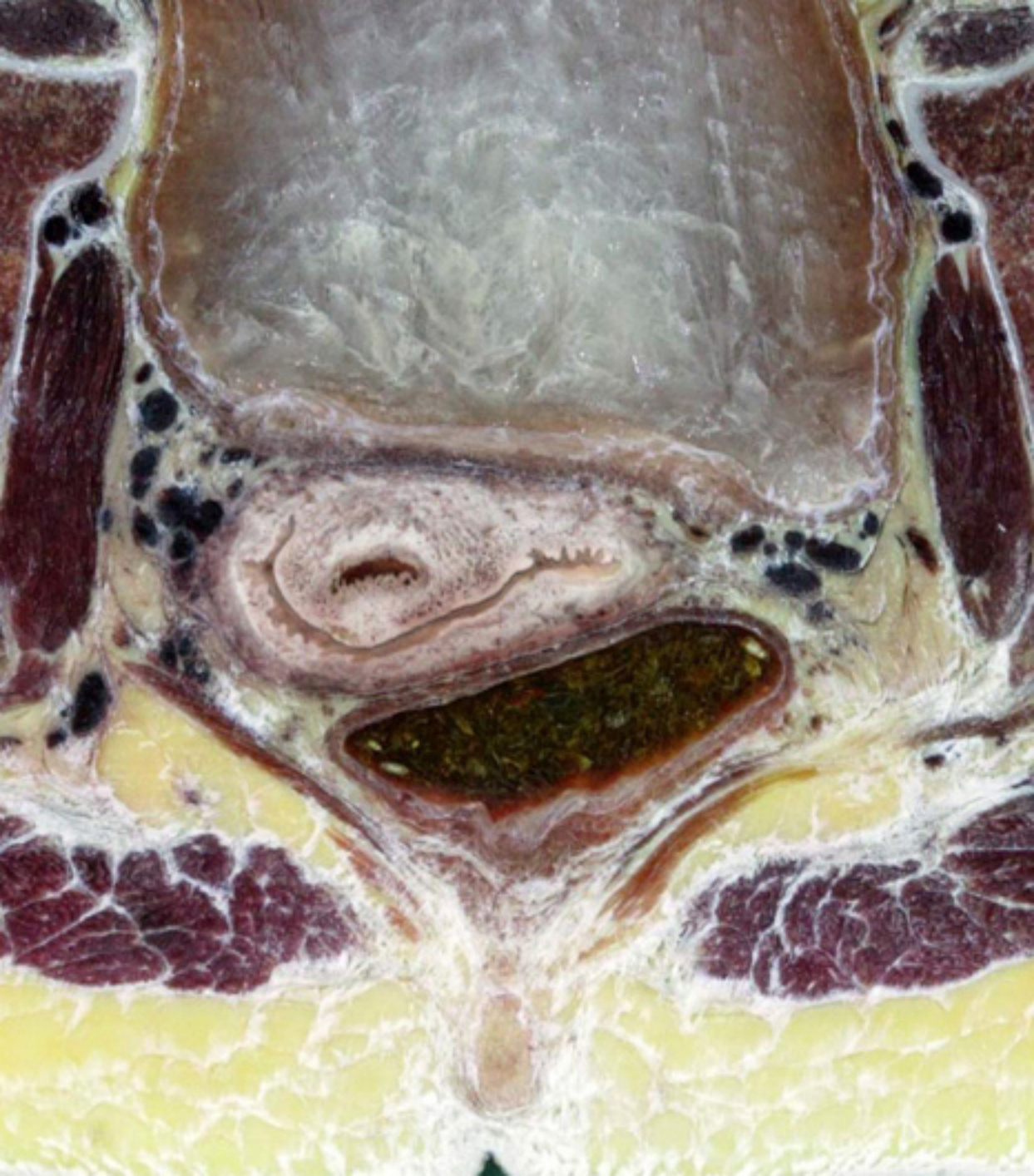

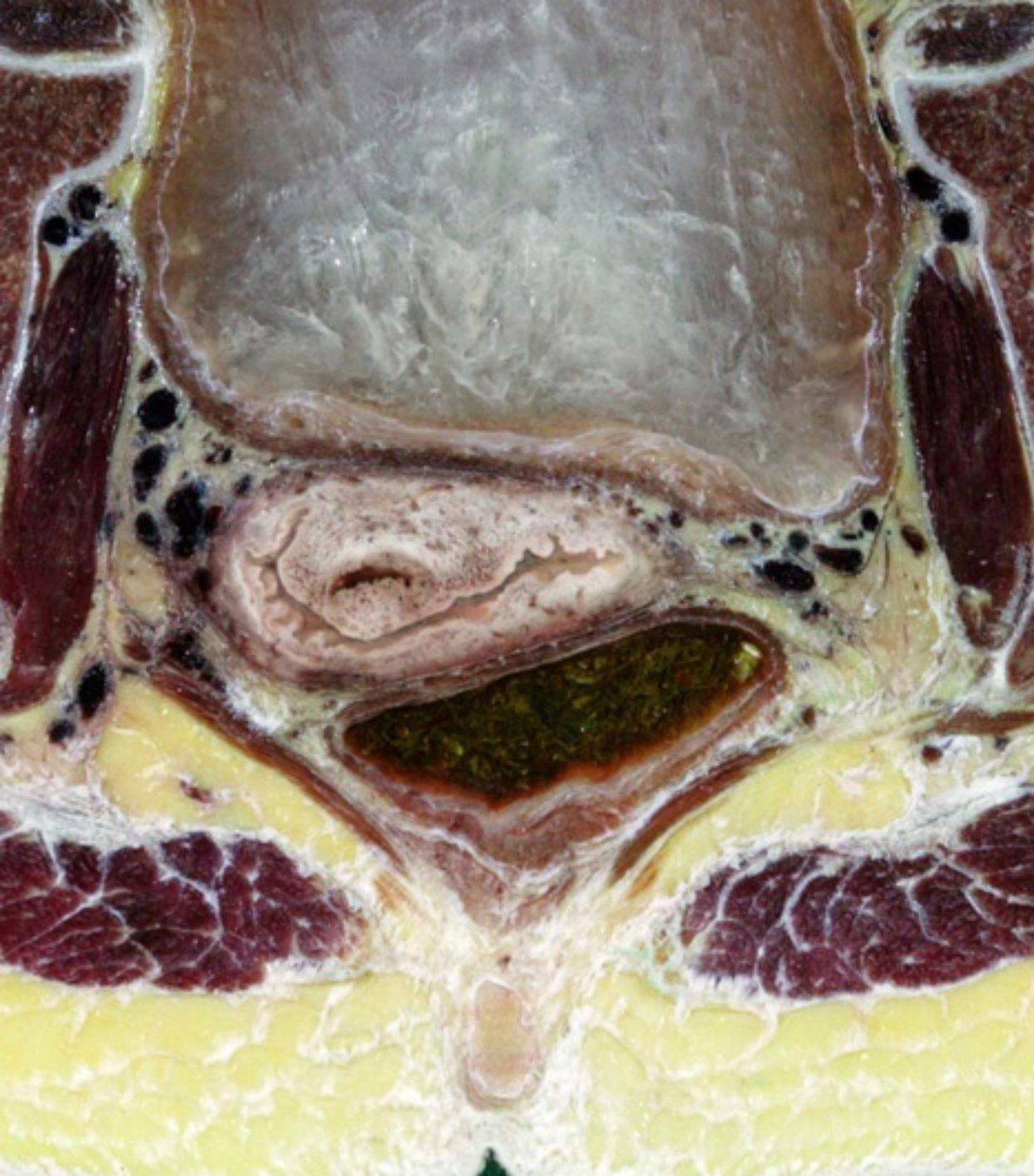

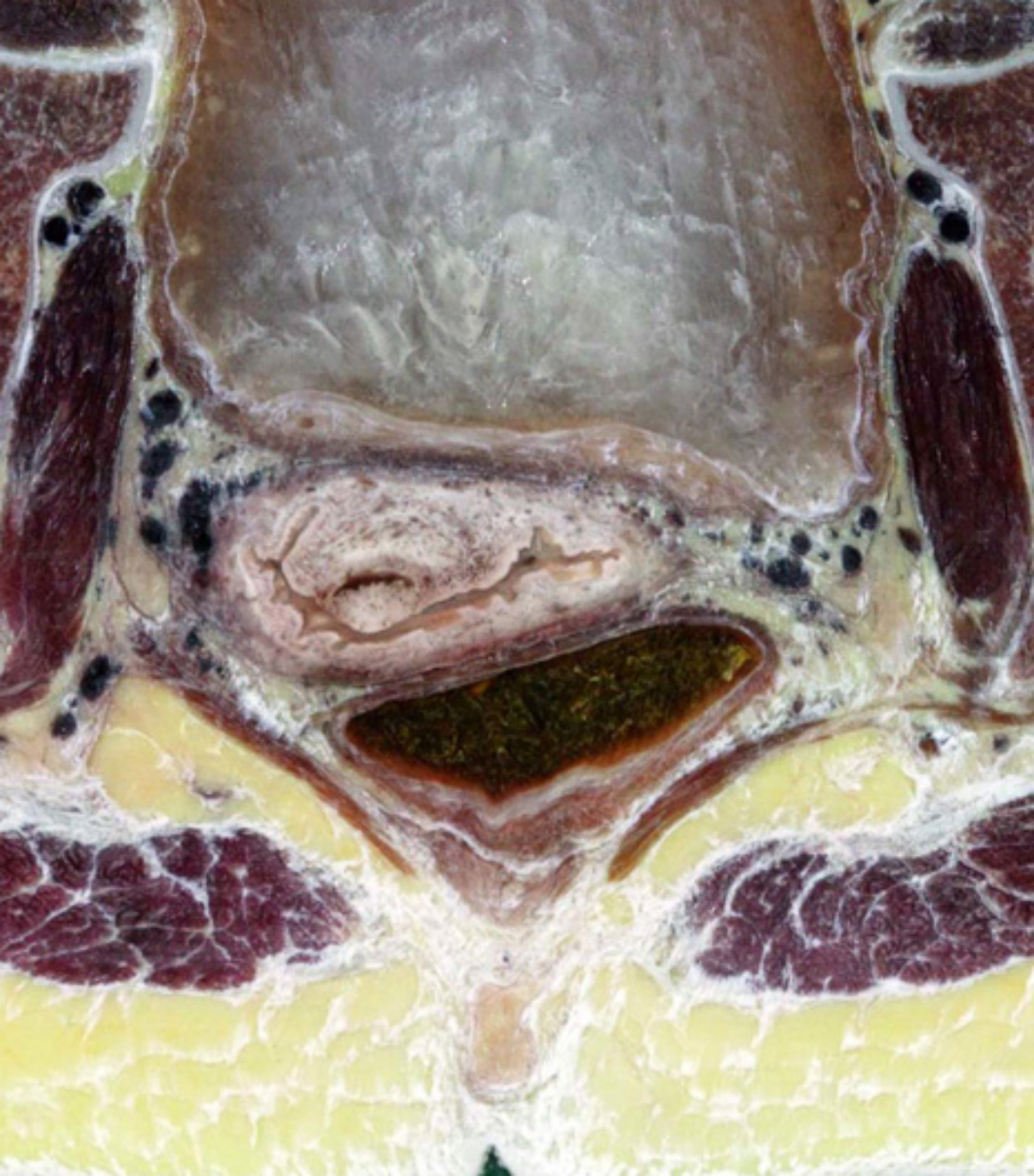

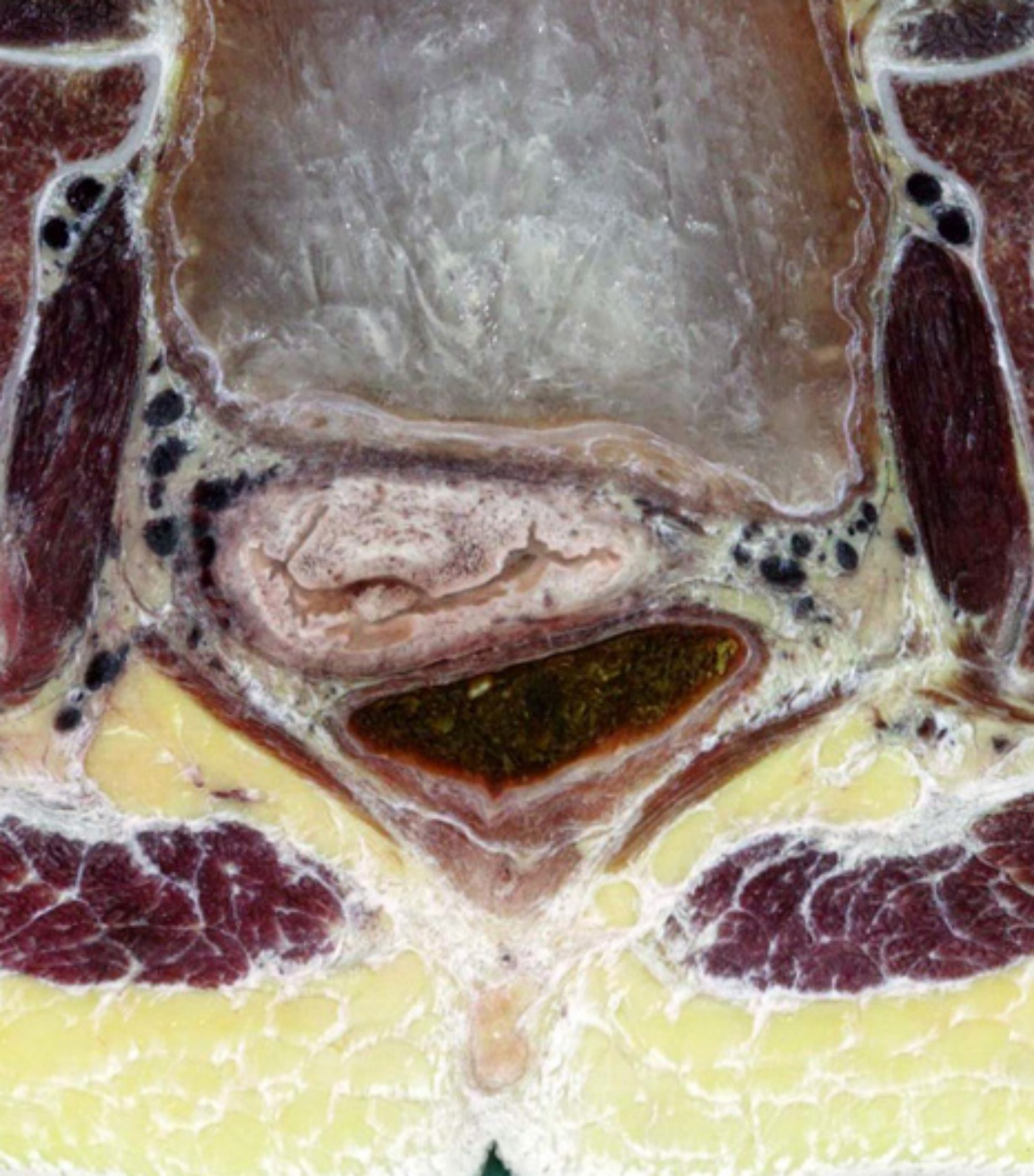

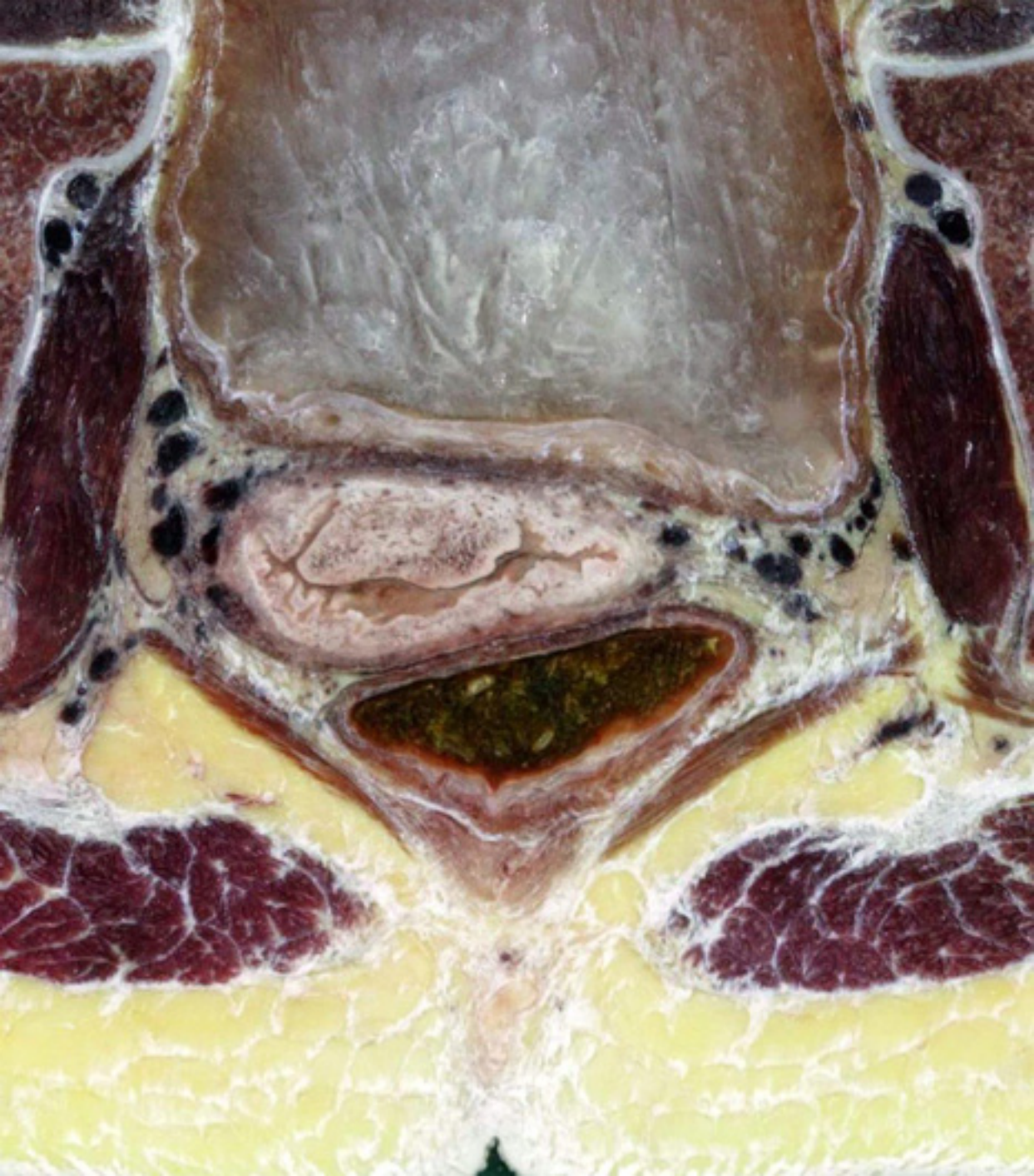

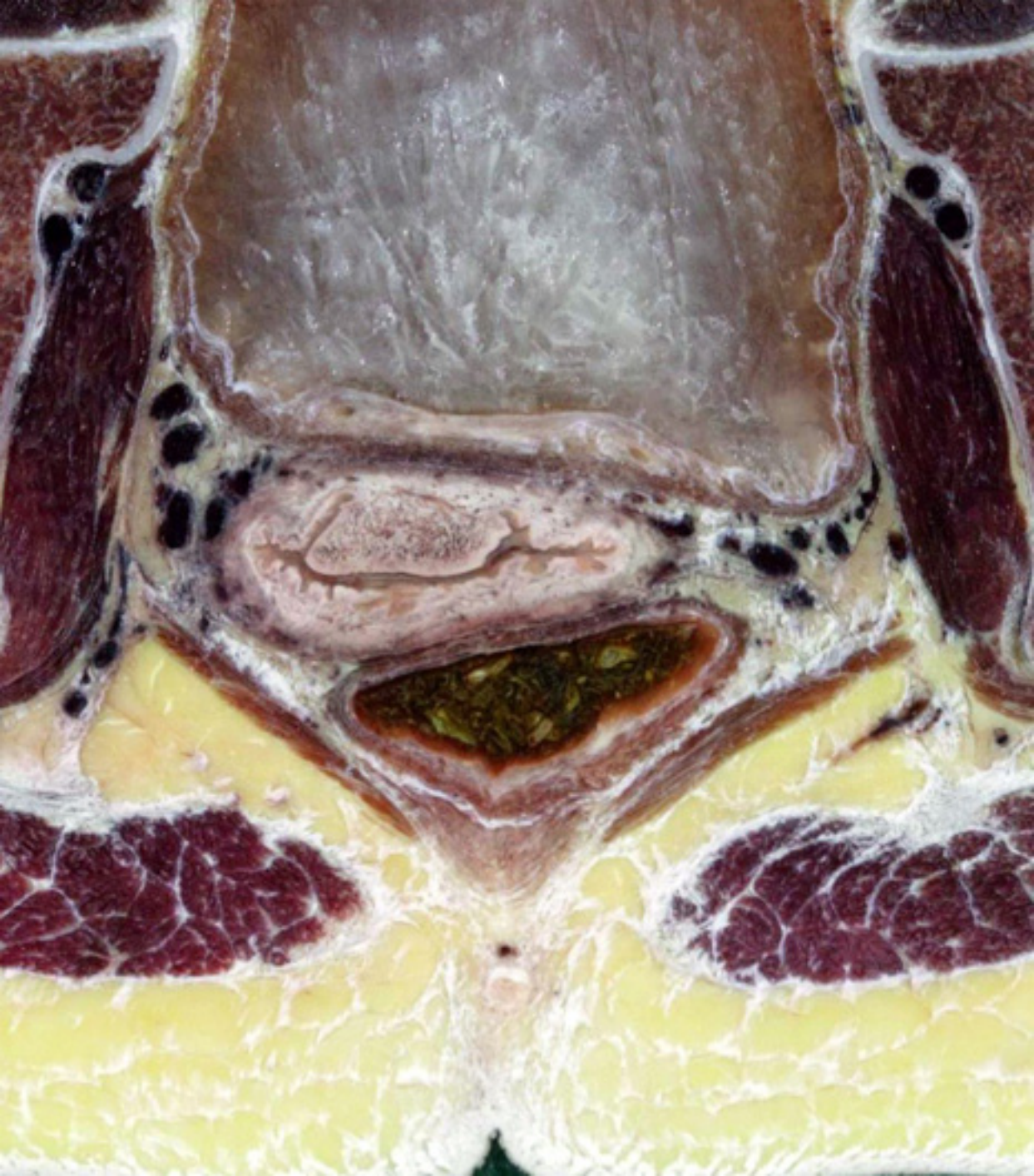

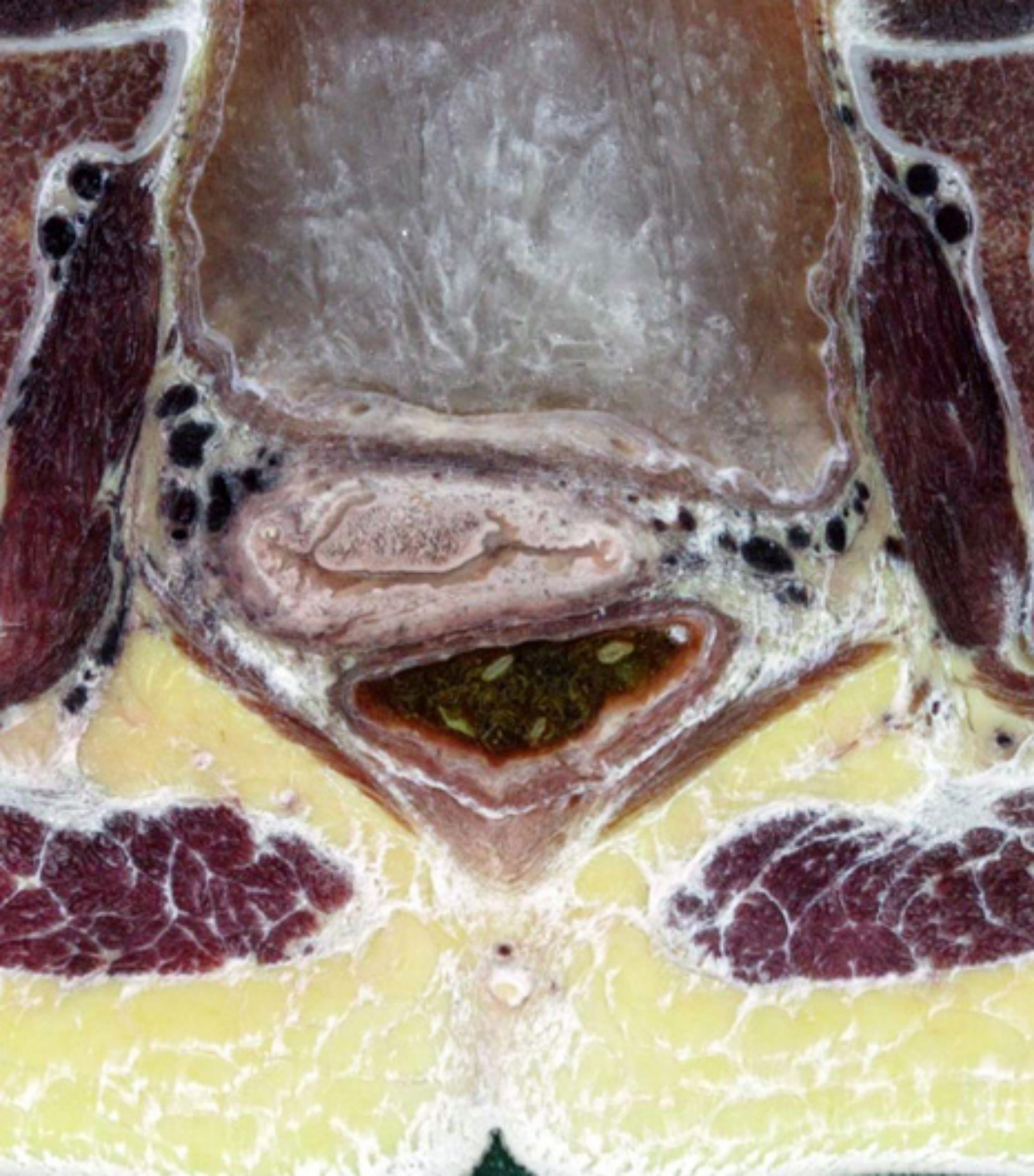

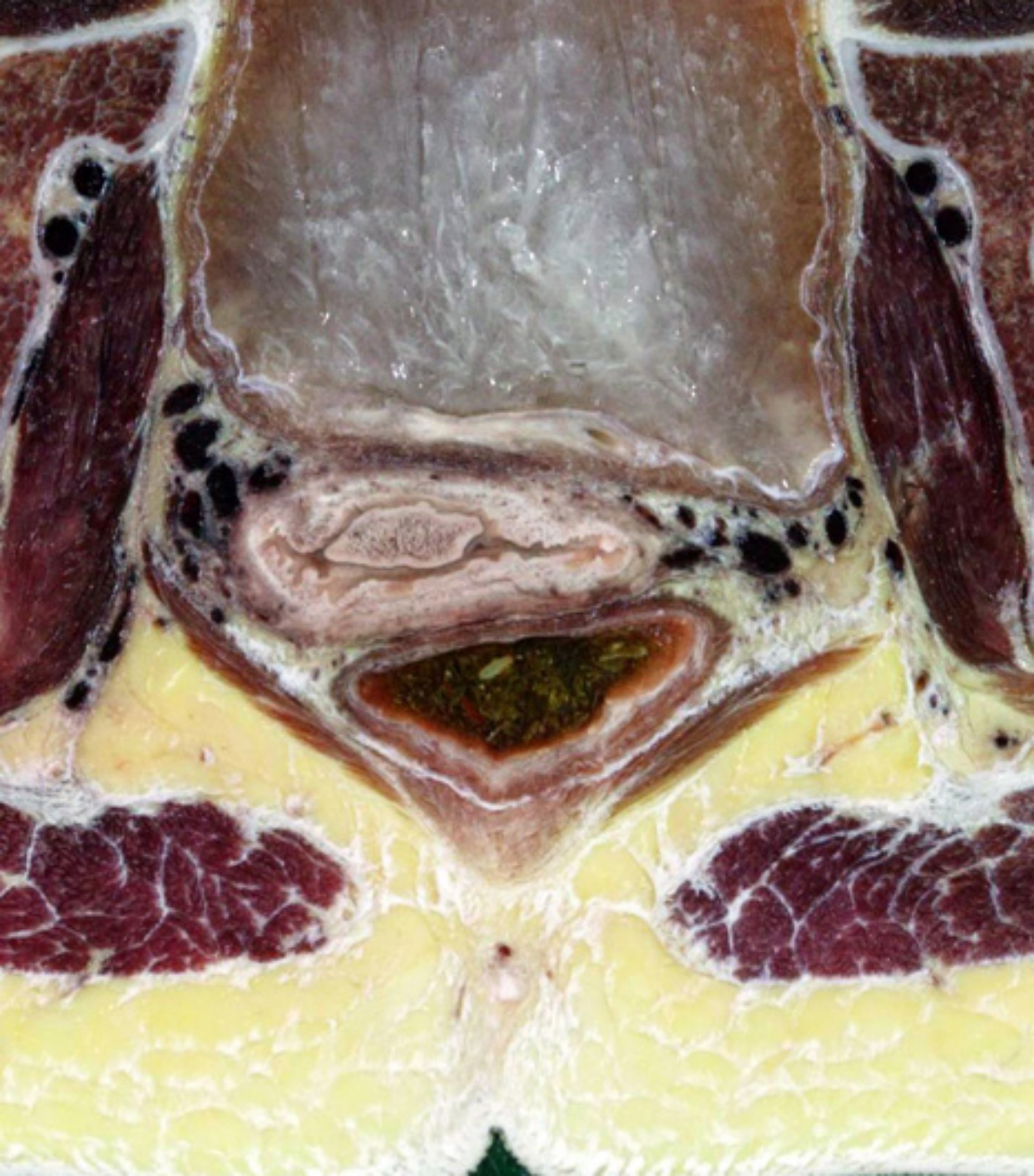

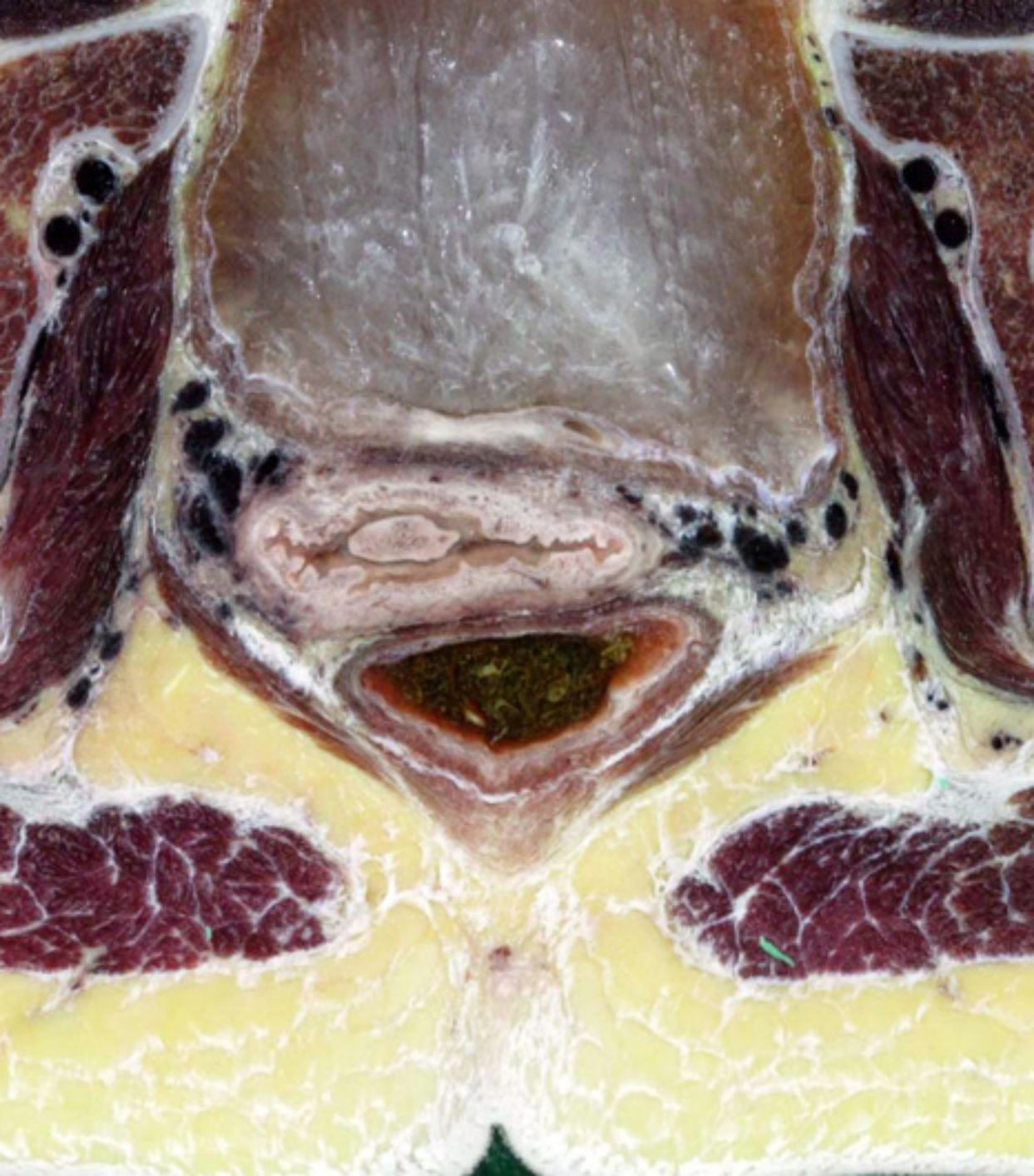

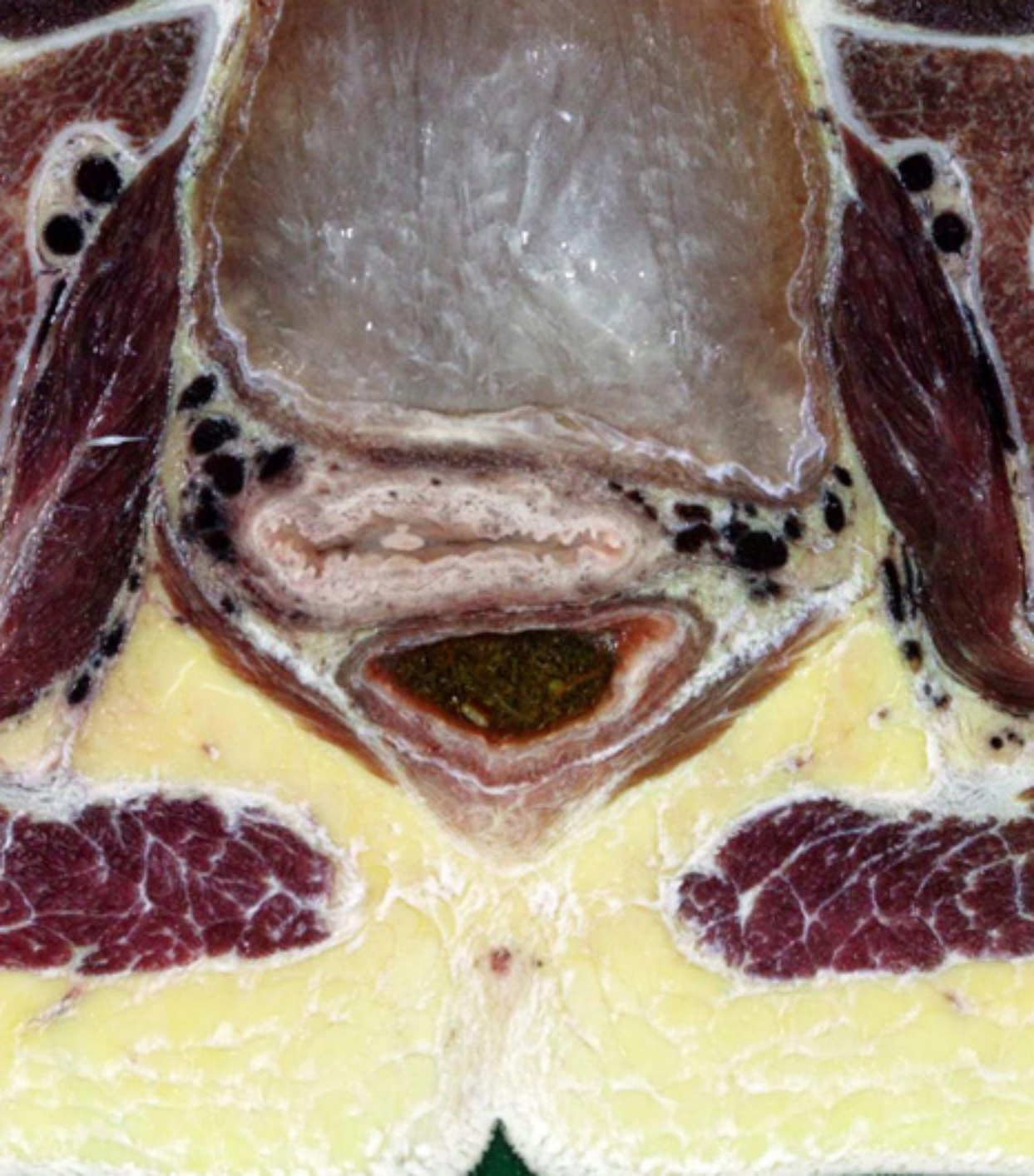

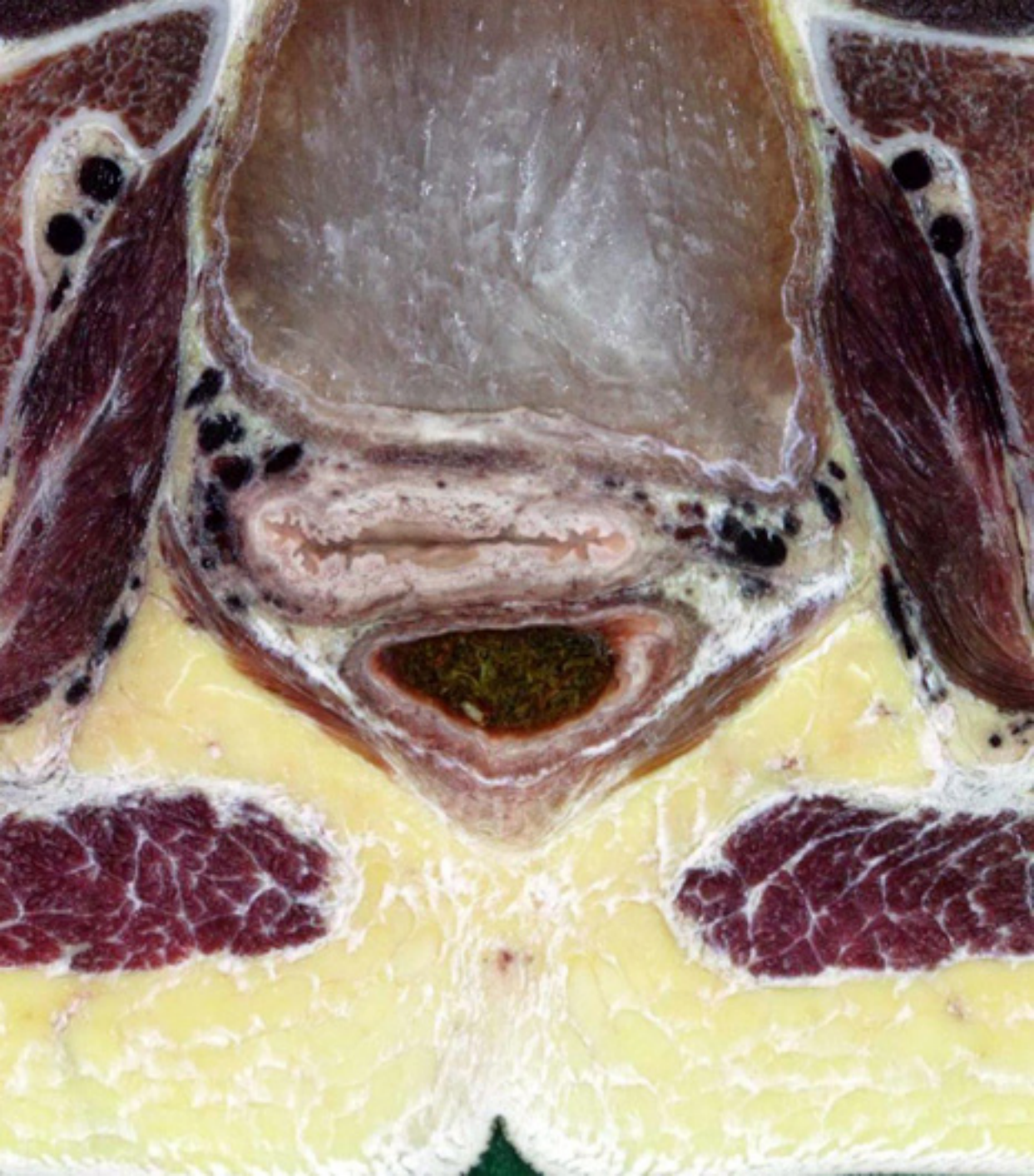

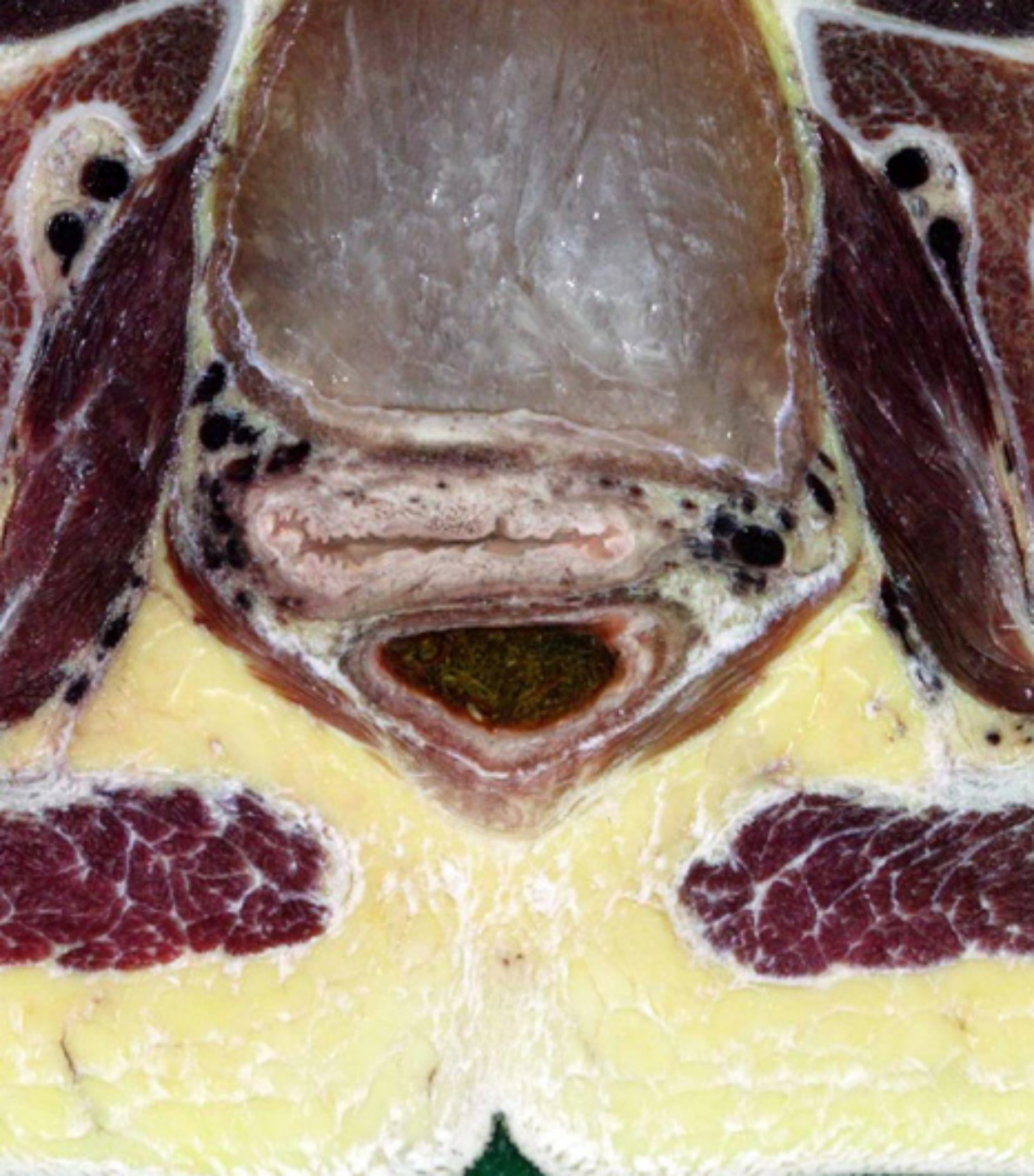

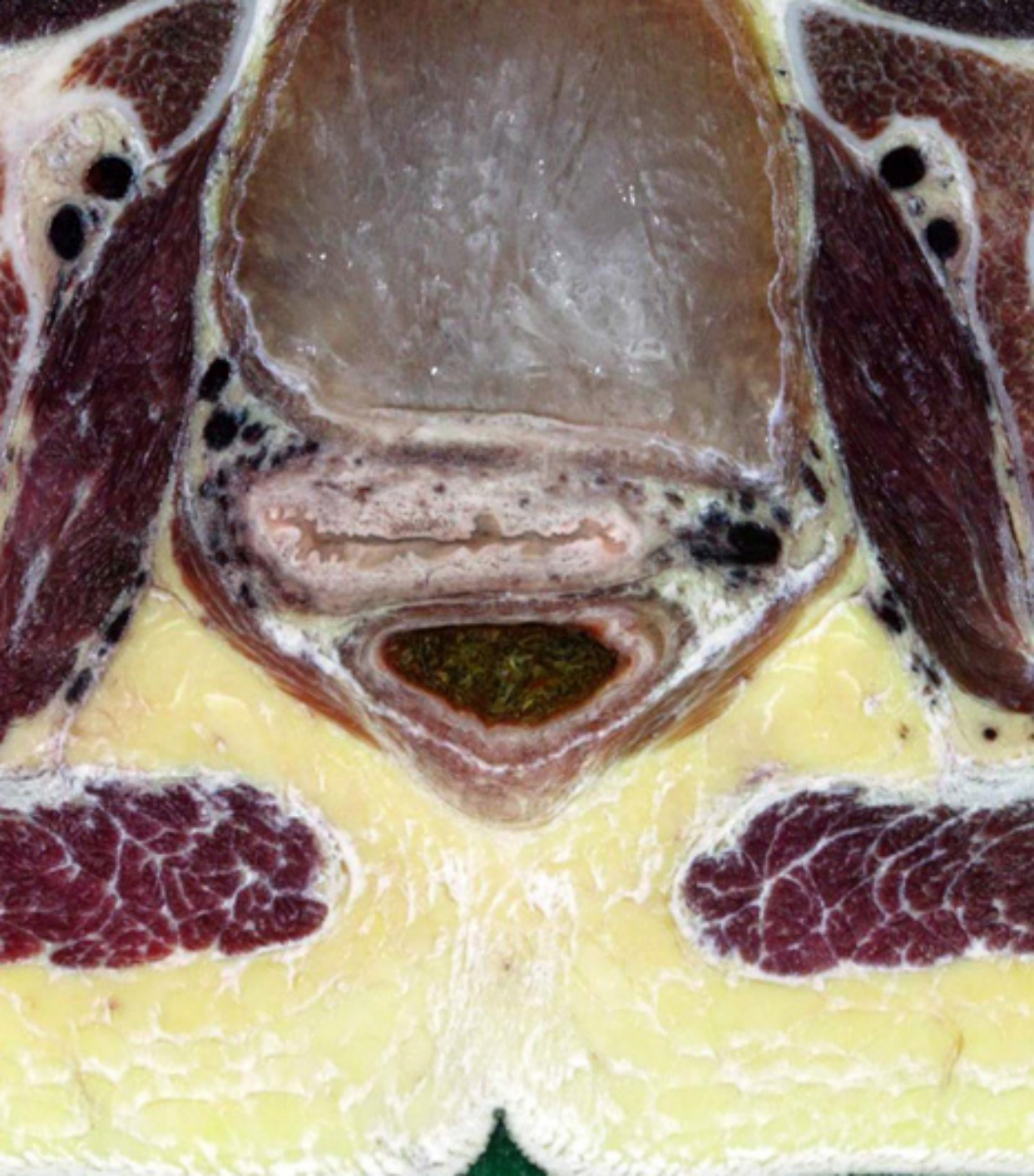

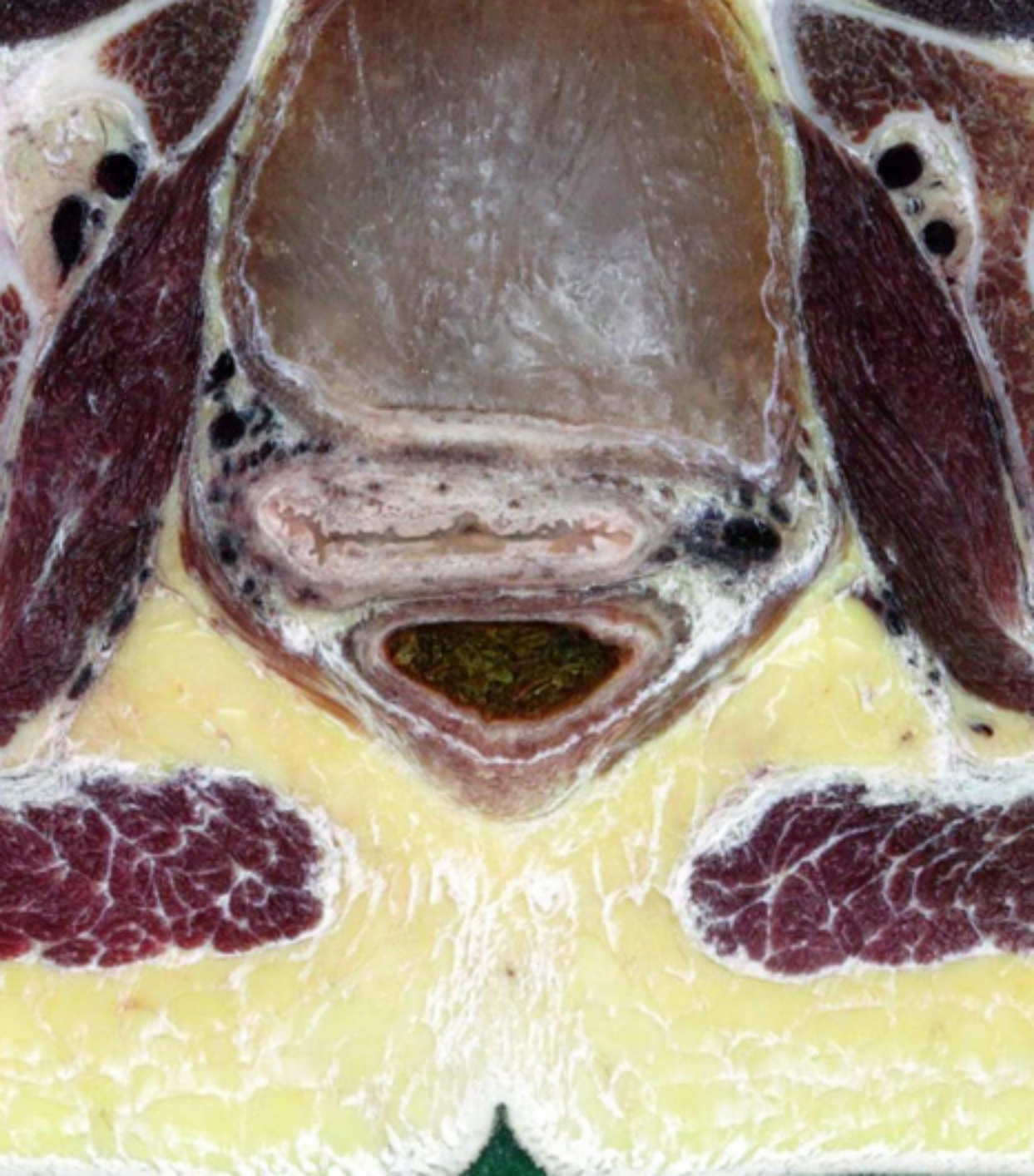

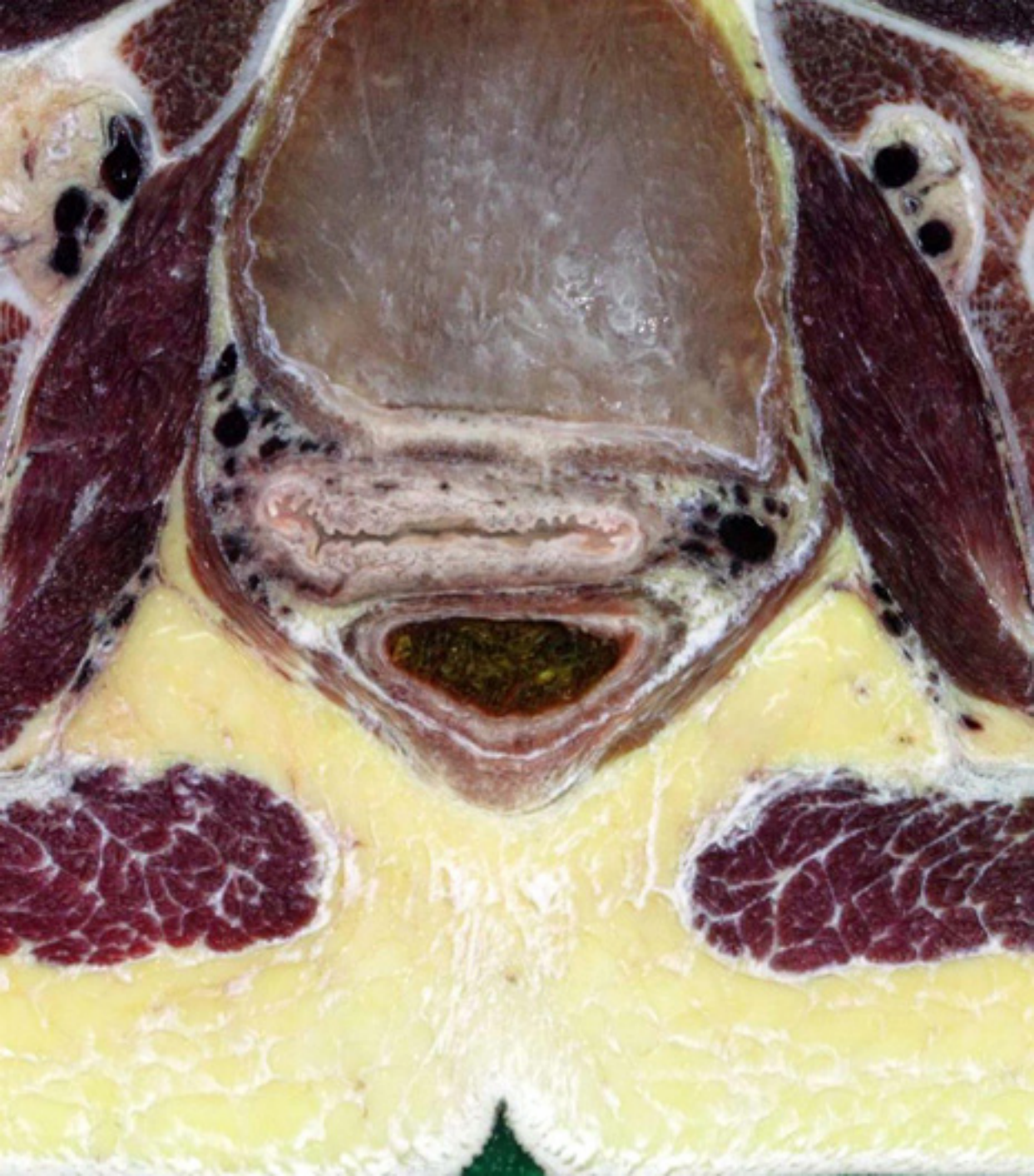

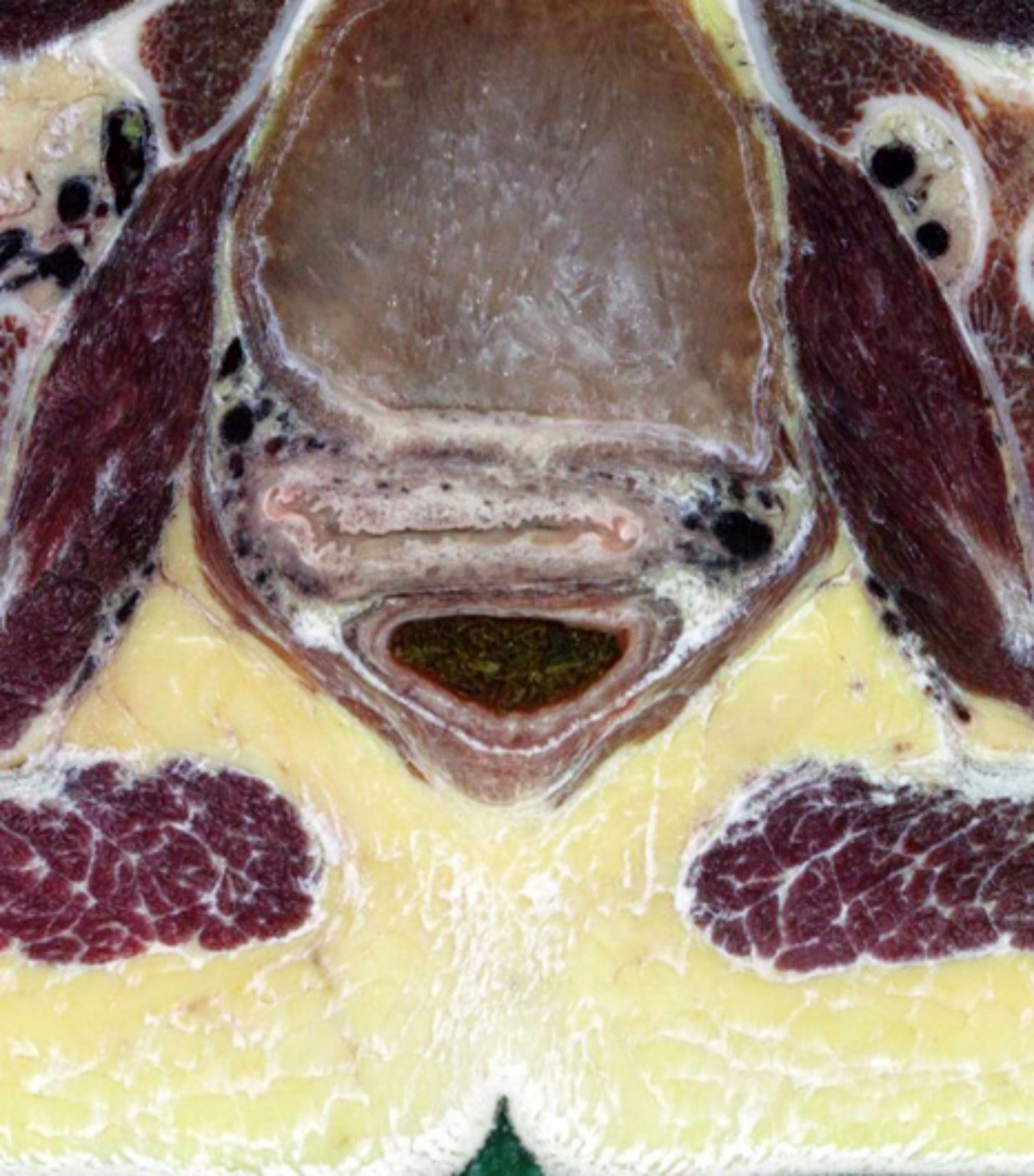

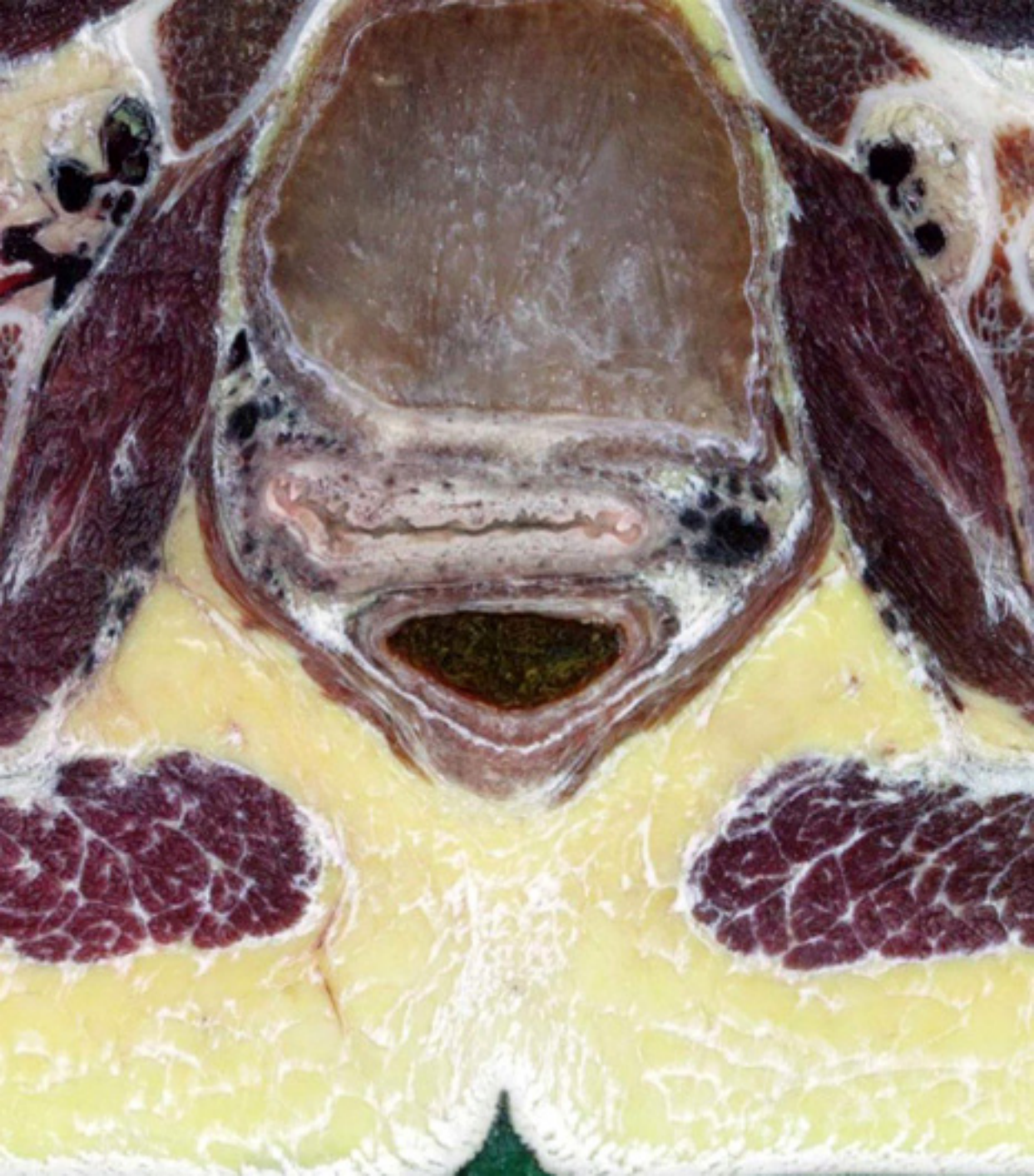

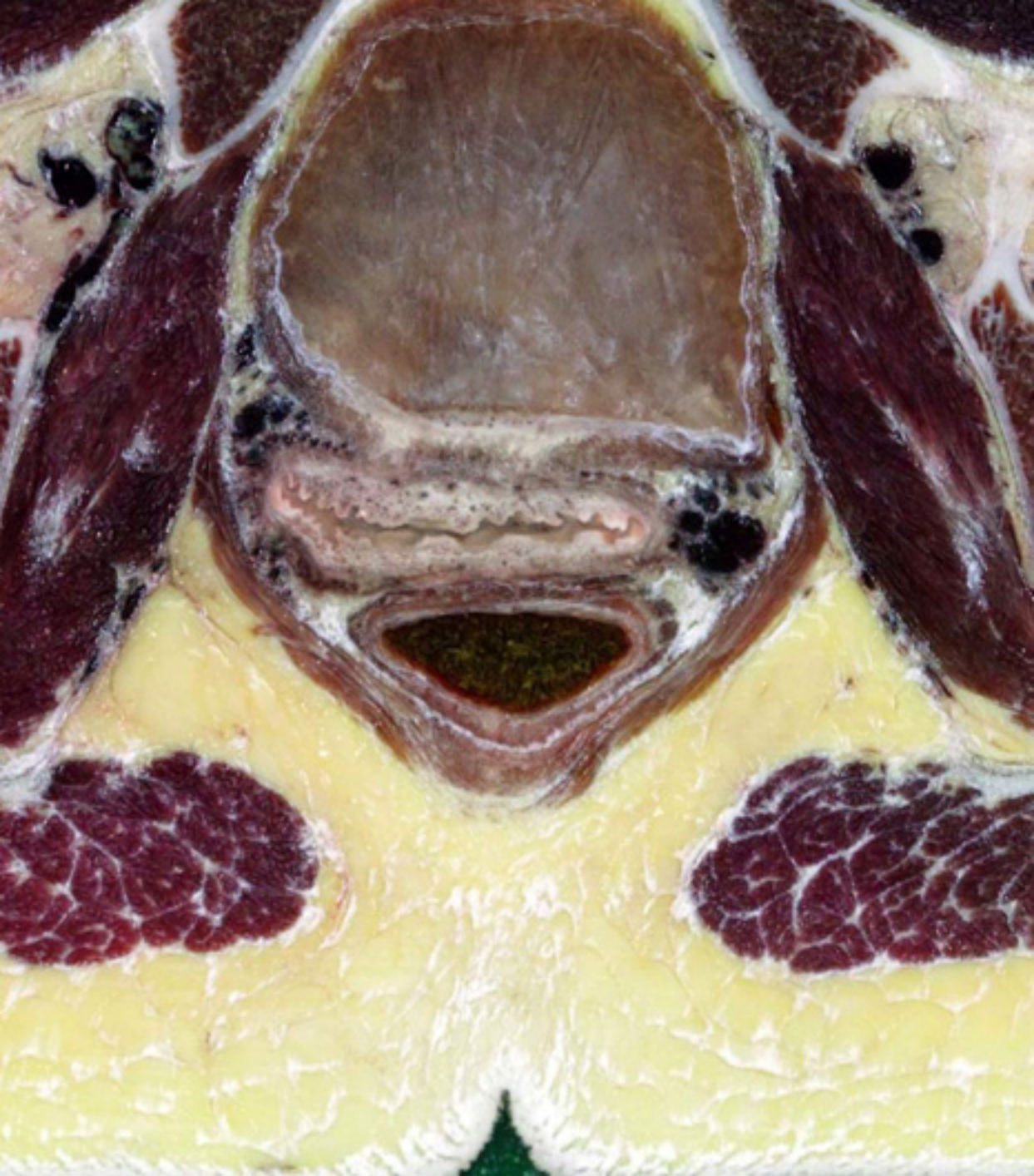

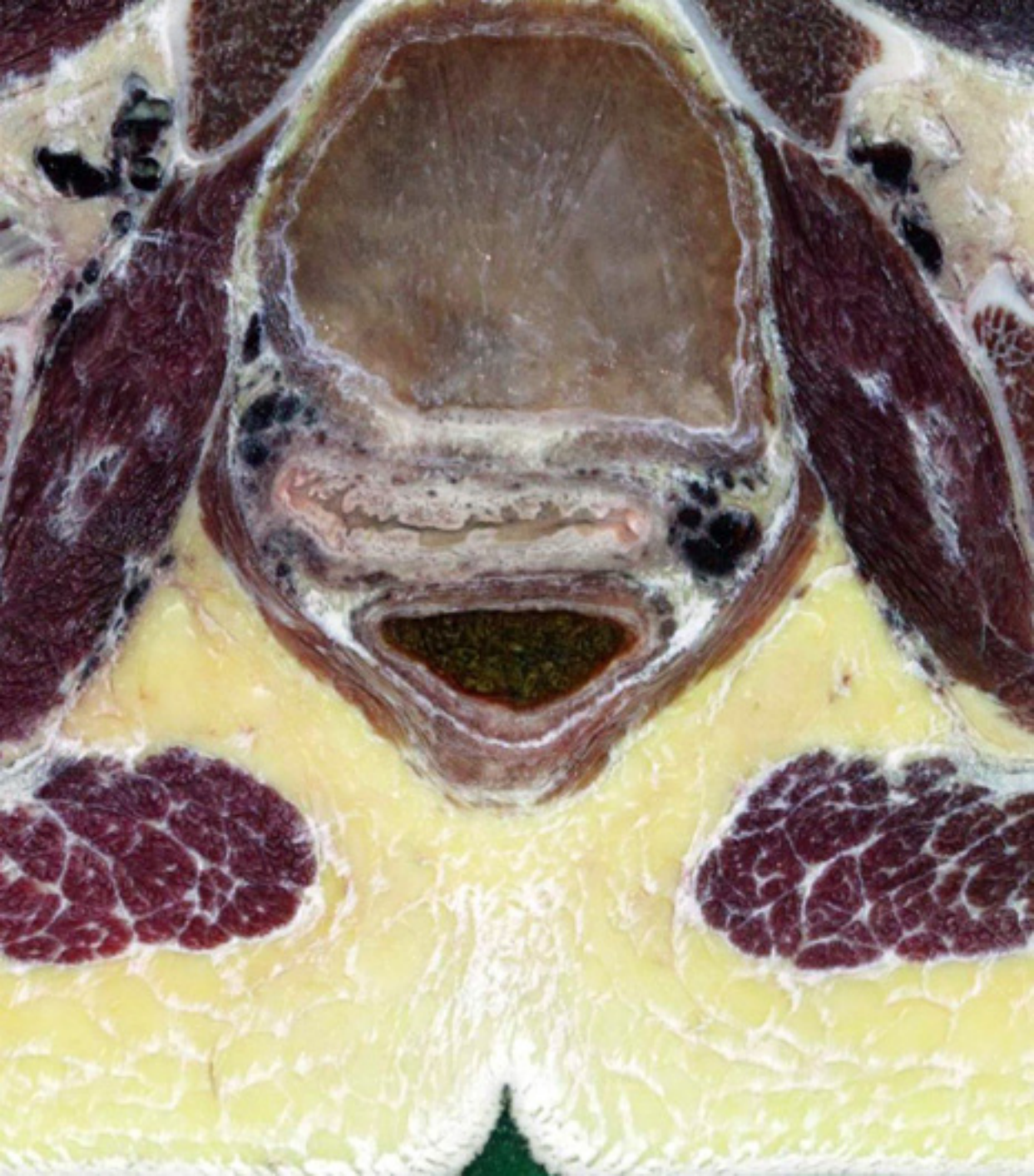

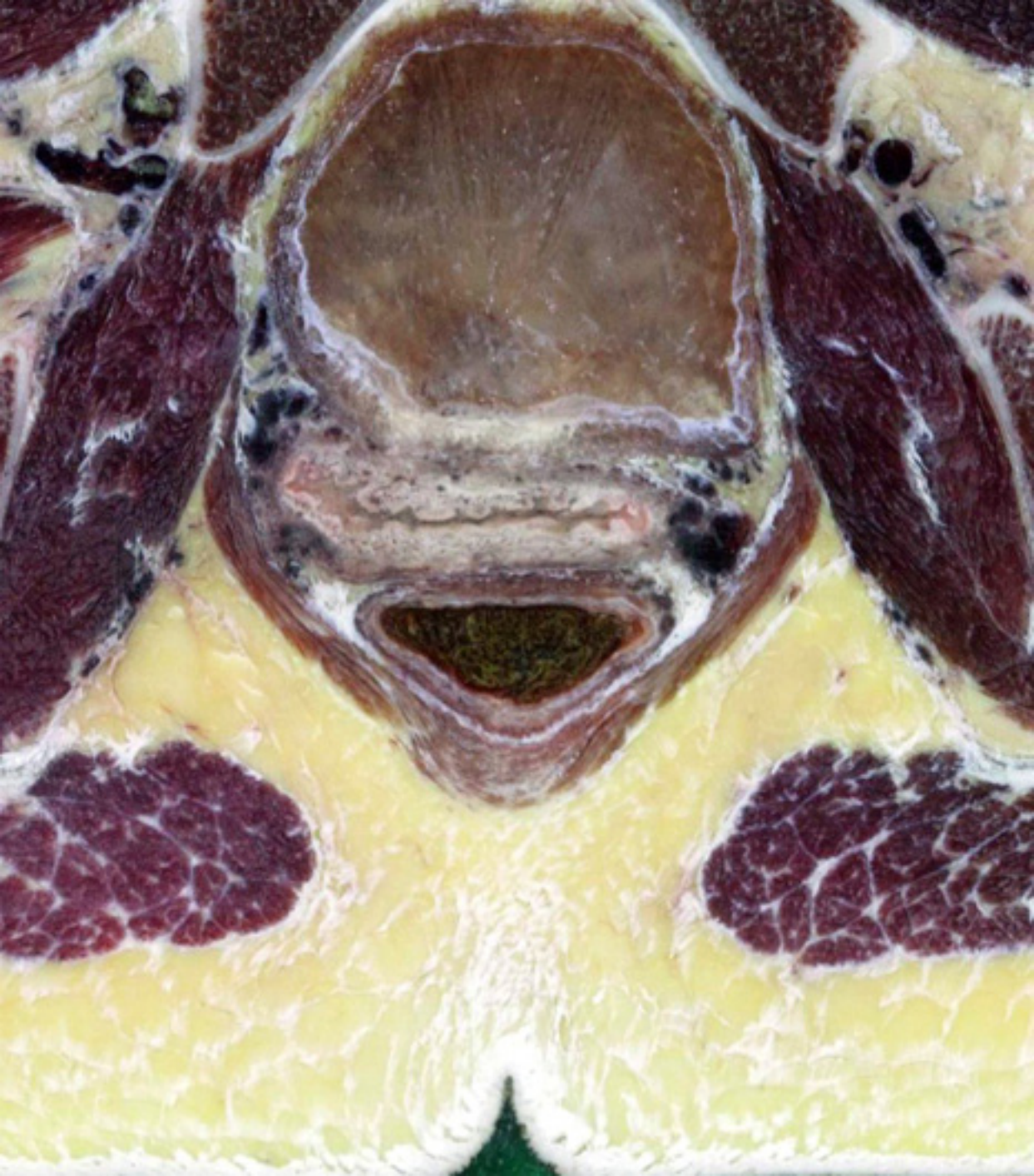

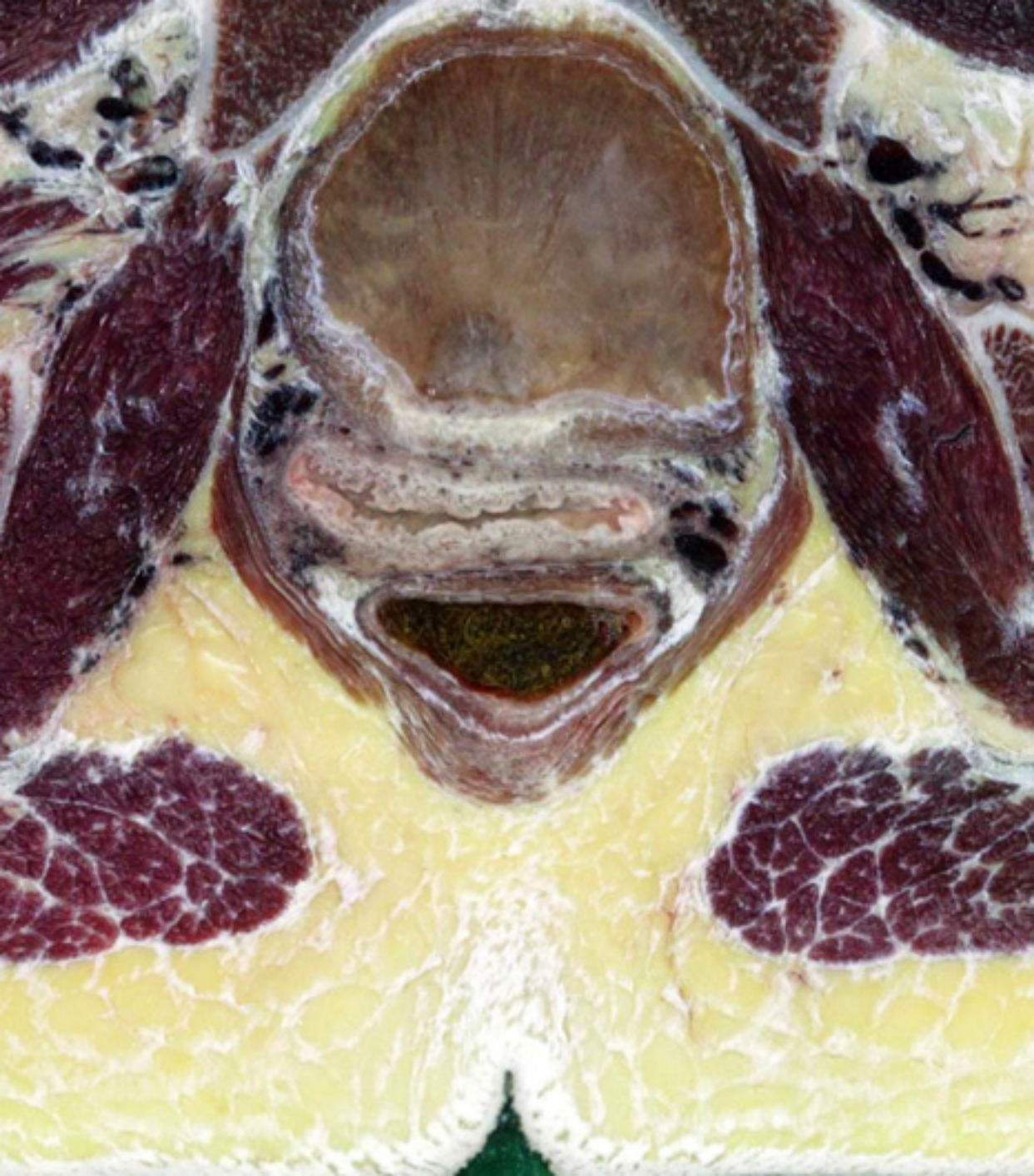

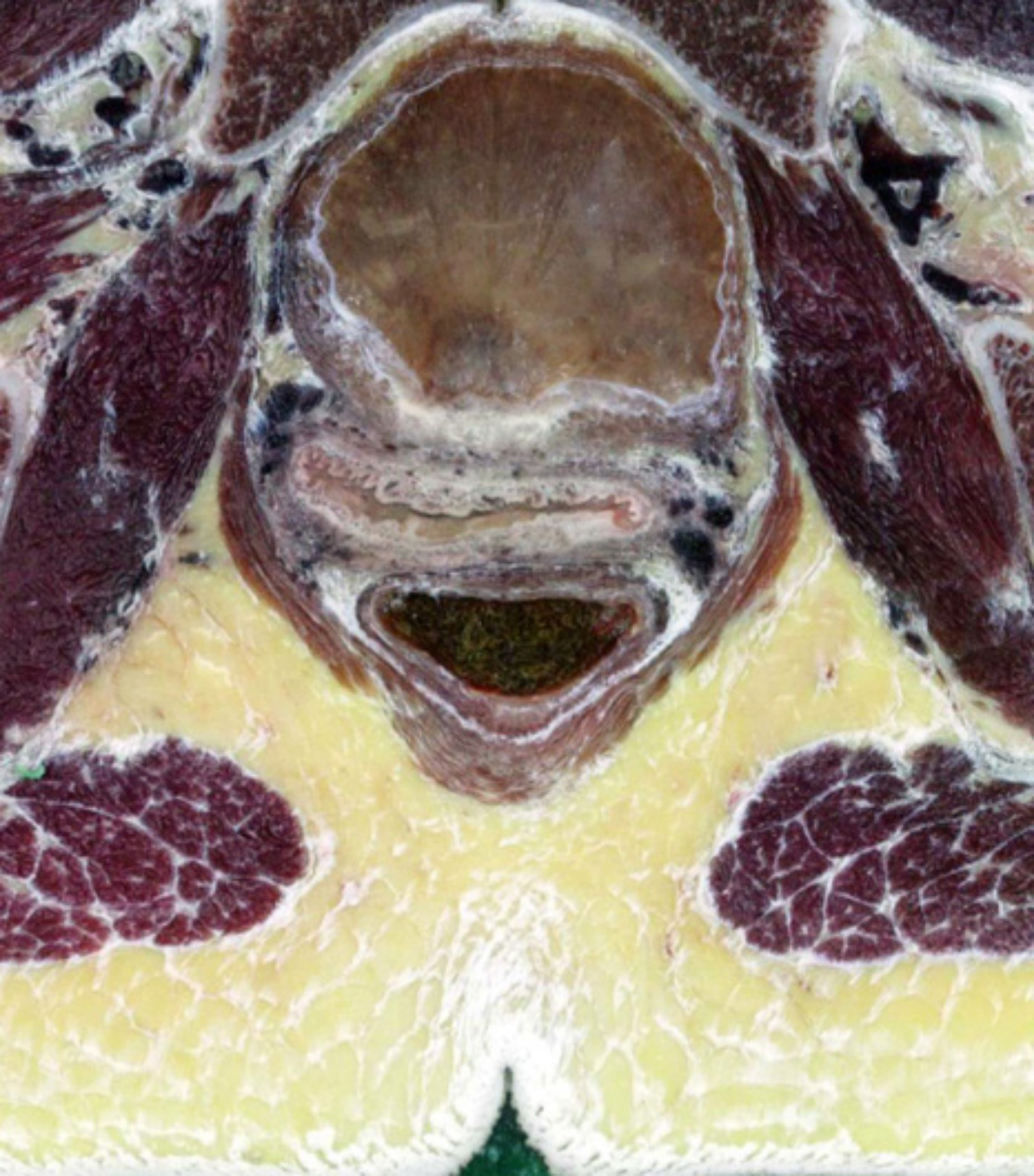

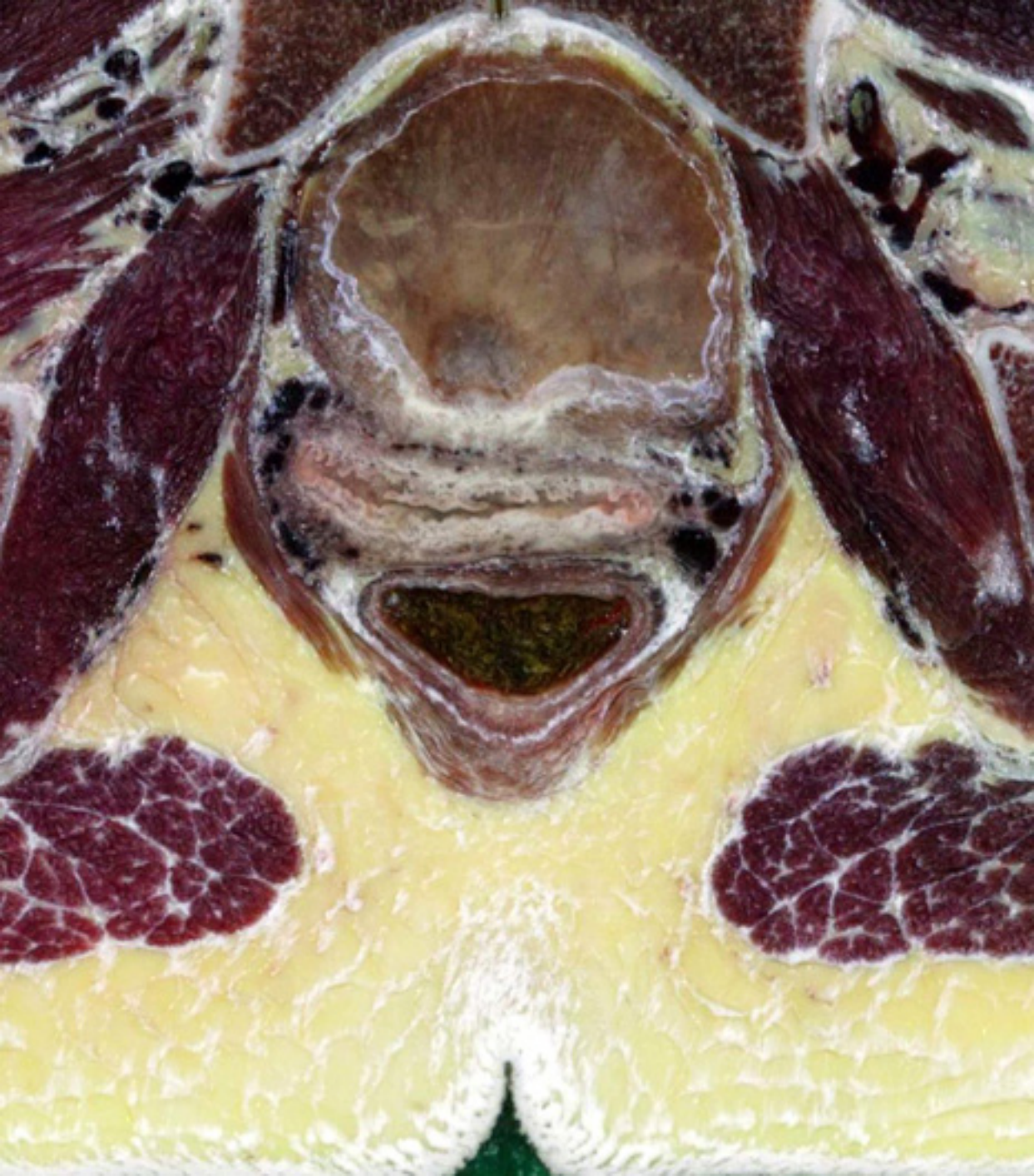

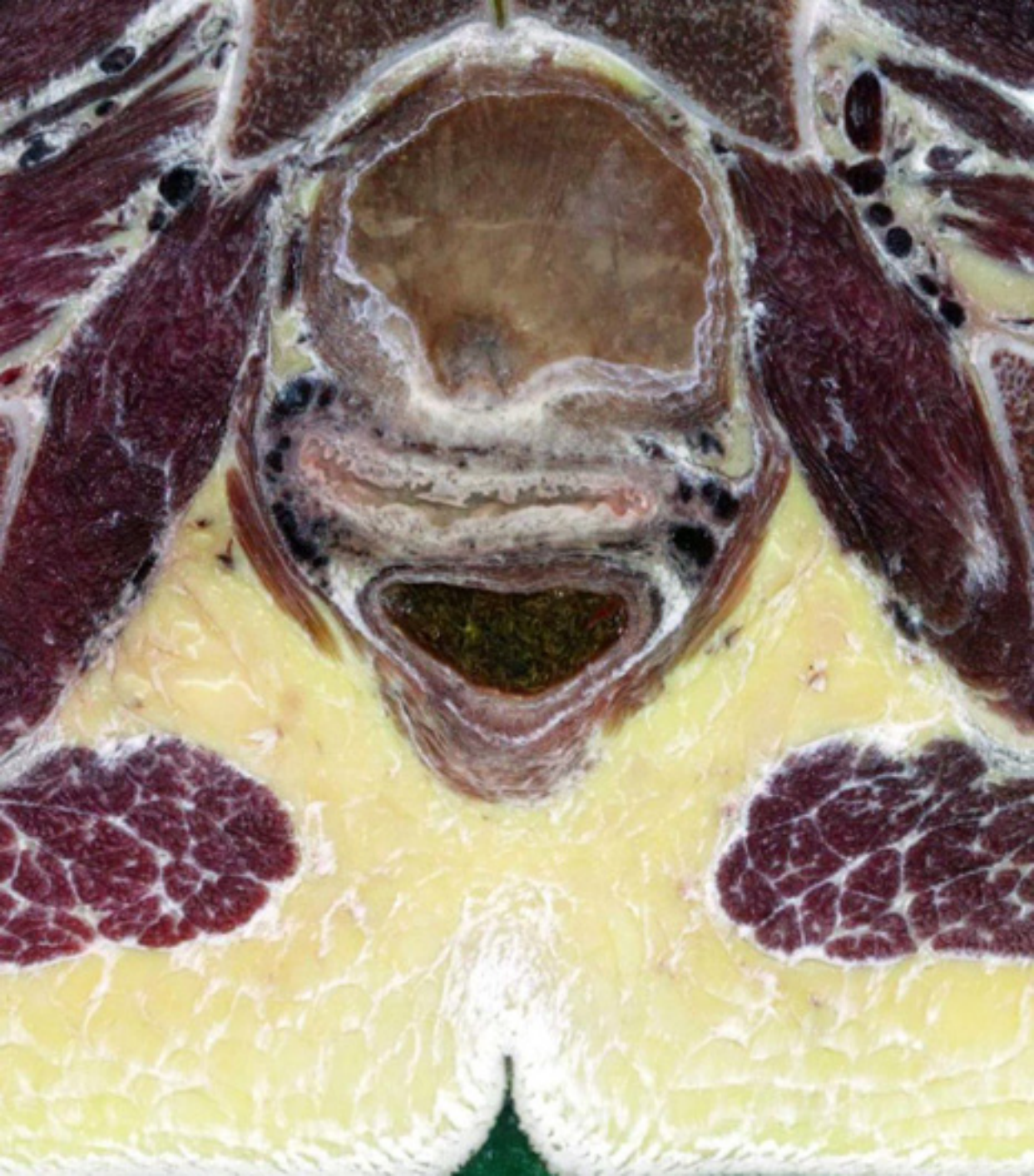

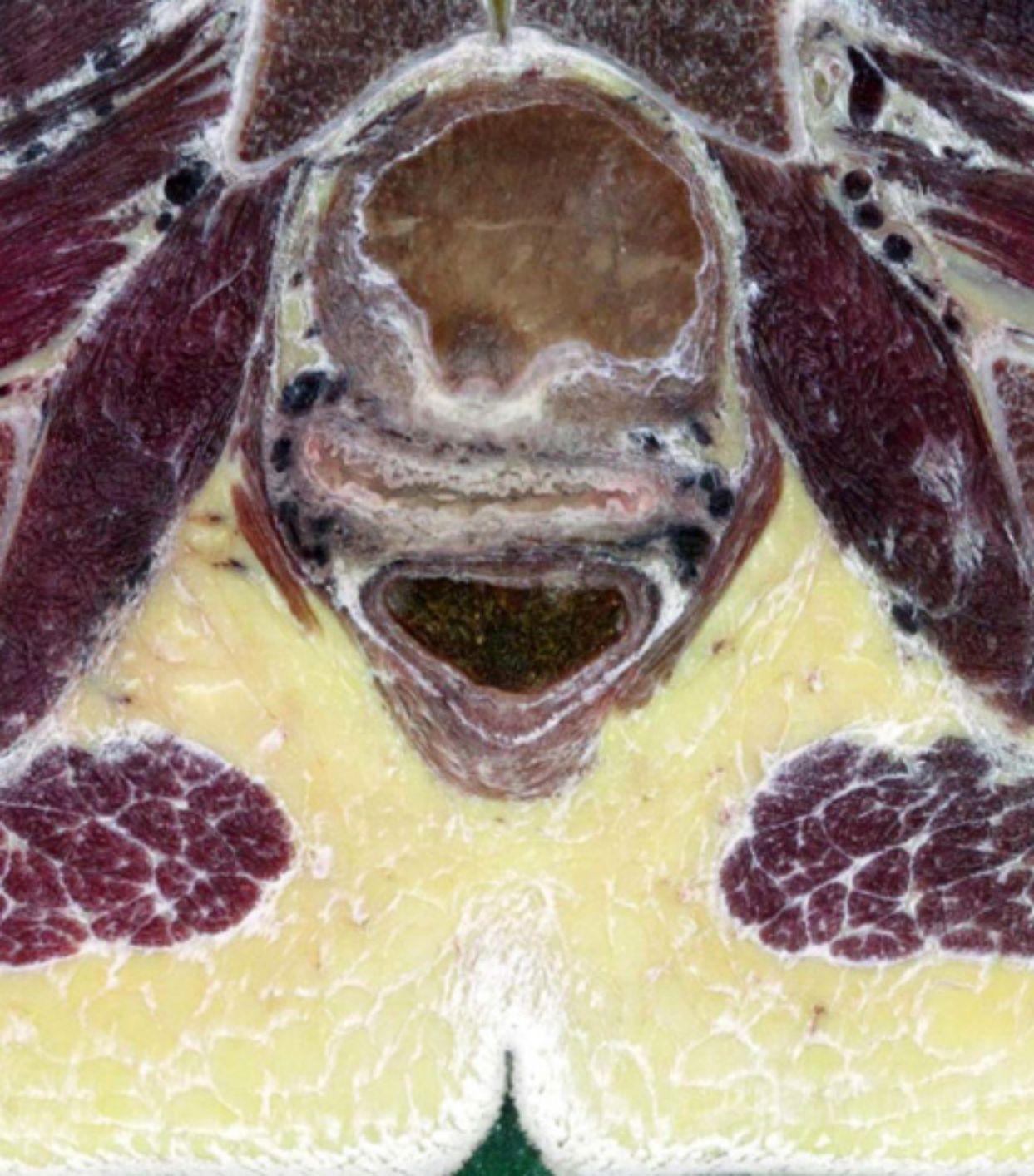

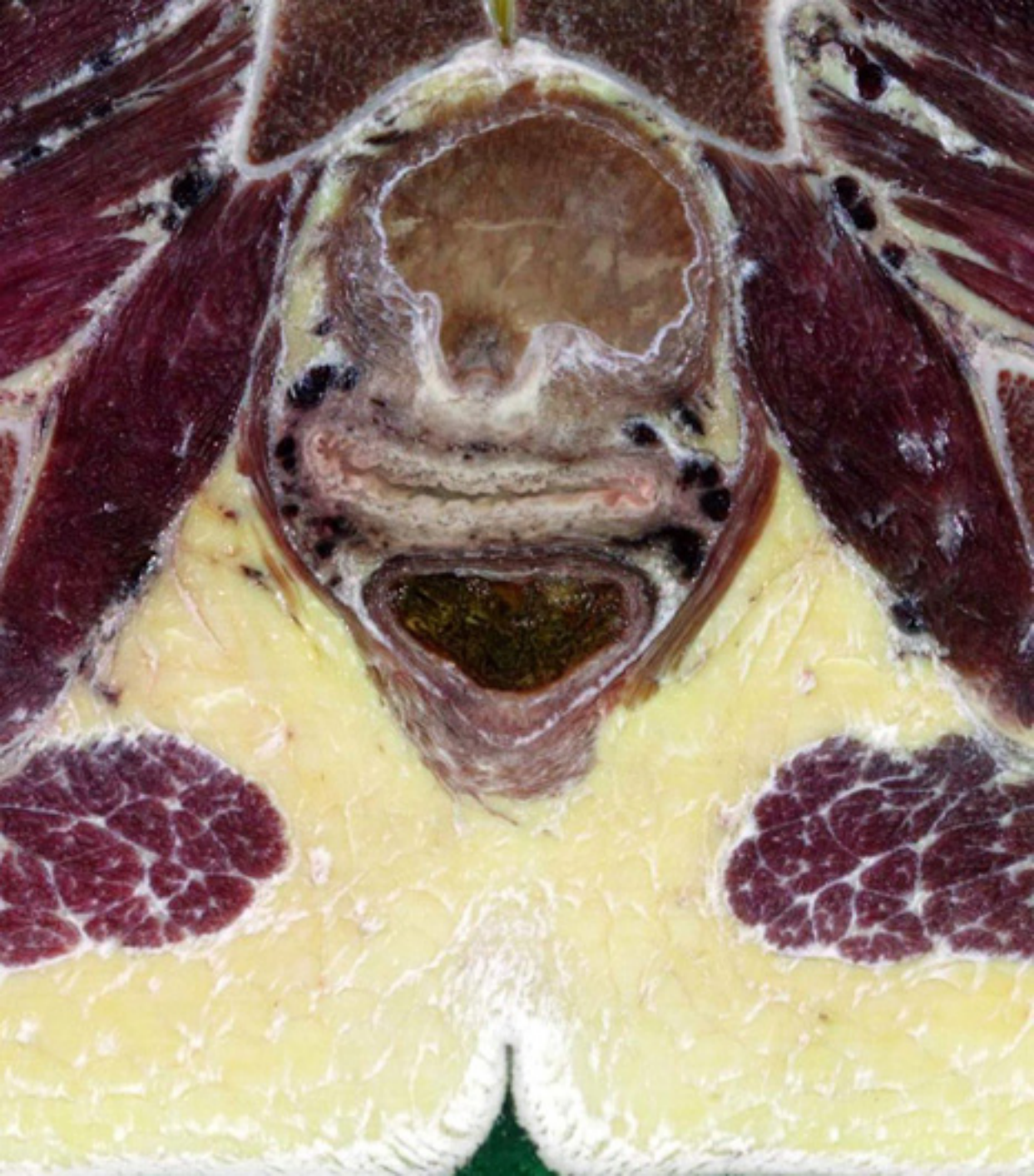

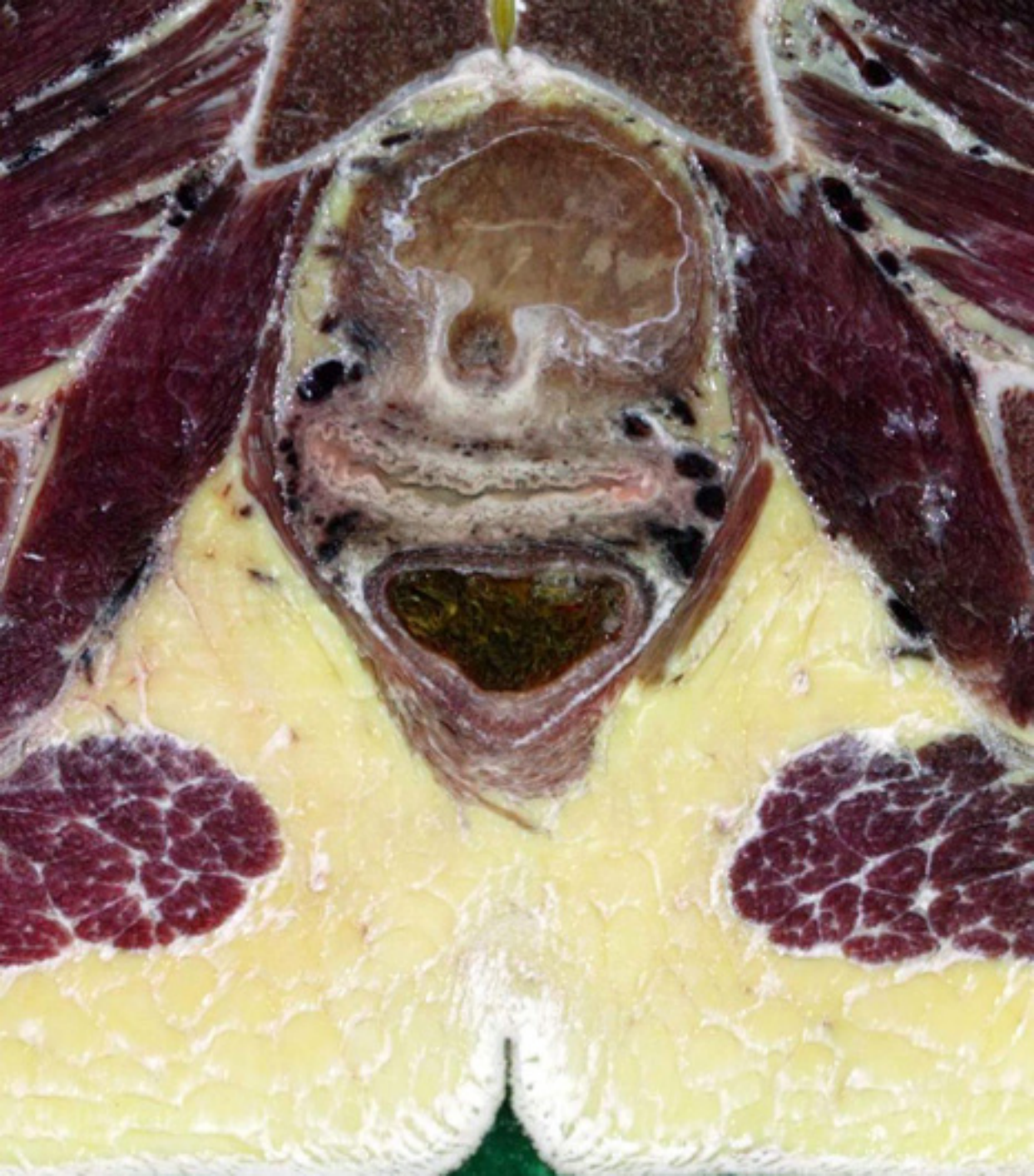

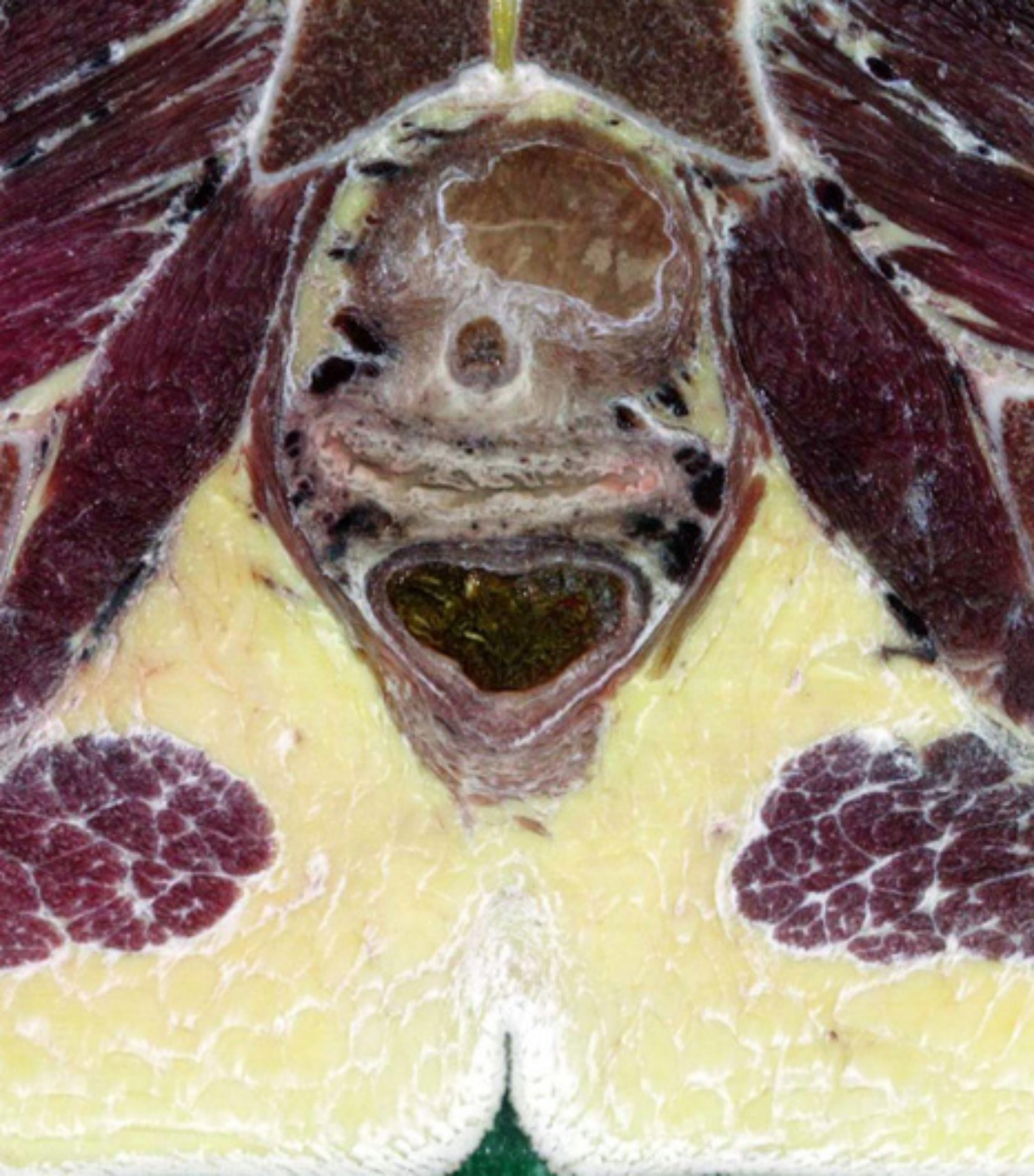

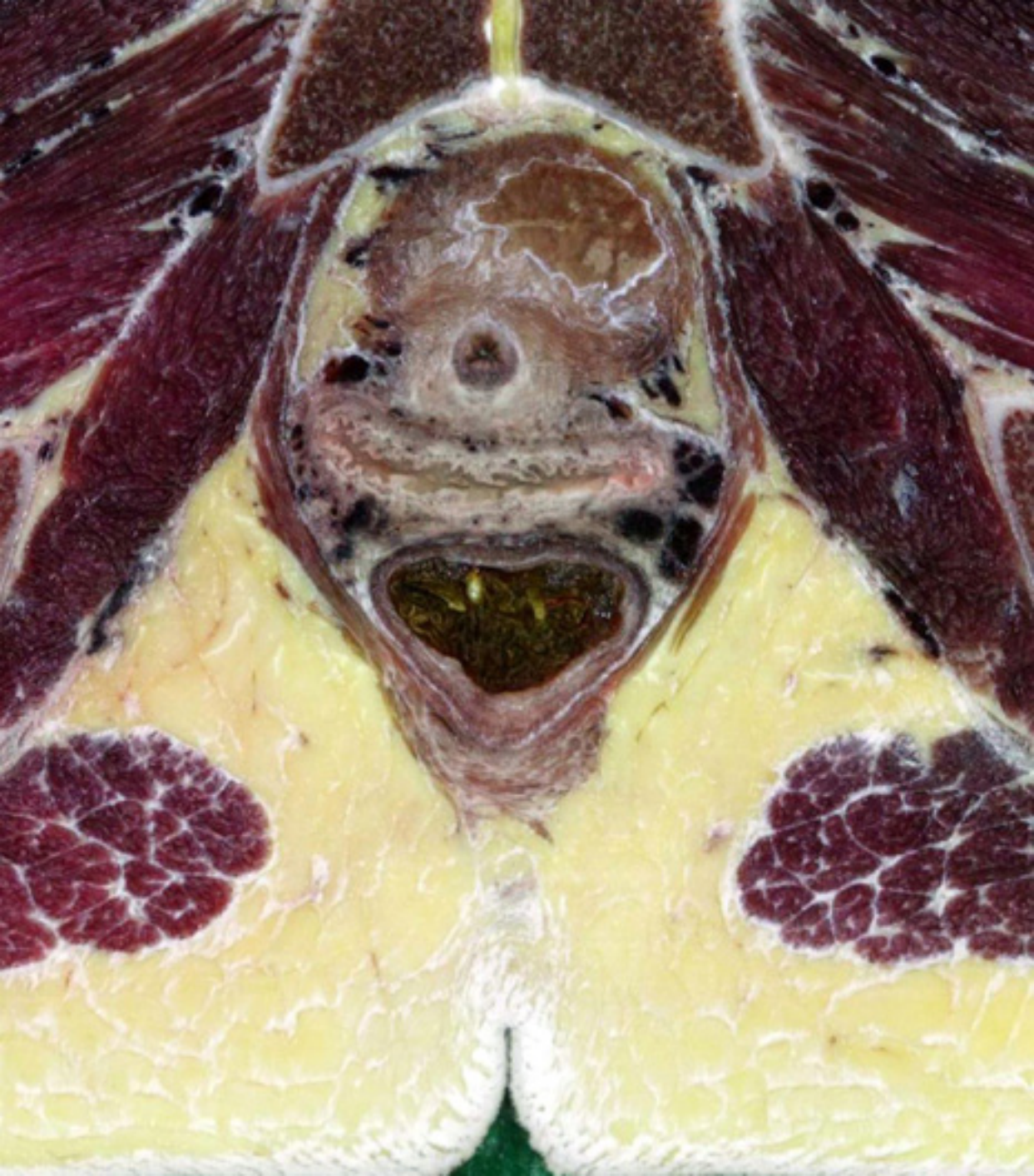

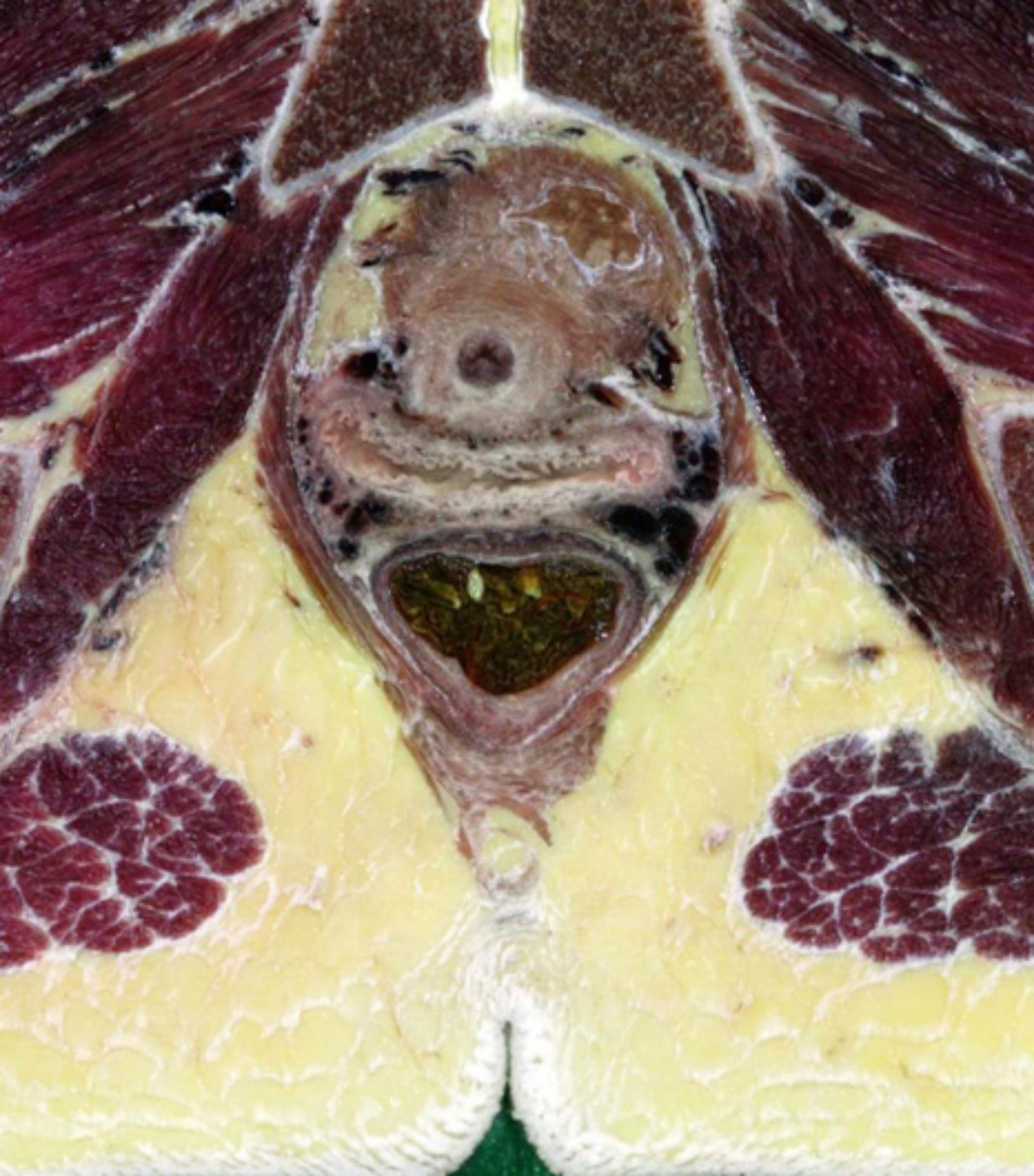

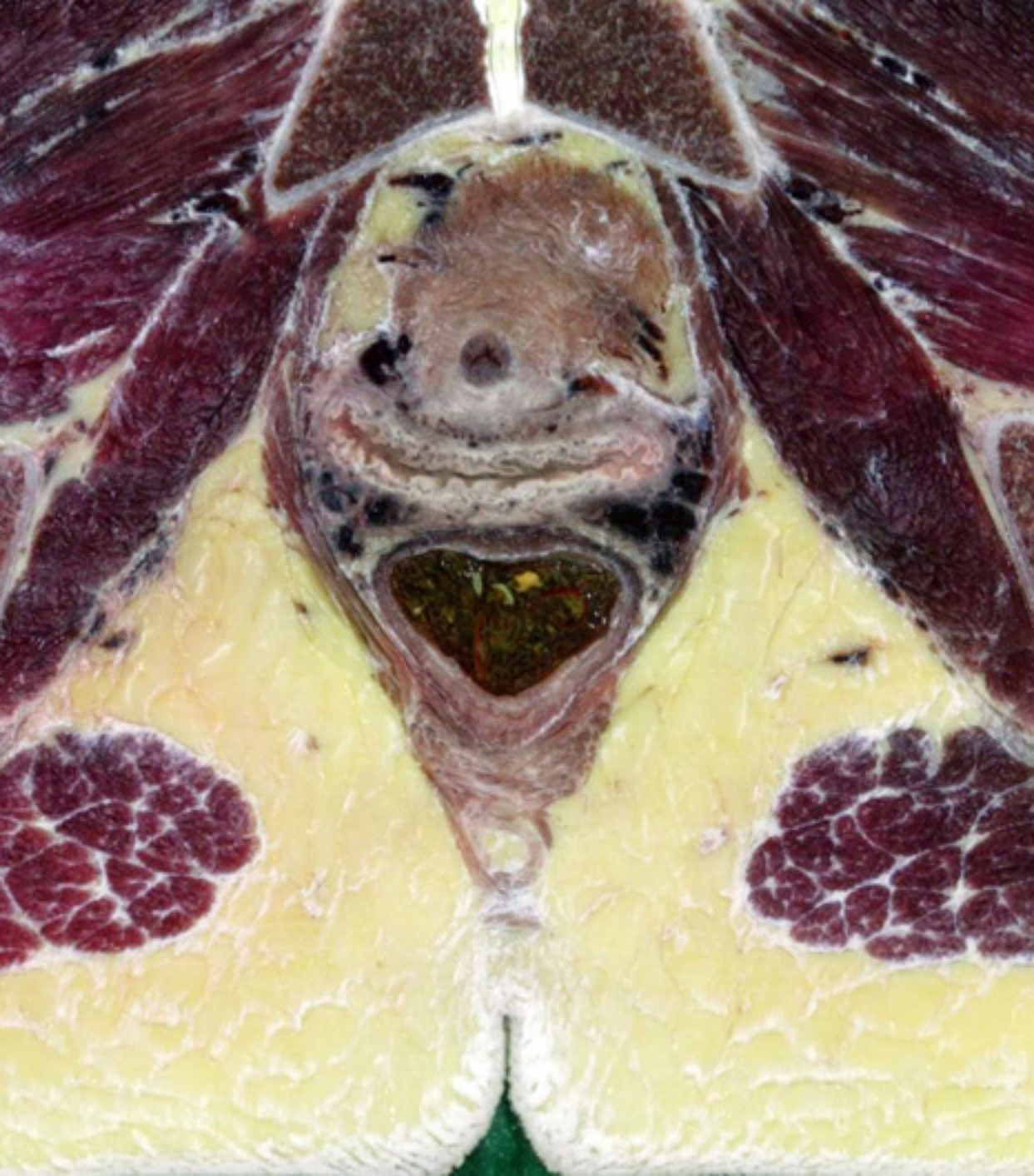

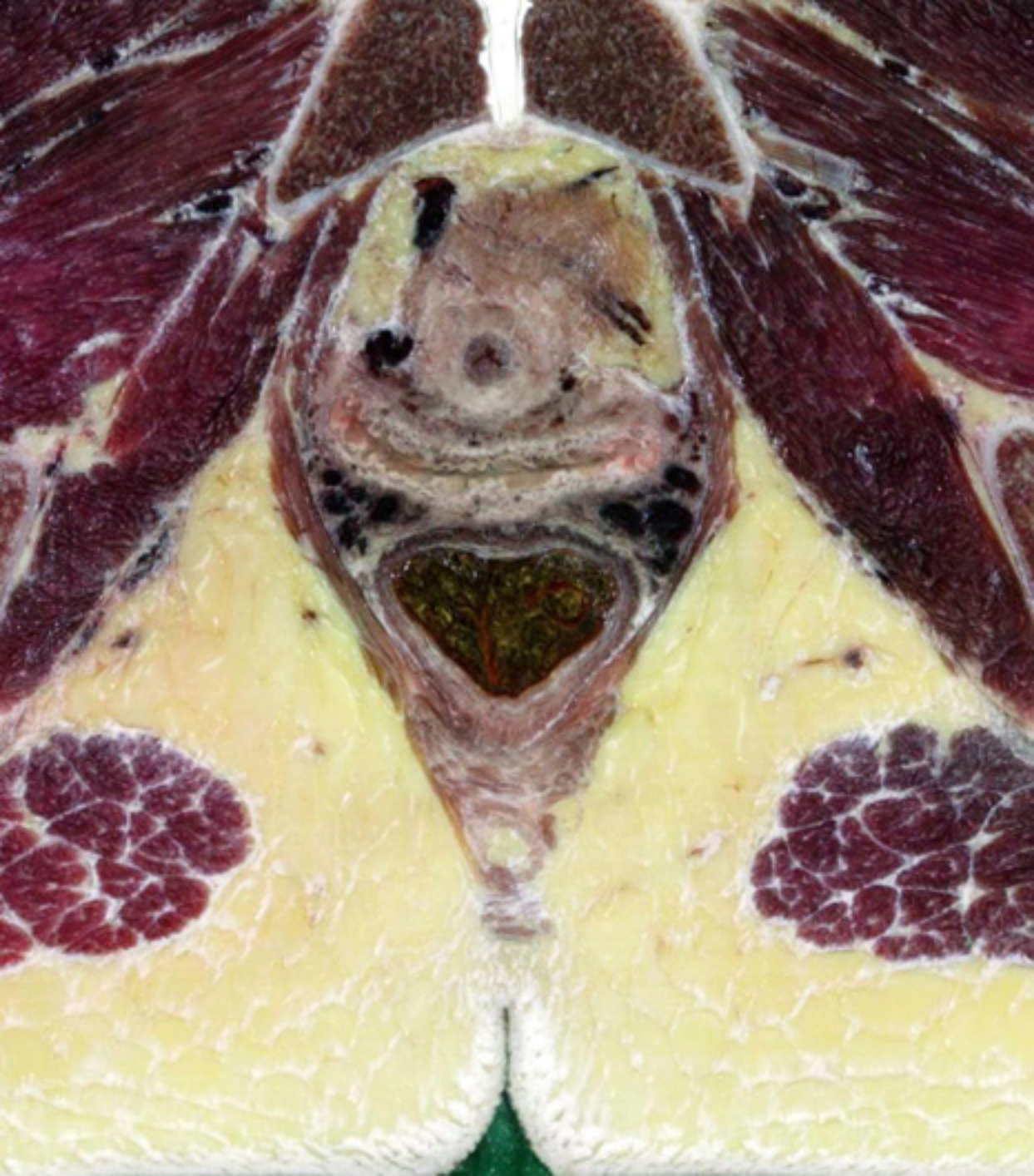

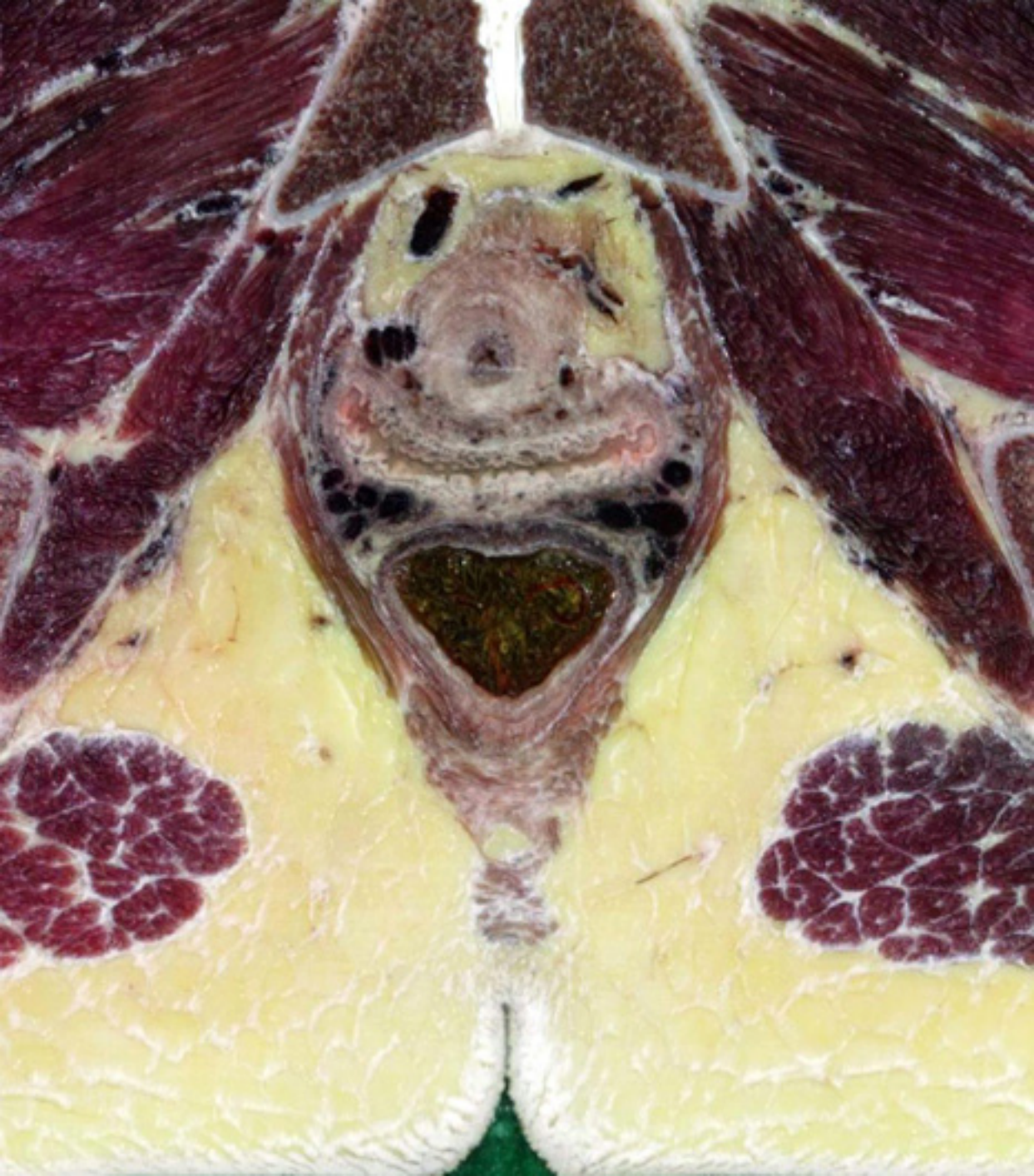

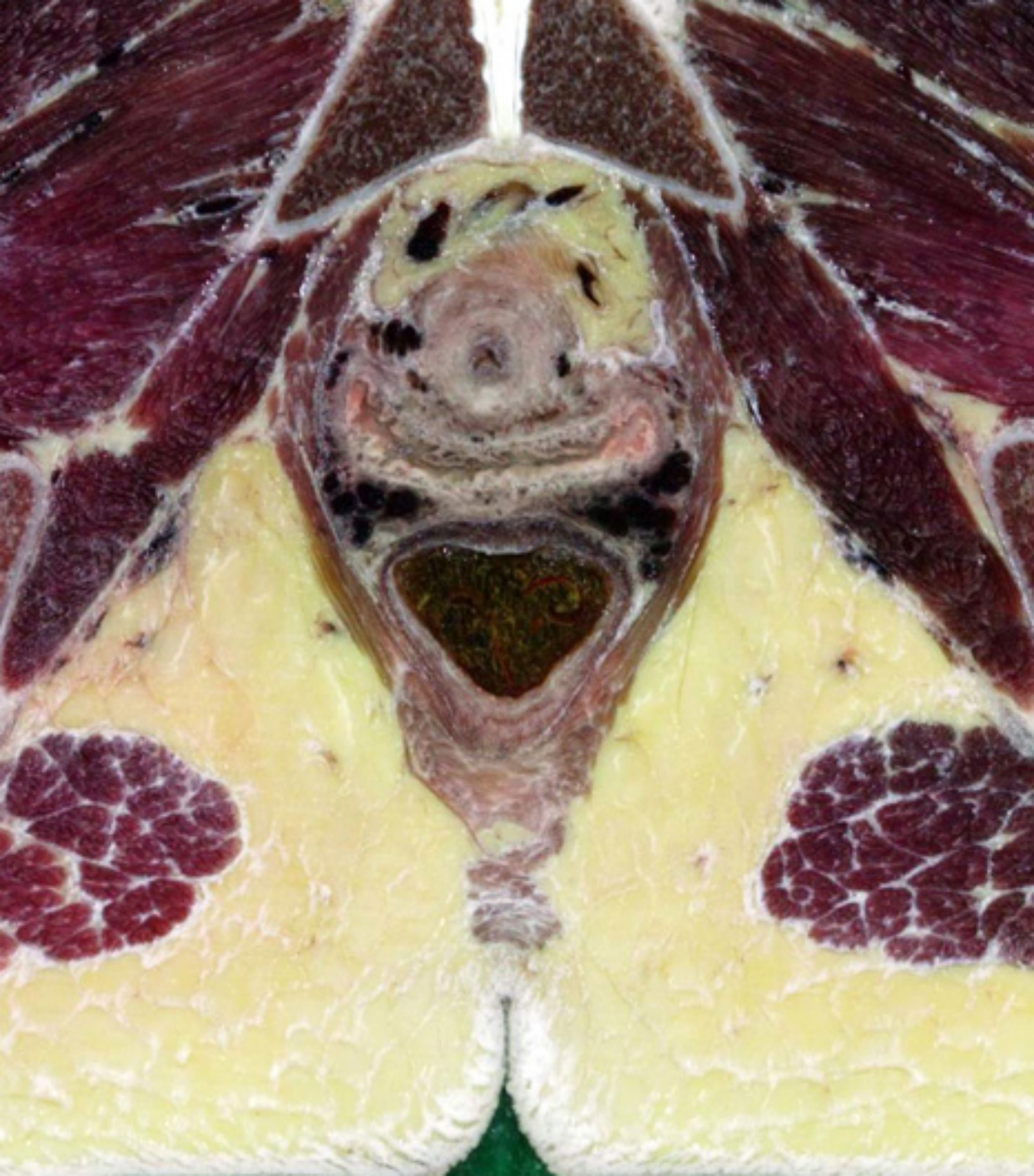

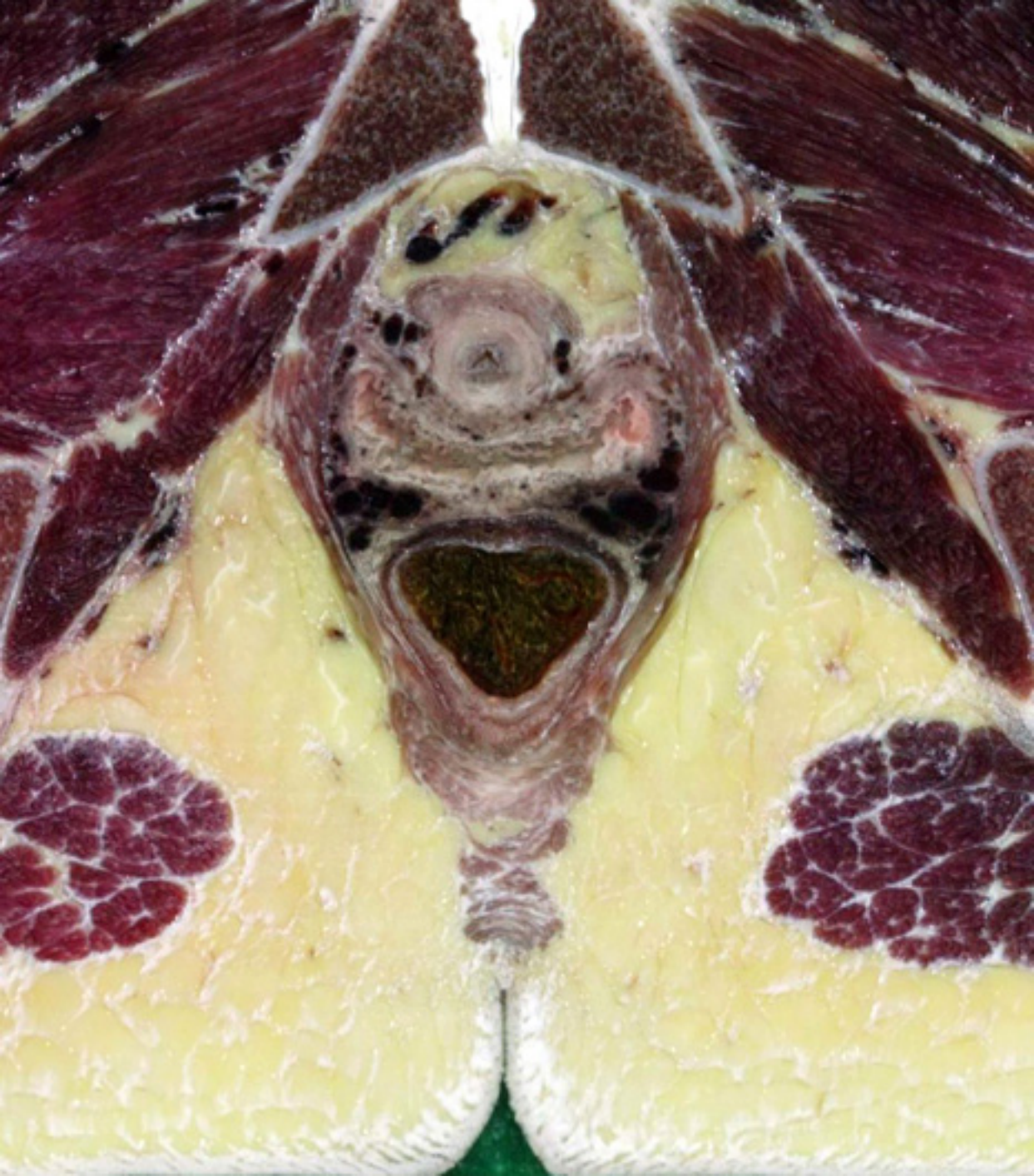

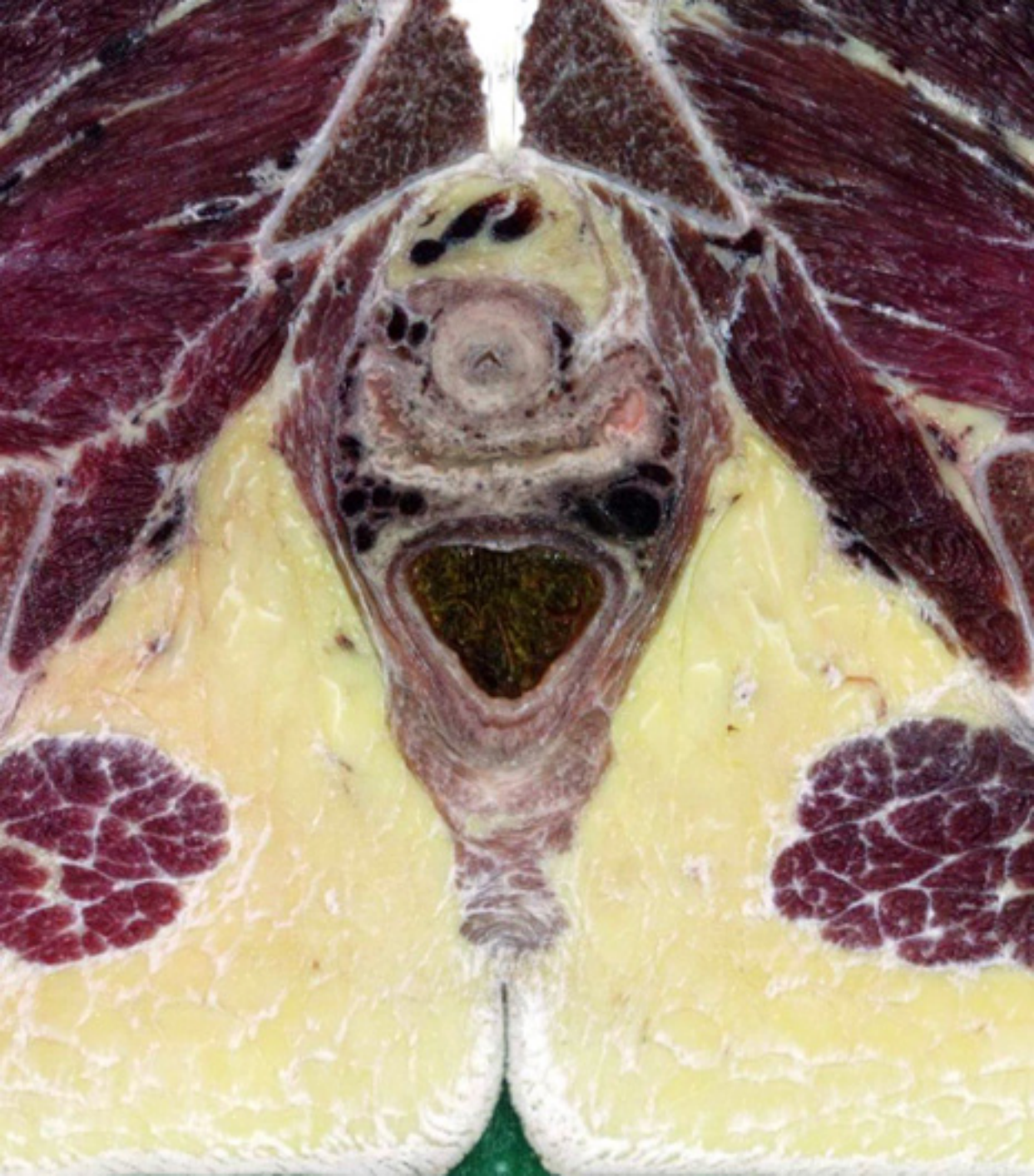

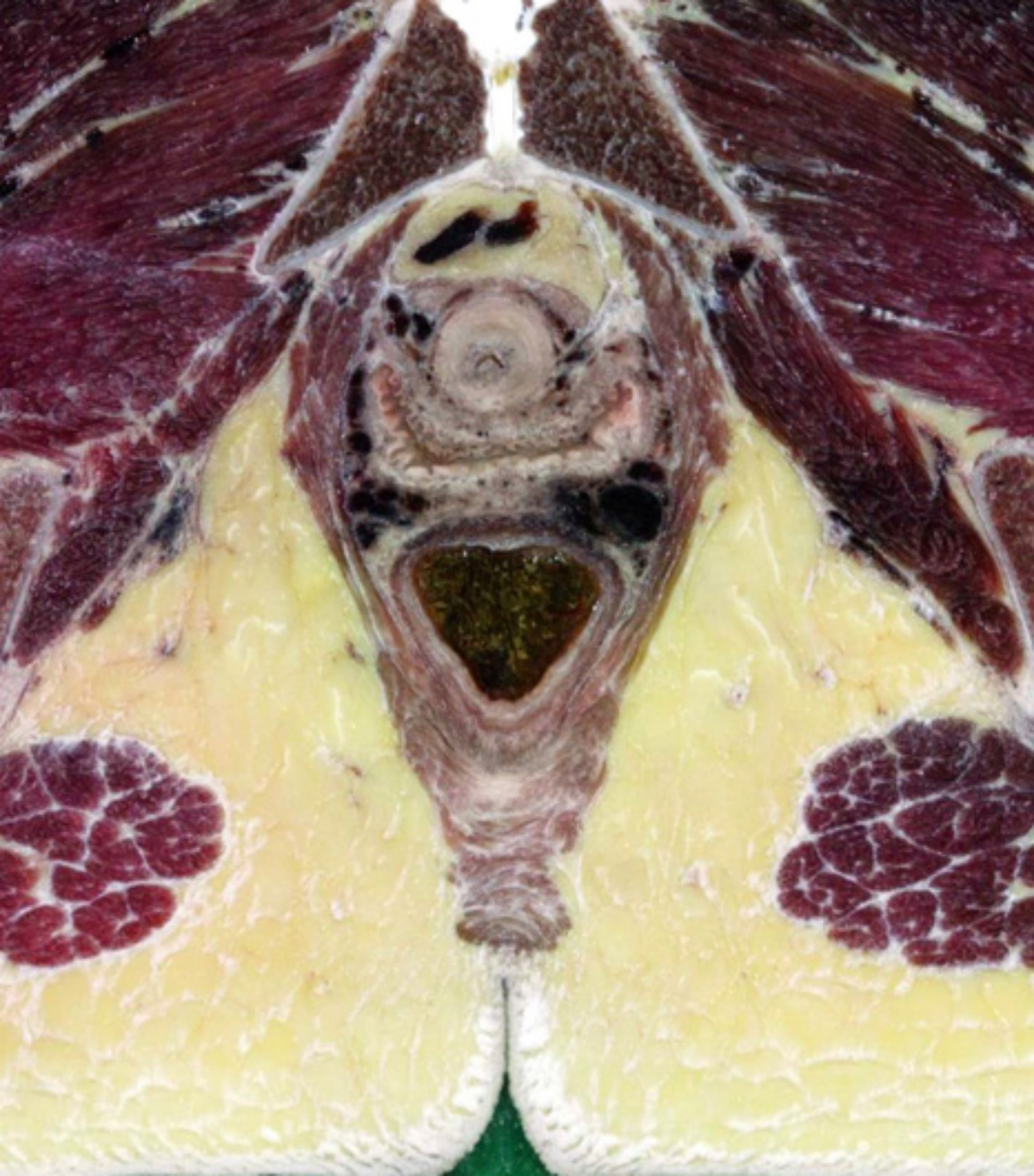

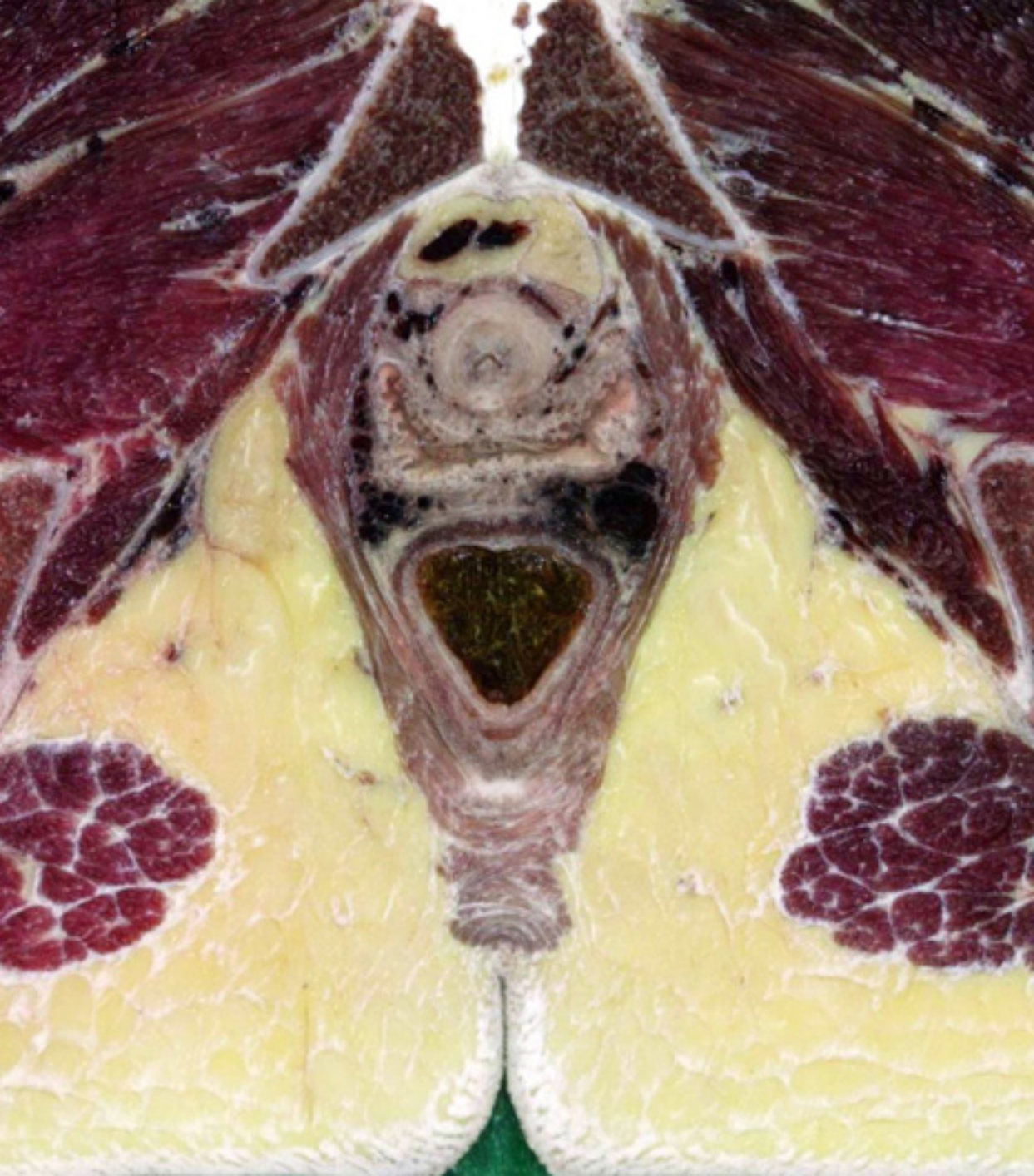

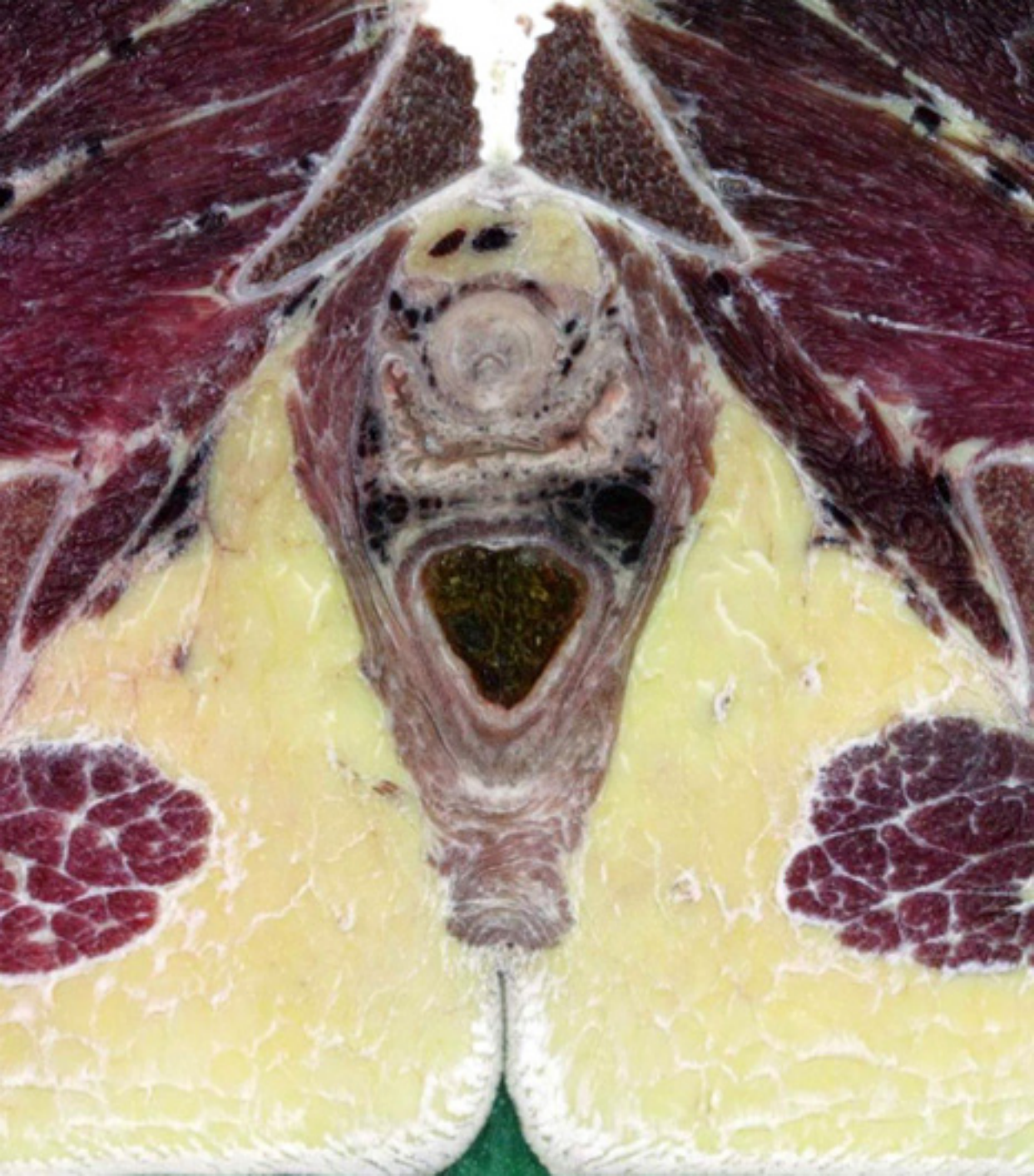

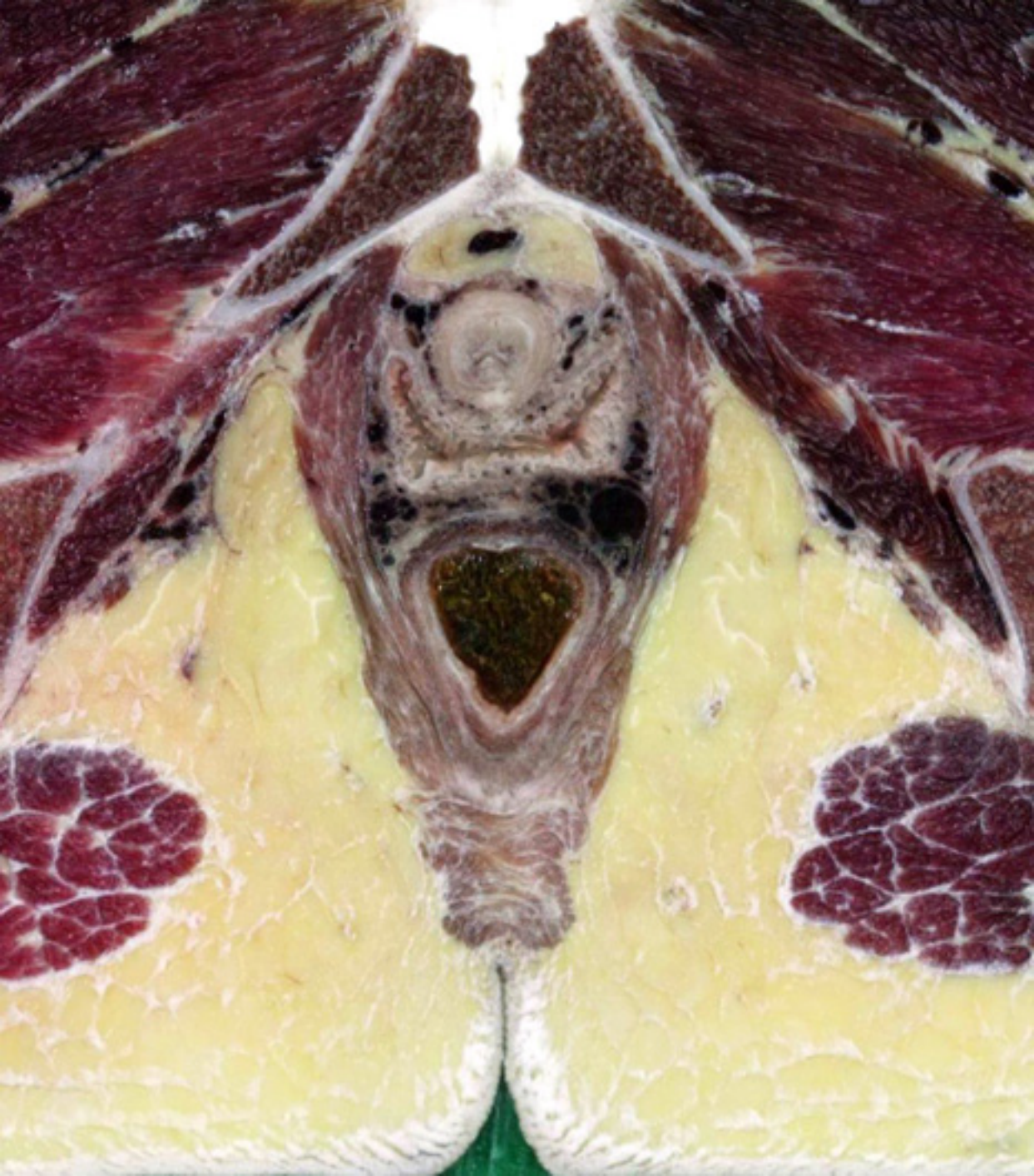

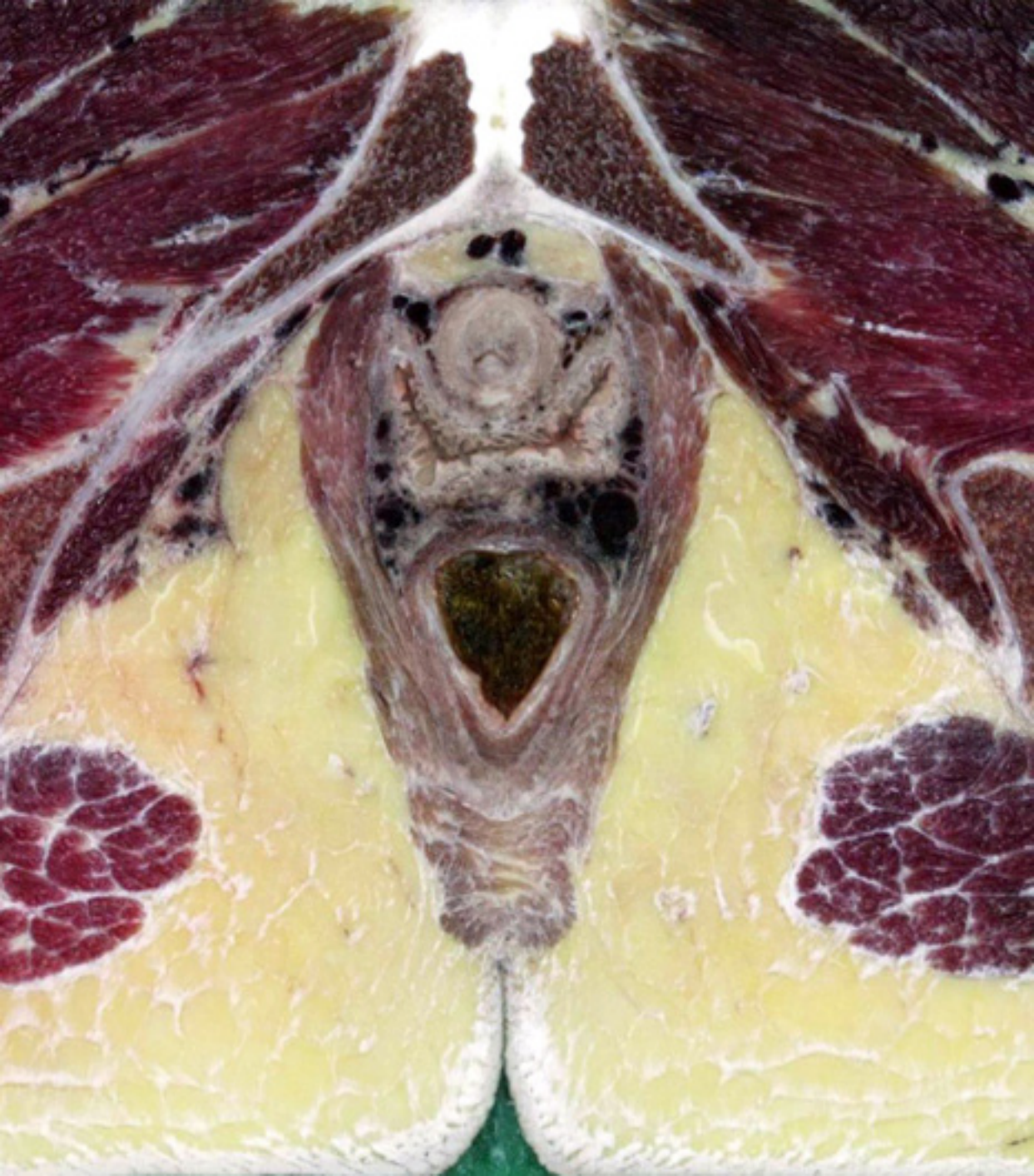

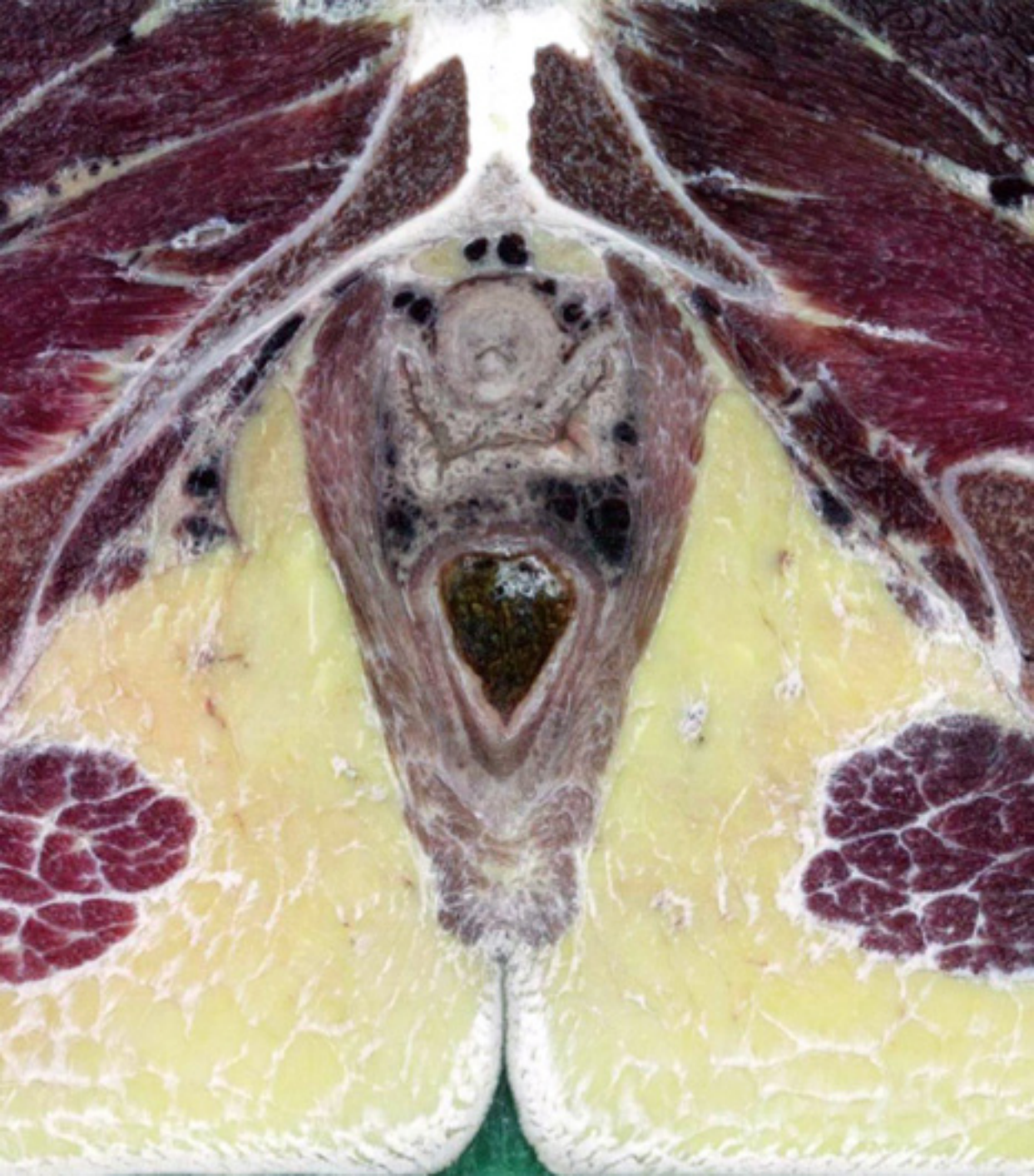

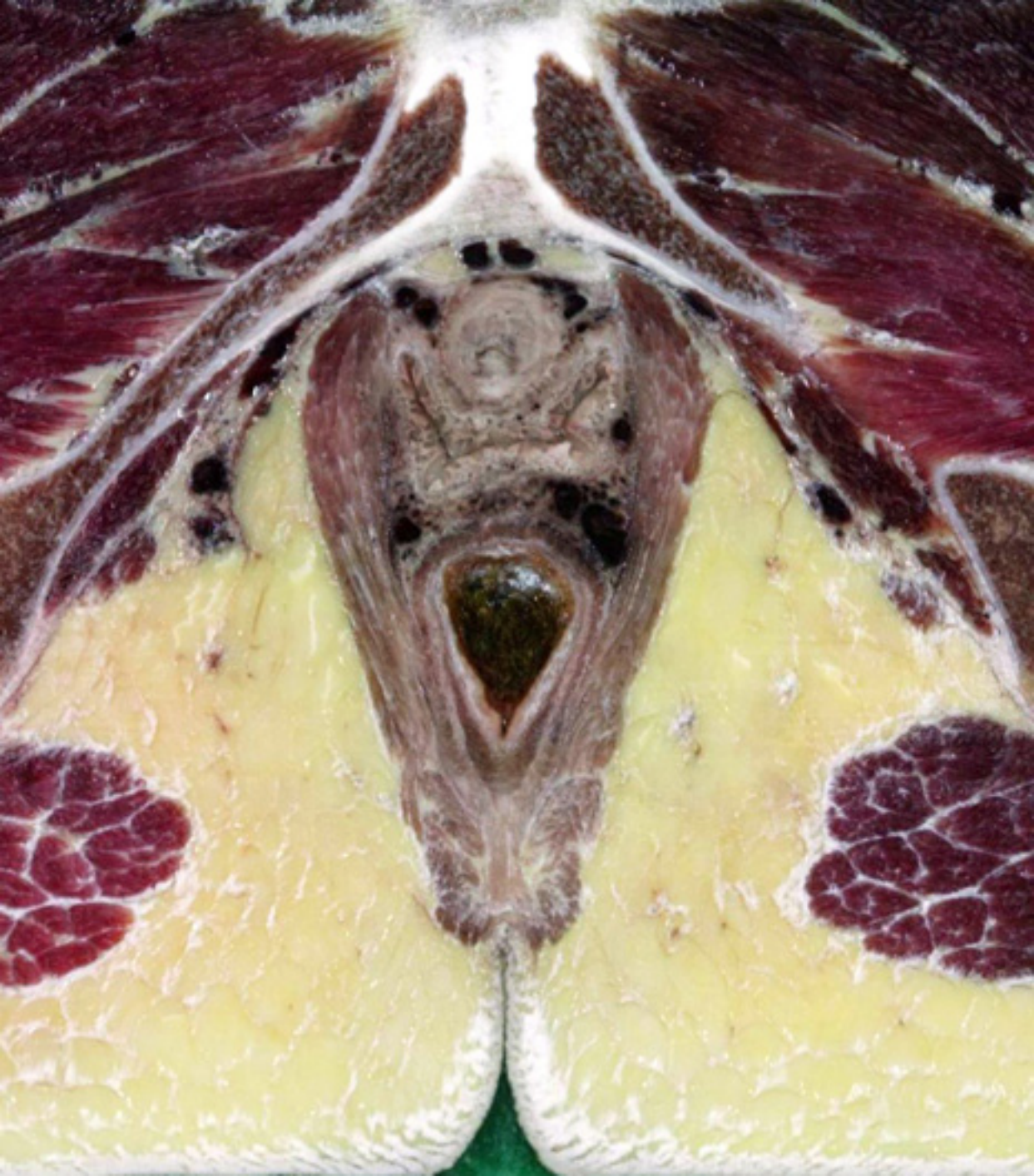

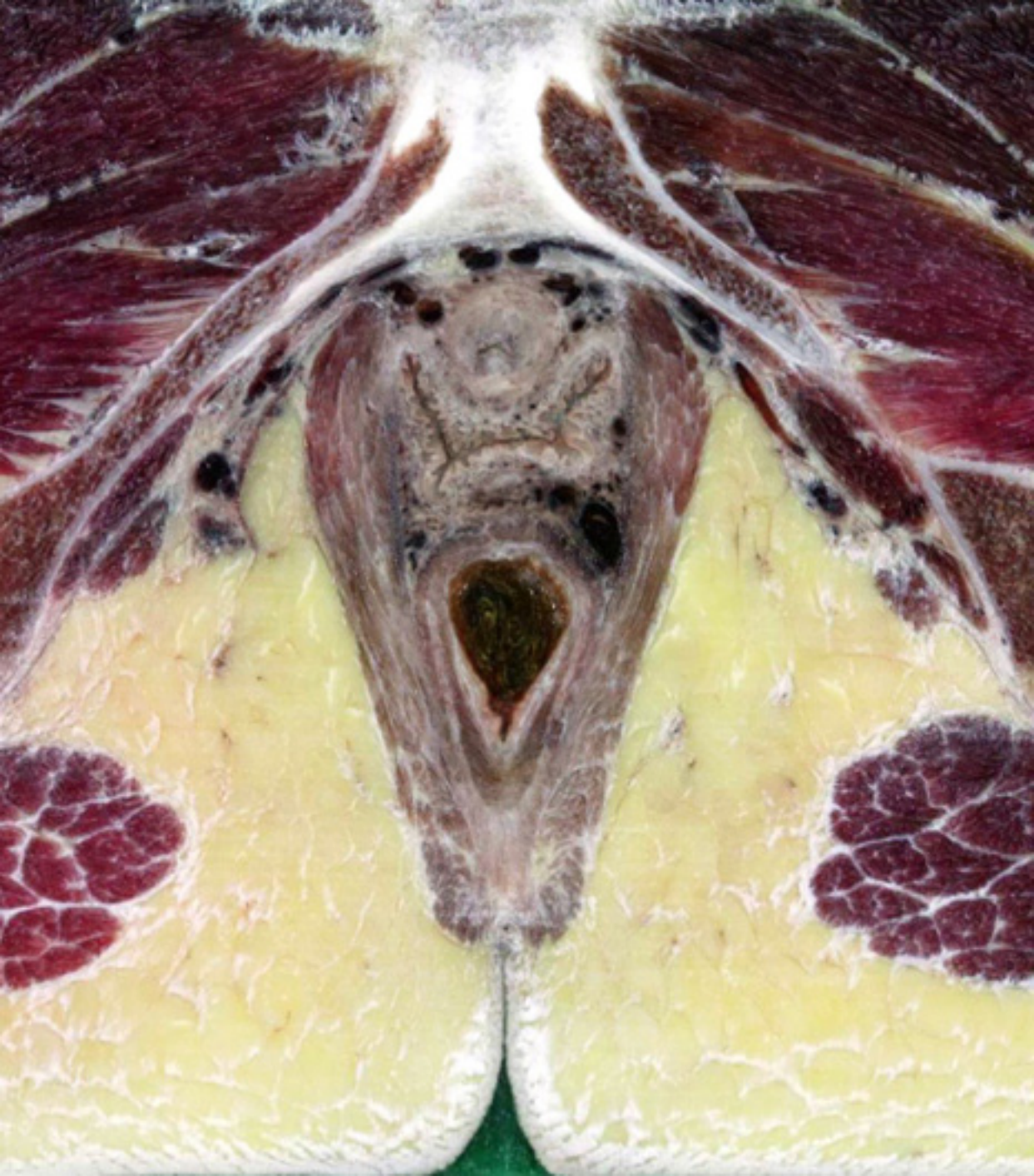

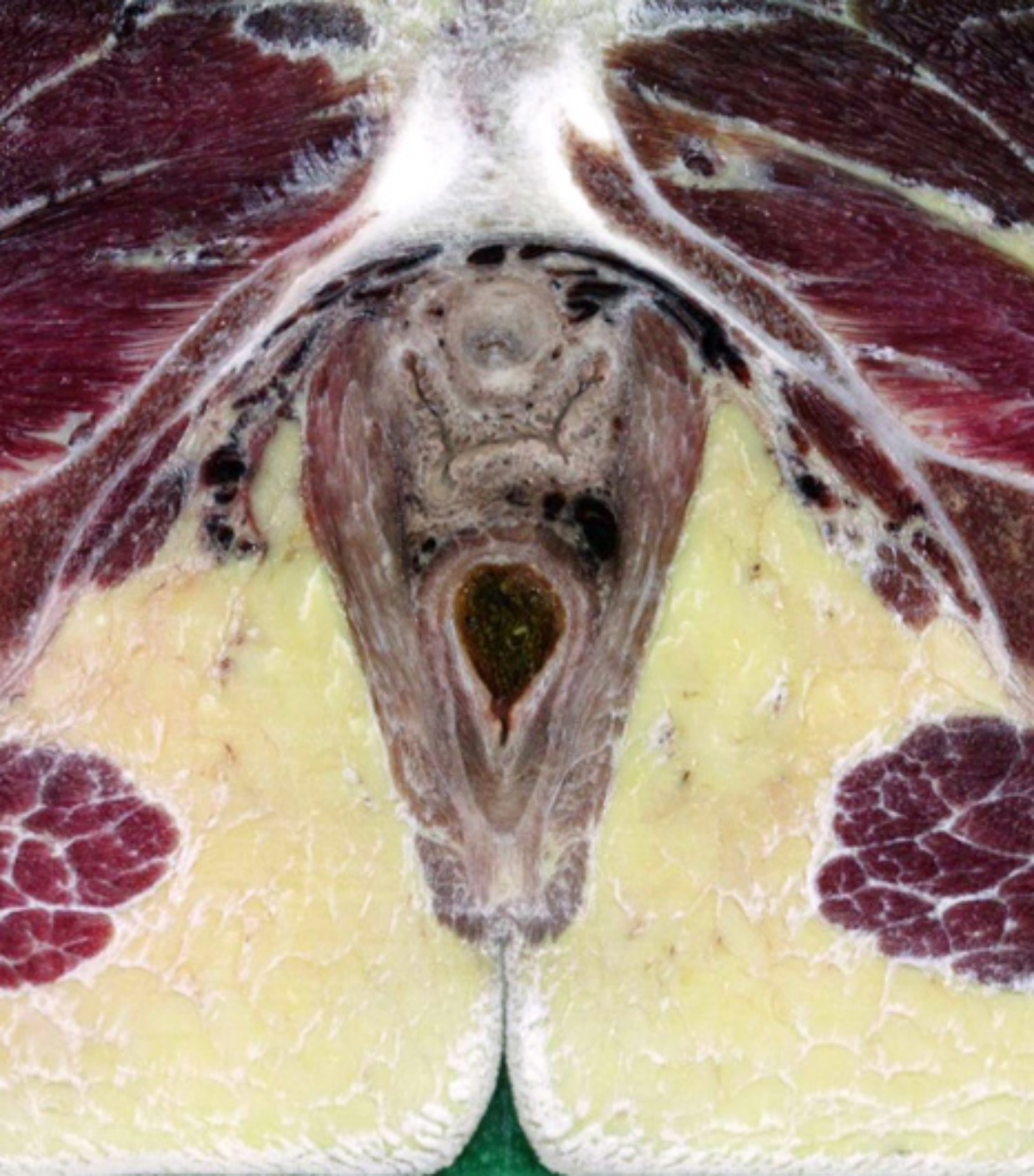

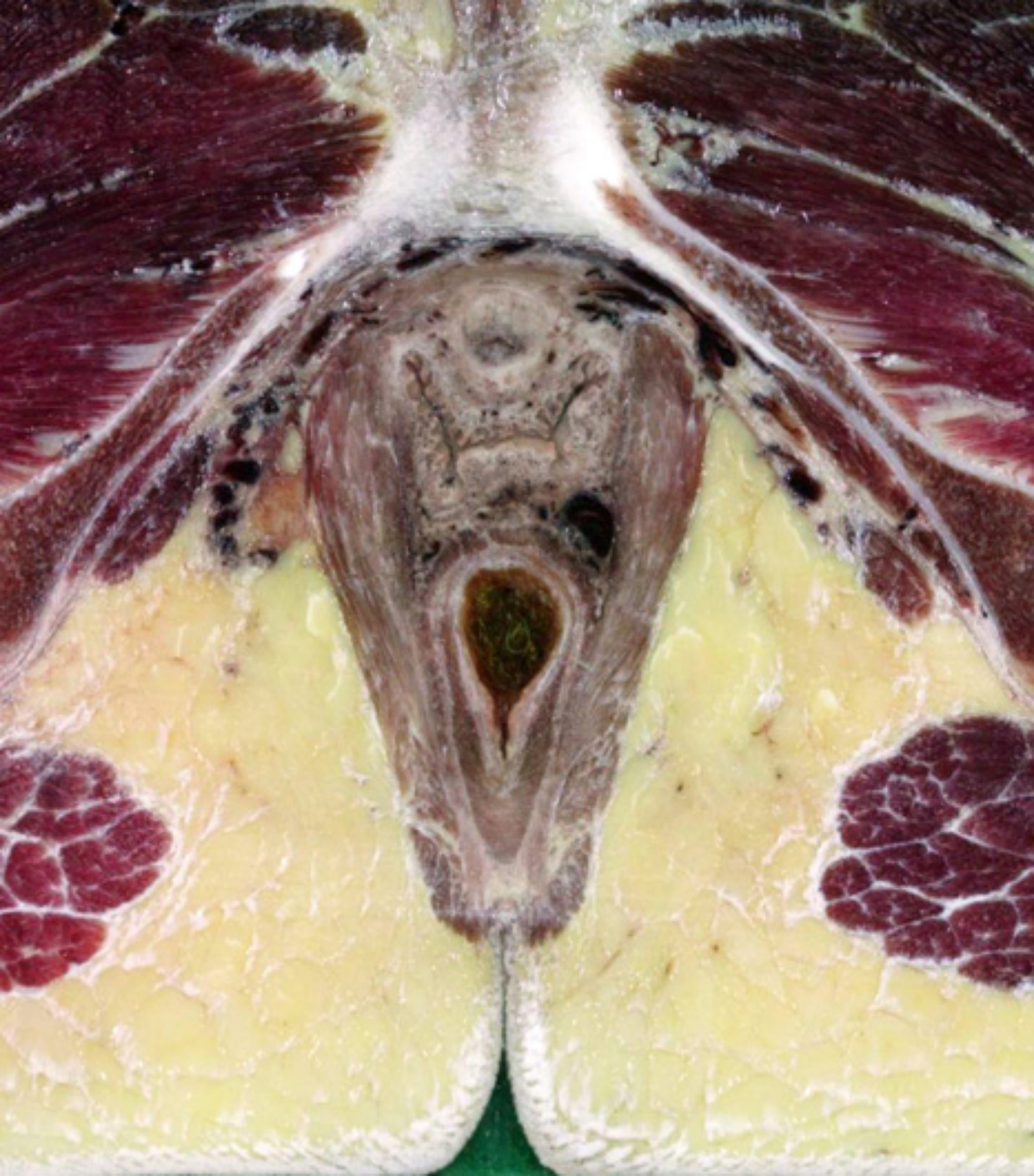

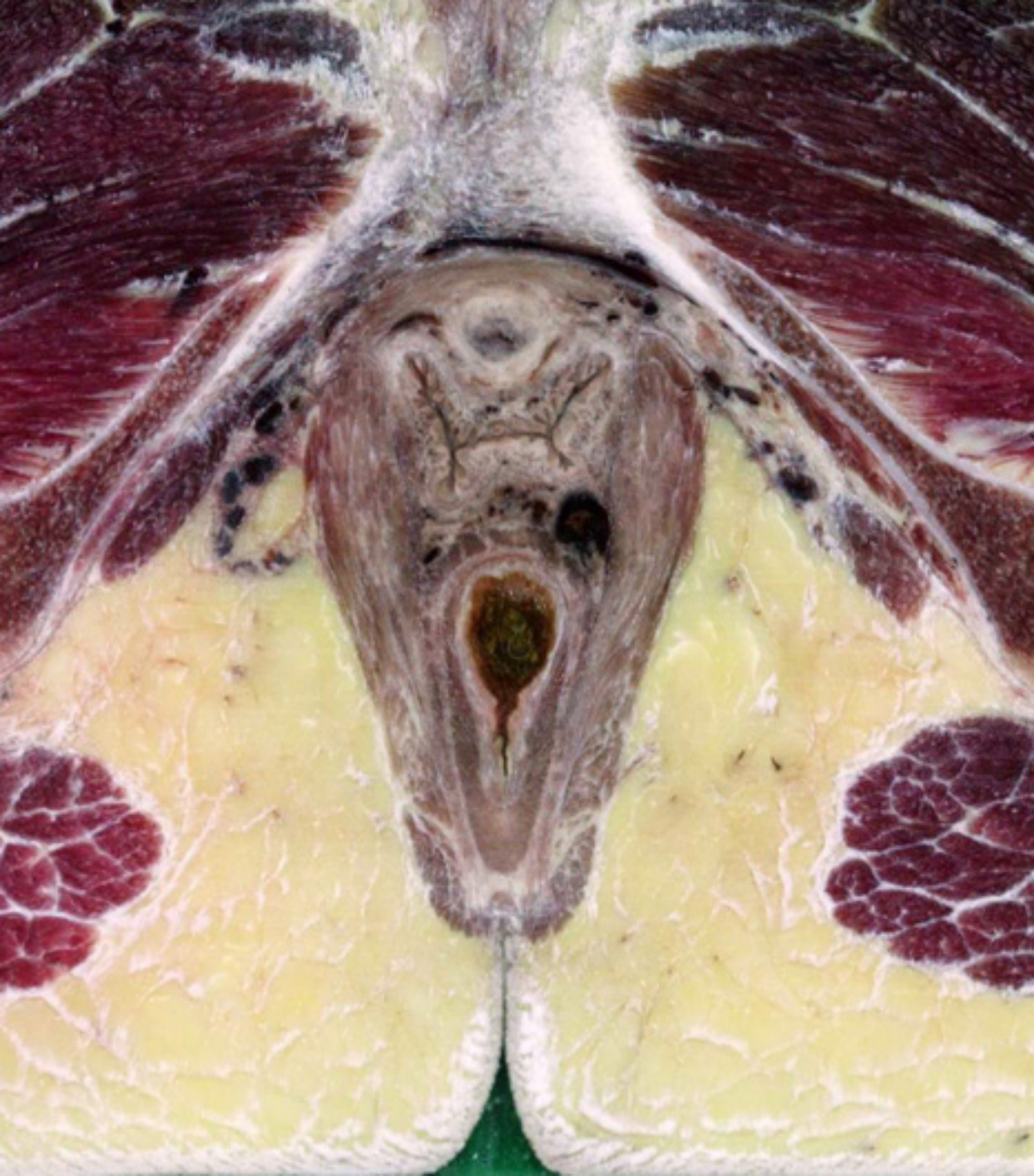

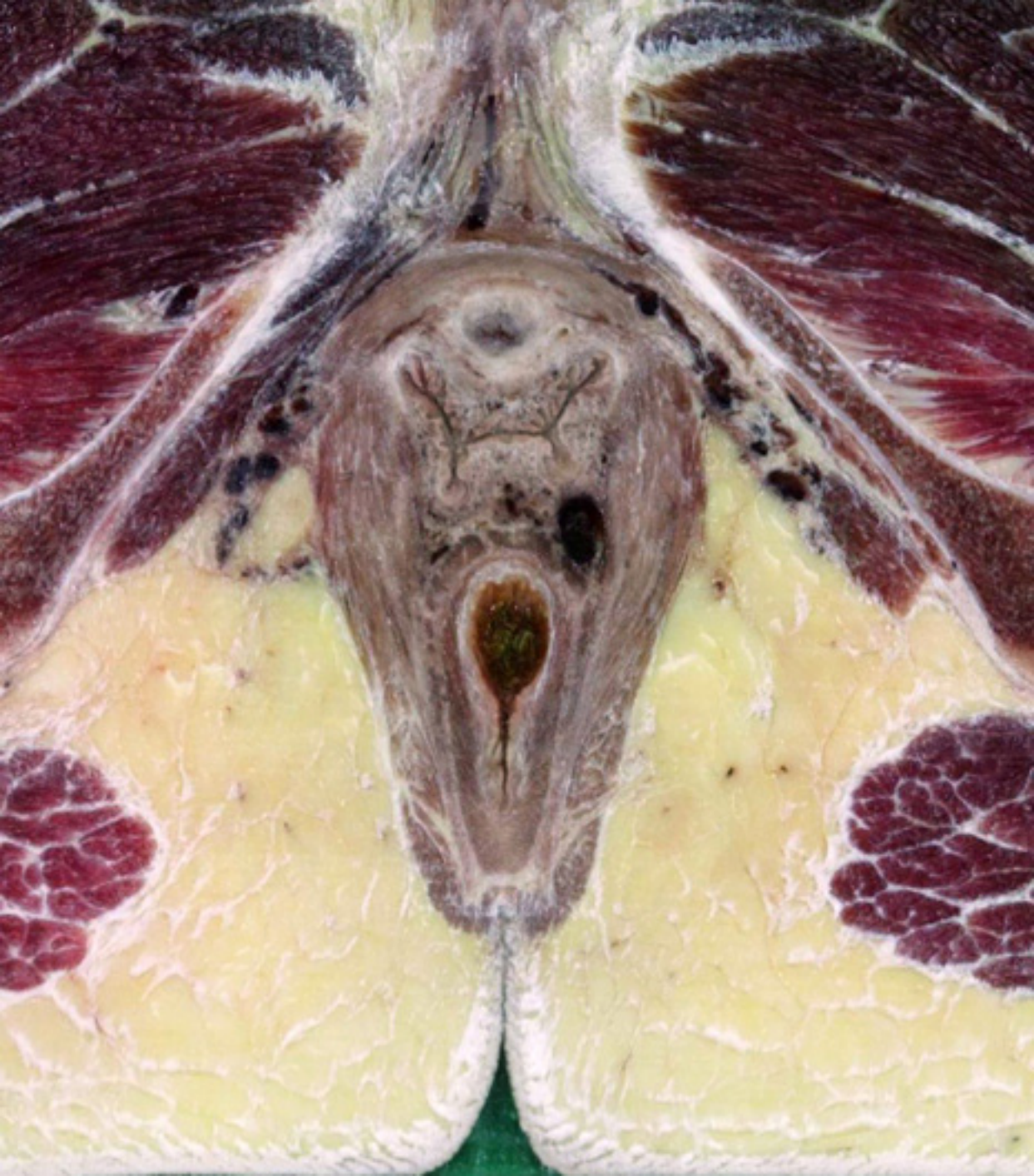

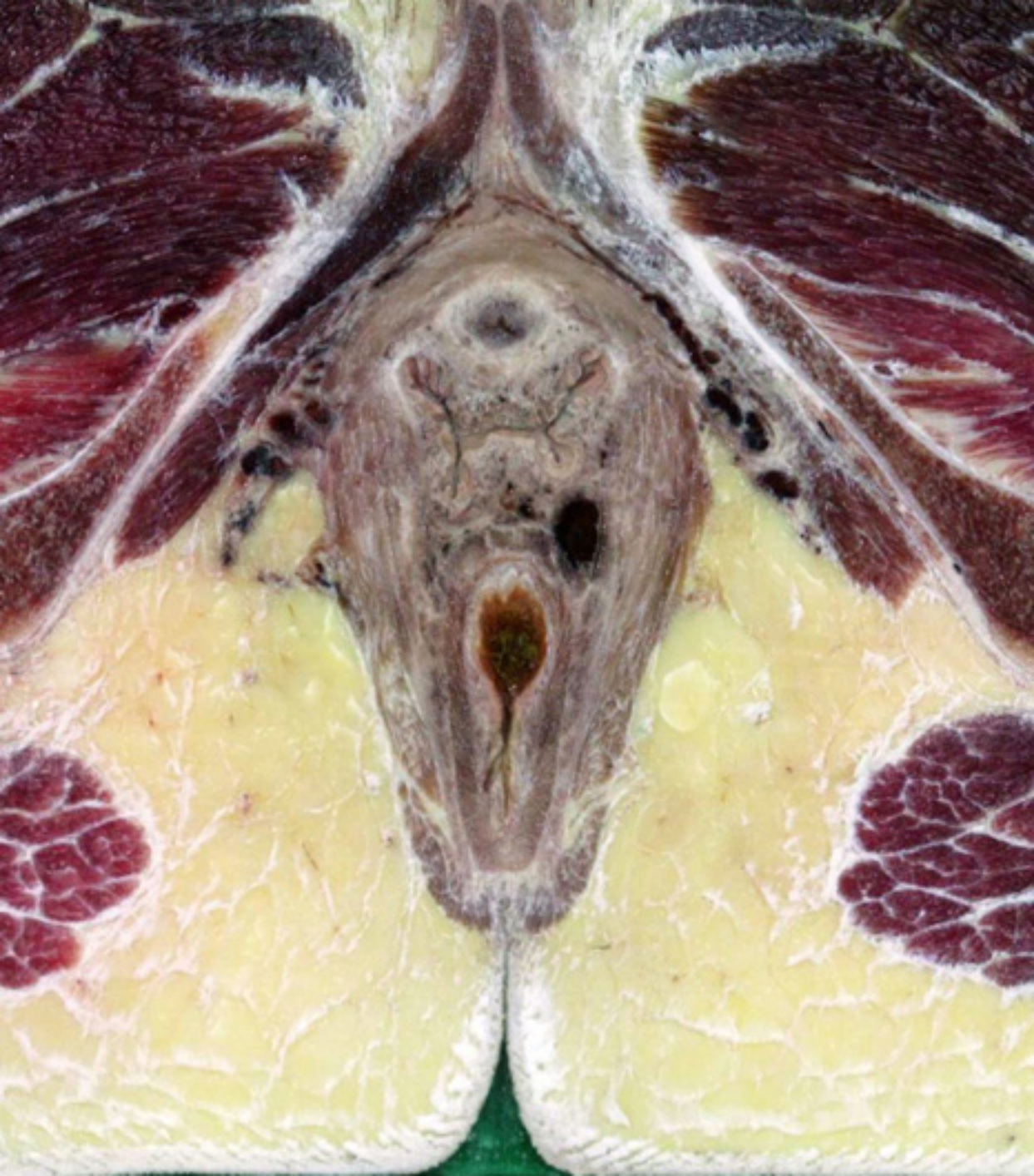

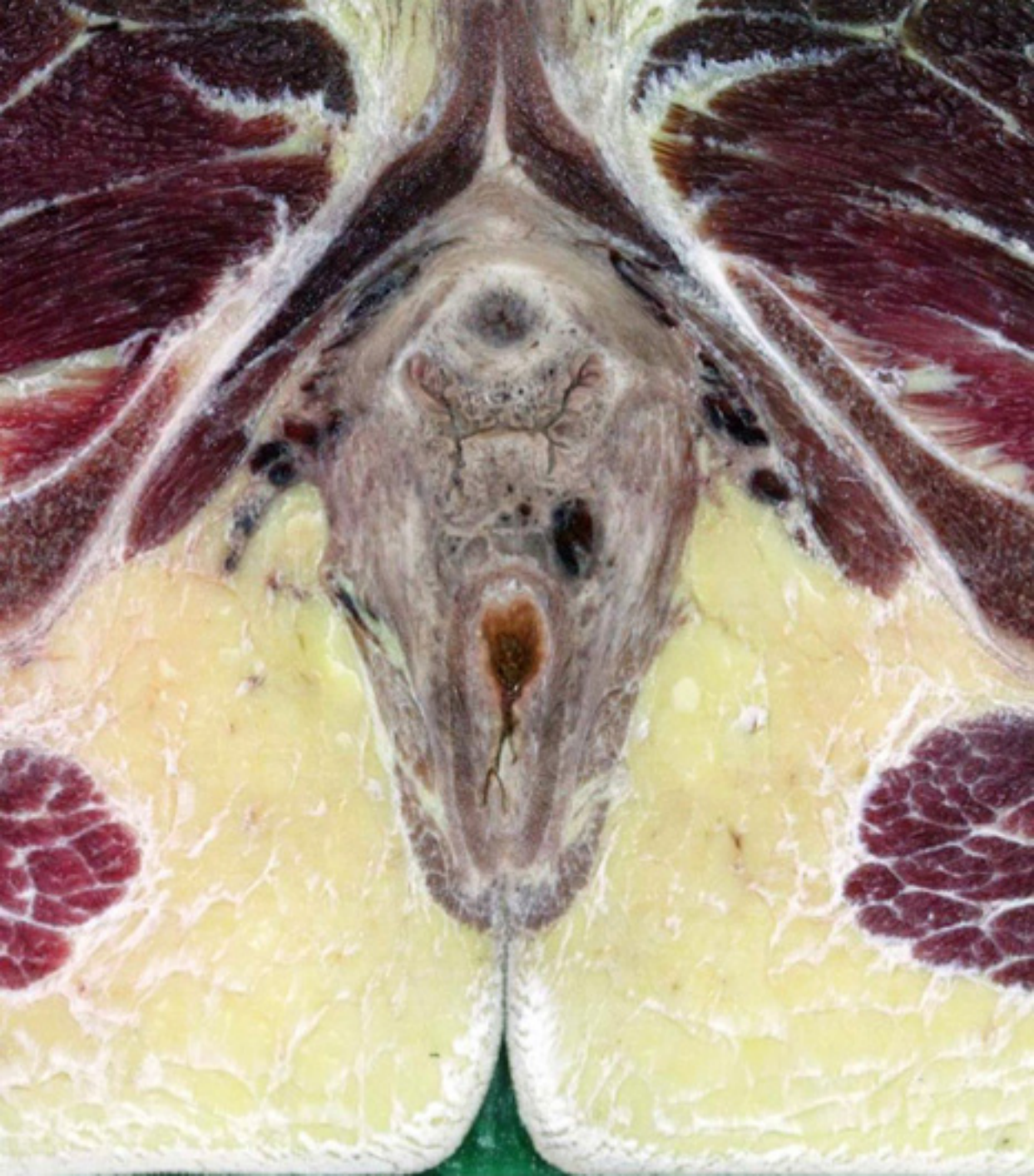

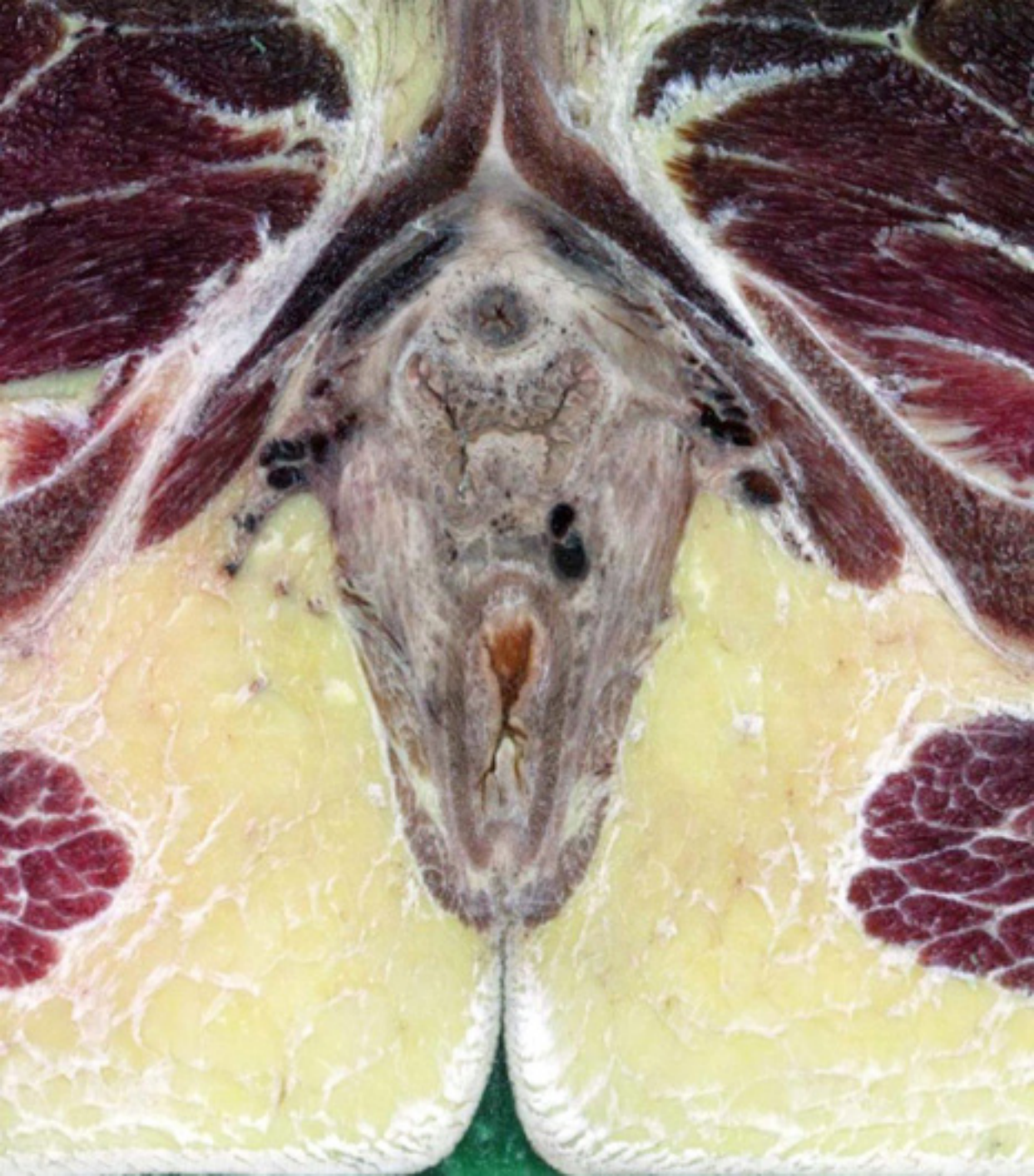

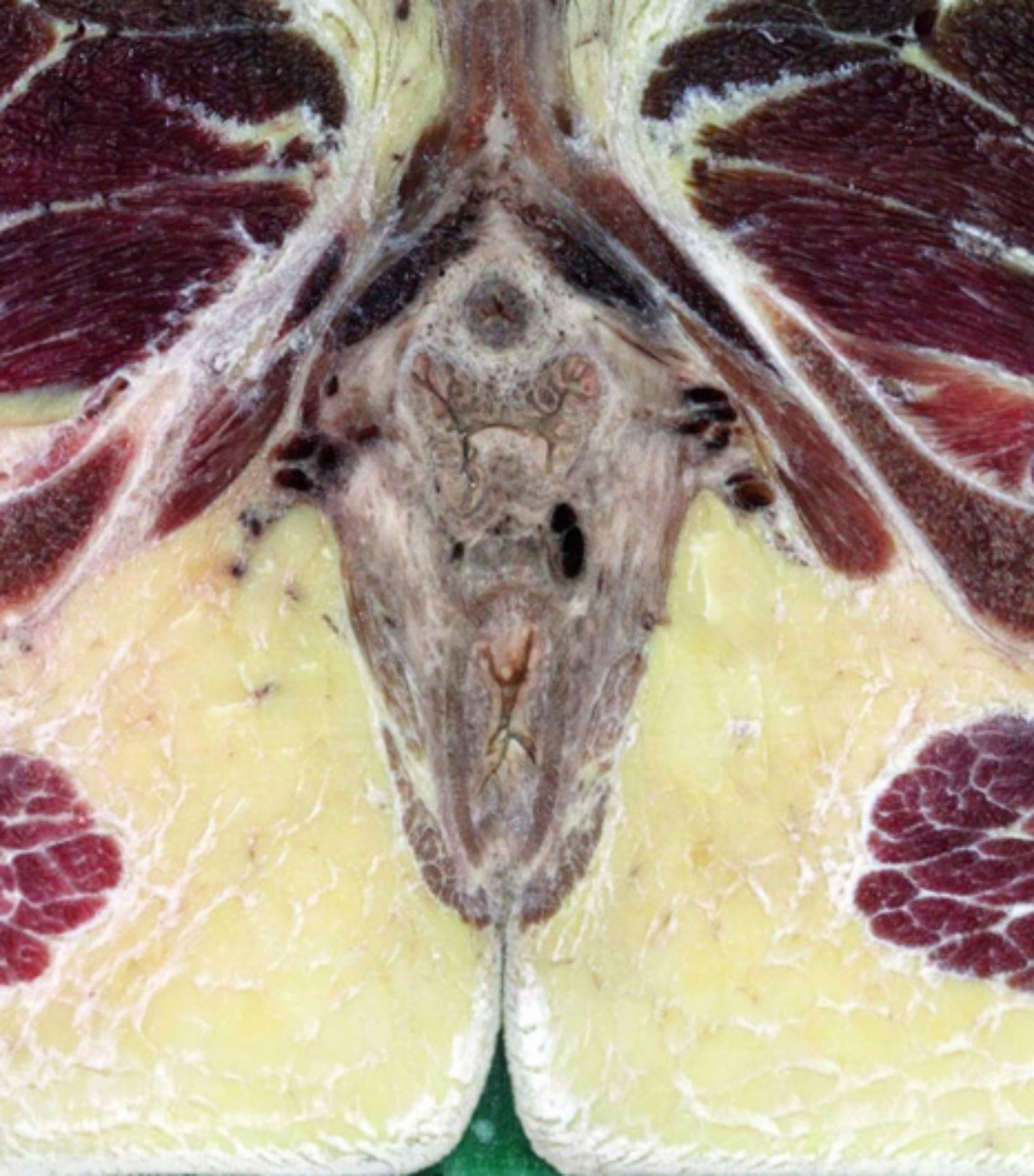

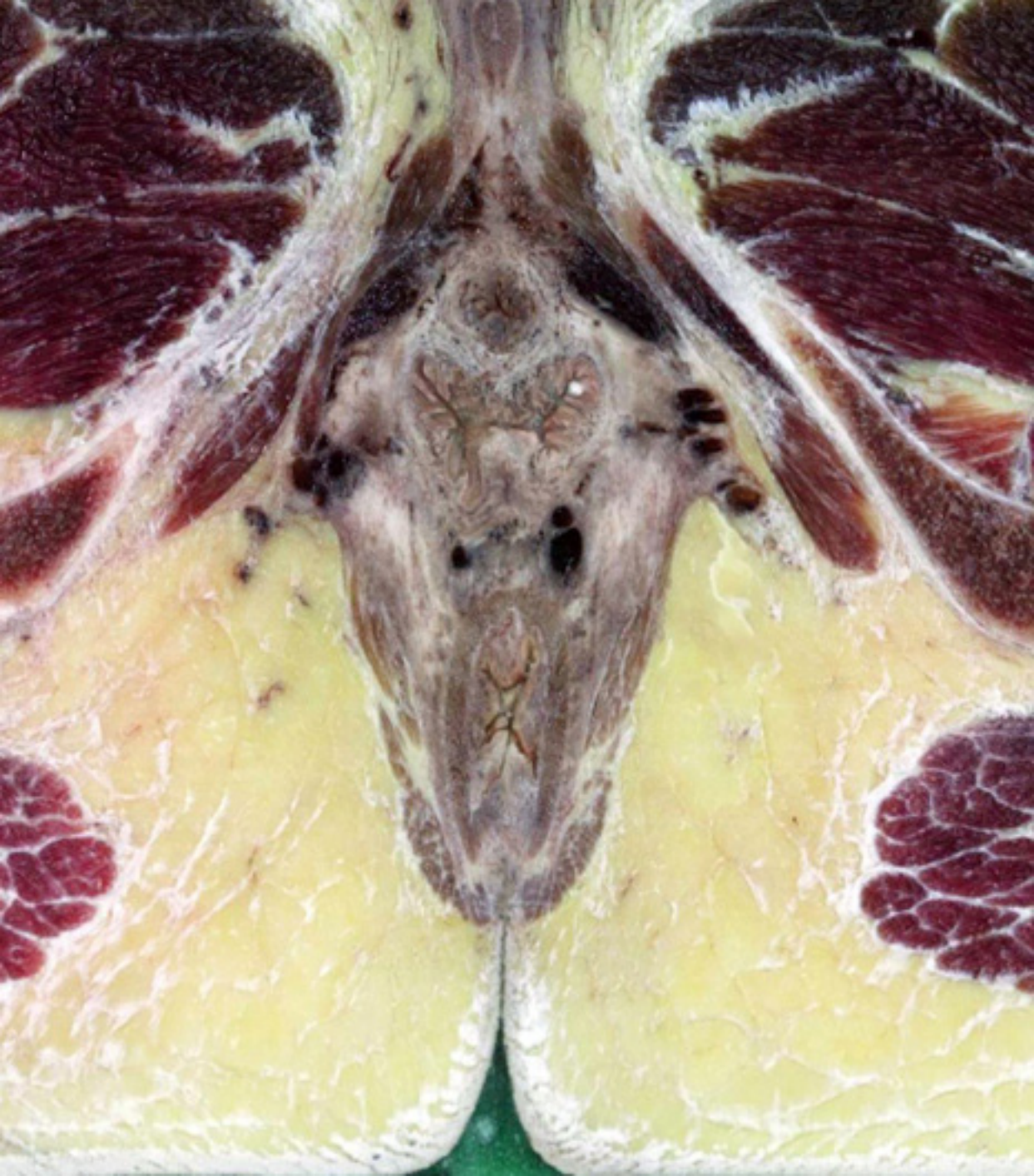

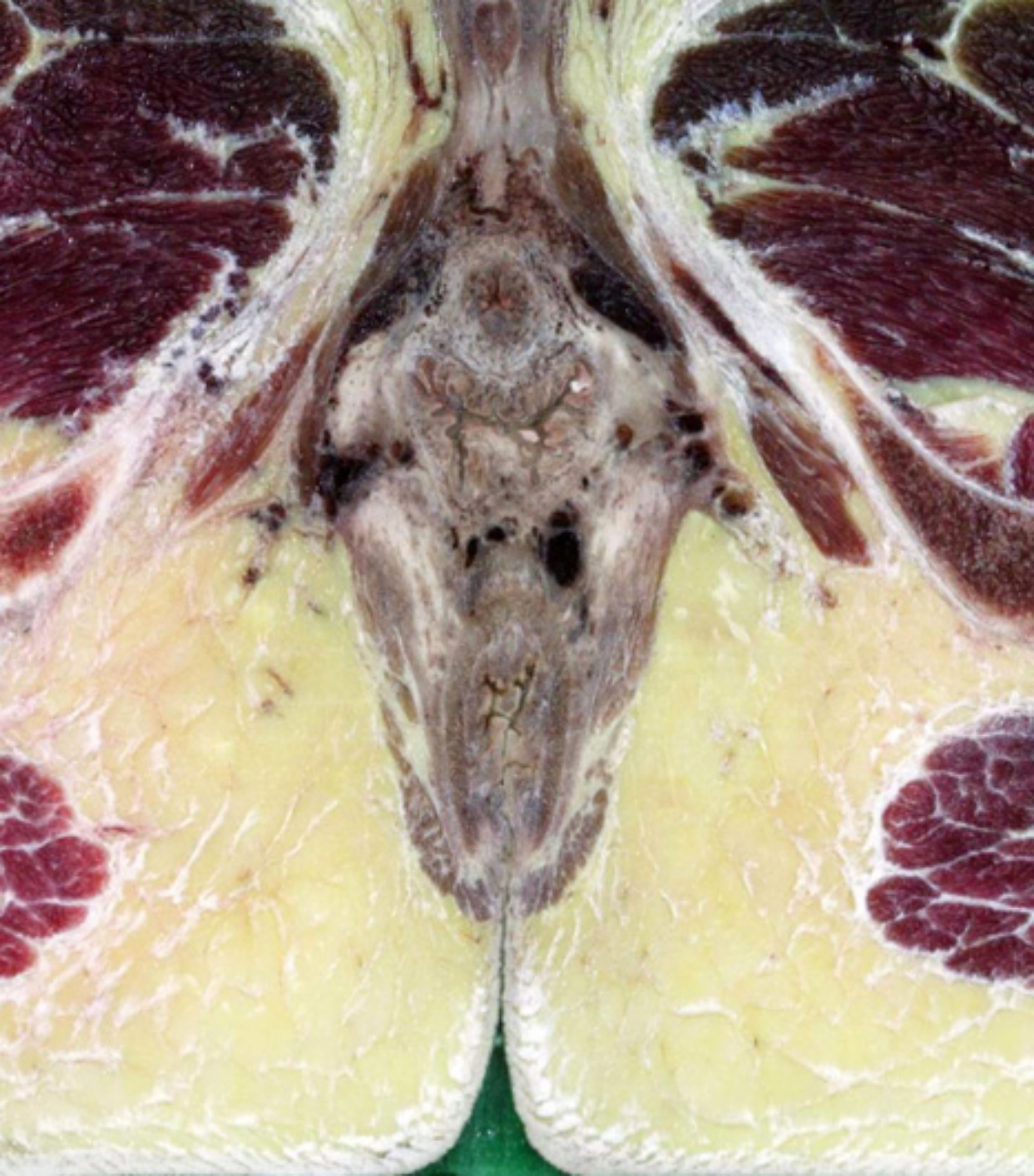

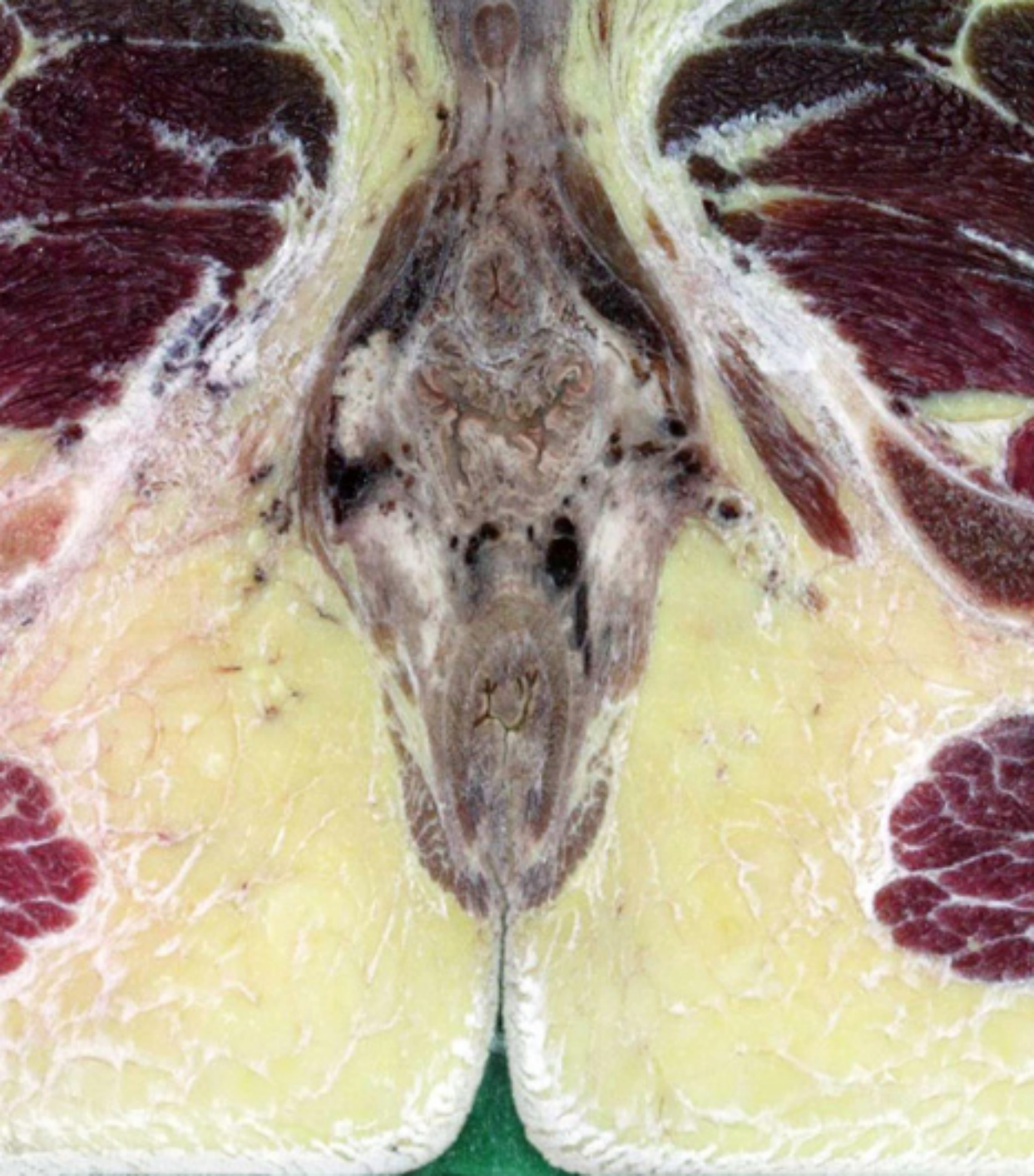

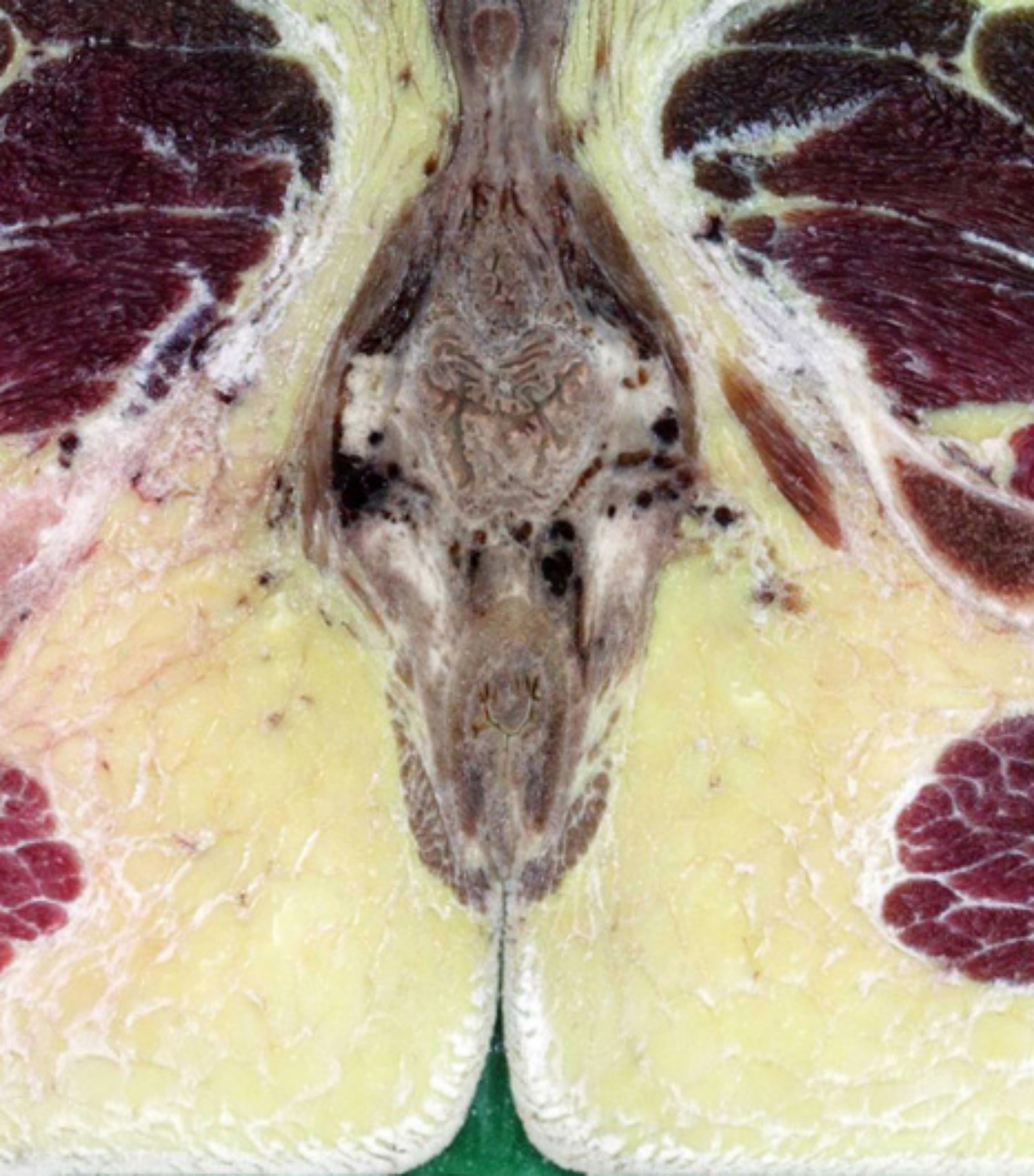

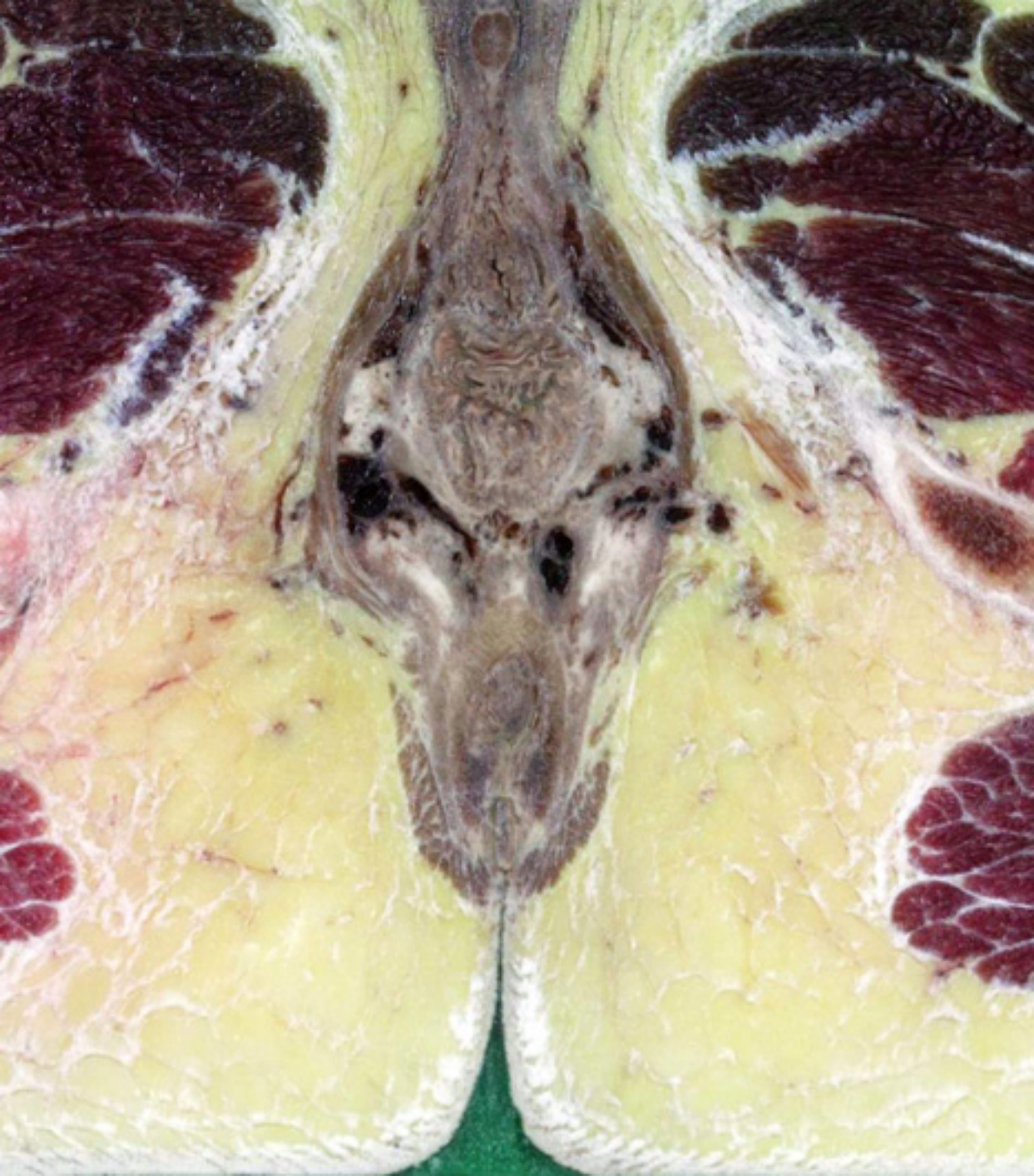

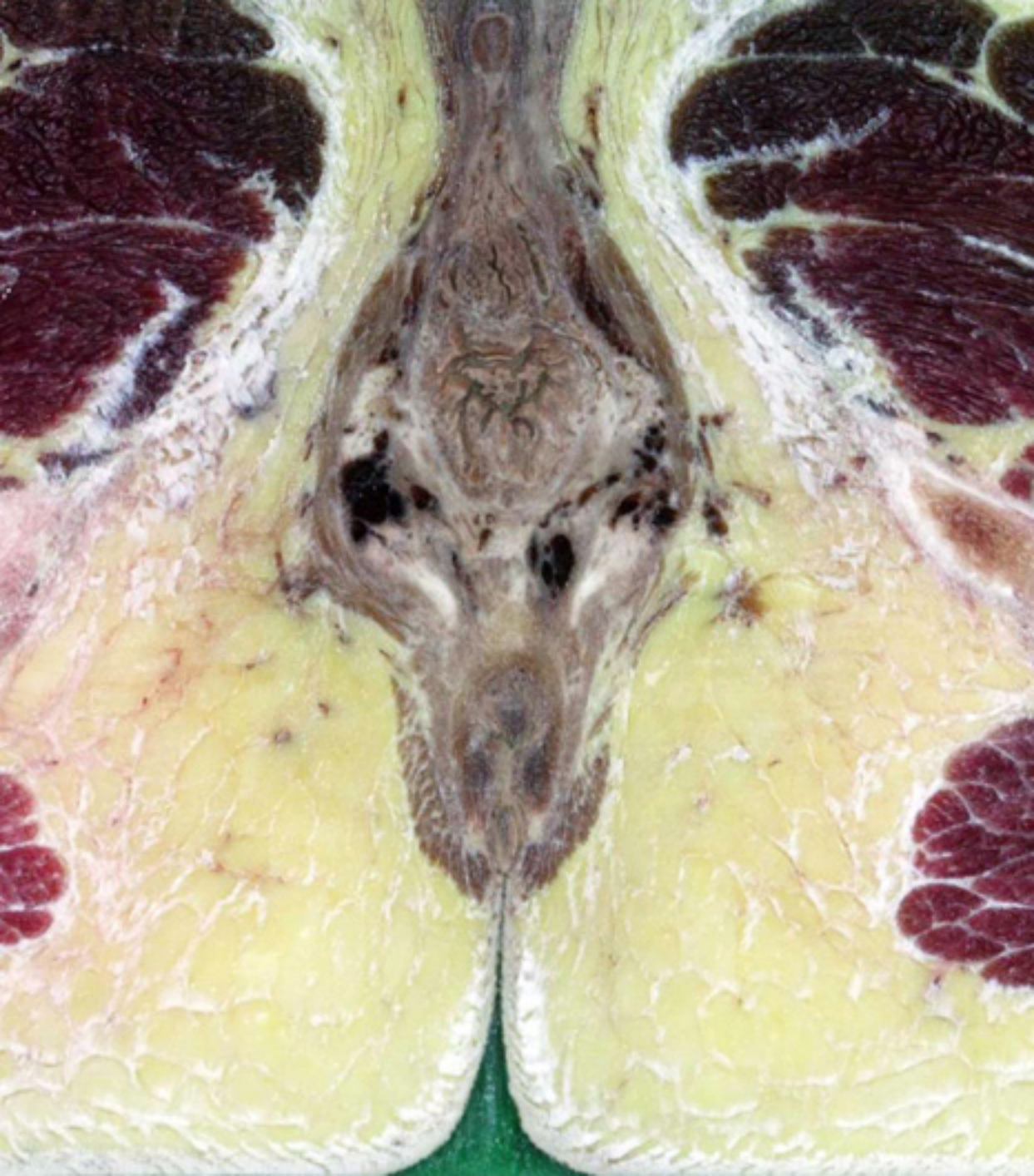

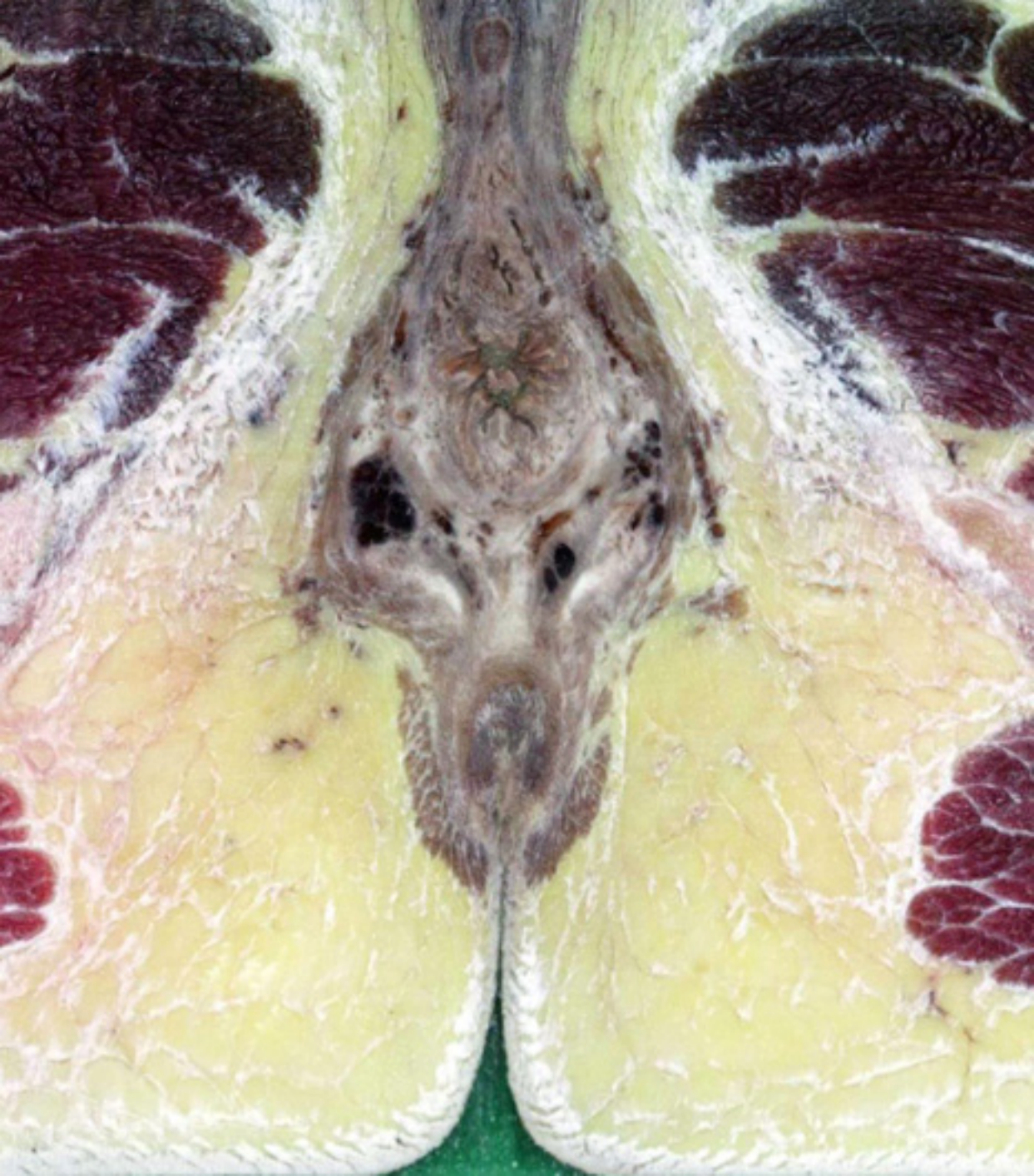

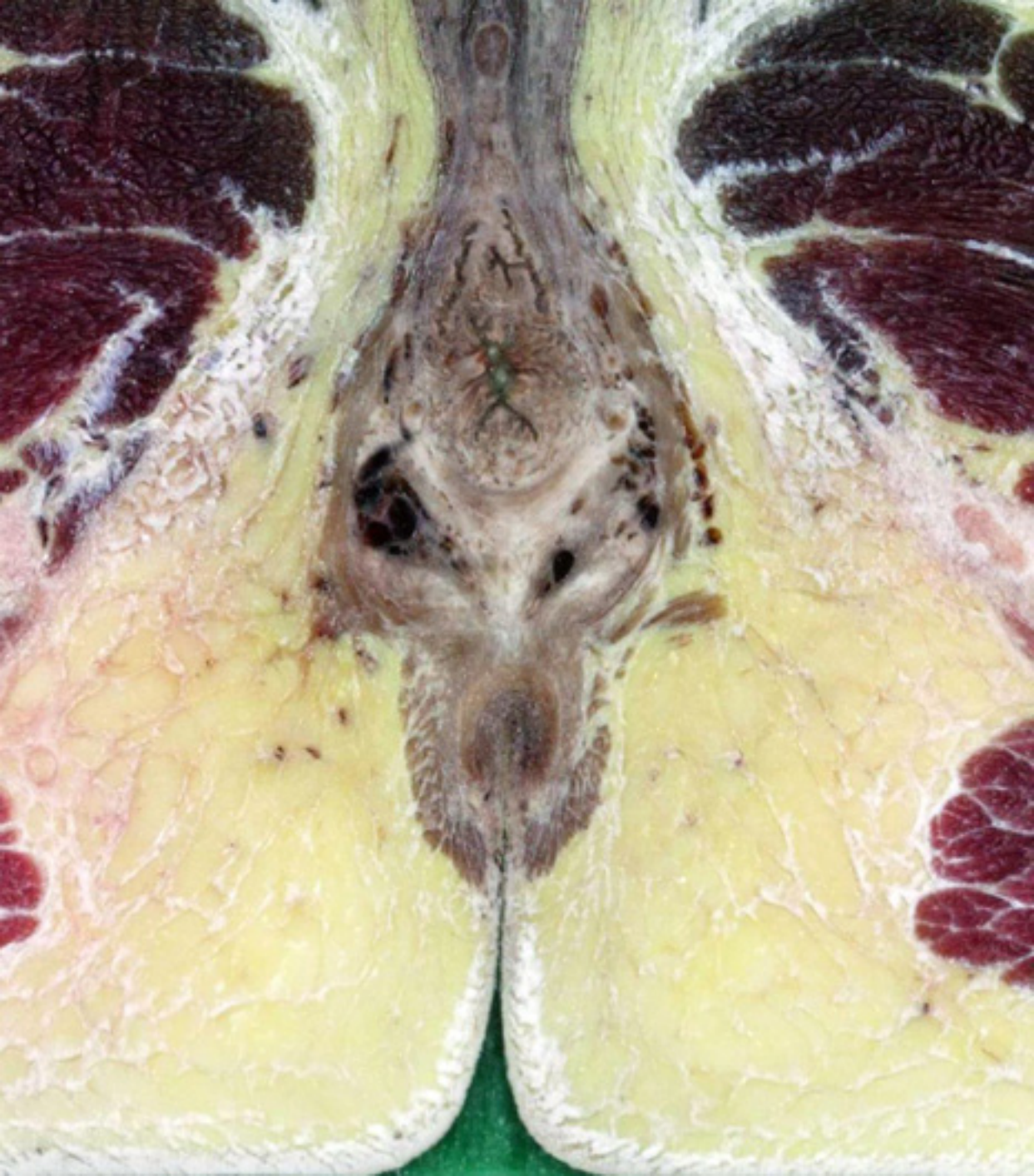

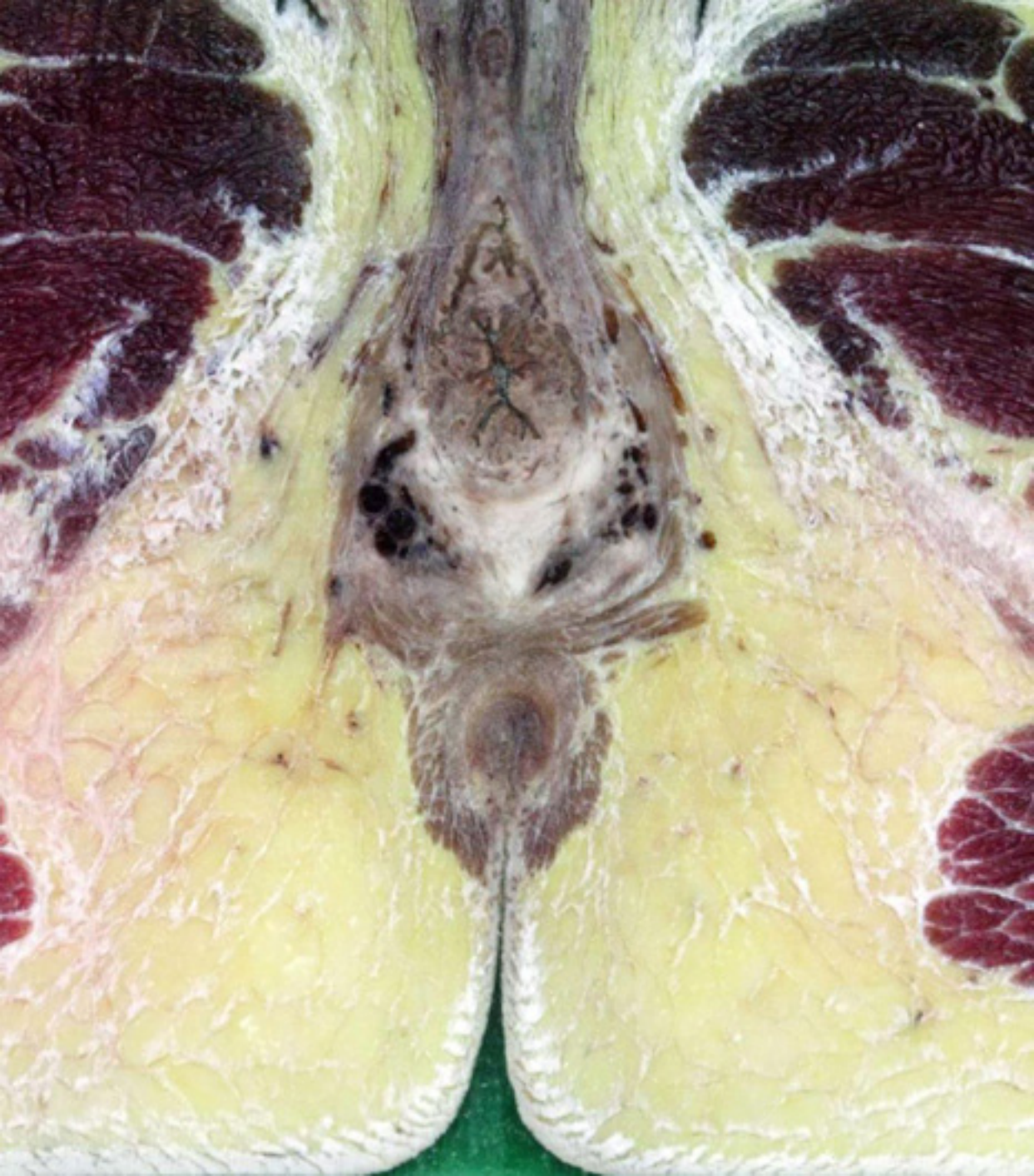

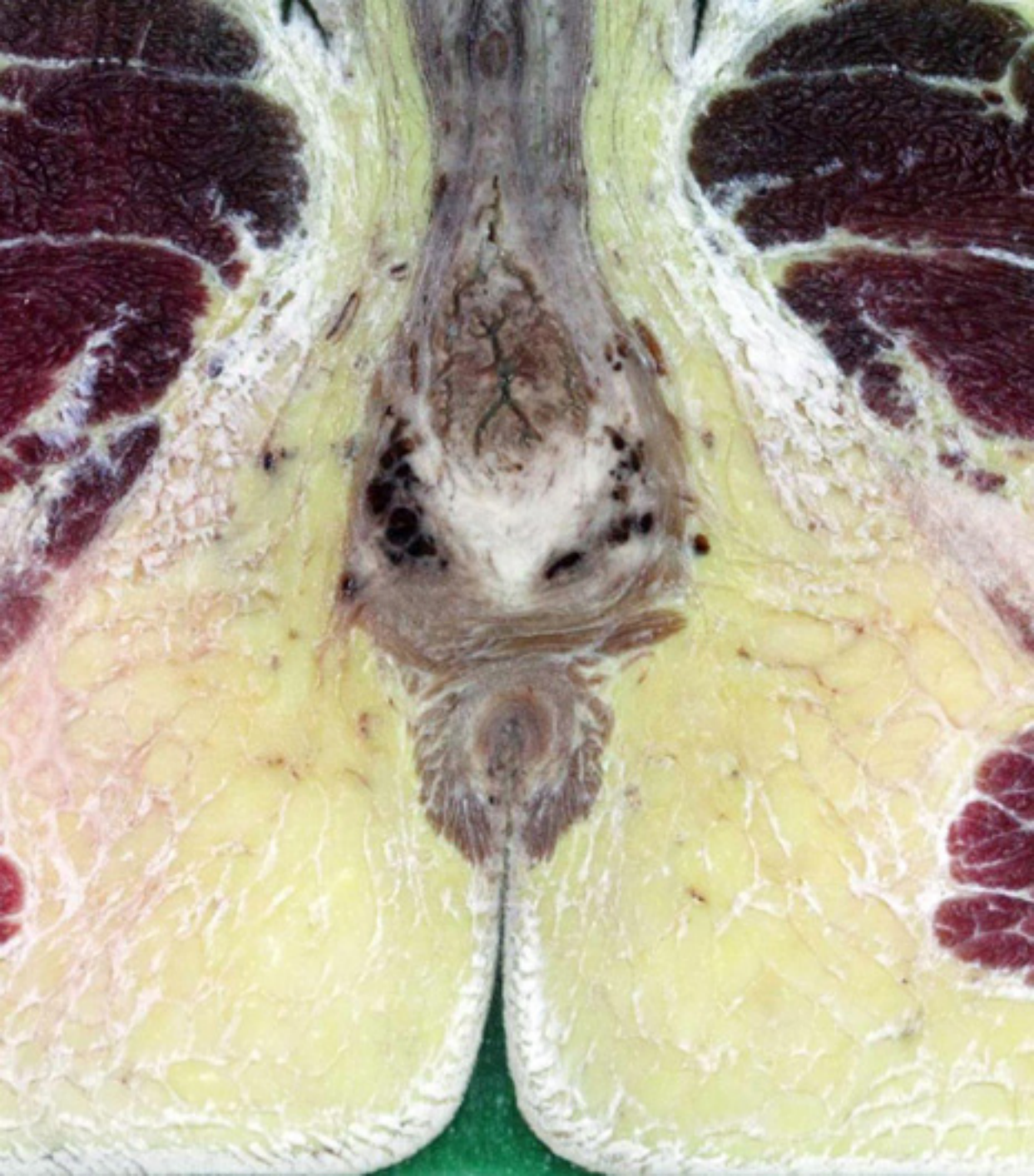

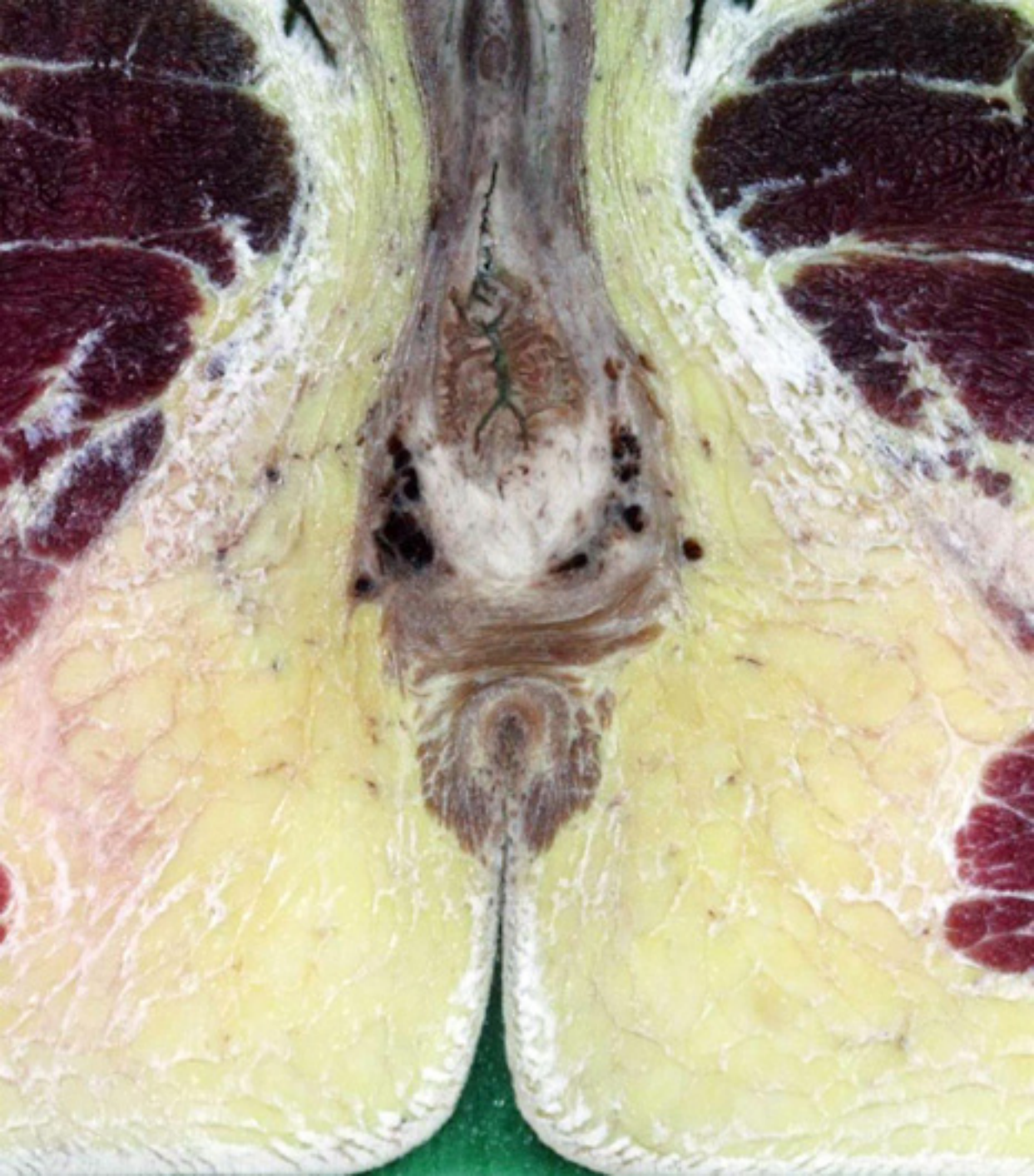

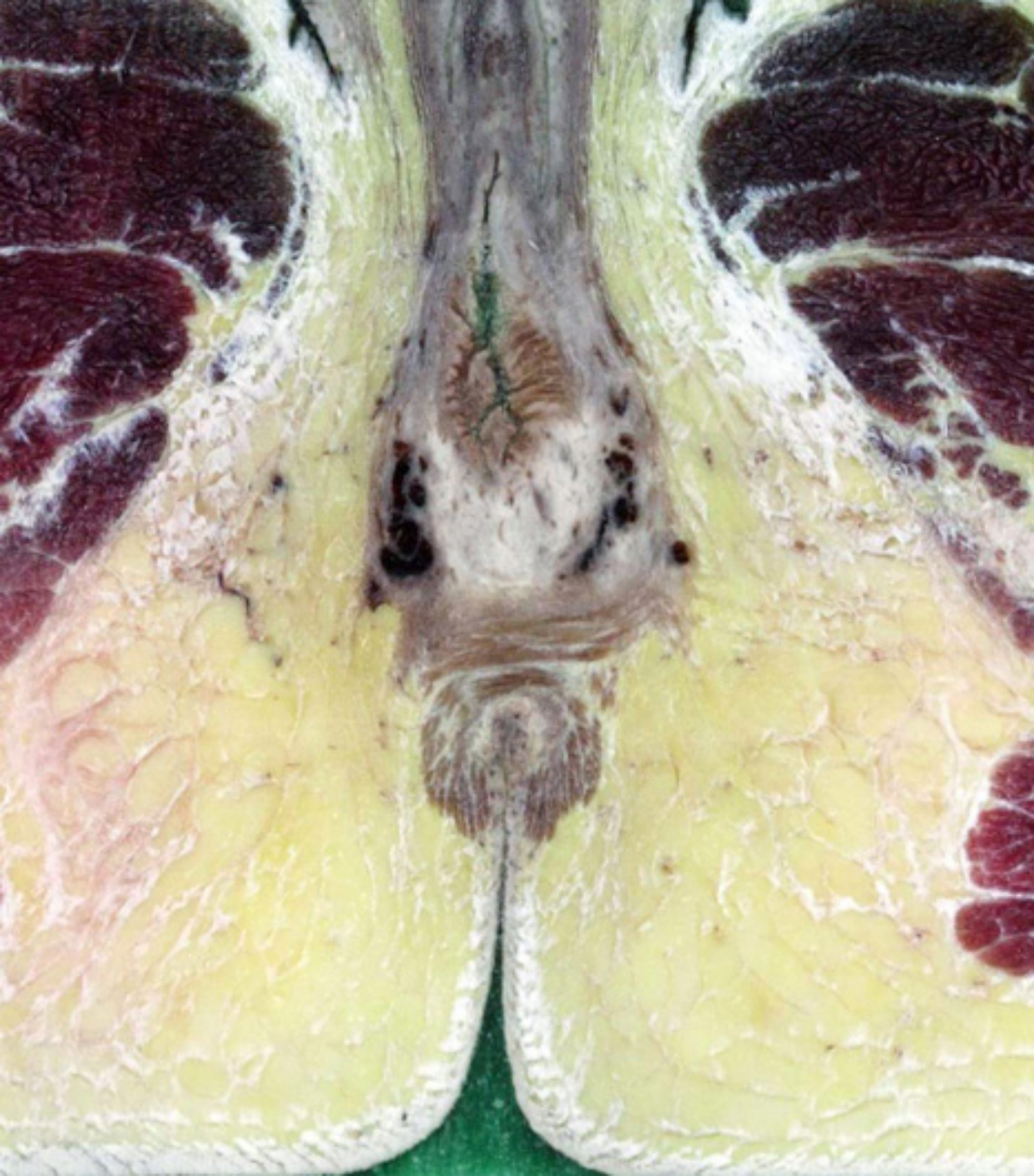

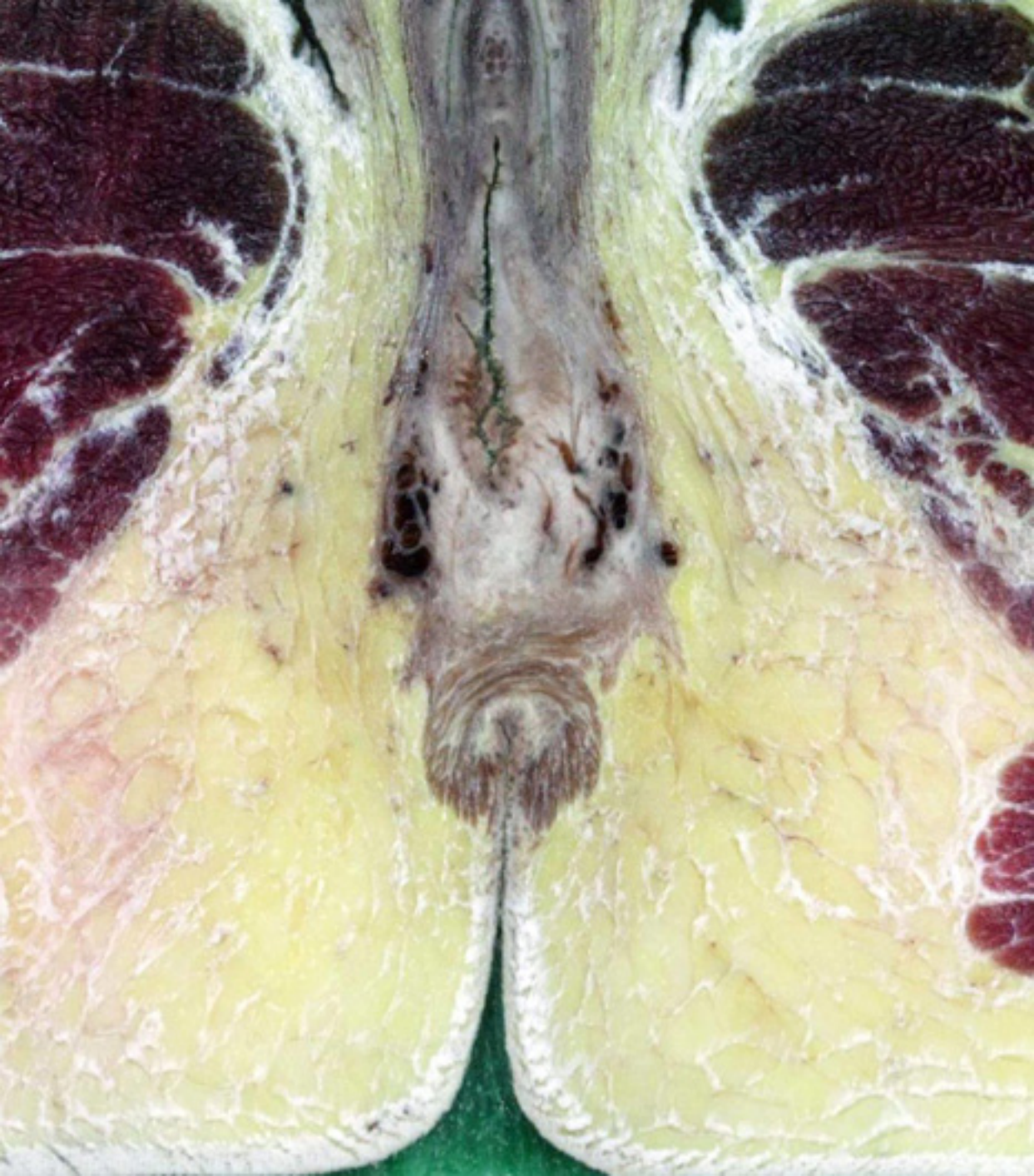

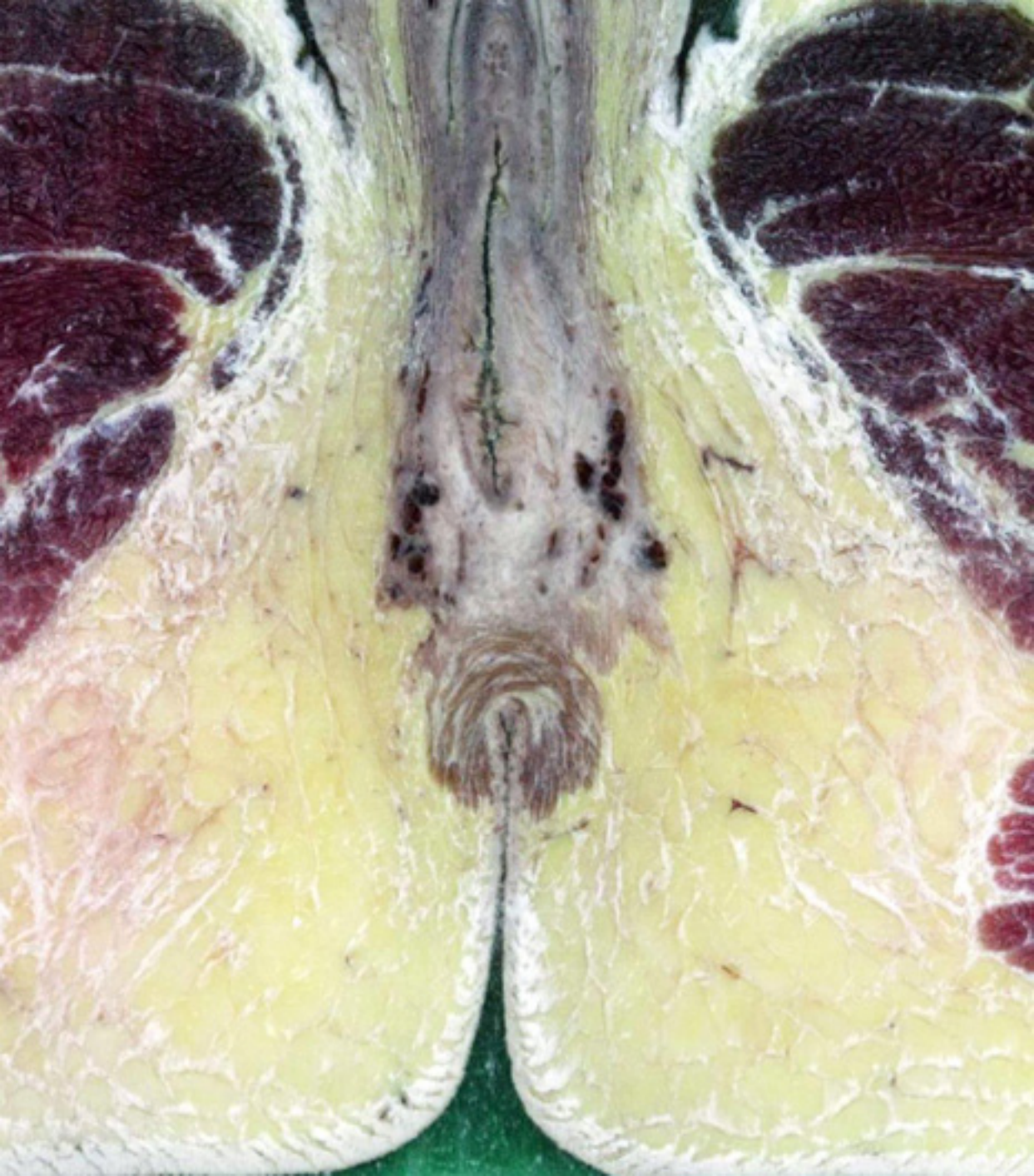

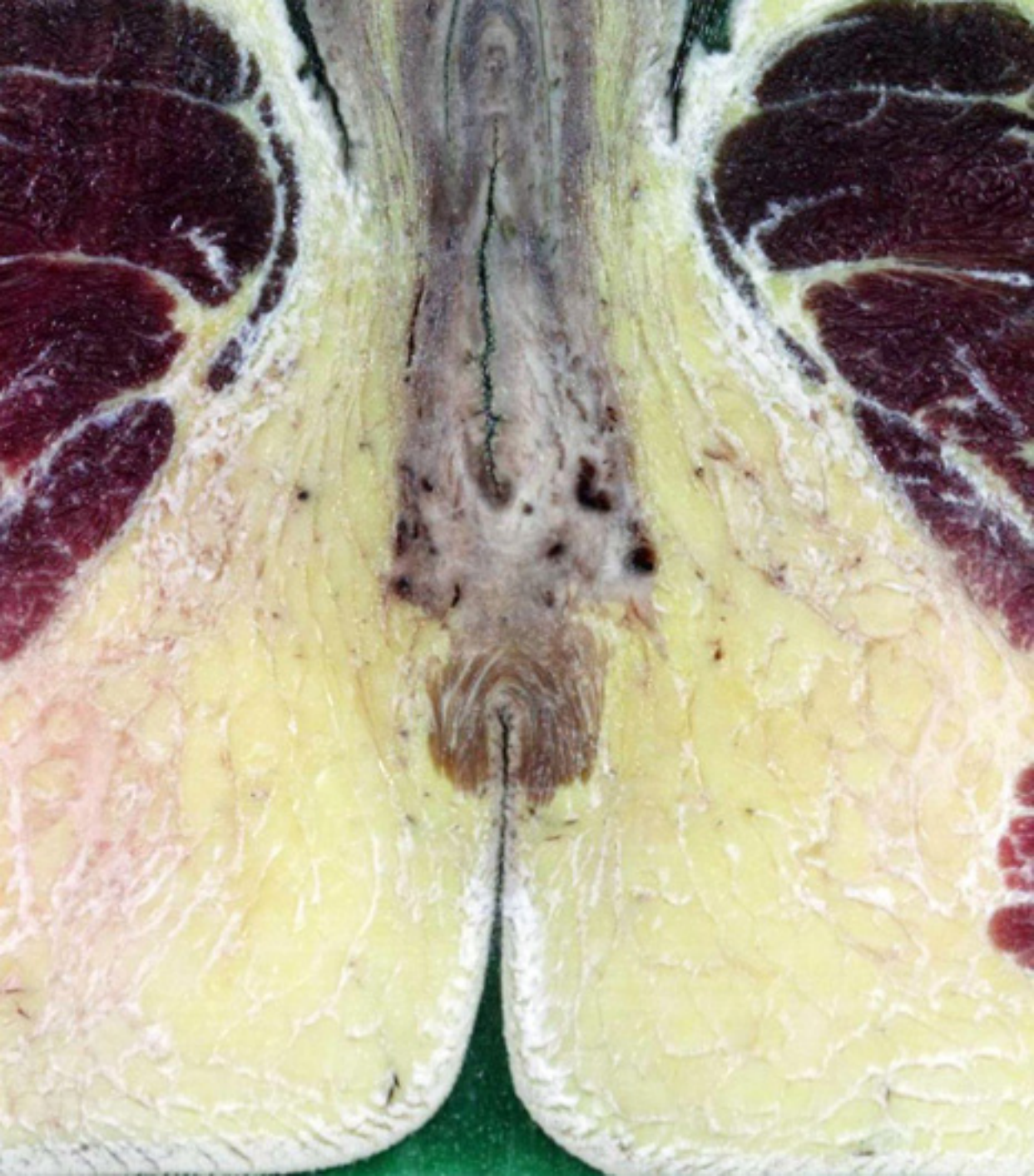

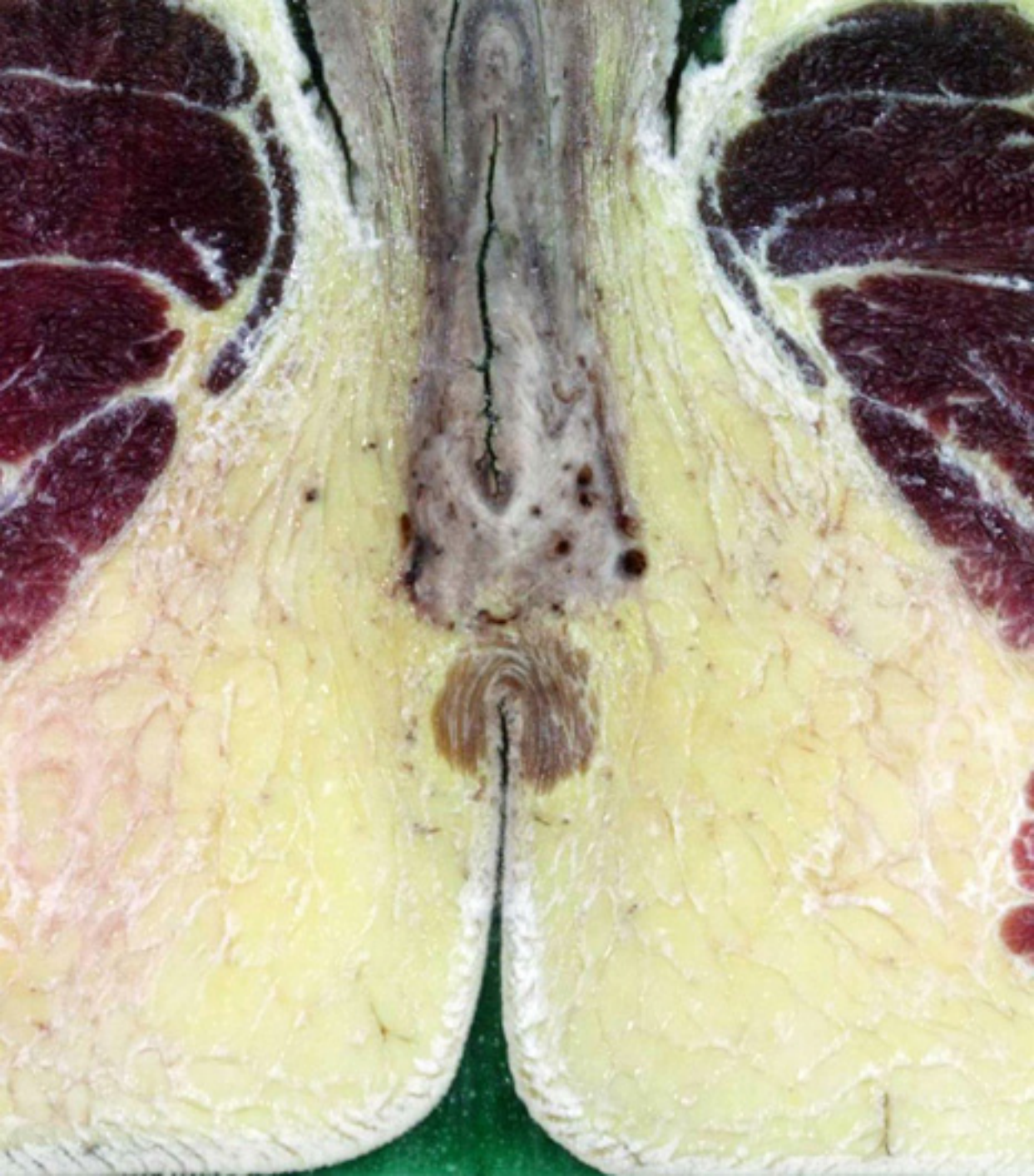

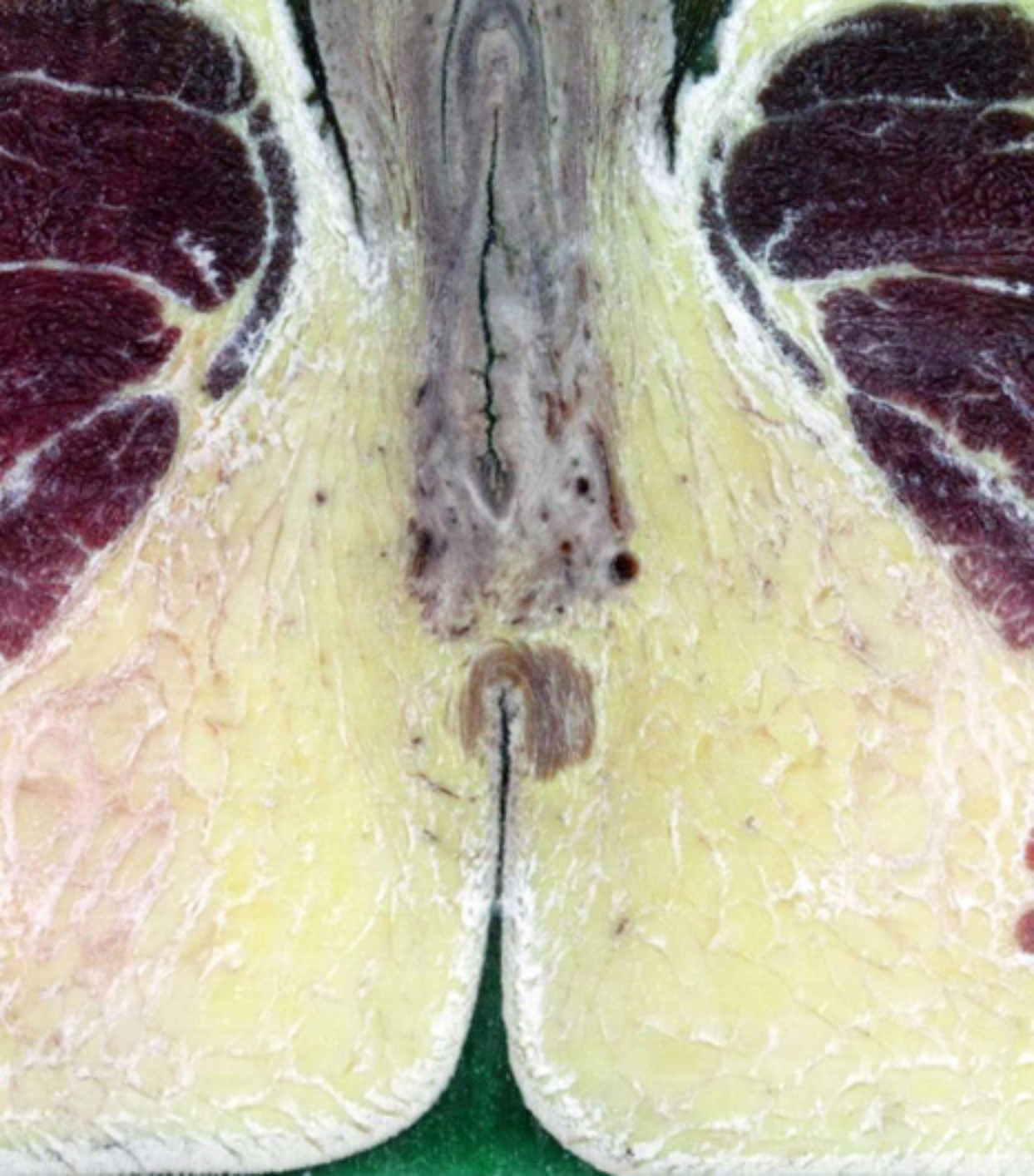

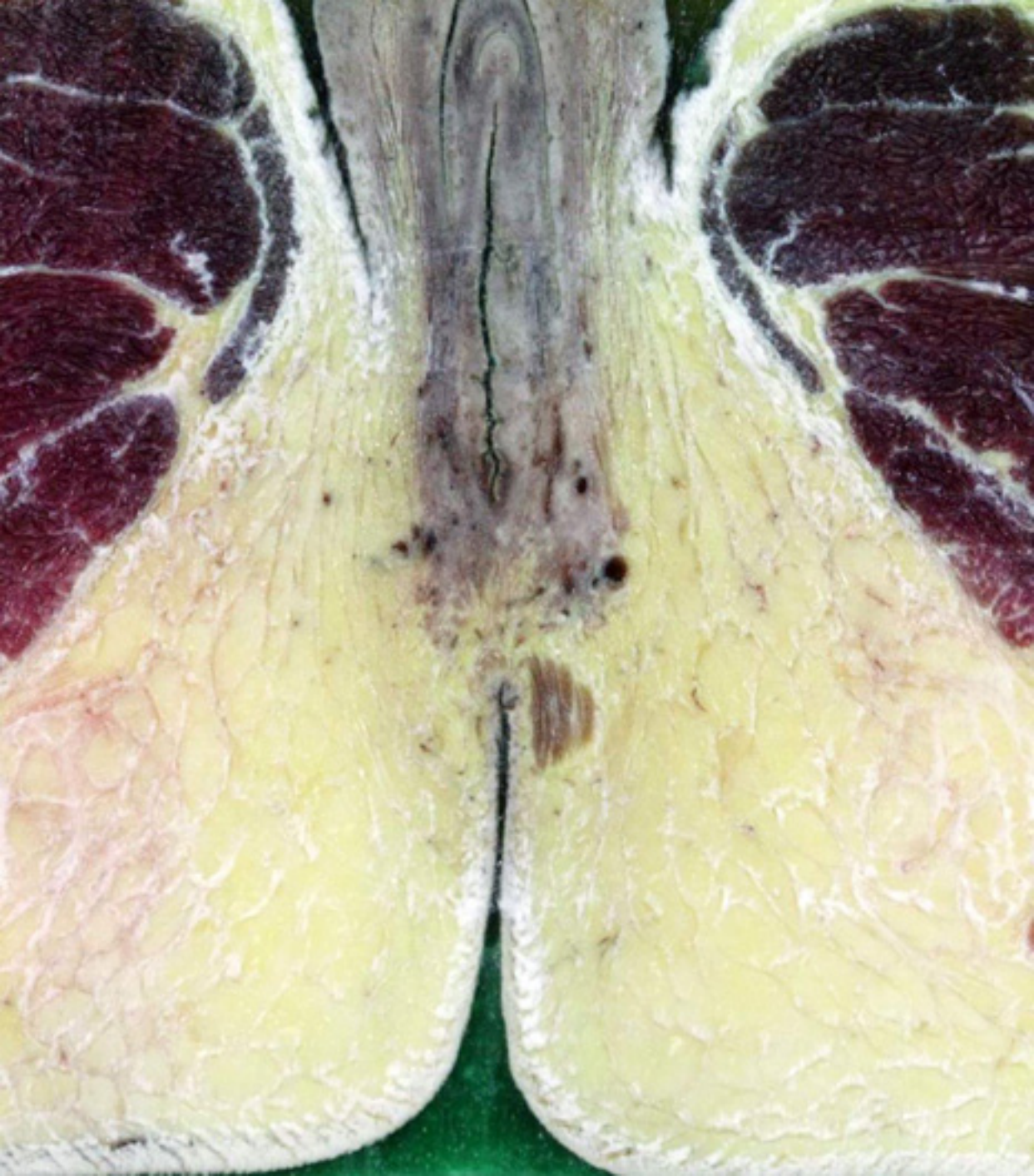

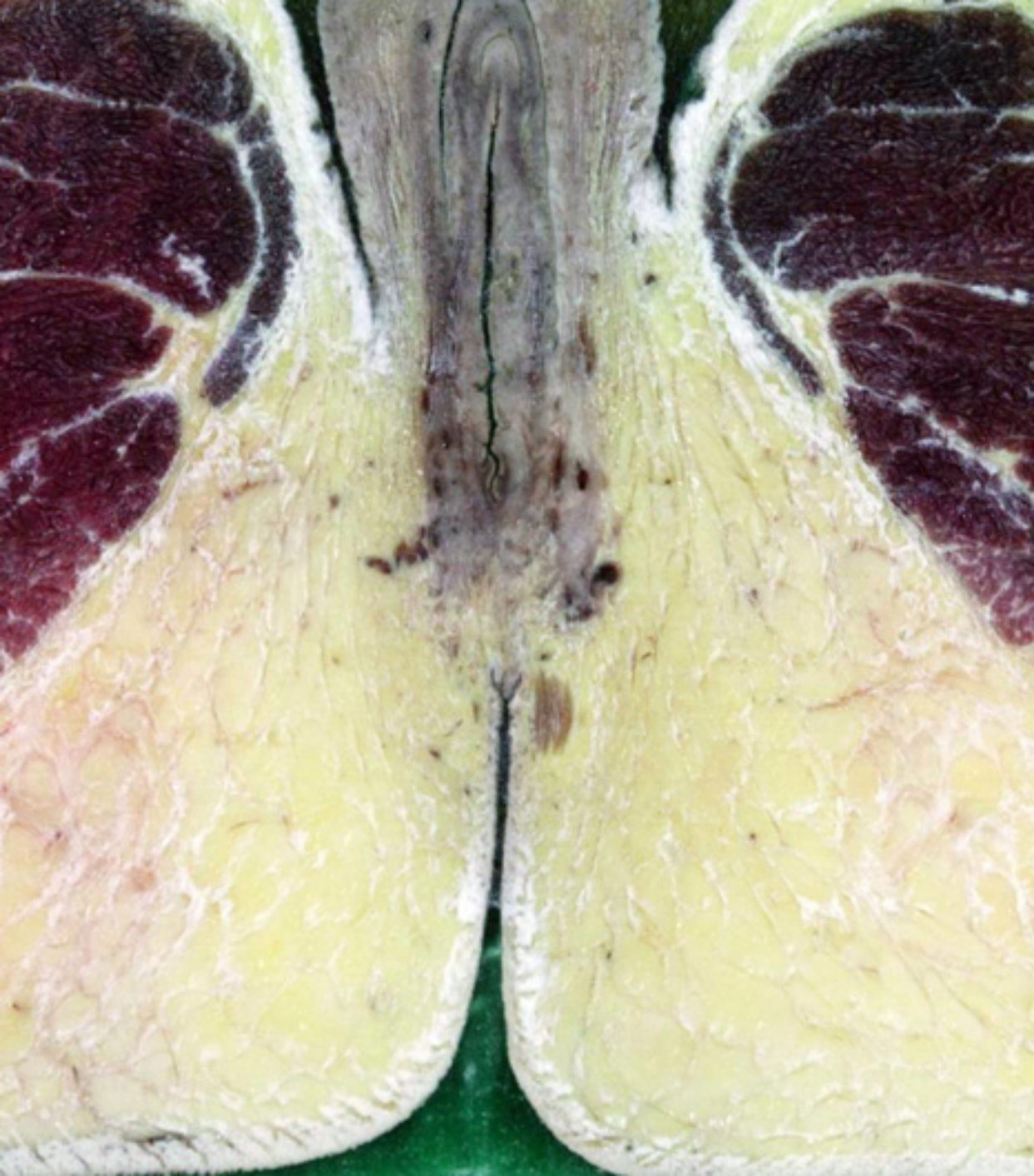

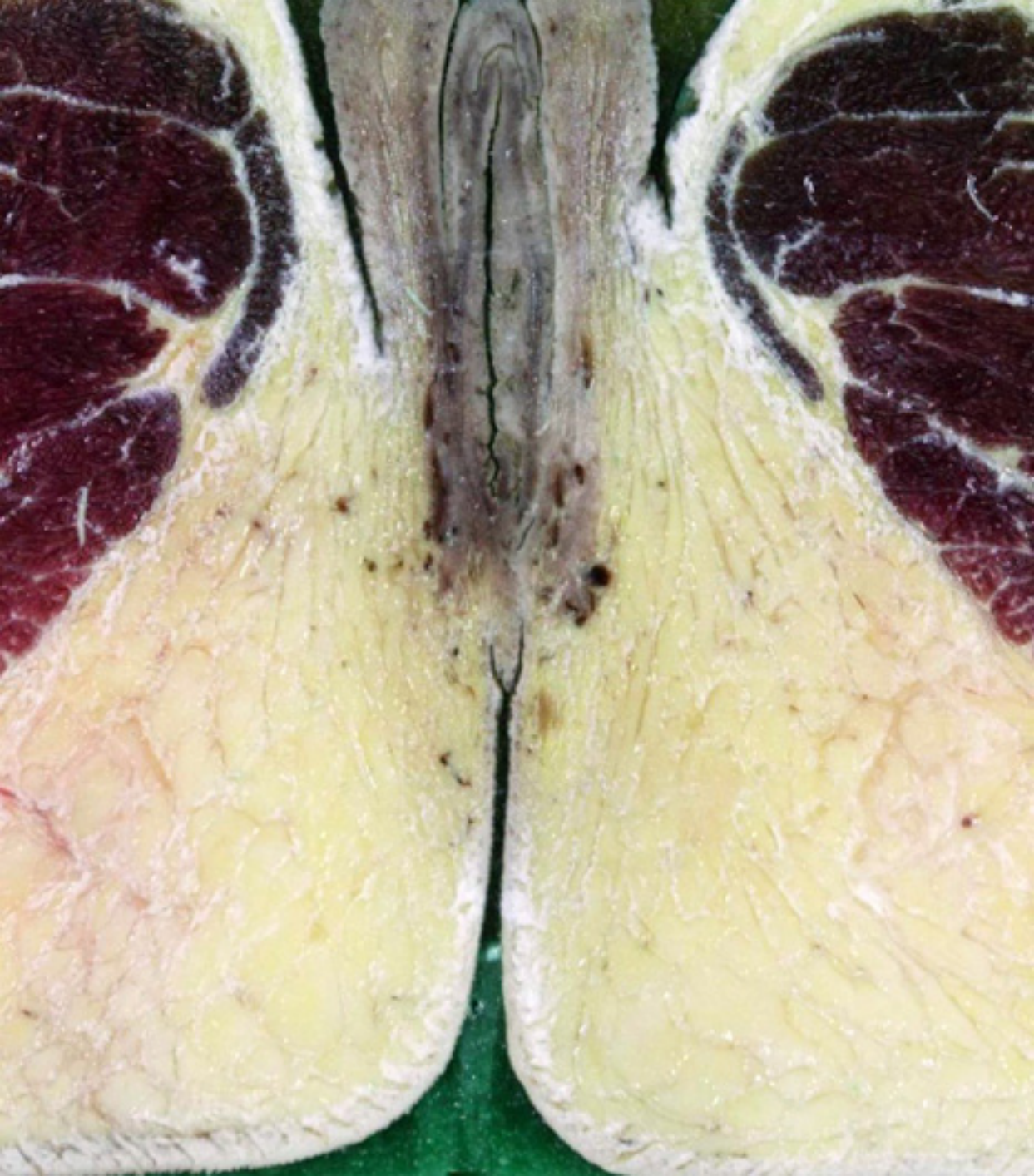

Supplement: S6 Fig — All Figures were magnified 1.3-fold. The panel labels are retained. (PDF) [file pone.0140736.s005.PDF]
